# Rules and guidelines for distancing in daily life to control coronavirus disease 2019 in Korea: 3rd version, announced on July 3, 2020

**DOI:** 10.3352/jeehp.2020.17.20

**Published:** 2020-07-13

**Authors:** Central Disaster and Safety Countermeasure Headquarters of the Republic of Korea

**Affiliations:** Ministry of Health and Welfare, Sejong, Korea; Hallym University, Korea

**Keywords:** COVID-19, Disinfection, Pandemics, Republic of Korea, Severe acute respiratory syndrome coronavirus 2

## Abstract

In Korea, the first case of coronavirus disease 2019 (COVID-19) was reported on January 21, 2020, after which the number of infected people began to increase. Intensive control measures stabilized the spread of COVID-19 in Korea. Therefore, the Korean government introduced the policy of “distancing in daily life” to support the maintenance of normal life starting on March 22, 2020. This policy provides rules and guidelines on distancing in daily life to facilitate the control of COVID-19 in Korea. “Distancing in daily life” refers to a new, sustainable way of life and social interactions that prepares society to face the possibility of long-term prevalence of COVID-19. These guidelines aim to achieve the goal of infection prevention and containment, while sustaining people’s everyday life, economic, and social activities. All members of society and communities are called upon to fulfill their respective responsibilities to combat the COVID-19 pandemic and to safeguard everyone’s health and well-being. Five key rules govern personal distancing in daily life: stay home for 3–4 days if you feel unwell; keep a distance of 2 arms’ length from others; wash your hands for 30 seconds and cough or sneeze into your sleeve; ventilate spaces at least twice a day and disinfect regularly; and stay connected while physically distancing. Collective distancing in daily life for communities and organizations is supported by these 5 key rules, and detailed guidelines are set out for different types of facilities. All individuals and communities are obliged to abide by these rules and guidelines for distancing as part of daily life.

## Introduction

This training material contains an English translation of the 3rd Korean-language version of rules and guidelines for distancing in daily life to coronavirus disease 2019 (COVID-19) in Korea, which was announced on July 3, 2020. The main goals of individual and collective infection control activities are first, to keep at bay the infiltration of the virus into living spaces; second, to remove conditions favorable for pathogen transmission and survival; third, to minimize shedding of the virus outside the body; and fourth, to trace and block transmission routes. These guidelines consist of 4 major parts: 5 key rules for personal distancing; 4 complementary actions for personal distancing; 5 key rules for collective distancing; and detailed guidelines for physical distancing in daily life for communities and organizations. The detailed guidelines are presented as supplemental material. All individuals and communities are obliged to abide by the rules and guidelines for distancing in daily life. If necessary, they can devise and implement additional guidelines tailored to their situation or conditions.

## Five key rules for individual infection control

### Rule 1: Stay home for 3–4 days if you feel unwell.

Evidence indicates that COVID-19 patients at an early stage with mild symptoms can transmit the virus. Everyone can contribute to containing the possible spread of COVID-19 by engaging in maximum social distancing from others when they have a fever or respiratory symptoms.

Action 1: Stay at home and rest for 3–4 days if you have a fever or respiratory symptoms such as cough, phlegm, sore throat, and nasal congestion.

Action 2: If you experience the above-mentioned symptoms, try your best to have as little contact with others as possible, and wear a mask when staying with others at home. In particular, refrain from contact (e.g., conversations, having a meal together) with the elderly and those with underlying health conditions.

Action 3: Return to daily life after rest when you are confident that you no longer have any COVID-19 symptoms. If a fever of 38°C or higher continues, or your symptoms deteriorate during rest, contact the Korea Centers for Disease Control and Prevention (KCDC) call center (1339, area code+120) or a local public health center.

Action 4: If you must leave home to visit a hospital/pharmacy, or to purchase living necessities, make sure to wear a mask.

Action 5: Company managers, employers, and others in a position of authority should encourage employees with COVID-19 symptoms to refrain from work or to return home to rest.

### Rule 2: Keep a distance of 2 arms’ length from others.

According to the currently available evidence, COVID-19 is primarily transmitted via respiratory droplets. Maintaining a distance of at least 2 meters from others can help limit the risk of contamination by droplets (through conversations, coughs, sneezes, etc.), thereby reducing the transmission of the severe acute respiratory syndrome coronavirus 2 (SARS-CoV-2, thereafter coronavirus) in communities.

Action 1: Try to stay away from enclosed spaces with poor ventilation or crowded places.

Action 2: Keep a distance of at least 2 m (1 m if 2 m is impossible) in everyday life.

Action 3: Arrange seats to ensure sufficient distancing between people.

Action 4: For unavoidable gatherings of large groups, obtain a space large enough for 2-m distancing, or adjust the timing of gatherings so that meetings do not overlap.

Action 5: Do not shake hands with or hug people.

### Rule 3: Wash your hands for 30 seconds. Cough or sneeze into your sleeve.

Good personal hygiene is important for preventing the virus from entering your body via contaminated hands. By maintaining proper cough etiquette, you can also minimize the possibility of droplet-based transmission.

Action 1: Wash your hands for at least 30 seconds with running water and soap, or clean them with hand sanitizer, before meals, after using the toilet, after returning home from outside, or after blowing your nose, coughing, or sneezing.

Action 2: Do not touch your eyes, nose, or mouth with unwashed hands.

Action 3: Ensure that sinks and soaps are available in private and public spaces to facilitate handwashing, or make hand sanitizer easily accessible and always within sight to promote its use.

Action 4: When you cough or sneeze, cover your nose and mouth with a bent elbow or tissue.

Action 5: If you have COVID-19 symptoms (such as fever, cough, phlegm, sore throat, or nasal congestion) or feel physically unwell, wear a mask for the safety of other people.

### Rule 4: Ventilate spaces at least twice a day and disinfect regularly.

Ventilation of living or working spaces can lower the concentration of coronavirus-containing saliva droplet particles in the air. Furthermore, disinfecting objects or surfaces on which infectious droplets could have landed is a key method that can curb the possibility of COVID-19 infection via the hands.

Action 1: If a space can be ventilated naturally, keep the windows open all the time. If the windows cannot remain open, ventilate the space regularly (at least twice a day). If possible, keep both the door and windows open while ventilating. Indoor ventilation is necessary regardless of exposure to fine particulate matter pollution.

Action 2: Always keep daily spaces (house, office, etc.) clean and disinfect the surfaces of high-touch objects, such as phones, remote controls, handles, doorknobs, tables, armrests, switches, keyboards, computer mice, and copiers, at least once a week.

Action 3: In public spaces and any other areas frequented by crowds, the surfaces of high-touch objects (elevator buttons, doors, handles, handrails, doorknobs, armrests, switches, etc.) and shared objects (shopping carts, etc.) must be disinfected daily.

Action 4: When disinfecting, make sure to comply with the instructions of the manufacturer, including the proper amount and safe usage, for each type of disinfectant. Examples of disinfectants include disinfectant wipes, alcohol (70% ethanol), and sodium hypochlorite (also known as household bleach).

### Rule 5: Stay connected while physically distancing.

The COVID-19 pandemic cannot be overcome by acting alone; only a strong and unified communal response can tackle the massive challenges posed by the virus. The best winning strategy for all is to work together in the spirit of solidarity, compassion, and love for one another.

Action 1: Remember to stay in touch with family and loved ones despite physically being apart.

Action 2: Engage yourself in building up a caring and sharing community. Raise your voice against discrimination and stigmatization toward COVID-19 patients, people in quarantine, and other vulnerable groups.

Action 3: Take actions and share thoughts to support socially vulnerable groups who are particularly susceptible to being left behind in times of the pandemic.

Action 4: When you hear suspicious information, check the source to make sure it is trustworthy. Do not spread rumors or misinformation and refrain from excessive media consumption.

## Four complementary actions for personal distancing

### Mask wearing

#### General principles and proper techniques of mask wearing

General principles: The use of masks can play a role in preventing and limiting the spread of coronavirus infection via saliva droplets. Nonetheless, it is worth emphasizing that the use of a mask alone is insufficient to prevent transmission of COVID-19, and masks must be accompanied by other infection prevention and control measures such as frequent hand-washing and physical distancing between people. When there is a risk of infection, high-risk individuals with underlying health conditions are recommended to wear medical or surgical masks. If medical or surgical masks are unavailable, wearing cloth masks can be helpful as well.

How to properly wear a mask: Select an appropriately sized mask for your face and attach the mask securely to the face, ensuring that it completely covers your nose and mouth. Wash your hands thoroughly before touching the mask to prevent contamination of the mask itself. While wearing the mask, try your best not to touch it with your hands to limit the risk of contamination via the hands. If you touch the mask by mistake, wash your hands for at least 30 seconds with soap and water or hand sanitizer. After using a mask, remove it without touching its front. Immediately discard a used medical mask into a waste bin (not leaving it unattended) and wash your hands. Cloth masks should be washed frequently according to the proper washing method for each product. Take care to avoid placing a tissue or a towel on the inside of a mask, as it can lead to diminished effectiveness in blocking pathogens due to air leakage or reduced tightness. A mask can be re-used only if it is being used temporarily by the same person in a place with a low risk of contamination.

#### When the use of a mask is recommended

It is advised to wear a mask in the following situations: taking care of persons suspected to be infected with COVID-19 (wear a mask rated KF94 [Korea filter 94] or higher); upon developing respiratory symptoms such as cough, sneeze, phlegm, runny nose, and sore throat; when visiting a medical institution, pharmacy, or facility for the elderly or disabled; when working in a profession that entails working directly with the public, for example, occupations in the service sector that involve physical contact with customers (such as sales assistants, restaurant workers, and customer service representatives), public transportation operators, train station workers, mail carriers, couriers, and janitors of large buildings; when those in high-risk groups (seniors, children, pregnant women, patients with chronic diseases, etc.) or with underlying health conditions (chronic lung disease, diabetes, chronic renal disease, chronic liver disease, chronic cardiovascular disease, blood cancer, cancer patients undergoing chemotherapy, or patients taking immune checkpoint inhibitors) come into contact with others within 2 m in a poorly ventilated space (e.g., mass gatherings, traveling by public transportation, etc.); when using an indoor multi-purpose facility (e.g., libraries, museums, sports facilities, movie theaters, shopping malls, or public transportation); and, being outdoors where 2-m distancing cannot be ensured

#### When the use of a mask is not recommended

The use of a mask is not advised when you are outdoors where proper distancing can be ensured, or when you spend time alone without meeting anyone. Children aged under 24 months, as well as those who are unable to remove a mask by themselves without help from others or have difficulties in breathing when wearing a mask, should not wear a mask.

### Disinfection of the environment

#### General principles

Disinfection performed following proper methods and recommended procedures can ensure the effective and safe removal of pathogens.

When disinfecting a space, make sure to ventilate the area by keeping windows open. Staff who conduct cleaning or disinfection should wear proper personal protective equipment, such as disposable gloves, masks, and, if necessary, disposable waterproof long-sleeved gowns (or waterproof aprons), and goggles (or face shields).

A proper disinfectant (disinfectant wipes, alcohol [70% ethanol], diluted sodium hypochlorite [also known as household bleach] and other equivalent products) approved by or registered to the Ministry of Environment must be used. Make sure to comply with the manufacturer’s instructions and recommendations, since excessive or improper use of disinfectants may be hazardous to one’s health.

When using sodium hypochlorite, prepare the solution (500 to 1,000 ppm) by diluting it just before disinfection. Rub the target area with a cloth that is sufficiently wet with the prepared solution, leave it for at least 10 minutes, and then wipe it with a cloth that has been dampened with clean water.

- To make a 500 ppm solution, pour 5 mL of sodium hypochlorite into an empty 500 mL PET bottle (well-cleaned and dried), fill the rest of the bottle with cold water, and mix well.

- To make a 1,000 ppm solution, pour 10 mL of sodium hypochlorite into an empty 500 mL PET bottle (well-cleaned and dried), fill the bottle with cold water, and mix well.

Use a cloth that has been sufficiently soaked with disinfectant, or a disinfectant wipe.

- Caution must be exercised when spraying a disinfectant into the air. This practice creates a risk of inhaling infectious aerosols, which is hazardous to one’s health. The sprayed area must also be wiped because the effect of disinfection may otherwise remain uneven due to the unclear scope of contact between the disinfectant and applied surfaces. After disinfection, remove your gloves and wash your hands with soap and water.

#### Disinfect everyday spaces such as home and offices according to the following guidance.

Disinfect spaces at least once a week, with particular attention to the surfaces of high-touch objects such as phones, remote controls, doorknobs, handles, tables, armrests, switches, keyboards, computer mice, and copiers. Children’s toys should be wiped with a clean cloth after disinfection to remove any remnants of the cleaning solution.

#### Areas frequented by many people (such as public places) should be disinfected according to the following guidance.

It is particularly important to thoroughly disinfect the surfaces of high-touch objects including doorknobs, handrails, handles, armrests, and switches. Clean and disinfect particularly high-contact areas, such as the entrance doors of a building and elevator buttons, at least once a day. Facility managers should provide cleaning and disinfecting staff with sufficient quantities of equipment for cleaning, disinfection, and personal protection (disinfectants, paper towels, masks, etc.). For more detailed information on disinfection, please refer to “Disinfection guidelines to prevent the spread of COVID-19 at public facilities and multi-purpose facilities” (edition 3-1) (Supplement 1).

### Guidelines for seniors and high-risk groups

#### General principles

Older people (aged 65 years and above), as well as particularly high-risk groups, must take extra caution to stay healthy because they are more susceptible to infections due to their weakened immune systems and are highly likely to develop severe illnesses or critical conditions. High-risk groups include people with chronic underlying medical problems, such as diabetes, chronic kidney disease, chronic liver disease, chronic lung disease, chronic cardiovascular disease, blood cancer, patients on cancer treatment, patients taking immunosuppressant drugs, and human immunodeficiency virus patients; those in special circumstances that require particular attention (e.g., individuals with morbid obesity, pregnant women, patients undergoing dialysis, organ transplantation recipients, or smokers.); and inpatients who require initial oxygen treatment due to an oxygen saturation (SpO_2_) of below 90% in indoor air.

In Korea, as of April 30, 2020, those aged 70 years or older accounted for more than 60% of severe or critically-ill COVID-19 patients, and almost 1 in 4 people who were 80 years of age or older died from COVID-19, with the fatality rate for this age group standing at 24.33%. A family member, relative, or caregiver who has respiratory symptoms or feels unwell should avoid visiting the elderly or any other people in high-risk groups. It is important for people with chronic conditions to remember to take prescribed medicines as part of their usual daily routines and to follow their medical appointment schedule. Abruptly stopping a prescribed medication may aggravate symptoms. To avoid any possible interruptions in treatment, it is recommended to obtain extra medication after consulting with your physician. Older adults (age 65 years and over) should receive all necessary vaccinations, including pneumococcal vaccines.

#### Stay at home.

Avoid outings to the greatest possible extent, except for grocery shopping and visits to medical institutions or pharmacies. Be careful not to host unnecessary gatherings or to engage in unnecessary travel. Refrain from inviting others to your home or visiting others at their homes. Follow a brief daily home exercise routine to maintain good health. Stay away from alcohol and smoking.

#### If you are sick, contact your local public health center and visit a screening station.

If a high fever (38°C or higher) persists or respiratory symptoms (cough, sore throat, runny nose, etc.) deteriorate, (1) contact the KCDC call center (1339 or area code+120) or a public health center, or (2) visit a screening station for treatment. When visiting a medical institution, make sure to wear a mask. Use your own vehicle if possible. However, people who are unable to remove a mask on their own without assistance or those who have trouble breathing while wearing a face mask are advised not to wear a mask.

#### If you must go outside, observe the following advice.

Avoid going to crowded places, especially enclosed and poorly-ventilated areas. If doing so is unavoidable, you must wear a mask. Maintain a healthy distance of 2 m from other people. In particular, pay particular attention not to have close contact with people who are sick. Avoid physical contact such as handshakes and hugs. Do not share food, drinks, or utensils with other people and use your own cutlery, towels, and other materials.

#### If you are struggling with stress, the following tips can help you.

Limit the amount of time you spend reading or watching news about COVID-19. Seek news updates at specific times during the day. Constant streams of news reports about the pandemic can cause you to feel anxious or distressed. Gather information only from trusted sources. Upon encountering suspicious information, check its source to see whether it is trustworthy. Do not share rumors or misinformation. Take good care of your health. Take time to engage in deep breathing, body stretching, and meditation. Maintain a healthy and balanced diet, regular exercise, and sufficient sleep. Communicate frequently with family members and friends. Talk to people you trust about your concerns and feelings over the phone or video calls.

### Healthy lifestyle

#### Engage in regular exercise.

Regular physical activity plays an important role in preventing and ameliorating various diseases, as well as in promoting health and fitness. It is recommended to limit sedentary leisure time spent sitting, leaning, and lying down (using a computer or smartphone, watching television, etc.) to less than 2 hours a day. Even in this challenging time of the COVID-19 pandemic, physical activity and exercise are crucial elements for maintaining a healthy lifestyle. Learn a daily physical exercise to perform at home, such as watching and following home workout demonstrations. Higher-intensity physical activity can cause increased secretion of saliva droplets, so make sure to ventilate indoors sufficiently after each round of physical exercise. If you have difficulty engaging in regular physical exercise due to old age or illness, seek a type of physical activity that is suitable for your condition and stay active. Avoid excessive physical activity or sudden body movements, which can have negative effects on your body if continued for an extended period of time.

#### Get regular health check-ups.

If you need regular healthcare and medications due to high blood pressure, diabetes, cardiovascular disease, or other chronic conditions, consult your doctor and maintain your treatment. In case of an emergency or another urgent situation, visit a medical institution as needed. Regular healthcare practices, such as timely immunizations and regular check-ups, can help prevent, reduce, or manage diseases.

#### Maintain a balanced diet.

Maintaining good nutrition by practicing a healthy diet in daily life can enhance your immune system to boost resistance to diseases and help you stay healthy. Eat a varied, well-balanced diet prepared using healthy recipes, and drink plenty of water. Do not skip breakfast, and reduce your salt, sugar, and oil intake in your diet.

#### Manage your psychological well-being during times of stress and crisis.

Feeling pressure and distress in the face of an epidemic is a normal experience for everyone. Turn to your family, friends, colleagues, or other trusted persons to communicate and share your experiences, which can help you de-stress and overcome anxiety. Only obtain information from reliable sources. Incorrect information may compound stress and impair proper judgment. Unpleasant emotions like anger and sadness are normal responses that anyone can experience, but if these emotions become excessive, you may need to seek assistance from specialists. For the general public and quarantined individuals, contact the Mental Health and Welfare Center at 1577-0199. Those who have COVID-19, as well as their family members, can contact the National Trauma Center at 02-2204-0001 (or 0002).

## Five key rules for community infection control

### Rule 1: Work together to protect your community.

Action 1: It has been widely recognized that COVID-19 can be transmitted even in its early stages by individuals with mild symptoms, and COVID-19 is characterized by rapid transmission, which raises the risk of community outbreaks. Preventing the community transmission of COVID-19 requires collective efforts on the part of the community/organization, as well as individual efforts.

Action 2: For effective prevention and early containment of the coronavirus, community leaders should establish in advance an efficient and well-organized response system that is based on the consensus and cooperation of community members.

Action 3: The importance of collective efforts to combat the COVID-19 pandemic is relevant for all types of communities, from business communities to non-profit ones such as religious groups and recreational clubs.

### Rule 2: Designate a quarantine manager for effective response.

Action 1: Each community should designate a quarantine manager who is responsible for supervising the community’s infectious disease prevention and control activities.

Action 2: The quarantine manager can be an individual or a team consisting of an appropriate number of people, depending on the size of the community.

Action 3: The quarantine manager serves to protect the community from the spread of COVID-19 and assumes responsibility for carrying out major infectious disease prevention and control activities.

Action 4: Community members should actively heed the requests of the quarantine manager for effective and efficient infection prevention and control.

### Rule 3: Respect your community’s quarantine guidelines.

Action 1: The quarantine manager should conduct a risk assessment that takes into consideration physical density, degree of ventilation, and other various risk factors, and should establish guidelines for quarantine based on the evaluation.

Action 2: The quarantine guidelines should be prepared by referring to relevant sections from the “Complementary guidelines for collective distancing (detailed guidelines).” They must also incorporate the 5 key rules for individual infection control and the 4 complementary actions for individuals..

Action 3: Communities with an environment of mass contact or a high level of enclosure should reinforce rules regarding the practices of frequent handwashing, social distancing of 1–2 m, wearing a mask (or a face-shield), symptom monitoring, and sufficient ventilation.

Action 4: The quarantine manager will hold regular training sessions for the community and assist all members in practicing the quarantine guidelines and taking necessary actions.

Action 5: When a group conducts infection-related activities in the absence of the quarantine manager, another member should support members in carrying out the quarantine guidelines on behalf of the quarantine manager.

Action 6: The quarantine manager should monitor the allocation of roles among community members and the management of facility environments to promote effective implementation of the quarantine guidelines, and should make requests to the community if any room for improvement is identified.

### Rule 4: The quarantine manager will do his or her best to protect the community.

Action 1: The quarantine manager should establish a network of emergency contacts with local public health center personnel and build in advance the cooperative partnerships required for quarantine activities.

Action 2: The quarantine manager should oversee the healthcare of community members, including monitoring their respiratory symptoms and body temperatures on a daily or per-activity basis. When doing so, it is recommended to collect and maintain a daily ledger that records respiratory symptoms for each community member.

Action 3: Community members who have a fever or respiratory symptoms must be immediately sent home and arranged to rest at home for 3–4 days. If the person is a member of a high-risk group or a senior aged 65 years and over, he/she should be guided to a screening station to receive a COVID-19 test.

Action 4: If 2 or more cases of relevant symptoms occur within 3 to 4 days in a specific area of the community where close contact takes place (such as the same division at work or the same class at school), the manager should direct symptomatic persons to undergo COVID-19 testing. If additional suspected cases occur, the manager should report the possible outbreak of a collective infection to the local public health center.

### Rule 5: The leader and members of the community will provide full support for the quarantine manager.

Action 1: The community leader should support the activities of the quarantine manager and cooperate with the manager’s requests (e.g., to reassign responsibilities or improve the environment) for compliance with the quarantine guidelines.

Action 2: The leader and each community member should follow the quarantine guidelines for the safety of the whole community and actively cooperate with the requests of the quarantine manager.

Action 3: The community leader should hold evaluation meetings to assess the community’s quarantine management efforts and discuss any possible improvement on at least a monthly basis.

### Detailed guidelines for physical distancing in daily life

These are presented in Supplement 2. Supplement 3 is the Korean version.

## Conclusion

The above rules and guidelines reflect the best possible current recommendations of the Korean government in the period of COVID-19. Of course, in case of a resurgence of COVID-19, the distancing level may be adjusted (for instance, by enhancing social distancing). It is not difficult to comply with the above recommendations if everybody is considerate of other people. These are the first steps to overcome the danger of being infected with the coronavirus and maintaining daily life on an ongoing basis. It may take a long time to solve the present viral epidemic completely. The new approach to healthy behavior recommended in these guidelines will be essential, not only for Koreans, but also for citizens of countries throughout the world.

## Supplementary materials

Data files are available from Harvard Dataverse: https://doi.org/10.7910/DVN/8JEMAY

Supplement 1. Disinfection guidelines to prevent the spread of COVID-19 at public facilities and multi-purpose facilities” (Edition 3-1)

Supplement 2. Detailed guidelines for physical distancing in daily life for communities and organizations (English translation)

Supplement 3. Rules and guidelines for distancing in daily life to control COVID-19 in Korea, 3rd version announced on July 3, 2020 (original Korean version)

Supplement 4. The audio recording of the abstract.

