# Supplementary material for: Rules and guidelines for distancing in daily life to control coronavirus disease 2019 in Korea: 3rd version, announced on July 3, 2020
Source: J Educ Eval Health Prof. 2020 Jul 13;17:20. doi: 10.3352/jeehp.2020.17.20 (PMC7403533; doi:10.3352/jeehp.2020.17.20)
Supplement: Supplementary file 1 — Supplement 1. Disinfection guidelines to prevent the spread of COVID-19 at public facilities and multi-purpose facilities” (Edition 3-1) [file jeehp-17-20-suppl1.pdf]

Disinfection Guidelines To Prevent the Spread of COVID-19 At Public and Multi-purpose Facilities

(Edition 3-1)

April 2, 2020

This document provides the COVID-19 Disinfection Guidelines of the Republic of Korea. Note that it may be modified to suit particular circumstances of each country and community.

Central Disease Control Headquarters
Central Disaster Management Headquarters

I Overview

| 1 | Introduction

- In the wake of the spread of coronavirus disease 2019 (hereinafter COVID-19), there is a heightened need to provide guidelines on disinfection to prevent and contain infectious disease transmission in local communities.
- This guidance is intended to provide information on prompt and effective disinfection of environments in public facilities and multi-purpose facilities* (hereinafter "facilities") visited by COVID-19 patients, their living spaces, etc.

  * Examples of public facilities: Schools, workplaces, facilities for youths and families, daycare centers, kindergartens, social welfare facilities, postnatal care centers, medical institutions, etc.

  ** Examples of multi-purpose facilities: Libraries, art centers, performance halls, sports facilities, public transportation vehicles (buses, trains, subways, taxis, etc.), shopping malls (large-scale retail stores, markets, duty-free shops, department stores, etc.), movie theaters, large-scale restaurants, public bathhouses, etc.

- Note that this guidance outlines the basic methods and requirements for disinfection in non-medical institutions.

Disinfection in *medical* institutions should be in compliance with *COVID-19 Infection Prevention and Control at Medical Institutions.* (Refer to the KCDC's COVID-19 website for response guidelines for relevant agencies.)

✓ In the event of a confirmed COVID-19 case, it is recommended to entrust cleaning and disinfection work to a professional disinfection service provider.

- This guidance also lays down methods for routine disinfection in local communities (public areas and households) for precautionary purposes to effectively address risks of COVID-19 transmission.

| 2 | Purpose of the Guidelines

- To provide instructions for safe and prompt disinfection in areas used by COVID-19 patients or their living spaces within public or multi-purpose facilities.
- To assist cleaning and disinfecting staff in selecting proper disinfection methods based on the type and state of objects so as to eradicate pathogens thoroughly and effectively.
- To stipulate precautions for cleaning and disinfection staff to protect their health and safety.

This guidance is based on currently available information on the epidemiological characteristics of COVID-19 and is subject to change.

- The legal basis of this guidance is Article 47 of the Infectious Disease Control and Prevention Act and other applicable provisions.

| 3 | Basic Information

- A recent study found that COVID-19 virus (SARS-CoV-2) can survive on surfaces of objects for hours, even days.
  - It is known that COVID-19 can be transmitted through respiratory droplets produced by infected people or by touching with hands the surface of objects and other areas on which these droplets have landed.
- Cleaning and disinfecting the surfaces of objects in spaces where a COVID-19 patient stayed is an effective way to prevent the transmission of SARS-CoV-2 and other viral respiratory diseases.
- Definitions of terms used in this guidance
  - Cleaning refers to a process that removes dirt and impurities (including microorganisms) from surfaces.

    : Cleaning does not inactivate infectious microorganisms, but reduces their number and, thereby, the risk of infection spreading.
  - Disinfecting refers to a process that eliminates microorganisms (except for bacterial spores) from the inanimate environment, generally performed via liquid chemicals or wet pasteurization.

▸ Refer to *The Guidelines on Disinfecting Instruments and Articles Used by Medical Institutions.*

: Killing infectious microorganisms that remain on surfaces after cleaning would further reduce the risk of infection spreading.

II Preparations and Precautions for Disinfection

| 1 | General Principles

- (Disinfection plan) Managers at facilities (hereinafter "managers") must determine the scope of disinfection and establish a disinfection plan after identifying the movement routes of patients*.
  - Formulate specific operating procedures** based on allocation of staff, the scope of disinfection, the selection of disinfectants, and the conditions of each facility (or each zone).

  * Conduct disinfection according to the movement routes of patients, and if impossible to identify the routes, a disinfection plan should be drawn based on identification of objects and zones that are frequently used and touched by people.

  ** If the disinfection is entrusted to a professional disinfection service provider, the provider may conduct the formulation of operating procedures.

- Staff in charge of disinfection work should receive training on operating procedures and infection prevention.
  - The training should include (1) instructions on properly wearing and removing personal protective equipment (PPE), (2) correct ways to wash or disinfect hands, and (3) guidance on monitoring of symptoms after the completion of disinfection work (such as fever, cough, shortness of breath) and actions to be taken following the onset of these symptoms.
  - The staff in charge must wear PPE when cleaning or disinfecting.

  * PPE consists of disposable double gloves (with the rubber outer glove), hygiene masks, protective coveralls (or disposable waterproof long-sleeved gowns or disposable waterproof aprons), goggles (or face shields), and shoe covers (or rubber boots).

- Equipment used for disinfection should be disposable or used exclusively for one purpose, wherever possible.

  * In the case of cleaning equipment that can be reused after washing, disinfect it using an appropriate disinfectant and dry it before storage.

- The criteria for reopening a disinfected facility for use will be determined taking into account the characteristics of each type of disinfectant used, the purposes of the facility, etc.
- Cleaning and disinfection staff must check the hazard information of disinfectants before use.
- Sufficient ventilation should be guaranteed during the entire disinfection process.

| 2 | Preparations before Disinfection

- (Required items) Disinfectants, water, a change of clothes, containers exclusively for medical waste, buckets, disposable cloths (or towels), disposable gloves, hygiene masks, mops, etc.
- (PPE) For routine disinfection, it is necessary to put on disposable gloves and hygiene masks (KF94 or its equivalent) and add disposable waterproof long-sleeved gowns, goggles, boots, etc. depending on the scope of infection and contamination.
- Selection of environmental disinfectants
  - It is recommended to use disinfectants approved by the Ministry of Environment (MOE) for coronavirus and products reported to the MOE as disinfectants* recognized by the World Health Organization (WHO), European Centre for Disease Prevention and Control (ECDC), etc.

  * Sodium hypochlorite (a.k.a. household bleach), alcohol (70% ethanol), quarternary ammonium compounds, peroxygen compounds, etc.

  - It is imperative to comply with the specified usage amount, methods, and precautions for each product.

Select disinfectants suitable for intended use when disinfection involves (1) direct application to the human body (drugs or quasi-drugs), (2) disinfection of food or utensils/containers that can come into contact with food (food additives).

Precautions when using environmental disinfectants

1. Select a disinfectant after checking information thereof, including whether it is approved by or reported to the MOE.

   * Check the official website *Chorok-Nuri* (http://ecolife.me.go.kr) for disinfectant information.

2. When using an environmental disinfectant, make sure to check its expiration date, follow recommendations given by the manufacturer* (such as safe usage and precautions for each product) and prepare a dilute solution according to the manufacturer's instructions.

   * They may include the dilution ratio, contact duration, applicable targets, etc.

3. When using a sodium hypochlorite solution, refer to precautions.

   * Prepare the solution by diluting it just before disinfection. The processing time should last for at least 10 minutes.
   * Do not mix it with ammonia or other disinfectants.

4. The method of spraying the disinfectant should not be applied to the disinfection of surfaces because it increases the risk of generation and inhalation of infectious aerosols, and its disinfecting effect is insufficient due to the unclear scope of contact between the disinfectant and applied surfaces.

   * If the recommended usage method is specified in the instructions of a disinfectant as spraying, spray the disinfectant on a disposable cloth (towel) until it gets wet and wipe surfaces using it.

5. Make sure to check the hazard information of the disinfectant and use it cautiously.
6. Do not mix different types of disinfectants, keep away from flammable materials, and use in well-ventilated areas.
7. As the disinfection effect may decrease over time, dilute only the required amount and use it immediately. Do not store the remaining amount and discard it immediately.
8. Keep the disinfectant out of the reach of infants and children, and pay attention to storage locations and methods.

| 3 | Precautions during Disinfection

- Wear disposable gloves, waterproof long-sleeved gowns, goggles (or face shields), hygiene masks, boots, etc. in compliance with the proper method of wearing PPE.
- Do not touch your eyes, nose or mouth during disinfection while wearing PPE.

  * Wear goggles to keep your hands away from your eyes.

- If gloves or masks become dirty or damaged, take them off safely and put on new ones.

  * The proper order: Remove gloves → Wash hands with soap and water → Remove the mask → Wash hands with soap and water → Wear a new mask → Wear new gloves

| 4 | Precautions after Disinfection

- After the completion of cleaning and disinfection, be careful not to contaminate your body parts and surroundings with pathogens that are present in your PPE when removing the equipment.
- Upon removing each piece of protective equipment, be cautious not to contaminate the surroundings.
- After discarding all disposable PPE used for disinfection in a medical waste container, seal it tightly, follow waste disposal procedures, and wash your hands with soap and water.

  * Reusable goggles can be reused after disinfection according to the manufacturer's guidelines.

- Report to a public health center if any worker who performed cleaning and disinfection subsequently develops a fever or respiratory symptoms within 14 days after disinfection.

III Disinfection of Patient-Use Areas (Zones)

| 1 | Cleaning and Disinfecting

After the disinfection of patient-use areas, the criteria for resuming the use of each facility can be adjusted according to the characteristics of each type of disinfectant used, the purpose of the facility, etc.

- Mark the confirmed contamination area and seal contaminated materials before conducting cleaning and disinfecting of surfaces in a space (zone) used by a patient.

  * This marking and sealing is to prevent further exposal to contamination.

- Leave the windows open for ventilation before, during, and after cleaning and disinfecting.

  * Ventilate for up to 24 hours before cleaning and disinfecting.

- Before cleaning and disinfecting, wear personal protective equipment (PPE) such as hygiene mask (KF94 or its equivalent) and gloves, and during disinfection avoid touching your face (eyes, nose, and mouth).

- Prepare a dilute solution of disinfectant and disinfection equipment.

  - Use MOE-reported and -approved disinfectants such as diluted solutions of sodium hypochlorite, alcohol (70% ethanol).

Illustrations on how to make sodium hypochlorite dilute solutions (5% concentration in the original bleach product)

Final chlorine concentration	Instructions to produce a dilute solution
0.05% (500 ppm)	Pour 5 ml of the product into an empty 500 ml bottle and fill the rest of the bottle with cool water and mix together.
0.1% (1,000 ppm)	Pour 10 ml of the product into an empty 500 ml bottle and fill the rest of the bottle with cool water and mix together.
0.5% (5,000 ppm)	Pour 50 ml of the product into an empty 500 ml bottle and fill the rest of the bottle with cool water and mix together.
1% (10,000 ppm)	Pour 100 ml of the product into an empty 500 ml bottle and fill the rest of the bottle with cool water and mix together.
2% (20,000 ppm)	Pour 200 ml of the product into an empty 500 ml bottle and fill the rest of the bottle with cool water and mix together.

* Calculation of the required amount of the original bleach product: Target amount of dilute solution × final concentration of chlorine ÷ concentration of bleach product (e.g. 500 ml × 0.05 ÷ 5 = 5 ml)

* Duration of contact for a dilute solution of sodium hypochlorite: At least 10 minutes for non-porous surfaces and 30 minutes for the immersion of materials.

- Different facilities require different types of disinfection equipment, and therefore the right type of equipment should be used as the situation requires. Non-disposable equipment must be disinfected properly before reuse.

Cleaning/Disinfecting Procedures (Example)

1. Remove bed linens, disposable curtains, etc.
2. Clean and disinfect the surfaces of all objects and furniture.
3. Clean and disinfect windows and their frames.
4. Clean and disinfect beds and mattresses.
5. Clean and disinfect the floor.
6. Replace the bed linens, curtains, etc. with new or disinfected ones.

- When visibly soiled, surfaces should be cleaned using detergent and water before disinfection.
- (Disinfecting surfaces) Clean the walls within arm's reach and all frequently-touched surfaces* with a cloth (fabric, etc.) wet with the prepared disinfectant, leave it for 10 minutes at least, and then wipe it with a cloth (fabric, etc.) dampened with clean water.

  * High-touch objects and surfaces: Elevator buttons, handrails, door knobs, armrests, backrests, desks, light control devices, keyboards, switches, blinds, and other surfaces with frequent human contact.

  * For surfaces made of metal or other materials which should avoid the use of dilute solutions of sodium hypochlorite, use alcohol (70% ethanol).

  - The method of spraying the disinfectant should not be applied to the disinfection of surfaces because it increases the risk of generation and inhalation of infectious aerosols, and its disinfecting effect is insufficient due to the unclear scope of contact between the disinfectant and applied surfaces.

  * To prevent the formation or splashing of aerosols, it is recommended to apply continuous wiping for cleaning and disinfecting surfaces.

  * The use of compressed air is not allowed due to the risk of aerosolizing infectious substances.

Precautions for cleaning and disinfecting a patient's bodily secretions (vomitus, blood, etc.)

1. Clean the surface first with a disposable paper towel, etc. dampened with disinfectant, and then discard it into a container exclusively for medical waste.

* The presence of foreign materials on a surface diminishes the disinfection effect.

* Patient-use areas, and surfaces or articles contaminated with vomitus, excretion, or secretion: 1,000 ppm dilute solution of sodium hypochlorite.

* Surfaces or articles contaminated by blood spill: 10,000 ppm dilute solution of sodium hypochlorite.

2. Thoroughly clean environmental surfaces using a clean cloth (or a towel) moistened with disinfectant or with a disinfectant wipe. (Caution: do not use compressed air to spray disinfectant.

* Avoid using a vacuum cleaner or other cleaning methods that are likely to aerosolize infectious substances.

3. Clean surfaces with a cloth (fabric, etc.) moistened with the prepared disinfectant, leave it for 10 minutes at least, and then wipe it with a cloth (fabric, etc.) moistened with clean water.

- (Disinfecting floors) Use a mop to clean the floor repeatedly from one end to the other with the prepared disinfectant.
  - Do not move from any area that has not been disinfected to a disinfected area to prevent contamination of the disinfected area.
  - Sweep down the floor repeatedly to avoid generating or splashing aerosols.
- (Bathroom) Use disinfectant to clean all surfaces in the bathroom that can be touched by hand, including the toilet.
- (Cleaning and disinfecting equipment) In principle, mop heads used for floor disinfection, cloths used for surfaces disinfection, etc. must be discarded into containers exclusively for medical waste.
  - Non-porous equipment used for one space must be disinfected before being used for another space.
  - Articles that are difficult to dispose must be disinfected by being immersed into a dilute solution of disinfectant for 30 minutes.

- Buckets should be immersed into a dilute solution of disinfectant (or 1,000 ppm sodium hypochlorite dilute solution) for 10 minutes or longer, or rinsed with hot water to disinfect.
- Waste materials produced during disinfection must be discarded into containers exclusively for medical waste.

* After disinfecting public and multi-purpose facilities to prevent infection, the ensuing waste must be double-sealed, disinfected and burned in a public incinerator or similar facility.

- Shower and change clothes immediately after cleaning and disinfecting.
- (Follow-up management for staff) If a worker who engaged in cleaning and disinfection subsequently develops a fever or respiratory symptoms, instruct the person to stay in a designated area within the facility and contact a public health center or the KCDC call center (1339 or area code+120).

Disinfection methods for a patient's living space

1. Household goods (dishware, drinking glasses, cups, eating utensils, bedding, etc.) must be used separately for each individual and not be shared. After use, clean them thoroughly with detergent and hot water.
2. For further information on specific disinfection procedures, refer to *III. Disinfection of Patient-use Areas (Zones) and Appendix 2. Disinfection Methods in a Patient's Home Setting.*

| 2 | Laundry

- When handling patients' laundry, wear a hygiene mask and disposable gloves, both of which must be discarded after each use. Reusable gloves must not be reused for other household chores.
- Do not shake patients' laundry.
- Clean bed linens, pillowcases, blankets, curtains and other washable fabric items in a washing machine using warm water and detergent (or disinfectant).
  - When putting laundry in the washing machine, wear the appropriate PPE, including a hygiene mask, gloves, and an apron.
  - For warm-water washing, use 70°C water and general detergent for 25 minutes at least.
  - For lower-temperature washing (less than 70°C), use the detergent or disinfectant appropriate for lower temperature washing.

* Products containing sodium hypochlorite or peroxide (a laundry sanitizer for fabric) may release hazardous gases and cause fabric damage and other problems during high-temperature washing, so use them at a temperature of 60°C or below according to the manufacturer's instructions.

- Dry laundry completely after washing

* If there is a dryer machine, it is recommended to dry at 80°C for two hours.

- For items that are difficult to wash such as a mattress or a carpet, entrust them to a professional disinfection service provider for proper disinfection, or carry out steam (high temperature) sterilization.
- Use disposable or single-use laundry hampers or similar cart. Otherwise, non-disposable carts should be disinfected before reuse.
- Disinfect the surfaces of clothes hangers after use.
- When laundry is soiled with blood or body fluids to an extent that cannot be cleaned properly, discard them.

Patients' clothes and other linen products from medical institutions should be handled in compliance with instructions outlined in *COVID-19 Infection Prevention and Control at Medical Institutions.*

- After laundry, remove disposable gloves, etc., and wash hands thoroughly with soap and water.
- If a cleaning and disinfecting worker develops a fever or respiratory symptoms within 14 days after laundry, instruct the person to stay in a designated area and contact a public health center or the KCDC call center (1339 or area code +120).

IV Routine Disinfection Methods for Communities (Public Places and Houses)

This section outlines routine disinfection methods for communities in the event of a COVID-19 outbreak.

| 1 | Maintaining Cleanliness at Public Places in Communities

- Persons who carry out cleaning and disinfection work should wear the appropriate personal protective equipment (PPE).

  * Wear disposable gloves and hygiene masks at all times and, if necessary, disposable long-sleeved waterproof gowns (or waterproof aprons), goggles (or face shields), etc.

- Surfaces of high-touch objects should be disinfected.

Examples of key areas for disinfection

1. Surfaces of high-touch objects (e.g. handles, handrails, doorknobs, armrests, plug sockets, switches, etc.)
2. High-touch surfaces in offices (e.g. keyboards, desks, chairs, phones, etc.)
3. Bathroom sink faucets, bathroom door handles, toilet lids, bathtubs, and other bathroom surfaces

- (Method) Wipe surfaces with cloth sufficiently wet with disinfectant, such as alcohol (70% ethanol), diluted sodium hypochlorite (500-1,000 ppm).

* For making 1,000 ml of a 500 ppm sodium hypochlorite dilute solution: Mix 10 ml of bleach (5%) in 1,000 ml of cold water.

* When using a sodium hypochlorite dilute solution, the concentration of 500 ppm is recommended for general surfaces and 1,000 ppm for bathrooms.

* Further consideration must be given to characteristics of each type of disinfectant, the usage of facility, etc.

- (Frequency) Disinfection of high-touch objects should be performed at least once a day.

* Caution should be exercised, as the excessive use of disinfectant may be hazardous to the human body.

* As sodium hypochlorite can cause skin damage, eczema, headaches triggered by unpleasant odor, etc., it is recommended to keep windows open and use a ventilator when this substance is used.

- Entrance doors, elevators, etc. of each building should be cleaned and disinfected on a more frequent basis.
  - For areas frequently used during commuting hours, etc., the employee in charge of cleaning and disinfection should wipe door handles and elevator buttons with cloth sufficiently saturated with disinfectant.

The method of spraying the disinfectant should not be applied to the disinfection of surfaces, because it increases the risk of generation and inhalation of infectious aerosols, and its disinfecting effect is insufficient due to the unclear scope of contact between the disinfectant and applied surfaces.

| 2 | Basic Principles

- Facility managers should provide a sufficient amount of cleaning and disinfecting products and PPE (disinfectant, paper towels, masks, etc.) to the employee in charge of cleaning and disinfection.
- If a worker who carried out cleaning and disinfection subsequently develops a fever or respiratory symptoms, instruct the person to stay in a designated area within the facility and contact a public health center or the KCDC call center (1339 or area code+120).

Appendix 1

Guidance on Criteria for Disinfection Timing and Reopening for Use after Disinfection

The criteria for reopening for use can be adjusted according to the characteristics of each type of disinfectant used and the purpose of the disinfected area.

- The virus will be destroyed after disinfection, but the precautions for each disinfectant should be taken into account in deciding the timing for reopening for use as it cannot be applied across the board due to the different characteristics of each product.
- In the case of disinfection using a sodium hypochlorite dilute solution (1,000 ppm or more), the area can be used after adequate ventilation. (It is recommended to restrict its use until the day after disinfection and to start its use only after adequate ventilation.)

Classification	Disinfection Timing	Criteria for Reopening for Use	Note
**Areas (zones) used by patients at public facilities and multi-purpose facilities**	When it is confirmed that a patient has used the area (zone) within the facility.	Adjustable in consideration of the characteristics of each type of disinfectant used and the purpose of the disinfected area	In the case of sodium chlorite, which requires the use of a highly-concentrated dilute solution, it is desirable to reuse the area after adequate ventilation for a day after disinfection, given the possibility of hazards, such as odor.
**Medical institutions (hospitals)**	Immediately disinfect the surfaces in environment contaminated with blood, body fluids, secretion or excrement.<br><br>Disinfect environment surrounding the isolation room where a patient is staying at least on a daily basis and high-touch surfaces more often.<br><br>Disinfect after a patient is discharged.	It is recommended to resume medical treatment after ventilating for at least 2 hours with a minimum air change rate of 6 per hour.	*Guidance on Infection Prevention and Control of Novel Coronavirus Disease (for hospital-level medical institutions)* (March 16, 2020)

**Medical institutions (clinics)**	Environmental management after a visit by a suspected COVID-19 case.	It is recommended to resume medical treatment after ventilating for at least 2 hours with a minimum air change rate of 6 per hour.	*Guidance on Infection Prevention and Control of Novel Coronavirus Disease (for clinic-level medical institutions)* (February 11, 2020)
**Medical institutions (emergency rooms)**	When a suspected COVID-19 case visits an emergency room.	It is recommended to resume medical treatment after ventilating for 4 hours or more under 6-12 air changes per hour.	*COVID-19 Working Guidance for Medical Institutions* (February 20, 2020)

Appendix 2

Disinfection Methods in a Patient's Home Setting

1. Before starting disinfection, wear a hygiene mask and gloves, and avoid touching your face (eyes, nose, mouth) during disinfection and cleaning.

* Wear personal protective equipment (PPE) such as a mask, goggles, a face shield and a disposable waterproof apron as the situation may require.

2. Prepare a diluted solution of disinfectant.

* Prepare a 1,000 ppm dilute solution of sodium hypochlorite (e.g. household bleach solutions), etc. and comply with the manufacturer's warnings and instructions.

3. Leave windows open to ventilate.
4. Disinfect the floor of the disinfection area repeatedly from one end to the other with the prepared disinfectant.
5. Clean all areas* used frequently and the surfaces of the bathroom with cloths (or towels) moistened with the prepared disinfectant.

* Handles, armrests, desks, chairs, keyboards and mice, switches, blinds, windows, walls, etc.

6. Wash bed linens, pillowcases, and blankets in a washing machine with hot water and detergent.

* As a laundry sanitizer for fabric could cause various problems like release of toxic gases or fabric damage, make sure to use it at 60°C or below under the manufacturer's instructions.

7. Do not use the mattresses, pillows, carpets, cushions, etc. that were used by a suspected COVID-19 patient until the test result is released.

* If the result shows positive, entrust the items that are difficult to wash, such as mattresses and carpets, to a professional disinfection service provider to disinfect properly, or carry out steam (high temperature) sterilization.

8. Put all cloths (towels) used for disinfection and other waste produced during disinfection into a designated bag.
9. Take off the disposable gown and wash hands with water and soap. → Remove gloves and wash hands with soap and water. → Remove goggles and wash hands with soap and water. → Remove the hygiene mask and wash hands with soap and water.
10. Each time you take off the gown, the gloves and the mask, put each of them into a designated bag.

11. All waste produced by disinfection must be disposed separately from other household waste.
12. Shower and change the clothes immediately after cleaning.
13. Ventilate disinfected spaces.
14. If the person who carried out disinfection develops a fever or respiratory symptoms within 14 days after disinfection, he or she must stay in a designated area and contact a public health center or the KCDC call center (1339 or area code +120).

In the event of a confirmed COVID-19 case, it is recommended to outsource disinfection to a professional disinfection service provider.

Appendix 3

Routine Disinfection (Example)

1. Make the dilute solution of disinfectant using a PET bottle in a well-ventilated place.

**Preparation**	• sodium hypochlorite (a.k.a. household bleach, 500-1,000* ppm)<br>• a 500 ml PET bottle (well-cleaned and dried), a measuring cup, paper towels<br>• a standard waste disposal bag (Putting a new bag in a waste bin in advance is recommended for easy disposal.)<br>• disposable gloves, rubber gloves, a mask, a waterproof apron, etc.<br>* Recommendation: 500 ppm for general surfaces, 1,000 ppm for bathrooms	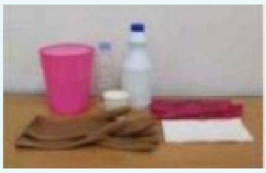

How to make a 0.05% (500 ppm) dilute solution of sodium hypochlorite, using 5% sodium hypochlorite:

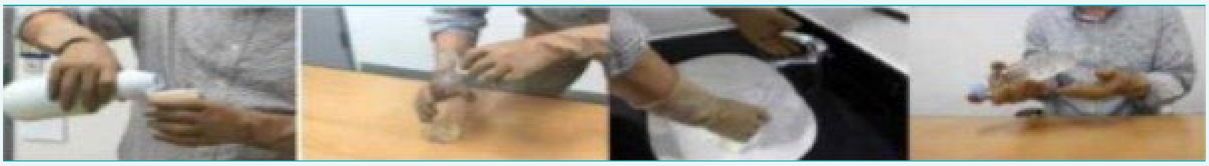

(1) Pour 10 ml of 5% solution into a PET bottle. (2) Add cold water up to 500ml, close the bottle and mix it well.

2. Disinfect high-touch places and bathroom surfaces while ventilating.

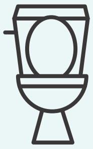	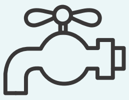	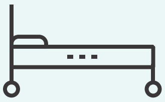	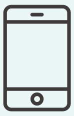	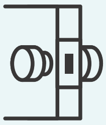	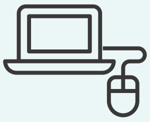
Toilet	Faucet	Bedrail	Phone	Doorknob, window frame, etc.	Computer and mouse

Source: www.cloroprofessional.com

* Metal surfaces are disinfected by alcohol (70% ethanol).

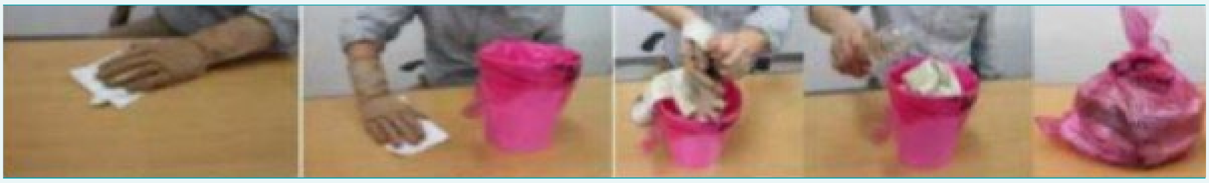

(1) Clean with a paper towel moistened with disinfectant and leave it for 10 minutes at least and wipe with a water-saturated paper towel. (2) After use, put disposable tools into a waste disposal bag, spray disinfectant into it, seal the bag and discard it.

3. Ventilate sufficiently.

4. After cleaning and disinfecting, wash hands with soap and water.

Precautions

- Disinfect high-touch surfaces like a door handle and a bathroom at least once daily in home setting and public places such as multi-purpose facilities. Note that higher-touch surfaces should be disinfected more frequently.
- If a surface is soiled with vomitus or other body fluids, clean with absorbent disposable towels before disinfection.

Appendix 4

Precautions for Using Sodium Hypochlorite

Effects

- Sodium hypochlorite (the main active ingredient in household bleach) is a strong and effective disinfectant that denatures protein in microorganisms, thereby killing bacteria, fungi and viruses.
- Sodium hypochlorite is widely used as a disinfectant due to its rapid effectiveness and low price.
  - Therefore, diluted sodium hypochlorite is recommended for use to disinfect the environment.
- (Disadvantages) Caution should be exercised in the use of sodium hypochlorite as it irritates mucous membranes, skin and respiratory tract and decomposes under heat and light, thereby causing it to easily react with other chemicals.
  - If not properly used, this substance may result in a reduced disinfection effect and an increased risk of injury.
  - The excessive use of sodium hypochlorite would pose environmental pollution risks.

Instruments and equipment

- Prepare all necessary instruments and equipment, such as sodium hypochlorite for household use, measuring tools, containers and personal protective equipment (PPE).

Preparation of dilute solutions

- Dilute and use sodium hypochlorite in well-ventilated areas.
- As this substance causes irritation to mucous membranes, skin and respiratory tract, wear appropriate PPE (e.g. masks, gloves, safety goggles, waterproof gowns, etc.) when manufacturing and using sodium hypochlorite dilute solutions.
- Use cold water when diluting, as hot water decomposes the active ingredient of sodium hypochlorite, thereby negating the disinfection effect.
- Properly dilute the sodium hypochlorite depending on the specific condition before using it.

  * Cleaning and disinfecting for general household use: 500 ppm

  * (1) Patient-use areas, and (2) surfaces or articles contaminated with vomitus, excretion, and secretion: 1,000 ppm

  * Surfaces or articles contaminated by blood spill: 10,000 ppm

Effective chlorine concentration (ppm)	Final concentration of sodium hypochlorite (%)	Mixture ratio of sodium hypochlorite and water	
		4% (40,000 ppm) bleach	5% (50,000 ppm) bleach
500 ppm	0.05%	1:80	1:100
1,000 ppm	0.1%	1:40	1:50
5,000 ppm	0.5%	1:8	1:10
10,000 ppm	1.0%	1:4	1:5

- Use a measuring spoon or measuring cup to accurately gauge the amount of sodium hypochlorite added.
- Thoroughly wash hands using soap and water after disinfection.

Precautions when using sodium hypochlorite

- Do not use on metals, wool, nylon, silk, dyed fabric and painted surfaces.
- Do not touch your eyes with hands smeared with a dilute solution.
  - If sodium hypochlorite enters the eyes, immediately rinse with water for at least 15 minutes and consult a physician.
- Do not mix sodium hypochlorite with other household detergents as their combined use may reduce the disinfection effect and cause dangerous chemical reactions.

  * (Example) When sodium hypochlorite is mixed with acidic detergents such as those used for toilet cleaning, this can produce a toxic gas and thereby cause accidents or injuries.

  ** If necessary, use detergents first and rinse thoroughly with water before using sodium hypochlorite as a disinfectant.
- As undiluted sodium hypochlorite emits a toxic gas when exposed to sunlight, it should be stored in a cool, shaded place, out of the reach of children.
- As sodium hypochlorite decomposes over time, purchase recently-produced sodium hypochlorite to ensure its effect.

  * When sodium hypochlorite is left unused, its decomposition increases over time. To ensure effective disinfection, use diluted sodium hypochlorite within 24 hours after diluting and discard unused portions after disinfection.
- As the presence of organic materials on a surface diminishes the effect of sodium hypochlorite, such materials should be completely removed before disinfecting.
