# Supplementary material for: Rules and guidelines for distancing in daily life to control coronavirus disease 2019 in Korea: 3rd version, announced on July 3, 2020
Source: J Educ Eval Health Prof. 2020 Jul 13;17:20. doi: 10.3352/jeehp.2020.17.20 (PMC7403533; doi:10.3352/jeehp.2020.17.20)
Supplement: Supplementary file 2 — Supplement 2. Detailed guidelines for physical distancing in daily life for communities and organizations (English translation) [file jeehp-17-20-suppl2.docx]

**Supplement 2.** Detailed guidelines for physical distancing in daily life for communities and organizations, May 3, 2020

Central Disaster and Safety Countermeasure Headquarters, Ministry of Health and Welfare, Seoul, Korea

Editor’s note: It is the translation of the first version of the guideline. Translation for the 3rd version will be added soon.

**[Contents]**

**I. At work (4 fields)**

1. When working

1-1. Workplace

1-2. Meeting

1-3. Public service counter

1-4. Post office

**II. Daily life (9 fields)**

1. When moving

1-1. Public transportation

2. Meals

2-1. Restaurant cafe/study cafe

3. Studying

3-1. School, reading room, etc.

4. Shopping

4-1. Large distribution facilities (department stores, large marts, etc.)

4-2. Traditional market

4-3. Small supermarket

5. Special event

5-1. Family events such as weddings

5-2. Funeral hall

6. Religious life

6-1. Religious facilities

**III. Leisure**

1. Traveling

1-1. Hotel, condominium

1-2. Amusement facilities

1-3. Campsite

1-4. Zoo

1-5. National park

2. Leisure, etc.

2-1. Outdoor activities

2-2. Public toilets

2-3. Barbershop and beauty salon

2-4. Bathhouse

2-5. Library

2-6. Theater

2-7. Movie theater

2-8. Museums and art museums

2-9. Baseball field, soccer field

2-10. Karaoke (singing room)

2-11. Indoor sports facilities

2-12. PC room

2-13. Amusement (nightlife) center

**I. At work: 1. When working**

**1-1. Distancing in daily life: workplace**

**<Laborer>**

**[Common information]**

● Do not go to work if you have a fever or respiratory symptoms (cough, sore throat, etc.) or have traveled overseas within the last 14 days.

● Keep at least 2 m (1 m) away from others

● Wash your hands for more than 30 seconds with running water and soap; otherwise, disinfect your hands with hand sanitizer

● When you cough or sneeze, cover your mouth and nose with tissue paper or sleeves

● Refrain from spitting water droplets (singing songs, shouting relief, etc.) or from physical contact (handshake, hugging, etc.)

● Wear a mask when using indoor multi-use facilities

● Wear a mask if you cannot keep a distance of 2 m from others outside

**[Specific information]**

● If fever or cough occurs during work, inform the employer and wear a mask to leave the office.

● Active use of flexible working hours (home-work, timed commuting, etc.), vacation system (family care leave, annual leave, sick leave, etc.)

● Domestic and foreign business trips should be reduced as much as possible.

● Workshop, education, training, etc. should be opened through online or video meetings whenever possible, and if a face-to-face meeting is required, keep personal hygiene practices such as wearing a mask and using disinfectants.

● Use personal items such as personal teacups and teaspoons

● Periodically disinfect the places where hands are frequently touched (table, keyboard, mouse, and phone, etc.)

● Ventilation offices, working place, etc.

● Avoid small group meetings, club activities, dinners, etc., and return home early after work. Sit in a row or zigzag and avoid conversation in the cafeteria. Wear a mask and refrain from talking in confined spaces such as elevators. Do not stay at the resting room with others.

● Allow the check of body temperature daily with a non-contact thermometer or thermal imaging camera and the confirmation of the respiratory symptoms.

**<Business owner>**

**[Common information] [B-Common principles for officers, employees]**

● Establish a quarantine cooperation system, such as designating a department (administrator) in charge of quarantine and securing a communication network for local health center officers.

● If 2 or more symptomatic suspected cases were found within 3–4 days in the same department, same class, or same place where close contact is made in the community, the symptomatic case will be directed to the SARS-CoV-2 test. Report the possibility of a mass infection if symptomatic cases were found additionally.

● If a worker has a fever or respiratory symptoms, exclude him or her from working and immediately ask him or her to return home.

● Keep the distance between people more than 2 m (at least 1 m).

● Provide facilities for hand washing or hand sanitizers, and post notices for hand washing and cough etiquette.

● If natural ventilation is possible, keep windows open at all times, and if it is difficult to keep windows open due to air conditioning, etc. ventilate at least twice daily, taking into account area and personnel.

● Commonly used objects (such as door handles) and their surfaces are disinfected more than once every day.

● Wear a mask when dealing directly with customers (users).

● If anyone has a fever or respiratory symptoms or has traveled overseas within the last 14 days, refrain him or her from visiting any places.

● Guide persons to wear a mask when using indoor multi-use facilities.

● Guide persons to wear a mask if they cannot maintain a distance of 2 m outside.

**[Specific information]**

● The quarantine manager makes a quarantine guideline in consideration of worker density, ventilation conditions, and work style.

● If laborers have a fever or respiratory symptoms, or have traveled abroad within the past 14 days, ask them to use telecommuting, sick leave, annual leave, and temporary leave.

● Check the body temperature of laborers daily with a non-contact thermometer or thermal imaging camera and confirm the presence of respiratory symptoms.

● Create an atmosphere where laborers can freely use the flexible working system and vacation.

● Domestic and foreign business trips should be reduced as much as possible.

● Use online or video for week shops, education, training, etc., but measure body temperature, wear a mask and provide disinfecting products when face-to-face.

● Workshop, education, training, etc. should be opened through online or video meeting whenever possible, and if a face-to-face meeting is required, prepare the body temperature tester, check the mask-wearing, and put the disinfectants in front of the meeting room.

● Keep the distance of 2 m (at least 1 m) between workers by adjusting the position and orientation of the monitor, desk, and workbench or by using an unoccupied space.

● Do not induce spitting drops (group relief, shouting aid, etc.).

● Install transparent bulkheads between the cafeteria seats or make them sit in a row or zigzag whenever possible.

● Provide or furnish personal cleaning and disinfecting products.

● Provide and supply masks and hygiene products according to the situation of the workplace or support laborers to purchase them.

● Post or train laborers for hygiene management measures such as hand washing, use of hand sanitizer, and cough etiquette.

● Guide not to use the resting room simultaneously by many laborers.

● Prepare the temporary meeting rooms, etc. that can be used for the clients according to the situation of the workplace.

**1-2. Distancing in daily life: meeting**

**[General principles]**

● If possible, use video conference, telephone conference, etc.

● Improve the working environment to enable video conferencing and telephone conferencing

● Secure large spaces for easy ventilation and for distancing in face-to-face meetings.

● Shorten meeting time by minimizing attendance and by efficient moderation.

**[When face-to-face meetings are opened, observe the following:]**

● If anyone has a fever, respiratory symptoms (throat pain, cough, dyspnea, boredom, headache, muscle pain, etc.) in advance, or if anyone has traveled abroad within 14 days, notify him or her not to participate in the meeting.

● The host or moderator checks fever, respiratory symptoms, etc. before the meeting, and the symptomatic person should abstain from attending the meeting.

● Avoid physical contact such as shaking hands before and after the meeting.

● Hand disinfectants are placed throughout the conference room so that attendees can use them frequently.

● Use a large space with easy ventilation for the meeting place and ventilate before the meeting.

● After an hour, take a break and open the doors and windows of the meeting place for ventilation.

● Keep a distance of 2 m between attendees of the meeting and at least 1 m even if the space is small.

● Avoid face-to-face meetings if the distance cannot be maintained over 2 m (at least 1 m) and ventilation is not possible every 1 hour.

● A face-to-face meeting is opened inevitably; all participants should wear a mask and keep wearing the mask even when speaking.

*The mask-wearing is optional by individual’s choice when maintaining enough distance and ventilation.

**1-3. Distancing in daily-life: public service counter**

**<User>**

**[Common information] [A-Common principles for user or visitor]**

● Do not visit the public service counter if you have a fever or respiratory symptoms (cough, sore throat, etc.) or have traveled overseas within the last 14 days.

● Keep at least 2 m (at least 1 m) away from others.

● Wash your hands for more than 30 seconds with running water and soap; otherwise, disinfect your hands with hand sanitizer.

● When you cough or sneeze, cover your mouth and nose with tissue paper or sleeves.

● Refrain from spitting water droplets (singing songs, shouting relief, etc.) or from physical contact (handshake, hugging, etc.).

● Wear a mask when using indoor multi-use facilities.

● Wear a mask if you can’t keep a distance of 2 m from others outside.

**<Officer and employee>**

**[Common information] [B-Common principles for officers, employees]**

**[Specific information]**

**<Worker>**

● If a worker has a suspected symptom such as fever and cough or has traveled overseas within the last 14 days, the department head can take measures to prevent the employee from going to work.

*The public officials go to work if there is no problem after watching the progress of symptoms for 3–4 days.

● Check the body temperature, etc., twice or more while working → Those with abnormal symptoms should follow the measures after consulting with the health authorities.

● If there is a confirmed patient, prepare a reporting system to identify unusual signs, and prepare a thorough record of the moving line.

● Employees should be able to use a flexible work schedule and vacation as freely as possible, and secure alternative workforce.

**<Public service counter>**

(1) Appoint the head of the civil affairs department as the person in charge of quarantine management so that he or she able to disinfect the working place as follows:

● Place disinfectant items such as hand sanitizers at the public service reception desk, unmanned civil service document issue machines, public spaces, toilets, etc.

● Disinfect civil service rooms and public areas more than twice a week, and clean the floor with disinfectants.

*For the disinfectant and disinfecting method, refer to “CVD-19 Response Group Disinfection and Multi-Use Facility Disinfection Guide.”

● Prepare and implement a quarantine countermeasure appropriate to the actual situation: to install a transparent screen for public service counter; to provide a disposable mask for visitors who do not wear a mask, and to close the doors except the only one central entrance.

● Check fever with the thermometer, or a thermal imaging camera, and prepare a waiting room for the visitors with fever (preparation and management of record book).

● Implement countermeasures appropriate to your situation, such as installing a transparent screen at the civil service counter, opening an automatic door always, or opening only one entrance after closing all other entrances.

(2) Maintaining the cooperation system of related organizations

● Maintaining emergency contact systems of related organizations (health centers, police stations, fire stations, medical institutions, etc.) and responding immediately in the event of an occurrence of COVID-19 cases.

● If there is a suspected case in the public service room, immediately report to the relevant health center and keep them at the isolation room.

● If there is a confirmed case in the public service room, immediately notify it to the competent health center, and take necessary preventive measures such as temporary closure of facilities, no access, isolation, and disinfection.

*For detailed information, refer to "Recommended measures for prevention and management in the event of COVID-19 confirmed cases at the facilities" (Central Disease Control Headquarters-125, 3.6.)

(3) The issuing of civil service documents should be minimized as much as possible by activating the use of “Government 24, unmanned civil service document issue machine, electronic certificate.”

**[Education and promotion of hygiene rules for public service workers and visitors]**

● Provide training on the prevention of infectious diseases such as the prevention of novel coronavirus infection, hand washing, and cough etiquette for employees at the public service counter

● Post various promotional materials* to major places in the facility, such as hygiene rules to prevent novel coronavirus infections such as hand washing and cough etiquette

* For related promotional materials, use the data posted on the website of the Korea Centers for Disease Control and Prevention (www.kcdc.go.kr)

*For matters not mentioned in terms of prevention and disinfection, refer to “Coronavirus Infectious Diseases-19 Guidelines for Collective Facilities and Multi-Use Facilities (3rd edition)” (Central Disease Control Headquarters, Central Disaster Management Headquarters, 3.25), “Coronavirus Infections-19 Response to Disinfection of Collective Facilities and Multi-Use Facilities (3rd edition)” (Central Disease Control Headquarters, Central Disaster Management Headquarter, 4.2.)

**1-4. Guidelines for distance in life: post office**

**<User/visitor>**

**[Common information] [A-Common principles for user or visitor]**

**[Specific information]**

● Use smart banking or internet banking if possible. When anyone visits the post office, use an automated teller machine (ATM).

● When using a postal service, use an unmanned postal service machine if that machine is provided.

**<Officer and employee>**

**[Common information] [B-Common principles for Officers, employees]**

**[Specific information]**

● Maintaining a gap with other people during mealtime (sitting in one direction, alternative sitting, sitting in a zigzag, etc.).

● Refrain from visiting different departments and moving between floors, avoid travel if not essential, avoid face-to-face conversations, and activate communication through telephone, messenger, and enable the non-face-to-face work-flow.

● Minimize large-scale events or joint activities and activate online training or meetings.

● Keep toilet paper and litter bins at entrances and facilities.

● Announce and request periodically for cooperation regarding the necessity of keeping personal hygiene rules and keeping distance in life.

● Announce the guideline to use smart banking, internet banking, ATM devices, and unmanned postal service.

**II. Daily life: 1. When moving**

**1-1. Distancing in daily life: public transportation**

**<Users>**

**[Common information]**

● Do not use public transportation if you have a fever or respiratory symptoms (cough, sore throat, etc.) or have traveled overseas within the last 14 days.

● Keep at least 2 m (at least 1 m) away from others.

● Wash your hands for more than 30 seconds with running water and soap; otherwise, disinfect your hands with hand sanitizer.

● When you cough or sneeze, cover your mouth and nose with tissue paper or sleeves.

● Refrain from spitting water droplets (singing songs, shouting relief, etc.) or from physical contact (handshake, hugging, etc.).

● Wear a mask when using indoor multi-use facilities.

● Wear a mask if you cannot keep a distance of 2 m from others outside.

**[Specific information]**

● Wear a mask and avoid conversation in a bus, subway, or taxi.

● Stay away from others in public transportation.

● When you book seats for public transport such as trains and highway buses, reserve the seat after confirming empty one near seat (priority for window-side seats, etc.).

● If the bus or subway is crowded, use the next one if possible.

● When taking a taxi, pay with an app payment method or the non-face-to-face payment method.

**<Officers and employees>**

**[Common information] [B-Common principles for Officers, employees]**

**[Specific information]**

● Guide users to wear a mask in public transportation, and to keep the physical distance from others as much as possible.

● Ventilate the transport vehicles frequently before and after the operation.

● To alleviate the crowdedness of public transportation, adjust the allocation interval flexibly by checking the crowdedness from time to time.

● When users make reservations for railways, airlines, high-speed buses, intercity buses, etc. allocate the seats with physical distance.

● Introduce the non-face-to-face automatic payment method when users reserve the seats for public transport or pay the call taxi.

● Switch cargo transportation such as courier service to non-face-to-face delivery whenever possible.

● Promote user compliance precautions through billboards and announcements.

**II. Daily life: 2. Meals**

**2-1. Guidelines for keeping physical distance in daily life: restaurants, cafes, study cafes**

**<Users>**

**[Common information] [A-Common principles for user or visitor]**

**[Specific information]**

● Minimizing time spent in restaurants and cafes.

● Sit at a distance of 2 m (at least 1 m) between tables or sit as far apart as possible from others.

● Sit so that you can face one direction without facing each other.

● Avoid conversation when eating.

● Take each food on one’s own plate.

● Use packaging and delivery service if possible.

● Do not recommend drinking alcohol.

**<Officers and employees>**

**[B-Common principles for Officers, employees]**

**[Specific information]**

● Avoid facing customers as much as possible by installing non-face-to-face devices or transparent partitions, etc.

● Prepare a method to keep the distance between tables, such as placing a space between tables 2 m apart (at least 1 m) or installing partitions between tables, and prohibiting the use of some fixed tables.

● Try not to face each other, such as placing the chairs in one direction or zigzag.

● Avoid holding large events.

● Facilitate packaging and delivery sales if possible.

● Conduct regular employee training on compliance with personal hygiene rules and the necessity of keeping distance in daily life.

● When waiting occurs, use a number tag or guide people to wait at a distance of 1 m between the waiting people.

● Prepare the facilities for clients to eat individually by providing dishes, spoon, tongs, etc.

**II. Daily life: 3. Studying**

**3-1. Guidelines for keeping distance in your life: private educational institute, reading room, etc.**

**<User>**

**[Common information] [A-Common principles for user or visitor]**

**[Specific information]**

● Visit this place after consideration of avoiding crowding of public facilities, such as indoor lounges, cafes, and canteens.

● Ask each other for compliance with the prevention guidelines when anyone without wearing a mask was met or febrile persons are found.

*High-risk group including senior citizen, pregnant women, chronic patients, etc. is recommended not to visit public facilities. If they should visit them, they should wear a mask.

● Cooperate in check of symptoms, such as fever, respiratory symptoms, etc.).

*When visiting restaurants, cafes, etc. in the facility, corresponding guidelines are applied.

**<Officer and employee>**

**[Common information] [B-Common principles for officers, employees]**

**[Specific information]**

● In the classroom and reading room, place seats at a distance of 2 m (minimum 1 m) or more, or zigzag so that the next and front seats are empty; otherwise, install partition walls such as transparent partitions.

● Instructors and lecturers must always wear masks and ask users to wear masks.

● Keep facing tissues and litter bins at entrances and several points in the facilities.

*The tissues used for coughing or sneezing must be discarded.

● Daily disinfect facilities, including door handles, railings, etc., especially in places with frequent passage and objects that are frequently touched by people at least 2 times/day. In large institutes, more than 1,000 m^2^, disinfectant least once a week, ventilate frequently, and prepare the ledger, including date and manager’s confirmation.

● Providing group meals is prohibited. However, if institutes operate boarding and whole-day class, the guidelines for restaurants and cafes may be applied accordingly.

● When using laptops, notebook, or tablet PCs, employees should use personal devices whenever possible.

● Periodically train and guide on compliance with personal hygiene rules and the necessity of keeping physical distance in daily life.

● Prevent users from being crowded by limiting the number of users and pace by time zone.

● High-risk groups, including senior citizens, pregnant women, chronic patients, etc. are advised to refrain from using the facility. If their visit is unavoidable, they should wear mask.

● Check the symptoms of people who enter and leave, including fever, respiratory symptoms, etc.

● Keep a list of visitors or users as much as possible and ask them to fill it out.

*If there are restaurants and cafés in the facility, those guidelines are applied.

**II. Daily life: 4. Shopping**

**4-1. Guidelines for distance in daily life: large distribution facilities**

*Department stores, large marts, complex shopping malls, corporate supermarkets, outlets, etc.

**<User>**

**[Common information] [A-Common principles for user or visitor]**

**[Specific information]**

● Keep a distance of more than 2 m (at least 1 m) from other visitors while picking goods up or standing on the checkout line.

● Shop with a minimum number of people.

● Disinfect surfaces before using a shared shopping cart or shopping basket; otherwise use a hand sanitizer.

● Avoid using cosmetic samples directly on the face or lips. Instead, test on the back of the hand. After testing, disinfect, or wash hands.

● Use electronic payment methods (mobile payment, QR code, NFC card, credit card, etc.).

*When visiting restaurants, cafes, etc. in the facility, corresponding guidelines are applied.

**<Officer and employee>**

**[Common information] [B-Common principles for officers, employees]**

**[Specific information]**

● Make employees’ working hours flexible and vacations as free as possible, and secure alternative workers to the extent possible.

● Refrain from events where many users can be crowded in one place at a time (e.g., on a first-come, first-served basis, handshake, autograph session, etc.). When inevitably conducting guest events, a plan to disperse the users should be prepared.

● Avoid acts that may cause splashings, such as acting loudly and replace it with announcements and leaflets.

● Stop or minimize the operation of the tasting and cosmetics test corner. Take measures to dispose of trash, such as toothpicks, cups, tissues, cotton, etc., which are generated at the tasting and cosmetics test corner, so that others cannot touch them.

● To keep the distance between users in the queue, such as admission and payment at least 2 m (at least 1 m). Guide clients to keep the distance through floor stickers, signs, etc. If it is difficult to keep a distance of more than 2 m, ask them to wear a mask and guide to keep a distance of at least 1 m.

● Ask employees not to follow customers who look around goods.

● Keep a distance of at least 2 m (at least 1 m) between the cashier/the receptionist and the user. Install transparent partitions if necessary.

● Put hand sanitizer near the shared shopping cart and shopping basket. Disinfect the handle from time to time.

● It is recommended to use electronic and contactless payment methods

● Minimize the operation of public facilities such as cultural centers and children’s play facilities. If operated, take measures to maintain distance between users.

*When using restaurants, cafes, etc. in the facility, the corresponding guidelines are applied.

**4-2. Guidelines for distance in daily life: traditional market**

**<User/visitor>**

**[Common information] [A-Common principles for user or visitor]**

*When using restaurants, cafes, etc. in the facility, the corresponding guidelines area applied.

**<Officer and employee>**

**[Common information] [B-Common principles for officers, employees]**

**[Specific information]**

● Avoid splattering acts such as loud guest call.

● If possible, refrain from conducting guest events and induce inevitable diversification

*When using restaurants, cafes, etc. in the facility, the corresponding guidelines are applied.

**4-3. Guidelines for keeping distance in your life: small and medium supermarket**

**<User>**

**[Common information] [A-Common principles for user or visitor]**

**[Specific practices]**

● Minimize clients’ time spent in shops

**<Officer and employee>**

**[Common information] [B-Common principles for officers, employees]**

**[Specific information]**

● In the place where clients stand in line, such as at a checkout counter, mark it so that there is a gap of 2 m (minimum 1 m) or more

**II. Daily life: 5. Special event**

**5-1. Guidelines for distance in life: family events such as weddings**

**<Visitor>**

**[Common information] [A-Common principles for user or visitor]**

**[Specific practice]**

● If possible, keep a distance between tables at least 2 m (at least 1 m).

● Look at one direction or sitting zigzag while facing each other during mealtime.

● When eating, eat each food on a separate plate.

● Make a congratulatory money online, if possible.

● Bow to the pastoral rather than shaking hands.

● Cooperate for the prevention of diseases, such as checking and listing symptoms, including fever, respiratory symptoms, etc.

**<Event organizer>**

**[Common information]**

● If anyone has a fever or respiratory symptoms or has traveled overseas within the last 14 days, refrain him or her from visiting any places. Keep the distance between people more than 2 m (at least 1 m).

● Guide persons to wear a mask when using indoor multi-use facilities.

● Guide persons to wear a mask if they cannot maintain a distance of 2 m outside.

**[Specific information]**

● Family events should be prepared as briefly as possible (or simple) or conducted online if necessary.

● Adjust the number of invitees so that they are not crowded considering the size of the venue.

● Postpone events if you have a fever or respiratory symptoms or have traveled overseas within the last 14 days.

● Provide enough guidance to the guests in advance of distance keeping in life.

● Consider distance keeping in life before the events.

● Avoid shaking hands. Provide gift rather than meals.

**<Officer and employee>**

**[Common information] [B-Common principles for officers, employees]**

**[Specific information]**

● Keep the distance between tables at least 2 m (at least 1 m) or at least 1 m away from fixed tables at the event.

● Arrange the chair to face one direction without facing each other; otherwise place chairs to zigzag.

● The event should be conducted with enough distance between visitors as far as possible.

● Conduct regular training for employees on compliance with personal hygiene rules and the necessity of keeping distance in their lives.

● Provide personal plates, scoops, and tongs for an individual meal.

● Checking the symptoms of people who enter and leave, including fever, respiratory symptoms, etc.

● Keep a list of visitors as much as possible and ask them to fill it out.

**5-2. Guidelines for distance in life: funeral homes**

**<Mourners and the family of the deceased>**

**[Common information] [A-Common principles for or visitor]**

**[Specific practice]**

● Wear a mask to greet the mourners. Greet them with a bow rather than a handshake.

● When dining in a reception room, seat in one direction without facing each other or seat in zigzag direction.

● When conveying condolences, bow preferably to express a feeling of comfort.

● Convey condolences as simple as possible and not to stay longer than 30 minutes.

● Family-member only funeral ceremony is recommended. Minimum number of people should participate in funeral procedures. Maintain a distance of 1 m between participants.

● Cooperate for the prevention of diseases, such as checking and listing symptoms, including fever, respiratory symptoms, etc.

**<Officer and employee>**

**[Common information] [B-Common principles for officers, employees]**

**[Applicable types]**

● Keep the distance between the tables at least 2 m (at least 1 m) as possible or maintain a distance such as not using some tables.

● Use the funeral ceremony room with a time interval.

● Ask the family of the deceased to invite the minimum number of people who can participate in the funeral ceremony.

● Ask the staff of the funeral hall and funeral services companies and salesperson of funeral supplies to comply with personal hygiene rules, including hand disinfection and wearing a mask, etc.

● Provide individual plates, scoops, and tongs to eat food on an individual plate.

● For empty rooms operate the ventilation system 2–3 times a day for 1 hour or more.

● Disinfect immediately after using a funeral room.

● Disinfect a funeral vehicle after driving.

● Check people who enter and leaver for their symptoms, including a fever and respiratory symptoms, etc.

● Keep the list of visitors and ask them to fill out the time of visiting, name, and cell phone number.

**II. Daily life: 6. Religious life**

**6-1. Daily life: religious facilities**

**<User>**

**[Common information] [A-Common principles for user or visitor]**

**[Applicable types]**

● Avoid group meals and, if unavoidable, maintain a gap between people (sit with one direction or sit alternatively, etc.).

● Use personal items (books, etc.).

● When taking a facility’s public vehicle, follow personal hygiene guidelines such as hand washing and mask-wearing.

● Provide the online, non-face-to-face, non-contact religious events.

● Avoid contact and face-to-face meetings.

● Wear a mask.

● High-risk groups, including senior citizens, pregnant women, chronic patients, etc. should refrain from visiting the religious facility and wear masks if visiting is unavoidable.

● Cooperate for the prevention of diseases, such as checking and listing symptoms, including fever, respiratory symptoms, etc.

**<Officer and employee>**

**[Common information] [B-Common principles for officers, employees]**

**[Applicable types]**

● Activate online, non-face-to-face, non-contact religious events; furthermore, minimize the large-scale events and group meetings.

● Arrange to keep the distance between participants of religious events more than 2 m (at least 1 m) and disperses the entrance and exit times.

● When using a microphone, be sure to use a cover and keep it for personal use whenever possible.

● Refrain from providing group meals and, if unavoidable, maintain a gap between people (sit with one direction, sit down alternatively, etc.).

● Disinfect doors and railings, especially places and objects that are frequently touched and ventilate before and after religious events.

● When operating a facility's public vehicle, provide the hand sanitizer in the vehicle, and ask passengers to wear a mask.

● Train and guide believers periodically on compliance with personal hygiene guidelines and the necessity of keeping distance in daily life.

● Ask all religious workers and believers to wear masks and provide disposable masks for visitors who do not wear masks

● High-risk groups, including senior citizens, pregnant women, chronic patients, etc. should be advised to refrain from visiting the religious facility. They should wear masks if visiting is unavoidable.

● Check people who enter and leaver for their symptoms, including fever and respiratory symptoms, etc.

● Keep the list of visitors and ask them to fill out the time of visiting, name and cell phone number.

**III. Leisure: 1. Traveling**

**1-1. Guidelines for distance in daily life: hotel and condominium**

**<User>**

**[Common information] [A-Common principles for user or visitor]**

**[Applicable types]**

● Cooperate for the prevention of diseases, such as checking and listing symptoms, including fever, respiratory symptoms, etc.

*When using restaurants, cafes, indoor gymnasiums, etc. in the hotel or condominium, apply the guidelines of the corresponding facilities.

**<Officer and employee>**

**[Common information] [B-Common principles for officers, employees]**

**[Applicable types]**

● Conduct disinfection and ventilate after the banquet event (write the ledger including date/time manager confirmation).

● Keep facing tissues and litter bins at entrances and facilities.

● After guest use, open the room window for ventilation, and to clean and disinfect the room and toilet.

● Train and guide employees periodically on compliance with personal hygiene guidelines and the necessity of keeping physical distance in daily life.

● Check people who enter and leaver for their symptoms, including fever and respiratory symptoms, etc.

*If there are restaurants, cafes, indoor gymnasiums, etc., follow the guidelines of the corresponding facilities.

**1-2. Guidelines for distance in daily life: amusement facilities**

**<User>**

**[Common information] [A-Common principles for user or visitor]**

**[Applicable types]**

● Use it in a dispersed way so that multi-use spaces such as indoor lounges, cafes, and shops are not crowded.

*When using restaurants, cafes, etc. in the facility, the corresponding guidelines are applied.

**<Officer and employee>**

**[Common information] [B-Common principles for officers, employees]**

**[Applicable types]**

● Prepare employee action guidelines to avoid physical contact with visitors and maintain a distance of 2 m (minimum 1 m) or more.

● Conduct disinfection of the devices (especially places and objects, such as handles, railings, etc. that are frequently touched by people) before and after boarding.

● Ask staff to use lounges and a dressing room with a different time interval.

● Prevent the crowdedness of visitors by limiting the number of visitors per hour, or by the operation of a reservation system.

● Encouraging the dispersion of customers by the advanced reservations rather than on-site sales of admission tickets, classifying entrance and exit times by zone, and managing queue to prevent crowding in public areas.

● When visitors take an amusement facility, allocate the seats in a zigzag direction.

● To maintain the physical distance in places where people are crowded, such as a queue, guide them to wait for entrance at least 2 m (at least 1 m) apart.

● Minimize large-scale events and joint activities.

● Maintain physical distance from others, e.g., viewing in one direction, alternative seat, etc. at mealtime or breaks.

● Keep facing tissues and litter bins at entrances and several points of facilities.

● Train and guide employees periodically on compliance with personal hygiene guidelines and the necessity of keeping physical distance in daily life.

*If there are restaurants and cafés in the facility, the corresponding guidelines are applied.

**1-3. Guidelines for distance in life: campsite**

**<User>**

**[Common information] [A-Common principles for user or visitor]**

**[Applicable types]**

● Refrain from visiting campsites by a large number of people other than family members. When installing tents, install at least 2 m away.

● Frequently ventilate camping facilities that are indoor spaces such as private tents, glamping, camping trailers, campers, etc.

● Maintain physical distance 2 m (at least 1 m) from other people when using public facilities in campsites such as management offices, kitchens, public sinks, and showers. Furthermore, wash hands frequently or use hand sanitizers frequently.

**<Officer and employee>**

**[Common information] [B-Common principles for officers, employees]**

**[Applicable types]**

● Disinfect and ventilate camping facilities (glamping, camping trailers) and public facilities (kitchens, showers, toilets, etc.).

● Prohibit group meals.

● Securing space in the campsite by limiting the number of daily visitors, such as operating a reservation system.

● When installing camper tents, guide them to keep a distance of at least 2 m.

● Arrange or display facilities so that users can maintain physical distance more than 2 m (at least 1 m) in public facilities in the camp, such as management offices, kitchens, public sinks, and showers.

**1-4. Guidelines for distance in life: zoo**

**<User>**

**[Common information] [A-Common principles for user or visitor]**

**[Applicable types]**

● Make a reservation for entrance rather than on-site purchase.

● Avoid direct contact with animals and wash and disinfect hands immediately before and after contact with animals if contacted.

*If there are restaurants and cafés in the facility, the corresponding guidelines are applied.

**<Officer and employee>**

**[Common information] [B-Common principles for officers, employees]**

**[Applicable types]**

**[Visitor guidance and campaigns (placards, posters, and announcements)]**

● Avoid contacting animals and disinfect hands immediately before and after contact if anyone contacted with animals.

● Do not stay in the same places such as popular animal cages or restaurants for a long time.

● Encourage online-reservations rather than on-site purchases to prevent crowding at the ticket window.

**[Animal infection prevention management]**

● When a manager (a zookeeper, veterinarian, etc.) comes into contact with animals, wear protective equipment such as masks and gloves.

● Immediately report it if there are any predictions and unusual symptoms regardless of animal infection to zoo manager, local government, and the Ministry of Environment.

**[Operation and facility management]**

● Do not allow visitors to feed animals that have a possibility of zoonosis.

● To guide the maintenance of the distance between people by attaching the floor mark at intervals of 2 m (at least 1 m) along the view queue. Refrain from holding events, events, etc.

● Prevent the crowdedness of visitors by limiting the number of visitors per hour, by the operation of a reservation system.

● Set the time difference for employees to rest in public areas such as lounges and dressing rooms.

● Keep 2 m (at least 1 m) apart from others during mealtimes or breaks. Furthermore, prohibit group meals.

● Keep hand sanitizers, facial tissues, and litter bins in the entrances and several points of facilities.

*If there are restaurants and cafés in the facility, the corresponding guidelines are applied.

**1-5. Guidelines for distance in daily life: national park**

**<Users and visitors>**

**[Common information] [A-Common principles for user or visitor]**

**[Applicable types]**

● Use hand sanitizer at the entrance to the trail and actively cooperate with staff’s guidance.

● Avoid group visits (walking) and minimize the number of visitors.

● Pass one line to the right on the trail.

● Do not eat face to face and do not share meals.

● When using multi-use facilities such as shelter and campsite, cooperate as follows: Measure body temperature according to the staff’s guide before entering (no access if above 37.5°C).

● Do not visit if you have a fever or respiratory symptoms.

● If you have traveled abroad in the last 2 weeks, do not visit facilities.

● Use public areas in the facility, including toilet, shower, etc. to minimize the contact with others.

*Other corresponding guidelines should be followed when using restaurants, cafes, accommodation, and campsites.

**<Facility operator and manager>**

**[Common information] [B-Common principles for officers, employees]**

**[Applicable types]**

**[COVID-19 management system and related organization cooperation system]**

● In each office, a quarantine manager is designated to assign responsibility for the prevention and management of COVID-19.

*He or she can use a checklist for the announcement, control of the environment, and hygiene.

● Establish an emergency report system with related organizations including city, province, county, district health center, and medical institute to immediately respond if there is a suspected case, or symptomatic persons, etc.

● Establish a hotline to connect with local governments if visitors show fever, respiratory symptoms, etc.

**[Information for the prevention of infectious diseases]**

● Promote the physical distance of visits by placards, text-message electronic board, announcements, etc.

- Keep more than 2 m away, do not stay long, and wear a mask in crowded places, including shelter, top, etc.).

- Keep the physical distance 2 m or more and walk-in right-side in trail.

- Use hand disinfection and wear a mask in public space including toilet, anti-tamper center, etc.

● Promotion of personal hygiene rules through placard, text-message electronic board, announcements, etc.).

- Follow COVID-19 prevention rules such as hand washing and coughing etiquette.

- Enter the park after using hand sanitizer at the entrance to the trail.

● Post a banner to measure body temperature, secure management personnel, and broadcast a message to encourage body temperature measurement.

**[Management of environmental hygiene]**

● Place hand sanitizer at the entrance to the trail.

● Prepare enough amount of soap and paper towels in the bathroom.

● Clean the main space. Disinfect periodically public facilities, including toilets, etc.

- Staff who work for cleaning and disinfection should take a piece of personal protection equipment: disposable gloves, mask, disposable waterproof long-sleeved gown or waterproof apron, and goggles or face protector if necessary.

- Disinfect surfaces of door handles, railings, switches, etc. that were frequently touched.

- Wipe with a cloth moistened with disinfectants such as 70% ethanol or diluted sodium hypochlorite (500–1,000 ppm).

**[Response to visitor]**

● Wear a mask, maintain a physical distance 2 m (at least 1 m) apart from visitors, ask visitors for one line passage.

● Keep personal hygiene including washing of hands and wearing a mask. when returning to the office.

● Maintain physical distance from others during meals, for example, sitting in one direction.

**[Operation and management of multi-use facilities such as shelter, campsite, and visitor]**

● Prohibit the use of facilities by symptomatic persons or those who have traveled overseas within the last 14 days.

● When placing a seat in a campsite or shelter, place them at least 2 m apart (at least 1 m) from each other.

● To maintain a physical distance in public areas including toilets, shower room, etc., do not let people stay for long periods.

● Informa visitors on wearing masks and prohibit those who do not wear masks to enter indoor facilities.

● Facilities should be ventilated at least twice per hour, at least 2 hours per day, and be sterilized at least once per day.

**III. Leisure: 2. Leisure, etc.**

**2-1. Guidelines for distance in life: outdoor activities**

**[Common information] [A-Common principles for user or visitor]**

**[Applicable types]**

● When purchasing admission tickets to amusement parks, tourist attractions, etc., reserve the ticket online.

● Refrain from entering a crowded area.

● When moving, move to the right so that the passage line does not overlap with the person coming across.

**2-2. Guidelines for distance in daily life: public toilets, etc.**

**<User>**

**[Common information] [A-Common principles for user or visitor]**

**[Applicable types]**

● Strictly use it cleanly because it is the public facilities used by many people.

● After using the toilet bowl, close the toilet lid, and flush it.

● Dispose of toilet paper, into the toilet. Sanitary products should be discarded in the sanitary product collection box.

**<Manager>**

**[Common information] [B-Common principles for officers, employees]**

**[Applicable types]**

**[Disinfection management such as disinfection]**

● Designate the department head in charge of quarantine management to observe the disinfection and sterilization.

**[If possible, refer to a professional sterilization company]**

● In the public toilet facility disinfection should be carried out frequently.

● Do not spray the disinfectant using a spray.

● With cleansing towel with 70% ethanol or diluted sodium hypochlorite (500–1,000 ppm), etc., wipe points frequently touched, including door handle, switch, toilet cover and lid, flush button, sink, faucet, hand dryer, diaper changing table, handle for a handicapped person, etc.

● Repeat disinfect floor from one end to the others.

● After disinfection, dry the space sufficiently dried. After that, open the space.

● Put a sign or safety belt at the entrance of the toilet to inform the user that it is being cleaned or disinfected so that the user can know.

**[Hygiene and facility management]**

● To prevent trash, etc. from being left for a long time, empty the litter and hygiene items collection box frequently.

● Periodically clean facilities such as public toilets including a urinal, a toilet stool, and diaper changing table, and hand dryer, etc.

● Frequently check the facilities in the public restroom to prevent them from being broken and left unattended.

*Public toilets exposed to the COVID-19 infected persons, open it after disinfection and kind of disinfectants. The virus is killed after disinfection immediately; however, the disinfectants remain in the air of the toilet. Therefore, the opening of the toiled should be decided after considering the exposure to the disinfectants.

**[Promotion management]**

● Promote active hygiene management for public toilet users.

- Wash your hands with soap in running water for more than 30 seconds, use the toilet cleanly, wear a mask in the public toilet, line up every 2 m (at least 1 m), close the toilet lid and flush, request to refrain from using suspicious boxes.

- Mark the floor so that 2 m (at least 1 m) of the toilet line is maintained.

- Install the separator so that the entry and exit lines do not overlap if there is space in the entrance and exit.

● Train sanitation managers of the public toilets for quarantine guidelines.

*Avoid unnecessary face-to-face group education and promote through documents.

**2-3. Guidelines for distance in daily life: beauty shop and barbershop**

**<User>**

**[Common information] [A-Common principles for user or visitor]**

**[Applicable types]**

● Cooperate when staff request clients to leave the shop for disinfection/cleaning/ventilation or when they refuse access due to overseas travel, fever, or respiratory symptoms of the clients.

● Cooperate with quarantine measure, such as checking for symptoms including fever, respiratory symptoms, etc. when entering.

**<Officer and employee>**

**[Common information] [B-Common principles for officers, employees]**

**[Applicable types]**

● Space between facilities (chair, bed, etc.) should be apart more than 2 m (at least 1 m), or crossing 1 space.

● Check clients if there were symptoms including fever, respiratory symptoms, etc., when entering and leaving.

**2-4. Guidelines for distance in life: public bathhouse**

**<User>**

**[Common information] [A-Common principles for user or visitor]**

**[Applicable types]**

● Cooperate when staff request clients to leave the bathhouse for disinfection/cleaning/ventilation or when they refuse access due to overseas travel, fever, or respiratory symptoms of the clients.

● Cooperate with quarantine measure, such as checking for symptoms including fever, respiratory symptoms, etc. when entering.

**<Officer and employee>**

**[Common information] [B-Common principles for officers, employees]**

**[Applicable types]**

● Ventilate from time to time, especially, closed spaces such as Finnish saunas.

*Disinfection/cleaning/ventilation time should be set in advance and published in a well visible place.

● Check the clients if they show the symptoms including fever, respiratory symptoms, etc., when entering and leaving.

**2-5. Guidelines for distance in life: library**

**<User>**

**[Common information] [A-Common principles for user or visitor]**

**[Applicable types]**

● Use it in a dispersed way so that multi-use spaces such as indoor lounges, cafes, and shops are not crowded.

● Cooperate with quarantine measure, such as checking for symptoms including fever, respiratory symptoms, etc. when entering.

*When using restaurants, cafes, etc. in the facility, the corresponding guidelines are applied.

Officer and employee.

**[Common information] [B-Common principles for officers, employees]**

**[Applicable types]**

● Prevent crowdedness by limiting the number of users and usage space by time zone.

● Operate with related equipment and training courses to enable online service.

● Refrain from large-scale events and joint activities

● Avoid training and events, but in case of conducting training and events, prepare a plan (adjustment of desk spacing, etc.) to maintain the participants' physical distance of 2 m (minimum 1 m).

● When using computer products such as laptops and tablet PCs, employees should use personal devices whenever possible.

● Periodic education and guidance on compliance with personal hygiene rules and the necessity of keeping physical distance in daily life.

● Place seats in a zigzag manner so that the next and front seats are empty, or install partitions such as transparent partitions.

● Check if users show the symptoms, including fever, respiratory symptoms, etc., when entering and leaving.

*If there are restaurants and cafés in the facility, the corresponding guidelines are applied.

**2-6. Guidelines for distance in daily life: concert hall**

**<Users and visitors>**

**[Common information] [A-Common principles for user or visitor]**

**[Applicable types]**

● Pre-book online as much as possible when purchasing admission tickets.

● Enter the hall slowly after arriving earlier.

● Use the multi-use spaces such as indoor lounges, cafes, and kiosks scatteringly so that those spaces are not crowded.

● Cooperate with quarantine measure, such as checking for symptoms including fever, respiratory symptoms, etc. when entering.

*When using restaurants, cafes, etc. in the facility, the corresponding guidelines are applied.

**<Officer and employee>**

**[Common information] [B-Common principles for officers, employees]**

**[Applicable types]**

● A ticket agent should use hand-sanitizer frequently. If she or he wears sanitary gloves, replace it frequently.

● Maintain the maximum distance between the stage and the audience (at least 2 m).

● Be sure to ventilate after the performance and disinfect the auditorium and stage facilities.

● Keep facing tissues and litter bins at entrances and the several points of facilities.

● Train employees on compliance with personal hygiene rules and the necessity of keeping distance in daily life.

● Ask the audience to enter the hall slowly to avoid crowding.

● In the case of moving or standing in line, take measures such as a floor sticker that can induce a distance of 2 m (at least 1 m) from others.

● Manage multi-use spaces such as indoor lounges, cafes, and kiosks so that users are not crowded.

● Advance seating is provided so that the seats are zigzag one space apart.

● Ask clients to wear masks in the hall and refrain them from eating food.

● Check if audiences show the symptoms including fever, respiratory symptoms, etc., when entering and leaving.

*If there are restaurants and cafés in the facility, the corresponding guidelines are applied.

**2-7. Guidelines for distance in life: movie theater**

**<Users and visitors>**

**[Common information] [A-Common principles for user or visitor]**

**[Applicable types]**

● Pre-book online as much as possible when purchasing admission tickets.

● Enter the hall slowly after arriving earlier.

● Enter the multi-use spaces such as indoor lounges, cafes, and kiosks scatteringly so that those spaces are not crowded.

● When watching a movie, make a reservation so that the seats are zigzag one space apart.

● Wear masks in movie theaters and refrain from eating food.

● Cooperate with quarantine measure, such as checking for symptoms including fever, respiratory symptoms, etc. when entering.

*When using restaurants, cafes, etc. in the facility, the corresponding guidelines are applied.

**<Officer and employee>**

**[Common information] [B-Common principles for officers, employees]**

**[Applicable types]**

● A ticket agent should use hand-sanitizer frequently. If she or he wears sanitary gloves, replace it frequently.

● After movie screening, be sure to ventilate sufficiently and disinfect the areas where the hands are frequently touched, such as the seat armrests.

● Refrain from large-scale attendance promotion events.

● Train employees on compliance with personal hygiene rules and the necessity of keeping distance in daily life.

● Ask the audience to enter the theater slowly to avoid crowding.

● In the case of moving or standing in line, take measures such as a floor sticker that can induce a distance of 2 m (at least 1 m) from others.

● Manage multi-use spaces such as indoor lounges, cafes, and kiosks so that users are not crowded.

● Advance seating is provided so that the seats are zigzag one space apart.

● Ask the audience to wear masks in the hall and refrain them from eating food.

● Check if audiences show the symptoms including fever, respiratory symptoms, etc., when entering and leaving.

*If there are restaurants and cafés in the facility, the corresponding guidelines are applied.

**2-8. Guidelines for keeping distance in your life: museums and art galleries**

**<Users and visitors>**

**[Common information] [A-Common principles for user or visitor]**

**[Applicable types]**

● Keep a distance of at least 2 m (at least 1 m) from other people, during the watching an exhibition, moving, or standing inline.

● Use the multi-use spaces such as indoor lounges, cafes, and kiosks scatteringly so that those spaces are not crowded.

*When using restaurants, cafes, etc. in the facility, the corresponding guidelines are applied.

**<Officer and employee>**

**[Common information] [B-Common principles for officers, employees]**

**[Applicable types]**

● Operate with related equipment and training courses to enable online services.

● Provide employee the guidelines to avoid physical contact with visitors and maintain a distance of 2 m (minimum 1 m).

● Keep facing tissues and litter bins at entrances and several points of facilities.

● Refrain from education course and events.

● When using computer products such as laptops and tablet PCs, employees should use personal devices whenever possible.

● Ask employees to use public areas such as employee's restrooms and dress rooms with intervals from others.

● Prepare isolated space for symptomatic users and employees and prepare for emergency measures for suspicious cases.

● Train employees periodically on compliance with personal hygiene rules and the necessity of keeping physical distance in daily life.

● Prevent the crowdedness of visitors by limiting the number of visitors per hour, by the operation of a reservation system.

*If there are restaurants and cafés in the facility, the corresponding guidelines are applied.

**2-9. Guidelines for keeping distance in your life: baseball field, soccer field**

**<Users and visitors>**

**[Common information] [A-Common principles for user or visitor]**

**[Applicable types]**

● Pre-book online as much as possible when purchasing admission tickets.

● Refrain from eating food in the stadium.

● Use personal items for sports equipment, cheering tools, sportswear, and towels.

● Avoid using public facilities such as dress rooms and showers.

● When you watch a game, make a reservation so that the seats are zigzag one space apart.

*If there are restaurants and cafés in the facility, the corresponding guidelines are applied.

*This guideline also can be used to other sports facilities.

**<Officer and employee>**

**[Common information] [B-Common principles for officers, employees]**

**[Applicable types]**

● Check if the player has aa fever when he or she stays in their team accommodation.

● Provide independent space for symptomatic users and employees

● Thoroughly disinfect areas with profoundly touched, such as toilets, shared facilities, inside and outside the stadium, squad lockers, and accommodations.

● Train employees on compliance with personal hygiene rules and the necessity of keeping distance in daily life.

● Encourage the diversification of customers, such as encouraging reservations rather than on-site sales of admission tickets, classifying entrance and exit times by zone, and managing moving lines to prevent crowding in public areas.

● Refrain from acts and events that induce contact between people, including high five, autograph sessions, handshake events, etc.

● Ask visitors to throw away the used tissues appropriately by placing trash bis with lids all over the facility.

● Advance ticketing is provided so that the seats are zigzag one space apart.

*If there are restaurants and cafés in the facility, the corresponding guidelines are applied.

*The guideline can be applied to other sports facilities.

**2-10. Guidelines for distance in daily life: karaoke**

**<User>**

**[Common information] [A-Common principles for user or visitor]**

**[Applicable types]**

● Cover the microphone for personal use.

● High-risk groups, including senior citizens, pregnant women, chronic disease patients, etc. should be refrained from using the facility and wear masks if unavoidable.

● Cooperate with quarantine measure, such as checking for symptoms including fever, respiratory symptoms, etc. when entering.

*If there are restaurants and cafés in the facility, the corresponding guidelines are applied.

**<Officer and employee>**

**[Common information] [B-Common principles for officers, employees]**

**[Applicable types]**

● Keep facial tissues and litter bins at entrances and several points of facilities.

● Train employees on compliance with personal hygiene rules and the necessity of keeping distance in daily life.

● Prepare enough number of microphone cover.

● Manage multi-use spaces such as indoor lounges, cafes, and kiosks so that users are not crowded.

● High-risk groups including senior citizen, pregnant women, chronic diseases patients, etc. are advised to refrain from using the facility and wear masks if unavoidable.

● Check if visitors show the symptoms including fever, respiratory symptoms, etc., when entering and leaving.

● Keep a list of visitors and ask them to fill it out.

*If there are restaurants and cafés in the facility, the corresponding guidelines are applied.

**2-11. Guidelines for distance in daily life: indoor sports facilities**

**<User>**

**[Common information] [A-Common principles for user or visitor]**

**[Applicable types]**

● It is recommended to use personal items for sportswear, towels, and portable exercise equipment.

● Avoid using public facilities such as dress rooms and showers.

● After using exercise equipment, clean the surface of the equipment with disinfecting supplies provided by the operator.

● High-risk groups including senior citizen, pregnant women, chronic diseases patients, etc. should refrain from using the facility and wear masks if unavoidable.

● Cooperate with quarantine measures, such as checking for symptoms, including fever, respiratory symptoms, etc.

*Outdoor sports facility' applies the same guidelines to this one.

**<Officer and employee>**

**[Common information] [B-Common principles for officers, employees]**

**[Applicable types]**

● Guide physical trainers, instructors, and users to wear masks and to avoid physical contact.

● It is recommended to use personal items such as sportswear, towels, and exercise equipment (individual portable products), and thoroughly disinfect when providing everyday items.

● Thoroughly disinfect auxiliary facilities such as dress rooms (locker rooms), shower rooms, and waiting rooms (relaxation rooms).

● Train employees on compliance with personal hygiene rules and the necessity of keeping distance in daily life.

● Manage the visitors s to maintain a physical distance of 2 m (minimum 1 m).

● Manage the appropriate number of people in the auxiliary facilities such as dressing room (locker room), shower room, the waiting room.

● Everybody should wear a mask on the commuter bus. Disinfect and ventilate it before and after boarding.

● Avoid group exercise programs including Zumba, Taebo, Spinning, etc., for a large number of students in a closed place, but, if implemented, maintain appropriate intervals (2 m) disinfection.

● High-risk groups, including senior citizens, pregnant women, chronic patients, etc. should be refrained from using the facility. They should wear masks if the visit is unavoidable.

● Check if visitors show the symptoms including fever, respiratory symptoms, etc., when entering and leaving.

● Keep a list of visitors and ask visitors to fill it out. For outdoor sports facility, the same guidelines can be adopted.

**2-12. Guidelines for distance in daily life: PC room**

**<User>**

**[Common information] [A-Common principles for user or visitor]**

**[Applicable types]**

● Seats are separated by one space.

● High-risk groups, including senior citizens, pregnant women, chronic disease patients, etc., should refrain from using the facility. They should wear masks if the visit is unavoidable.

● Cooperate with quarantine measure, such as checking for symptoms including fever, respiratory symptoms, etc. when entering.

**<Officer and employee>**

**[Common information] [B-Common principles for officers, employees]**

**[Applicable types]**

● Keep facial tissues and litter bins at entrances and several points of facilities.

● Train employees on compliance with personal hygiene rules and the necessity of keeping distance in daily life

● Seating is guided so that the seats are separated by one space.

● High-risk groups, including senior citizens, pregnant women, chronic disease patients, etc. are advised to refrain from using the facility. They should wear masks if visiting is unavoidable.

● Check if visitors show the symptoms including fever, respiratory symptoms, etc., when entering and leaving.

● Keep a list of visitors as much as possible and ask visitors to fill it out.

**2-13. Guidelines for distance in daily life: entertainment facilities**

**<User>**

**[Common information] [A-Common principles for user or visitor]**

**[Applicable types]**

● Minimizing the time spent staying at entertainment facilities.

● Sit at a distance of 2 m (at least 1 m) between tables or sit as far apart as possible from other people.

● Sit so that you can face one direction without facing each other.

● Avoid conversation when eating.

● Take food on your plate.

● Don not recommend drinking.

● High-risk groups including senior citizen, pregnant women, chronic diseases patients, etc. should refrain from using the facility and wear masks when unavoidable visits.

● Cooperate with quarantine measure, such as checking for symptoms including fever, respiratory symptoms, etc. when entering.

**<Officer and employee>**

**[Common information] [B-Common principles for officers, employees]**

**[Applicable types]**

● Avoid facing customers as much as possible by installing non-facing devices or transparent partitions, etc.

● Prepare a method to keep the distance between tables, such as placing a space between tables at least 2 m (at least 1 m) or installing partitions between tables, and prohibiting the use of some fixed tables.

● Try not to face each other, such as placing the chairs in one direction or zigzag.

● Avoid opening large events.

● Train employees on compliance with personal hygiene rules and the necessity of keeping distance in daily life.

● When waiting occurs, use a number tag or guide people to wait at a distance of 1 m between the waiting persons.

● Provide individual scoop, tongs, etc. to eat the meal with the individual plate.

● If there is a karaoke machine, replace the microphone cover for each guest.

● High-risk groups, including senior citizens, pregnant women, chronic disease patients, etc. are advised to refrain from using the facility. They should wear masks of visiting is unavoidable.

● Check if audiences show the symptoms including fever, respiratory symptoms, etc., when entering and leaving

● Keep a list of visitors as much as possible and ask them to fill it out.
