# Supplementary material for: Rules and guidelines for distancing in daily life to control coronavirus disease 2019 in Korea: 3rd version, announced on July 3, 2020
Source: J Educ Eval Health Prof. 2020 Jul 13;17:20. doi: 10.3352/jeehp.2020.17.20 (PMC7403533; doi:10.3352/jeehp.2020.17.20)
Supplement: Supplementary file 3 — Supplement 3. Rules and guidelines for distancing in daily life to control COVID-19 in Korea, 3rd version announced on July 3, 2020 (original Korean version) [file jeehp-17-20-suppl3.pdf]

생활 속 거리 두기 세부지침(3판)

2020. 7. 3.

중앙재난안전대책본부

코로나19 생활 속 거리 두기 수칙과 지침 안내

개요·구성 및 활용

○ **(생활 속 거리 두기란?) 코로나19의 장기유행에 대비**하여 국민의 **일상 생활**과 **경제활동**을 보장하면서, 코로나19 유행 차단을 위한 **감염 예방 및 차단 활동이 함께 조화**되도록 전개하는 **생활습관과 사회구조 개선**

* 코로나19 확산 시 **고강도 사회적 거리 두기로 전환**하는 등 유연한 대응

○ (기본원리) 개인과 공동체가 함께 코로나19 바이러스의

▴ 생활공간 침입 차단 ▴ 생존 환경 제거 ▴ 몸 밖 배출 최소화

▴ 전파경로 차단을 위한 수칙을 알고 실천해 공동체를 보호

○ (구성) 개인방역(5대 핵심수칙과 4개 보조수칙), **집단방역**(5대 핵심수칙과 각 부처별 세부시설 지침) 2개 영역으로 구성

- (개인방역) 개인이 지켜야 할 **5가지 핵심수칙**(수칙별 4~5개 행동요령)과 **4가지 보조수칙**(마스크, 환경소독, 고위험군, 건강생활)으로 **구성**

- (집단방역) 개인과 공동체가 지켜야 할 5가지 **핵심수칙** 및 각 부처별 세부시설 지침으로 구성

○ (활용) 개인과 공동체는 개인 및 집단방역 수칙, 지침 등을 참고하고 필요 시 상황 및 여건에 맞는 별도의 생활 속 거리 두기 지침 등을 마련해 일상에서 실천

목 차

□ 기본지침

1. 개인방역 5대 핵심수칙 ································ 2

2. 개인방역 4개 보조수칙 ································ 5

3. 집단방역 5대 핵심수칙 ································ 10

□ 세부지침

Ⅰ. 업 무 (11분야)

1. 일할 때

1-1. 사업장 ·· 15

1-2. 회의 ·· 18

1-3. 민원창구 ·· 19

1-4. 우체국 ·· 22

1-5. 국내출장 ·· 24

1-6. 방문서비스 ·· 25

1-7. 콜센터 ·· 28

1-8. 건설업 ·· 31

1-9. 은행지점 ·· 34

1-10. 물류센터 ·· 36

1-11. 전시행사 ·· 39

목 차

Ⅱ. 일 상 (18분야)

1. 이동할 때

1-1. 대중교통 ········ 44
1-2. 여객선(국제·연안) ········ 46

2. 식사할 때

2-1. 음식점·카페/스터디카페 ········ 49

3. 공부할 때

3-1. 학원·독서실 등 ········ 58
3-2. 고시원 ········ 60
3-3. 연수시설 ········ 63
3-4. 학술행사 ········ 66

4. 쇼핑할 때

4-1. 대형유통시설(백화점, 대형마트 등) ········ 70
4-2. 전통시장 ········ 73
4-3. 중소슈퍼 ········ 75

5. 특별한 날

5-1. 결혼식 등 가족 행사 ········ 78
5-2. 장례식장 ········ 81
5-3. 산후조리원 ········ 83
5-4. 기념식 ········ 86

6. 종교생활

6-1. 종교시설 ········ 90

7. 병·의원 갈 때

7-1. 병·의원(외래진료 및 면회) ········ 94

8. 에어컨 사용할 때

8-1. 에어컨 사용 ········ 97

9. 공동생활

9-1. 기숙사 ········ 99

목 차

Ⅲ. 여 가 (23분야)

1. 여행할 때

1-1. 호텔·콘도업 ········ 103
1-2. 유원시설(워터파크) ········ 105
1-3. 야영장 ········ 108
1-4. 동물원 ········ 110
1-5. 국립공원 ········ 112
1-6. 해수욕장 ········ 116
1-7. 하천·계곡 ········ 119
1-8. 수상레저 ········ 122
1-9. 지역축제 ········ 125

2. 여가 등

2-1. 야외 활동 ········ 130
2-2. 공중화장실 등 ········ 131
2-3. 이·미용업 ········ 134
2-4. 목욕장업(목욕탕, 찜질방) ········ 136
2-5. 도서관 ········ 141
2-6. 공연장 ········ 143
2-7. 영화상영관 ········ 145
2-8. 박물관·미술관 ········ 147
2-9. 야구장·축구장 ········ 149
2-10. 노래연습장 ········ 151
2-11. 실내체육시설 ········ 153
2-12. 수영장 ········ 155
2-13. 피시(PC)방 ········ 158
2-14. 유흥시설 ········ 160

< 지침별 담당부처 연락처 >

지침명	소관부처	연락처
▶ 총괄	중앙사고수습본부	044-202-3805
	중앙방역대책본부	043-719-9081
▶ 사업장·회의	고용노동부 산업보건과	044-202-7744
▶ 민원창구	행정안전부 민원제도혁신과	044-205-2441
▶ 우체국	우정사업본부 운영지원과(과기정통부)	044-200-8832
▶ 국내출장·방문서비스·콜센터	고용노동부 산업보건과	044-202-7744
▶ 건설업	국토교통부 건설산업과	044-201-3546
▶ 은행지점	금융감독위원회 은행과	02-2100-2951
▶ 물류센터	국토교통부 물류시설정보과	044-201-3786
▶ 전시행사	산업통상자원부 무역진흥과	044-203-4037
▶ 대중교통	국토교통부 교통정책조정과	044-201-3786
▶ 여객선	해양수산부 해운정책과·연안해운과	044-200-5718, 5738
▶ 음식점·카페/스터디카페	식품의약품안전처 식품안전관리과	043-719-2054
▶ 학원·독서실 등	교육부 평생학습정책과	044-203-6380
▶ 대형유통시설	산업통상자원부 유통물류과	044-203-4381, 4383
▶ 전통시장	중소벤처기업부 전통시장육성과	042-481-4581
▶ 중소슈퍼	중소벤처기업부 소상공인경영지원과	042-481-4490
▶ 결혼식 등 가족행사	여성가족부 가족문화과	02-2100-6363
▶ 장례식장	보건복지부 노인지원과	044-202-3473
▶ 산후조리원	보건복지부 출산정책과	044-202-3398
▶ 종교시설	문화체육관광부 종무1담당관	044-203-2317
▶ 병·의원	보건복지부 의료기관정책과	044-202-2471
▶ 호텔·콘도업	문화체육관광부 관광산업정책과	044-203-2871

지침명	소관부처	연락처
▶ 유원시설(워터파크 등)	문화체육관광부 관광산업정책과	044-203-2863
▶ 야영장	문화체육관광부 관광산업정책과	044-203-2866
▶ 동물원	환경부 생물다양성과	044-201-7244
▶ 국립공원	환경부 자연공원과	044-201-7313
▶ 해수욕장	해양수산부 해양레저관광과	044-200-5254
▶ 하천 · 계곡	행정안전부 재난안전점검과	044-205-4249
▶ 수상레저	해양경찰청 수상레저과	032-835-2352
▶ 지역축제	문화체육관광부 국내관광진흥과	044-203-2854
	행정안전부 재난안전점검과	044-205-4256
▶ 야외 활동	문화체육관광부 관광산업정책과	044-203-2888
▶ 공중화장실 등	행정안전부 생활공간정책과	044-205-3545
▶ 이·미용업	보건복지부 건강정책과	044-202-2881
▶ 목욕장업	보건복지부 건강정책과	044-202-2881
▶ 도서관	문화체육관광부 도서관정책기획단	044-203-2612
▶ 공연장	문화체육관광부 공연전통예술과	044-203-2732
▶ 영화상영관	문화체육관광부 영상콘텐츠산업과	044-203-2432
▶ 박물관·미술관	문화체육관광부 문화기반과	044-203-2638
▶ 야구장·축구장	문화체육관광부 스포츠산업과	044-203-3153
▶ 노래연습장	문화체육관광부 대중문화산업과	044-203-2464
▶ 실내체육시설	문화체육관광부 스포츠산업과	044-203-3156
▶ 수영장	문화체육관광부 스포츠산업과	044-203-3156
▶ 피시(PC)방	문화체육관광부 게임콘텐츠산업과	044-203-2446
▶ 유흥시설	식품의약품안전처 식품안전관리과	043-719-2054

기본지침

1 개인방역 5대 핵심수칙

【핵심 수칙 메시지】

○ **(제 1 수칙)** “아프면 3∼4일 집에 머물기”

○ **(제 2 수칙)** “사람과 사람 사이, 두 팔 간격 건강 거리 두기”

○ **(제 3 수칙)** “30초 손 씻기, 기침은 옷소매”

○ **(제 4 수칙)** “매일 2번 이상 환기, 주기적 소독”

○ **(제 5 수칙)** “거리는 멀어져도 마음은 가까이”

< 핵심 수칙 >

제 1 수칙) “아프면 3~4일 집에서 쉽니다”

(이유) 코로나19는 증상이 가벼운 초기에도 전염될 수 있습니다. 열이 나거나 호흡기 증상이 있을 때 다른 사람과의 접촉을 최대한 줄이면, 혹시 있을지 모를 코로나19의 전파 가능성을 줄일 수 있습니다.

① 열이 나거나 기침, 가래, 인후통, 코막힘 등 호흡기 증상이 있으면, 집에 머물며 3~4일간 쉽니다.

② 증상이 있으면 주변 사람과 만나는 것을 최대한 삼가고, 집 안에 사람이 있으면 마스크를 쓰고 생활합니다. 특히 고령자·기저질환자와의 대화·식사 등 접촉을 자제합니다.

③ 휴식 후 증상이 없어지면 일상에 복귀하고, 휴식 중에 38도 이상 고열이 지속되거나 증상이 심해지면 콜센터(☎1339, ☎지역번호+120)나 보건소에 문의합니다.

④ 병원 또는 약국에 가거나 생필품을 사기 위해 어쩔 수 없이 외출을 해야 할 때에는 꼭 마스크를 씁니다.

⑤ 기업, 사업주 등은 증상이 있는 사람이 출근하지 않게끔, 또는 집으로 돌아가 쉴 수 있도록 돕습니다.

제 2 수칙) “사람과 사람 사이에는 두 팔 간격으로 충분한 거리를 둡니다”

(이유) 코로나19는 주로 침방울을 통해 전파됩니다. 사람과 사람 사이 거리를 2m 이상 두는 경우 대화, 기침, 재채기 등을 통해 침방울이 튀는 위험을 줄여 코로나19의 감염 가능성을 줄일 수 있습니다.

	① 환기가 안 되는 밀폐된 공간 또는 사람이 많이 모이는 곳은 되도록 가지 않습니다.
	② 일상생활에서 사람과 사람 사이에 2m의 거리, 아무리 좁아도 1m 이상의 거리를 둡니다.
	③ 다른 사람과 충분한 거리를 유지할 수 있도록 자리를 배치합니다.
	④ 많은 사람들이 모여야 할 경우 2m 간격을 유지할 수 있는 공간을 확보하거나 모이는 시간을 서로 다르게 합니다.
	⑤ 만나는 사람과 악수 혹은 포옹을 하지 않습니다.
**제 3 수칙) "손을 자주 꼼꼼히 씻고, 기침할 때 옷소매로 가립니다"**	
(이유) 오염된 손을 거쳐 바이러스가 몸에 들어오는 것을 막고, 기침 예절을 지켜 침방울을 통한 전파도 최소화합니다.	
	① 식사 전, 화장실 이용 후, 외출 후, 코를 풀거나 기침 또는 재채기를 한 후에는 흐르는 물과 비누로 30초 이상 손을 씻거나, 손 소독제를 이용해 손을 깨끗이 합니다.
	② 씻지 않은 손으로 눈, 코, 입을 만지지 않습니다.
	③ 개인·공용장소에는 쉽게 손을 씻을 수 있는 세수대와 비누를 마련하거나 곳곳에 손 소독제를 비치합니다.
	④ 기침이나 재채기를 할 때는 휴지 혹은 옷소매 안쪽으로 입과 코를 가립니다.
	⑤ 발열, 기침, 가래, 인후통, 코막힘 등의 증상이 있거나 몸이 안 좋다고 생각되면 다른 이들을 위해 마스크를 착용합니다.
**제 4 수칙) "매일 2번 이상 환기하고, 주기적으로 소독합니다"**	
(이유) 환기를 통해 코로나19 바이러스가 들어 있는 침방울의 공기 중 농도를 낮출 수 있고, 바이러스가 포함된 침방울이 묻을 수 있는 곳을 소독하면 손을 통한 코로나19의 감염 가능성을 줄일 수 있습니다.	
	① 자연 환기가 가능한 경우 창문을 항상 열어두고, 계속 열지 못하는 경우는 주기적으로(매일 2회 이상) 환기합니다. 환기를 할 때는 가능하면 문과 창문을 동시에 열어 놓습니다. 미세먼지가 있어도 실내 환기는 필요합니다.

	② 가정, 사무실 등 일상적 공간은 항상 깨끗하게 청소하고, 손이 자주 닿는 곳(전화기, 리모콘, 손잡이, 문고리, 탁자, 팔걸이, 스위치, 키보드, 마우스, 복사기 등)은 주 1회 이상 소독합니다.
	③ 공공장소 등 여럿이 오가는 공간은 손이 자주 닿는 곳(승강기 버튼, 출입문, 손잡이, 난간, 문고리, 팔걸이, 스위치 등)과 공용 물건(카트 등)을 매일 소독합니다.
	④ 소독을 할 때는 소독제(소독제 티슈, 알코올(70% 에탄올), 차아염소산나트륨(일명 가정용 락스 희석액 등)에 따라 제조사의 권고사항을 준수(용량과 용법 등)하여 안전하게 사용합니다.
**제 5 수칙) “거리는 멀어져도 마음은 가까이 합니다”**	
(이유) 코로나19는 혼자가 아니라 우리 모두의 노력이 있어야 극복할 수 있습니다. 서로를 배려하고 위로하며 함께 노력하는 사회를 만들어야 합니다.	
	① 모이지 않더라도 가족, 가까운 사람들과 자주 연락하는 등 마음으로 함께 할 기회를 만듭니다.
	② 공동체를 위한 나눔과 연대를 생각하고, 코로나19 환자, 격리자 등에 대한 차별과 낙인에 반대합니다.
	③ 소외되기 쉬운 취약계층을 배려하는 마음을 나누고, 실천합니다.
	④ 의심스러운 정보를 접했을 때 신뢰할 수 있는지 출처를 확인하고, 정확하지 않은 소문은 공유하지 않으며, 과도한 미디어 몰입을 삼갑니다.

2	개인방역 4개 보조수칙

1 마스크 착용

< 생활 속 거리 두기 실천지침 : 마스크 착용 >

1 마스크 착용 일반 원칙 및 올바른 착용 방법

<일반 원칙>

- ㅇ 마스크 착용보다 손 씻기, 사람 간의 거리 두기 등이 코로나19 감염 예방에 더 효과적이며 중요합니다.
- ㅇ 마스크는 침방울을 통한 감염 전파를 차단하는 효과가 있습니다.
- ㅇ 감염 위험이 있는 경우, 기저질환이 있는 고위험군에는 보건용·수술용 마스크 착용을 권장하며, 보건용·수술용 마스크가 없을 경우 면마스크 착용도 도움이 됩니다.

<올바른 착용 방법>

- ㅇ 개인의 얼굴 크기에 맞는 적당한 마스크를 선택하여 호흡기인 코와 입을 완전히 덮도록 얼굴에 잘 밀착해 착용합니다.
- ㅇ 마스크 자체가 오염되지 않도록 마스크를 만지기 전에 손을 깨끗이 씻습니다.
- ㅇ 마스크 착용 시에는 손을 통한 오염을 방지하기 위하여 마스크를 최대한 만지지 않습니다. 만졌다면, 30초 이상 비누로 손을 씻거나 손 소독제로 손을 깨끗이 해야 합니다.
- ㅇ 마스크 사용 후에는 마스크 앞면에 손을 대지 않고 벗습니다. 보건용 마스크의 경우 아무 곳에나 두지 말고 즉시 쓰레기통에 버리고 손을 씻습니다. 면마스크의 경우 제품 특성에 맞는 방법으로 자주 세탁합니다.
- ㅇ 마스크 내부에 휴지나 수건을 덧대면 공기가 새거나 밀착력이 떨어져 차단 효과가 낮아지므로 주의해야 합니다.
- ㅇ 오염 우려가 적은 곳에서 일시적으로 사용하는 경우 동일인에 한하여 재사용하실 수 있습니다.

2 다음의 경우는 마스크 착용을 권장합니다.

- ㅇ 코로나19 의심자를 돌보는 경우 (KF 94 이상)
- ㅇ 기침, 재채기, 가래, 콧물, 목 아픔 등 호흡기 증상이 있는 경우
- ㅇ 의료기관, 약국, 노인·장애인 시설 등을 방문하는 경우
- ㅇ 많은 사람을 접촉해야 하는 직업군에 종사하는 사람
  예) 판매원, 요식업 종사자, (창구)상담원 등 고객을 직접 응대해야 하는 직업종사자, 대중교통 운전기사, 역무원, 우체국 집배원, 택배기사, 대형건물 관리원 등
- ㅇ 건강취약계층, 기저질환자 등이 환기가 잘 안 되는 공간에서 2m 이내에 다른 사람과 접촉하는 경우 (예: 군중모임, 대중교통 등)
  * 건강취약계층에 속하는 사람 : 노인, 어린이, 임산부, 만성질환자 등
  * 기저질환을 갖고 있는 사람 : 만성 폐질환, 당뇨, 만성 신질환, 만성 간질환, 만성심혈관질환, 혈액암, 항암치료 암환자, 면역억제제 복용 중인 환자 등
- ㅇ 실내 다중이용시설을 이용하는 경우, 실외에서 2m 거리 유지가 안되는 경우

3 다음의 경우는 마스크 착용을 권하지 않습니다.

- ㅇ 거리를 유지할 수 있는 야외에서나 사람을 만나지 않을 경우 착용을 권고하지 않습니다.
- ㅇ 24개월 미만의 유아, 주변의 도움 없이 스스로 마스크를 제거하기 어려운 사람, 마스크 착용 시 호흡이 어려운 사람은 마스크 착용을 하지 않습니다.

2 환경 소독

< 생활 속 거리 두기 실천지침 : 환경 소독 >

1 환경 소독의 일반 원칙

- ㅇ 적절하고 올바른 방법으로 소독을 하면 효과적이고 안전하게 병원균을 제거할 수 있습니다.
- ㅇ 소독 시에는 창문을 열어 환기를 합니다.
- ㅇ 청소 및 소독 작업을 수행하는 사람은 적절한 개인보호구(일회용 장갑, 마스크, 필요시 일회용 방수용 긴팔 가운 또는 방수 앞치마, 고글 또는 안면보호구)를 착용해야 합니다.
- ㅇ 환경부의 승인 또는 신고된 소독제 중 적절한 제품(소독제 티슈, 알코올(70% 에탄올), 차아염소산나트륨(일명 가정용 락스) 희석액 등)을 사용하며, 소독제를 과도하거나 부적절한 방법으로 사용하면 인체에 해로울 수 있으므로 제조사의 권고사항을 반드시 준수합니다.
  - 차아염소산나트륨(일명 가정용 락스)을 사용하는 경우 소독 직전 희석하여 준비(500~1,000ppm 희석액 등)하고, 희석액을 천에 묻혀 문지르고 10분 이상 그대로 두었다가 깨끗한 물을
적신 천으로 다시 한 번 닦아냅니다.
    * (500ppm 희석액) 빈 생수통 500ml에 차아염소산나트륨(일명 가정용 락스) 원액 5ml를 붓고(예, 생수통 1/2 뚜껑) 냉수를 통 가득 채우고 섞음
    * (1,000ppm 희석액) 빈 생수통 500ml에 차아염소산나트륨(일명 가정용 락스) 원액 10ml를 붓고(예, 생수통 1 뚜껑) 냉수를 통 가득 채우고 섞음
- ㅇ 소독 시에는 소독제가 충분히 묻은 천(또는 소독제 티슈)을 이용합니다.
  - 소독제를 공기 중에 분사하는 방법은 사람에게 흡입되어 위험할 가능성이 있어 주의해야 합니다. 또한, 소독제를 분사하고 닦지 않는 경우에는 분사 표면의 범위가 불분명하고, 소독 효과가 고르지 않을 수 있습니다.
- ㅇ 소독을 마친 후에는 사용한 장갑을 벗고 물과 비누로 반드시 손을 씻어야 합니다.

2 가정, 사무실 등 일상적 공간에서는 다음과 같이 환경소독 합니다.

- ㅇ 소독 시에는 손이 자주 닿는 곳(전화기, 리모콘, 손잡이, 문고리, 탁자, 팔걸이, 스위치, 키보드, 마우스, 복사기 등)을 중심으로 주 1회 이상 소독합니다.
- ㅇ 아이들의 손이 닿는 장난감 등은 소독 후 소독제가 묻어 있는 채로 입과 손에 닿아 위험하지 않도록 깨끗한 천으로 닦아 건조한 후에 사용합니다.

3 공공장소 등 여럿이 오가는 공간에서는 다음과 같이 환경소독 합니다.

- ㅇ 소독 시에는 여러 사람의 손이 자주 닿는 곳(손잡이, 난간, 문고리, 팔걸이, 스위치 등)을 중심으로 합니다.
- ㅇ 건물의 출입문, 승강기 버튼 등 불특정 다수의 접촉이 많은 곳은 매일 1회 이상 청소 및 소독을 합니다.
- ㅇ 시설 관리자는 청소・소독 담당자에게 청소, 소독 및 개인보호 용품 (소독제, 종이 타월 및 마스크 등)을 충분히 제공하도록 합니다.

  ※ 그 외 소독 관련 세부 안내사항은 “「코로나바이러스감염증-19」 대응 집단시설·다중이용시설 소독 안내” 최신지침(2020.4.2.기준 [제3-1판]) 을 참조하세요.

3 65세 이상 어르신 및 고위험군 생활수칙

< 생활 속 거리 두기 실천지침: 어르신 및 고위험군 >

1 어르신 및 고위험군 생활수칙의 일반 원칙

- 어르신 및 고위험군은 면역이 약해 감염이 쉽게 되고 갑자기 중증 또는 위중 상태로 가는 비율이 높기 때문에, 더욱 주의하여야 합니다.
  - 우리나라의 경우 70세 이상 어르신이 코로나19 중증 및 위중환자의 약 60% 이상을 차지하고, 80세 이상 어르신 중에는 10명 중 약 2명이 돌아가시는 상황(치명률: 24.33%) 입니다 (2020.4.30. 0시 기준).
- 가족, 친척 및 간병인 등 호흡기 증상이 있거나 몸이 아픈 사람은 어르신 및 고위험군 방문을 자제합니다.
- 만성질환이 있는 분은 잊지 말고 약을 먹고, 진료일정을 챙깁니다.
  - 평상시 복용해야 하는 약을 중지할 경우 질환이 악화될 수 있으니 주치의와 상의하여 여분의 약을 확보해 두는 것이 좋습니다.
- 65세 이상 어르신은 폐렴구균 등 필요한 예방접종은 반드시 하시기 바랍니다.

2 집에 머무르세요.

- 식료품 구매나 의료기관, 약국 방문 이외에는 가급적 외출을 자제하세요. 그 밖의 꼭 필요하지 않은 외출이나 모임, 여행은 자제합니다.
- 집에 사람들을 초대하거나 다른 사람의 집을 방문하는 것은 자제합니다.
- 건강관리를 위해 가정에서 매일 간단한 운동을 하시고, 술과 담배는 자제합니다.

3 아플 때는 보건소에 연락하고 선별진료소를 방문하세요.

- 38도 이상 고열이 지속되거나 호흡기 증상(기침, 인후통, 콧물 등)이 심해지면 ① 콜센터(☎1339, ☎지역번호+120)나 보건소에 문의하거나 ② 선별진료소를 방문하여 진료를 받습니다.
- 의료기관 방문 시에는 반드시 마스크를 착용하고, 가급적 자기 차로 이동합니다.
  - 주변의 도움 없이 스스로 마스크를 제거하기 어려운 사람, 마스크 착용 시 호흡이 어려운 사람은 마스크 착용을 하지 않습니다.

4 꼭 외출을 해야 하는 경우에는 다음 사항을 실천합니다.

- 사람이 많이 모이는 혼잡한 장소, 특히 밀폐되고 환기가 잘되지 않는 곳은 가지 마세요. 불가피하게 방문을 해야 할 경우 마스크를 쓰고 가십시오.
- 다른 사람과 2m 건강 거리 두기를 합니다. 특히 아픈 사람과 가까이하지 마세요.
- 악수, 포옹 등 신체접촉은 하지 않습니다.
- 다른 사람과 식기를 공유하여 음식을 같이 먹지 말고, 수저 또는 수건 등은 개인 물품을 사용합니다.

5 스트레스로 힘드시다면 다음과 같이 실천합니다.

- 뉴스는 반복해서 보지 말고 시간을 정해놓고 그 시간에만 보는 것이 좋습니다. 너무 자주 들으면 두렵고 우울해집니다.
- 의심스러운 정보를 접했을 때 신뢰할 수 있는지 출처를 확인하고, 부정확한 소문은 공유하지 않으며, 과도한 미디어 몰입을 삼갑니다.
- 몸을 잘 관리합니다.

- 심호흡, 스트레칭, 명상을 하세요.
- 건강하고 균형 잡힌 식사와 규칙적인 운동, 충분히 잠을 자세요.

ㅇ 가족 및 가까운 사람들과 자주 연락합니다.

- 걱정되는 것과 느낌에 대해 신뢰하는 사람들과 통화나 영상으로 이야기하세요.

※ 65세 이상 어르신 및 고위험군

- **만성 기저질환**: 당뇨, 만성 신질환, 만성 간질환, 만성 폐질환, 만성심혈관질환, 혈액암, 항암 치료 암환자, 면역억제제 복용 중인 환자, HIV 환자 등
- **특수상황**: 고도비만, 임신부, 투석환자, 이식환자, 흡연자
- **입원환자**: 실내 공기로 산소포화도 90 미만으로 초기 산소치료 필요 환자

4 건강한 생활습관

< 생활 속 거리 두기 실천지침 : 건강한 생활 습관 >

1 규칙적인 운동으로 건강한 생활습관을 가져봅니다.

- ㅇ 규칙적인 신체활동은 건강증진과 체력향상 외에도 여러 질환의 예방과 관리를 도와줍니다.
  - 깨어있지만 움직이지 않고 앉거나 기대거나 누워서 보내는 여가 시간(컴퓨터, 스마트폰 사용, 텔레비전 시청 등)을 하루 2시간 이내로 줄이는 것이 좋습니다.
- ㅇ 감염병이 유행할 때도 신체활동과 운동은 필요합니다. 실내에서 동영상을 보고 따라 하는 등 혼자 하는 운동을 권장합니다.
- ㅇ 신체 활동 강도를 높이면 침방울이 많이 튈 수 있기 때문에 자주 환기를 합니다.
- ㅇ 어르신이나 질환으로 신체활동을 수행하기 어려울 때는 체력, 신체조건 등 각자의 상황에 맞게 가능한 만큼 신체활동을 하도록 노력합니다.
  - 갑작스런 신체활동 및 운동은 무리하게 장시간 하지 않도록 합니다.

2 정기적으로 건강관리를 실천합니다.

- ㅇ 고혈압, 당뇨, 심뇌혈관 질환 등 지속적인 건강관리와 약물 복용이 필요한 경우에는, 담당 의사와 상담하고 지속적으로 진료받으세요.
- ㅇ 응급상황 등 꼭 필요한 경우에는 반드시 의료기관 방문이 필요합니다.
- ㅇ 적정 시기에 예방접종을 맞고, 정기 검진을 하는 등 주기적인 건강관리를 하면, 질병을 예방할 수 있습니다.

3 균형 있는 영양 섭취를 합니다.

- ㅇ 평소에 바람직한 식생활을 실천하여 영양 상태를 잘 유지하면, 면역력을 증강시켜 질병에 대한 저항력을 키우고 건강을 유지할 수 있습니다.
- ㅇ 다양한 식품을 건강한 조리법으로 골고루 섭취하고, 물을 충분히 마십니다.
- ㅇ 아침밥을 꼭 먹는 것이 좋으며, 덜 짜게, 덜 달게, 덜 기름지게 먹습니다.

4 감염병 스트레스 정신건강 대처법은 다음과 같습니다.

- ㅇ 누구나 감염병에 대한 스트레스를 느낄 수 있습니다. 가족, 친구, 동료와 소통하며 힘든 감정을 나누는 것만으로도 도움이 됩니다.
- ㅇ 믿을만한 정보에 집중하세요. 잘못된 정보는 스트레스를 가중시키고 올바른 판단을 방해합니다.
- ㅇ 힘든 감정은 누구나 경험 할 수 있는 정상적인 반응이지만, 과도한 경우에는 전문가의 도움(일반인 및 격리자 : 정신건강복지센터 ☎ 1577-0199), 확진자 및 가족 : 국가트라우마센터 ☎ 02-2204-0001~2)을 받을 수 있습니다.

3 집단방역 5대 핵심수칙

【핵심 수칙 메시지】

○ **(제 1 수칙)** “공동체가 함께 노력하기”

○ **(제 2 수칙)** “공동체 내 방역관리자 지정하기”

○ **(제 3 수칙)** “공동체 방역지침 만들고 준수하기”

○ **(제 4 수칙)** “방역관리자는 적극적으로 역할 수행”

○ **(제 5 수칙)** “공동체의 책임자와 구성원은 방역관리자에게 적극 협조하기”

< 핵심 수칙 >

제 1 수칙) “우리 공동체를 보호하기 위해 모두가 함께 노력합니다”	
	① 코로나19는 증상이 가벼운 초기에도 전염될 수 있고 전파 속도가 빨라 쉽게 집단 발생이 일어날 수 있습니다. 집단 발생의 예방은 개인뿐 아니라 집단적 노력이 중요합니다.
	② 코로나19의 예방과 조기 차단을 위하여 공동체의 책임자는 구성원의 동의와 협력을 기반으로 효과적인 대응체계를 미리 만들어 두어야 합니다.
	③ 이러한 노력은 회사와 같은 공적 공동체뿐만 아니라 종교모임, 취미모임 등 사적 공동체에도 꼭 필요합니다.
제 2 수칙) “공동체 내에서 방역관리자를 지정합니다”	
	① 구성원의 동의와 협력을 기반으로 공동체의 방역을 관리할 책임자를 방역관리자로 지정합니다.
	② 방역관리자는 공동체 규모에 따라 개인이 될 수도 있고, 적정 인원으로 구성된 팀이 될 수도 있습니다.
	③ 방역관리자는 코로나19 전파로부터 공동체를 보호하는 역할을 하며, 주요한 활동 과정에서 방역관리의 책임을 맡습니다.
	④ 공동체의 구성원은 공동체의 방역 관리를 위한 방역관리자의 요청에 적극적으로 협력합니다.

제 3 수칙) “방역관리자는 방역지침을 만들고 모두가 준수하도록 합니다”	
	① 방역관리자는 우리 공동체의 밀폐도, 밀집도, 그 밖의 특성 등을 고려하여 위험도를 평가하고 이를 고려해 방역지침을 만듭니다.
	② 방역지침은「집단방역 보조수칙(세부지침)」중 적합한 지침을 참고하되, 아플 때 집에서 3~4일 쉬기, 손 씻기, 소독과 환기, 사람 간의 거리 두기, 마음은 가까이 등 개인방역 5대 수칙과 고위험군, 환경소독, 마스크, 건강생활 보조수칙도 포함하여 작성합니다.
	③ 공동체의 특성상 많은 사람을 만나거나 밀폐된 환경인 경우 자주 손 씻기, 1~2m 거리 두기, 마스크 착용 또는 안면보호대 설치, 유증상자 확인, 자주 환기 등을 강화하여 방역지침을 작성합니다.
	④ 방역관리자는 공동체의 구성원을 주기적으로 교육하고 각자가 이를 실천하도록 도와 방역지침을 실천할 수 있도록 합니다.
	⑤ 방역관리자가 없는 가운데 집단의 활동이 이루어질 경우 다른 구성원이 방역관리자의 역할을 대신하여 방역지침을 실천하도록 돕습니다.
	⑥ 방역관리자는 방역지침을 실천할 수 있도록 구성원의 역할 배정과 시설 환경을 점검하고 문제가 있을 경우 공동체에 개선을 요구합니다.
제 4 수칙) “방역관리자는 공동체 보호를 위해 적극적 역할을 수행합니다”	
	① 방역관리자는 지역의 보건소 담당자의 연락망을 확보하고 방역활동에 필요한 협력관계를 만들어 두어야 합니다.
	② 방역관리자는 구성원들의 호흡기 증상 유무를 체크하고 체온을 측정하는 등의 활동을 매일 또는 매번 해야 합니다. 이 경우 가급적 구성원들의 일자별 증상 여부를 기록해 보관하도록 합니다.
	③ 호흡기 증상 또는 발열이 확인되는 구성원에 대해서는 즉각적으로 집으로 보내 3~4일 쉬도록 합니다. 고위험군 또는 65세 이상 어르신의 경우 코로나19 검사를 받도록 선별진료소로 안내합니다.
	④ 공동체 내 밀접 접촉이 일어나는 동일 부서, 동일 학급, 동일 장소 등에 2~3명 이상의 유증상자가 3~4일 내에 발생 시 유증상자가 코로나19 검사를 받도록 안내하며, 유증상자가 추가 발생 시 보건소에 집단감염 가능성을 신고합니다.

제5수칙) "공동체의 책임자와 구성원은 방역관리자를 적극적으로 돕고 따릅니다"	
	① 공동체의 책임자는 방역관리자의 활동을 지지하고, 방역지침의 준수에 필요한 역할 변경, 환경 개선 등의 요청에 적극적으로 응해야 합니다.
	② 공동체의 책임자와 각 구성원들은 우리 모두의 안전을 위해 방역지침을 준수하고, 방역관리자의 요청에 적극적으로 협력합니다.
	③ 공동체의 책임자는 매월 1회 이상 공동체의 방역관리 노력을 평가하고 개선이 필요한 사항을 점검하는 평가 회의를 운영합니다.

세부지침

Ⅰ. 업 무 (1. 일할 때)

1-1. 생활 속 거리 두기 지침 : 사업장

1 근로자

[공통사항]

- ○ 발열 또는 호흡기 증상(기침, 인후통 등)이 있거나 최근 14일 이내 해외여행을 한 경우 출근을 자제하기
- ○ 다른 사람과 2m(최소 1m) 이상 거리 두기
- ○ 흐르는 물과 비누로 30초 이상 손을 씻거나 손 소독제로 손 소독하기
- ○ 기침이나 재채기를 할 때는 휴지, 옷소매로 입과 코 가리기
- ○ 침방울이 튀는 행위(노래부르기, 구호외치기 등)나 신체접촉(악수, 포옹 등) 자제하기
- ○ 실내 다중이용시설을 이용하는 경우 마스크 착용하기
- ○ 실외에서 2m 거리 유지가 안되는 경우 마스크 착용하기

[해당 유형 적용사항]

- ○ 근무 중 발열, 기침 등이 나타나면 사업주에게 알린 후 마스크 착용하고 퇴근하기
- ○ 유연근무제(재택근무, 시차출퇴근 등), 휴가제도(가족돌봄휴가, 연차휴가, 병가 등) 적극 활용하기
- ○ 국내·외 출장은 가급적 줄이기
- ○ 워크숍, 교육, 연수 등은 가급적 온라인 또는 영상을 이용하고, 대면하는 경우 마스크 착용, 손 소독제 사용 등 개인위생수칙 준수하기
- ○ 개인 찻잔·찻숟가락 등 개인물품 사용하기
- ○ 손이 자주 닿는 곳(탁자, 키보드, 마우스, 전화기 등) 주기적으로 소독하기
- ○ 사무실, 작업장 등을 환기하기
- ○ 소규모 모임, 동아리 활동, 회식 등은 자제하고, 퇴근 후 일찍 귀가하기
- ○ 구내식당 이용 시 가급적 일렬 또는 지그재그로 앉고 대화 자제하기
- ○ 엘리베이터 등 밀폐된 공간에서는 마스크를 착용하고 대화 자제하기
- ○ 휴게실 등은 여러 명이 함께 이용하지 않기
- ○ 매일 비접촉식 체온계나 열화상카메라 등으로 증상 여부(발열, 호흡기 증상 등) 확인에 협조하기

2 사업주

[공통사항]

○ 방역을 관리하는 담당부서(관리자)를 지정하고 지역 보건소 담당자의 연락망을 확보하는 등 방역 협력체계 구축하기

○ 공동체 내 밀접 접촉이 일어나는 동일 부서, 동일 장소 등에 2~3명 이상의 유증상자가 3~4일 내에 발생 시 유증상자가 코로나19 검사를 받도록 안내하며, 유증상자가 추가 발생 시 보건소에 집단감염 가능성을 신고하기

○ 종사자가 발열 또는 호흡기 증상이 있는 경우 출근 중단 및 즉시 퇴근 조치하기

○ 사람 간 간격을 2m(최소 1m) 이상 거리 두기

○ 손을 씻을 수 있는 시설 또는 손 소독제를 비치하고, 손 씻기 및 기침예절 준수 안내문 게시하기

○ 자연 환기가 가능한 경우 창문을 상시 열어두고, 에어컨 사용 등으로 상시적으로 창문을 열어두기 어려운 경우 2시간마다 1회 이상 환기하기

○ 공용으로 사용하는 물건(출입구 손잡이 등) 및 표면은 매일 1회 이상 자주 소독하기

○ 고객(이용자)을 직접 응대하는 경우 마스크 착용하게 하기

○ 발열 또는 호흡기 증상이 있거나 최근 14일 이내 해외여행을 한 경우 방문 자제 안내하기

○ 실내 다중이용시설을 이용하는 경우 마스크 착용 안내하기

○ 실외에서 2m 거리 유지가 안되는 경우 마스크 착용 안내하기

[해당 유형 적용사항]

○ 방역관리자는 근로자 밀집도, 환기상태, 업무방식 등을 고려하여 방역지침 만들기

○ 발열이나 호흡기증상이 있거나, 최근 14일 이내 해외여행이나 해외출장을 다녀온 사람은 재택근무, 병가·연차휴가·휴직 등을 사용하게 하기

* 필요시 취업규칙 등에 반영

○ 매일 비접촉식 체온계나 열화상카메라 등으로 근로자의 증상 여부(발열, 호흡기 증상 등) 확인하기

○ 유연근무제 및 휴가를 자유롭게 활용할 수 있는 분위기를 조성하기

○ 국내·외 출장은 가급적 줄이기

○ 워크숍, 교육, 연수 등은 온라인 또는 영상 활용하되, 대면방식으로 실시하는 경우 체온측정, 마스크 착용, 소독용품 비치하기

○ 모니터·책상·작업대 위치 및 방향을 조정하거나 유휴공간을 활용하여 근로자 간 간격을 2m(최소 1m) 이상 유지하기

○ 침방울이 튀는 행위(노래부르기, 구호외치기 등) 유도하지 않기

○ 구내식당 좌석 간 투명 가림막을 설치하거나 가급적 일렬 또는 지그재그로 앉게 하기

○ 개인용 청소·소독용품을 지급 또는 비치하기

○ 마스크 및 위생물품을 사업장 상황에 맞게 지급·비치하거나 구입 지원하기

○ 손 씻기, 손 소독제 사용, 기침예절 등 위생관리방안을 게시 또는 교육하기

○ 휴게실 등은 여러 명이 함께 이용하지 않도록 안내하기

○ 외부인을 응대할 수 있는 간이 회의실 등을 사업장 상황에 맞게 마련하기

1-2. 생활 속 거리 두기 지침 : 회의

1 일반 수칙

○ 가급적 영상회의, 전화회의 등을 활용하기

○ 영상회의, 전화회의 등이 가능하도록 업무 환경을 개선하기

○ 대면회의 때는 환기가 용이하고 간격을 넓게 둘 수 있는 큰 공간을 회의 장소로 확보하기

○ 참석 인원을 최소화하고 효율적으로 진행하여 회의 시간을 단축하기

2 대면회의를 할 때에는 다음의 사항을 준수

○ 사전에 발열, 호흡기 증상(인후통(목아픔), 기침, 호흡곤란, 권태감, 두통, 근육통 등) 등이 있거나 14일 이내 해외 여행력이 있으면 회의에 참여하지 말 것을 공지하기

○ 개최자 또는 사회자는 회의 시작 전에 발열, 호흡기 증상 등을 확인하고 유증상자는 회의 참석 자제하기

○ 회의 전후에 악수 등 신체적 접촉 자제하기

○ 회의실 곳곳에 손 소독제를 비치하여, 참석자는 수시로 사용하기

○ 회의 장소는 환기가 용이한 넓은 장소를 활용하고, 회의 시작 전에 환기하기

○ 1시간이 지난 때에는 휴식 시간을 가지며 회의 장소의 문과 창문을 열고 환기하기

○ 회의 참석자 간의 간격은 2m, 공간이 좁더라도 최소 1m 이상은 유지하기

○ 2m(최소 1m) 이상 거리 유지와 1시간 간격 환기를 준수하지 못하는 경우 대면회의는 자제하기

- 불가피하게 대면회의를 하는 경우에는 참석자 전원 마스크를 쓰고 발언 시에도 마스크를 계속 착용하기

* 간격 유지 및 환기 등을 준수하는 경우 마스크는 개인 선택에 따라 착용

1-3. 생활 속 거리 두기 지침 : 민원창구

1 이용자

[공통사항]

○ 발열 또는 호흡기 증상(기침, 인후통 등)이 있거나 최근 14일 이내 해외여행을 한 경우 방문 자제하기

○ 다른 사람과 2m(최소 1m) 이상 거리 두기

○ 흐르는 물과 비누로 30초 이상 손을 씻거나 손 소독제로 손 소독하기

○ 기침이나 재채기를 할 때는 휴지, 옷소매로 입과 코 가리기

○ 침방울이 튀는 행위(노래부르기, 소리지르기 등)나 신체접촉(악수, 포옹 등) 자제하기

○ 실내 다중이용시설을 이용하는 경우 마스크 착용하기

○ 실외에서 2m 거리 유지가 안되는 경우 마스크 착용하기

2 책임자·종사자

[공통사항]

○ 방역관리자 지정 및 지역 보건소 담당자의 연락망을 확보하는 등 방역 협력체계 구축하기

○ 공동체 내 밀접 접촉이 일어나는 동일 부서, 동일 장소 등에 2~3명 이상의 유증상자가 3~4일 내에 발생 시 유증상자가 코로나19 검사를 받도록 안내하며, 유증상자가 추가 발생 시 보건소에 집단감염 가능성을 신고하기

○ 종사자가 발열 또는 호흡기 증상이 있는 경우 출근 중단 및 즉시 퇴근 조치하기

○ 민원인 또는 직원 간 간격을 2m(최소 1m) 이상 거리 두기

○ 손을 씻을 수 있는 시설 또는 손 소독제를 비치하고, 손 씻기 및 기침예절 준수 안내문 게시하기

○ 자연 환기가 가능한 경우 창문을 상시 열어두고, 에어컨 사용 등으로 상시적으로 창문을 열어두기 어려운 경우 2시간마다 1회 이상 환기하기

○ 공용으로 사용하는 물건(출입구 손잡이 등) 및 표면은 매일 1회 이상 자주 소독하기

○ 고객(이용자)을 직접 응대하는 경우 마스크 착용하기

○ 발열 또는 호흡기 증상이 있거나 최근 14일 이내 해외여행을 한 경우 방문 자제 안내하기

○ 실내 다중이용시설을 이용하는 경우 마스크 착용 안내하기

○ 실외에서 2m 거리 유지가 안되는 경우 마스크 착용 안내하기

[해당 유형 적용사항]

1. 근무자

○ 근무자에게 발열 및 기침 등 의심증상이 있거나 최근 14일 이내 해외여행을 한 경우 부서장은 해당 공무원 등에 대해 출근하지 않도록 조치할 수 있고, 이 경우 ‘재택근무’ 또는 ‘공가’ 처리하기

* 해당 공무원은 3~4일간 경과를 지켜본 후 이상이 없을 경우 출근

○ 근무 중 2회 이상 체온 등 체크 ⇒ 이상 증상자는 보건당국 상담 후 조치에 따름

○ 확진자 발생 시 출입자 동선 파악 등에 대비하여 이상 징후자 파악 보고 체계 확립 및 복무상황에 대한 철저한 기록 유지하기

○ 직원은 유연근무 및 휴가를 가급적 자유롭게 활용할 수 있도록 하고, 대체 인력 확보하기

2. 민원창구

○ 민원담당 부서장을 방역관리책임자로 지정하여 방역소독 철저히 하기

- 손 소독제 등 방역 물품 비치(민원접수대, 무인민원발급기, 공용공간, 화장실 등), 손이 닿는 출입문 등 수시 소독하기
- 민원실·공용공간 등 주 2회 이상 소독, 바닥 청소 시 소독제 사용하기

  * 소독제 및 소독방법은「코로나바이러스감염증-19 대응 집단시설·다중이용시설 소독 안내」참조

- 자체 실정에 맞는 방역대책 마련·시행: 민원창구 투명 가림막 설치, 마스크 미착용 방문객 1회용 마스크 제공, 중앙현관을 제외한 출입문 폐쇄 등
- 열화상카메라 설치 등 발열 체크, 발열자 대기실 마련(관리대장 작성 및 관리)하기
- 민원창구 투명 가림막 설치, 자동문을 개방하거나 출입문 일원화 등 자체 실정에 맞는 방역대책 시행하기

○ 유관기관 협력체계 유지

- 유관기관(보건소, 경찰서, 소방서, 의료기관 등) 비상연락체계 유지 및 상황 발생 시 즉시 대응하기
- 민원실 내 유증상자 발생시 즉시 관할 보건소 신고 및 격리 공간 대기하기
- 민원실 내 확진자 발생시 즉시 관할 보건소에 신고하고, 시설 일시폐쇄, 출입금지, 격리, 소독 등 필요한 방역조치를 시행하기

 * 구체적인 사항은「코로나19 확진환자 발생 시 시설 방역관리 등 조치사항 안내」(중대본-125, 3.6.) 참고

○ 민원서류발급은 "정부24·무인민원발급기·전자증명서" 이용 안내 활성화하여 가급적 대면 민원처리를 최소화하기

3. 민원창구 근무자 및 방문객 대상 위생수칙 교육·홍보

○ 민원창구 직원 대상으로 코로나19 감염증 예방수칙, 손 씻기, 기침예절 등 감염병 예방 교육 실시하기

○ 손 씻기, 기침 예절 등 코로나19 감염증 예방을 위한 위생수칙 등 각종 홍보물*을 시설 내 주요 장소에 부착하기

 * 관련 홍보물은 질병관리본부 홈페이지(www.kcdc.go.kr)에 게시된 자료 활용

 * 방역 및 소독관련 언급되지 않은 사항은 「코로나바이러스감염증-19 집단시설·다중이용시설 대응지침(제3판)」(방대본·중수본,3.25), 「코로나바이러스감염증-19 대응 집단시설·다중이용시설 소독 안내(제3-1판)」 (방대본·중수본,4.2.) 참조

1-4. 생활 속 거리 두기 지침 : 우체국

1 이용자·방문객

[공통사항]

○ 발열 또는 호흡기 증상(기침, 인후통 등)이 있거나 최근 14일 이내 해외여행을 한 경우 방문 자제하기

○ 다른 사람과 2m(최소 1m) 이상 거리 두기

○ 흐르는 물과 비누로 30초 이상 손을 씻거나 손 소독제로 손 소독하기

○ 기침이나 재채기를 할 때는 휴지, 옷소매로 입과 코 가리기

○ 침방울이 튀는 행위(노래부르기, 소리지르기 등)나 신체접촉(악수, 포옹 등) 자제하기

○ 실내 다중이용시설을 이용하는 경우 마스크 착용하기

○ 실외에서 2m 거리 유지가 안되는 경우 마스크 착용하기

[해당 유형 적용사항]

○ 가능한 한 금융업무는 스마트 뱅킹, 인터넷 뱅킹을 이용하고, 우체국 방문하여 금융 업무 시 ATM 기기 활용하기

○ 우편 업무로 방문 시 무인우편접수기 이용하기(설치우체국 한함)

2 책임자·종사자

[공통사항]

○ 방역관리자 지정 및 지역 보건소 담당자의 연락망을 확보하는 등 방역 협력체계 구축

○ 공동체 내 밀접 접촉이 일어나는 동일 부서, 동일 장소 등에 2~3명 이상의 유증상자가 3~4일 내에 발생 시 유증상자가 코로나19 검사를 받도록 안내하며, 유증상자가 추가 발생 시 보건소에 집단감염 가능성을 신고하기

○ 종사자가 발열 또는 호흡기 증상이 있는 경우 출근 중단 및 즉시 퇴근 조치하기

○ 사람 간 간격을 2m(최소 1m) 이상 거리 두기

○ 손을 씻을 수 있는 시설 또는 손 소독제를 비치하고, 손 씻기 및 기침예절 준수 안내문 게시하기

○ 자연 환기가 가능한 경우 창문을 상시 열어두고, 에어컨 사용 등으로 상시적으로 창문을 열어두기 어려운 경우 2시간마다 1회 이상 환기하기

○ 공용으로 사용하는 물건(출입구 손잡이 등) 및 표면은 매일 1회 이상 자주 소독하기

○ 고객(이용자)을 직접 응대하는 경우 마스크 착용하기

○ 발열 또는 호흡기 증상이 있거나 최근 14일 이내 해외여행을 한 경우 방문 자제 안내하기

○ 실내 다중이용시설을 이용하는 경우 마스크 착용 안내하기

○ 실외에서 2m 거리 유지가 안되는 경우 마스크 착용 안내하기

[해당 유형 적용사항]

○ 식사 시간에 다른 사람과 간격 유지(한 방향 보기, 띄워 앉기, 지그재그 앉기 등 가능하도록 스티커 등 표시)하기

○ 타 부서 방문 및 층간 이동 자제, 꼭 필요한 경우가 아니면 출장 지양, 대면 대화 자제하고 내선전화 또는 내부전용 메신저 이용하여 의사소통, 비대면 결재 활성화하기

○ 대규모 행사, 공동 활동 등 최소화, 교육 및 회의 계획 시 온라인 교육·회의 활성화하기

○ 출입구 및 시설 내 곳곳에 휴지 및 뚜껑 있는 쓰레기통 비치하기

○ 개인위생수칙 준수, 생활 속 거리 두기의 필요성 등에 대해 주기적으로 안내 및 협조 요청하기

○ 스마트 뱅킹, 인터넷 뱅킹, ATM기기, 무인우편접수기 이용 안내하기

1-5. 생활 속 거리 두기 지침 : 국내출장

1 일반 수칙

○ 출장은 가급적 줄이기

○ 코로나19 지역사회 감염이 확산된 곳으로 출장 자제 또는 연기

○ 출장 인원, 소요 시간, 경로를 최소화하기

○ 출장자는 출장 전 스스로 체온 확인하고, 발열(37.5℃) 및 호흡기 증상(기침, 인후통 등)이 있는 경우 출장 연기하기

○ 출장자는 손 씻기, 마스크 착용 등 개인위생수칙 준수하기

○ 출장 중 실내 다중이용시설을 이용하는 경우 마스크 착용하기

○ 출장 중 실외에서 2m 거리 유지가 안 되는 경우 마스크 착용하기

2 국내 출장 중 준수 사항

○ 공용·개인 차량 이동 시 동승자가 있는 경우 탑승자 모두 마스크 착용하기

○ 버스, 지하철, 택시 등 대중교통 이용 시 마스크 착용하고 대화 자제하기

○ 출장지에서 다른 사람과 만나는 경우 신체 접촉 자제 및 2m(최소 1m) 이상 거리 두기

○ 가급적 환기가 원활하고 사람이 밀집되지 않은 장소에서 업무 보기

○ 출장 중에 발열, 호흡기 증상(기침, 인후통 등) 등이 있는 아픈 사람과 가급적 접촉하지 않기

○ 음식점·카페 등 이용 시 머무르는 시간 최소화, 대화 자제, 음식은 개인 접시에 덜어 먹기

○ 출장 업무 외 소규모 모임, 회식 등을 가급적 자제하고 일찍 귀가하기

○ 출장 업무 외 다수가 밀집된 공간 및 다중이용시설 등의 방문을 자제하기

○ 출장지에서 숙박을 하는 경우 불가피한 경우를 제외하고 동료와 방을 같이 쓰지 않기

○ 출장 중에 발열 또는 호흡기 증상이 있을 경우 출장을 중단하고 퇴근하기

○ 출장 후 발열 및 호흡기 증상이 나타난 경우 콜센터(☎ 1339, ☎ 지역번호+120)나 관할 보건소에 상담하기

※ 출장 중 회의 시 해당 유형의 지침을 준용

1-6. 생활 속 거리 두기 지침 : 방문서비스

① 근로자

[공통사항]

○ 발열 또는 호흡기 증상(기침, 인후통 등)이 있거나 최근 14일 이내 해외여행을 한 경우 출근을 자제하기

○ 다른 사람과 2m(최소 1m) 이상 거리 두기

○ 흐르는 물과 비누로 30초 이상 손을 씻거나 손 소독제로 손 소독하기

○ 기침이나 재채기를 할 때는 휴지, 옷소매로 입과 코 가리기

○ 침방울이 튀는 행위(노래부르기, 구호외치기 등)나 신체접촉(악수, 포옹 등) 자제하기

○ 실내 다중이용시설을 이용하는 경우 마스크 착용하기

○ 실외에서 2m 거리 유지가 안되는 경우 마스크 착용하기

[해당 유형 적용사항]

○ 사무실 출근 시 비접촉식 체온계나 열화상카메라 등으로 증상 여부(발열, 호흡기 증상 등) 확인에 협조하기, 사무실에 출근하지 않는 경우는 스스로 체온 확인하기

○ 근무 중 발열, 기침 등이 나타나면 사업주에게 알린 후 마스크 착용하고 퇴근하기

○ 휴가제도(가족돌봄휴가, 연차휴가, 병가 등) 적극 활용하기

○ 워크숍, 교육, 연수 등은 가급적 온라인 또는 영상을 이용하고, 대면하는 경우 마스크 착용, 손 소독제 사용 등 개인위생수칙 준수하기

○ 고객과 불필요한 대면을 최소화하고, 대면하는 경우 마스크 착용하기

○ 고객이 자가격리 중이거나 발열 또는 호흡기 증상이 있을 경우 방문 연기

○ 공용·개인 차량 이용 시 동승자가 있는 경우 탑승자 모두 마스크 착용하기

○ 공용·개인 차량의 내부(운전대, 기어손잡이 등), 업무용품(무선카드단말기, 펜 등)을 주기적으로 청소 및 소독하기

○ 버스, 지하철, 택시 등 대중교통 이용 시 마스크 착용하고 대화 자제하기

○ 현장 결제 시 가능한 전자 결제방식(모바일페이, QR코드, NFC카드, 신용카드 등)을 활용하기

○ 음식점·카페 등 이용 시 머무르는 시간 최소화, 대화 자제, 음식은 개인 접시에 덜어 먹기

○ 소규모 모임, 동아리 활동, 회식 등은 자제하고, 퇴근 후 일찍 귀가하기

2 이용자(고객)

[공통사항]

- ○ 발열 또는 호흡기 증상(기침, 인후통 등)이 있거나 최근 14일 이내 해외여행을 한 경우 출근을 자제하기
- ○ 다른 사람과 2m(최소 1m) 이상 거리 두기
- ○ 흐르는 물과 비누로 30초 이상 손을 씻거나 손 소독제로 손 소독하기
- ○ 기침이나 재채기를 할 때는 휴지, 옷소매로 입과 코 가리기
- ○ 침방울이 튀는 행위(노래부르기, 구호외치기 등)나 신체접촉(악수, 포옹 등) 자제하기
- ○ 실내 다중이용시설을 이용하는 경우 마스크 착용하기
- ○ 실외에서 2m 거리 유지가 안되는 경우 마스크 착용하기

[해당 유형 적용사항]

- ○ 방문근로자와 불필요한 대면을 최소화하고, 대면하는 경우 마스크 착용하기
- ○ 자가격리 중이거나 발열 또는 호흡기 증상이 있을 시 방문근로자 방문 일정 연기하기
- ○ 온라인 사전 결제 등을 적극 활용하되, 현장 결제 시 가능한 전자 결제방식(모바일 페이, QR코드, NFC카드, 신용카드 등)을 활용하기

3 사업주

[공통사항]

- ○ 방역을 관리하는 담당부서(관리자)를 지정하고 지역 보건소 담당자의 연락망을 확보하는 등 방역 협력체계 구축하기
- ○ 공동체 내 밀접 접촉이 일어나는 동일 부서, 동일 장소 등에 2~3명 이상의 유증상자가 3~4일 내에 발생 시 유증상자가 코로나19 검사를 받도록 안내하며, 유증상자가 추가 발생 시 보건소에 집단감염 가능성을 신고하기
- ○ 종사자가 발열 또는 호흡기 증상이 있는 경우 출근 중단 및 즉시 퇴근 조치하기
- ○ 사람 간 간격을 2m(최소 1m) 이상 거리 두기
- ○ 손을 씻을 수 있는 시설 또는 손 소독제를 비치하고, 손 씻기 및 기침예절 준수 안내문 게시하기
- ○ 자연 환기가 가능한 경우 창문을 상시 열어두고, 에어컨 사용 등으로 상시적으로

창문을 열어두기 어려운 경우 2시간마다 1회 이상 환기하기

○ 공용으로 사용하는 물건(출입구 손잡이 등) 및 표면은 매일 1회 이상 자주 소독하기

○ 고객(이용자)을 직접 응대하는 경우 마스크 착용하게 하기

○ 발열 또는 호흡기 증상이 있거나 최근 14일 이내 해외여행을 한 경우 방문 자제 안내하기

○ 실내 다중이용시설을 이용하는 경우 마스크 착용 안내하기

○ 실외에서 2m 거리 유지가 안되는 경우 마스크 착용 안내하기

[해당 유형 적용사항]

○ 방역관리자는 근로자 밀집도, 환기상태, 업무방식 등을 고려하여 방역지침 만들기

○ 발열, 호흡기증상이 있거나, 최근 14일 이내 해외여행이나 해외출장을 다녀온 사람은 병가·연차휴가·휴직 등을 사용하게 하기 * 필요시 취업규칙 등에 반영

○ 사무실 출근 시 비접촉식 체온계나 열화상카메라 등으로 근로자 체온 검사하고, 호흡기 증상 여부 확인하기

○ 유연근무제 및 휴가를 자유롭게 활용할 수 있는 분위기를 조성하기

○ 워크숍, 교육, 연수 등은 온라인 또는 영상 활용하되, 대면방식으로 실시하는 경우 체온측정, 마스크 착용, 소독용품 비치하기

○ 침방울이 튀는 행위(구호외치기 등) 유도하지 않기

○ 가급적 비대면으로 서비스 제공하기

○ 버스, 지하철, 택시 등 대중교통 이용 시 마스크 착용하고 대화 자제 안내하기

○ 공용차량을 제공하는 경우 차량 내부를 주기적으로 소독하기

○ 온라인 사전 결제 등을 활용하도록 하되, 현장 결제시 전자 결제방식(모바일페이, QR코드, NFC카드, 신용카드 등)을 활용하도록 하기

○ 음식점·카페 등 이용 시 머무르는 시간 최소화, 대화 자제, 음식은 개인 접시에 덜어 먹도록 안내하기

○ 마스크, 휴대용 손 소독제 등 위생물품을 사업장 상황에 맞게 지급하거나 구입할 수 있게 지원하기

○ 손 씻기, 손 소독제 사용, 기침예절 등 위생관리방안을 게시 또는 교육하기

1-7. 생활 속 거리 두기 지침 : 콜센터

1 근로자

[공통사항]

- ○ 발열 또는 호흡기 증상(기침, 인후통 등)이 있거나 최근 14일 이내 해외여행을 한 경우 출근을 자제하기
- ○ 다른 사람과 2m(최소 1m) 이상 거리 두기
- ○ 흐르는 물과 비누로 30초 이상 손을 씻거나 손 소독제로 손 소독하기
- ○ 기침이나 재채기를 할 때는 휴지, 옷소매로 입과 코 가리기
- ○ 침방울이 튀는 행위(노래부르기, 구호외치기 등)나 신체접촉(악수, 포옹 등) 자제하기
- ○ 실내 다중이용시설을 이용하는 경우 마스크 착용하기
- ○ 실외에서 2m 거리 유지가 안되는 경우 마스크 착용하기

[해당 유형 적용사항]

- ○ 매일 비접촉식 체온계나 열화상카메라 등으로 증상 여부(발열, 호흡기 증상 등) 확인에 협조하기
- ○ 근무 중 발열, 기침 등이 나타나면 사업주에게 알린 후 마스크 착용하고 퇴근하기
- ○ 휴가제도(가족돌봄휴가, 연차휴가, 병가 등) 적극 활용하기
- ○ 재택근무, 시차출퇴근제 등을 적극 활용하여 동시 근무 인원 최소화하기
- ○ 국내·외 출장은 가급적 줄이기
- ○ 워크숍, 교육, 연수 등은 가급적 온라인 또는 영상을 이용하고, 대면하는 경우 마스크 착용, 손 소독제 사용 등 개인위생수칙 준수하기
- ○ 가급적 마스크 착용하고 근무하기
  - 사무실 내 환기, 근로자 간 간격 및 등 설치를 준수한 경우 마스크는 개인 선택에 따라 착용
- ○ 개인 찻잔·찻숟가락 등 개인물품 사용하기
- ○ 자주 사용하는 사무기기(전화기, 헤드셋, 마이크)에 1회용 덮개 사용 또는 주기적 소독
- ○ 사무실, 작업장 등을 환기하기

○ 소규모 모임, 동아리 활동, 회식 등은 자제하고, 퇴근 후 일찍 귀가하기
○ 구내식당 이용 시 가급적 일렬 또는 지그재그로 앉고 대화는 자제하기
○ 엘리베이터 등 밀폐된 공간에서는 마스크를 착용하고 대화를 자제하기
○ 휴게실 등은 여러 명이 함께 이용하지 않기

2 사업주

[공통사항]

○ 방역을 관리하는 담당부서(관리자)를 지정하고 지역 보건소 담당자의 연락망을 확보하는 등 방역 협력체계 구축하기
○ 공동체 내 밀접 접촉이 일어나는 동일 부서, 동일 장소 등에 2~3명 이상의 유증상자가 3~4일 내에 발생 시 유증상자가 코로나19 검사를 받도록 안내하며, 유증상자가 추가 발생 시 보건소에 집단감염 가능성을 신고하기
○ 종사자가 발열 또는 호흡기 증상이 있는 경우 출근 중단 및 즉시 퇴근 조치하기
○ 사람 간 간격을 2m(최소 1m) 이상 거리 두기
○ 손을 씻을 수 있는 시설 또는 손 소독제를 비치하고, 손 씻기 및 기침예절 준수 안내문 게시하기
○ 자연 환기가 가능한 경우 창문을 상시 열어두고, 에어컨 사용 등으로 상시적으로 창문을 열어두기 어려운 경우 2시간마다 1회 이상 환기하기
○ 공용으로 사용하는 물건(출입구 손잡이 등) 및 표면은 매일 1회 이상 자주 소독하기
○ 고객(이용자)을 직접 응대하는 경우 마스크 착용하게 하기
○ 발열 또는 호흡기 증상이 있거나 최근 14일 이내 해외여행을 한 경우 방문 자제 안내하기
○ 실내 다중이용시설을 이용하는 경우 마스크 착용 안내하기
○ 실외에서 2m 거리 유지가 안되는 경우 마스크 착용 안내하기

[해당 유형 적용사항]

○ 방역관리자는 근로자 밀집도, 환기상태, 업무방식 등을 고려하여 방역지침 만들기
○ 발열이나 호흡기증상이 있거나, 최근 14일 이내 해외여행이나 해외출장을 다녀온 사람은 재택근무, 병가·연차휴가·휴직 등을 사용하게 하기
  * 필요시 취업규칙 등에 반영

○ 매일 비접촉식 체온계나 열화상카메라 등으로 근로자 증상 여부(발열, 호흡기 증상 등) 확인하기

○ 유연근무제 및 휴가를 자유롭게 활용할 수 있는 분위기를 조성하기

○ 재택근무가 가능하도록 시스템 구축하기

○ 국내·외 출장은 가급적 줄이기

○ 워크숍, 교육, 연수 등은 온라인 또는 영상 활용하되, 대면방식으로 실시하는 경우 체온측정, 마스크 착용, 소독용품 비치하기

○ 모니터·책상 위치 및 방향을 조정하거나 유휴공간을 활용하여 근로자 간 간격을 2m(최소 1m) 이상 유지하기

○ 근로자 간 투명 칸막이 또는 가림막 등 설치(권장 높이: 책상 면에서 90cm)하기

○ 근로자가 고정좌석에서 근무하게 하기

○ 채팅이나 챗봇 등 비음성 상담 방식을 활용하기

○ 상담건수, 응답률 등을 이유로 휴가 사용을 제한하거나 불이익 주지 않기

○ 침방울이 튀는 행위(구호외치기 등) 유도하지 않기

○ 부서 또는 층별로 점심시간 시차운영 활용하기

(예: A부/11:30~12:30, B부/12:30~13:30)

○ 구내식당 좌석 간 투명 가림막을 설치하거나 가급적 일렬 또는 지그재그로 앉게 하기

○ 충분한 휴식 시간 부여하기

○ 개인용 청소·소독용품을 지급 또는 비치하기

○ 마이크 사용 시, 덮개 사용하거나 개인별로 사용하도록 하기

○ 마스크 및 위생물품을 사업장 상황에 맞게 지급·비치하거나 구입 지원하기

○ 손 씻기, 손 소독제 사용, 기침예절 등 위생관리방안을 게시 또는 교육하기

○ 휴게실 등은 여러 명이 함께 이용하지 않도록 안내하기

○ 외부인을 응대할 수 있는 간이 회의실 등을 사업장 상황에 맞게 마련하기

1-8. 생활 속 거리 두기 지침 : 건설업

1 근로자

[공통사항]

○ 발열 또는 호흡기 증상(기침, 인후통 등)이 있거나 최근 14일 이내 해외여행을 한 경우 출근을 자제하기

○ 다른 사람과 2m(최소 1m) 이상 거리 두기

○ 흐르는 물과 비누로 30초 이상 손을 씻거나 손 소독제로 손 소독하기

○ 기침이나 재채기를 할 때는 휴지, 옷소매로 입과 코 가리기

○ 침방울이 튀는 행위(노래부르기, 구호외치기 등)나 신체접촉(악수, 포옹 등) 자제하기

○ 실내 다중이용시설을 이용하는 경우 마스크 착용하기

○ 실외에서 2m 거리 유지가 안되는 경우 마스크 착용하기

[해당 유형 적용사항]

○ 매일 비접촉식 체온계나 열화상카메라 등으로 증상 여부(발열, 호흡기 증상 등) 확인에 협조하기

○ 근무 중 발열, 기침 등이 나타나면 사업주에게 알린 후 마스크 착용하고 퇴근하기

○ 개인 용품 등을 주기적으로 소독하기

○ 작업공간을 충분히 환기 후 작업 실시하기

○ 맨손 또는 작업용 장갑을 낀 채로 얼굴(특히 눈, 코, 입)을 만지지 않기

○ 조회(TBM), 교육 등은 온라인 또는 유인물 배포 등을 활용, 대면이 필요한 경우는 마스크 착용, 손 소독제 사용 등 개인위생수칙을 준수한 후 가급적 소규모로 실시

○ 가능하면 작업순서를 조정하여 다수가 밀집하여 작업하지 않기

○ 소규모 모임, 회식 등은 자제하고, 퇴근 후 일찍 귀가하기

○ 점심시간 및 휴게시간은 시차를 두어 분산하여 이용하기

○ 구내식당 이용 시 가급적 일렬 또는 지그재그로 앉고 대화는 자제하기

○ 엘리베이터 등 밀폐된 공간에서는 마스크를 착용하고 대화를 자제하기

○ 휴게시설 등은 여러 명이 함께 이용하지 않기

2 사업주(원청 포함)

[공통사항]

○ 방역을 관리하는 담당부서(관리자)를 지정하고 지역 보건소 담당자의 연락망을 확보하는 등 방역 협력체계 구축하기

○ 공동체 내 밀접 접촉이 일어나는 동일 부서, 동일 장소 등에 2~3명 이상의 유증상자가 3~4일 내에 발생 시 유증상자가 코로나19 검사를 받도록 안내하며, 유증상자가 추가 발생 시 보건소에 집단감염 가능성을 신고하기

○ 종사자가 발열 또는 호흡기 증상이 있는 경우 출근 중단 및 즉시 퇴근 조치하기

○ 사람 간 간격을 2m(최소 1m) 이상 거리 두기

○ 손을 씻을 수 있는 시설 또는 손 소독제를 비치하고, 손 씻기 및 기침예절 준수 안내문 게시하기

○ 자연 환기가 가능한 경우 창문을 상시 열어두고, 에어컨 사용 등으로 상시적으로 창문을 열어두기 어려운 경우 2시간마다 1회 이상 환기하기

○ 공용으로 사용하는 물건(출입구 손잡이 등) 및 표면은 매일 1회 이상 자주 소독하기

○ 고객(이용자)을 직접 응대하는 경우 마스크 착용하게 하기

○ 발열 또는 호흡기 증상이 있거나 최근 14일 이내 해외여행을 한 경우 방문 자제 안내하기

○ 실내 다중이용시설을 이용하는 경우 마스크 착용 안내하기

○ 실외에서 2m 거리 유지가 안되는 경우 마스크 착용 안내하기

[해당 유형 적용사항]

○ 방역관리자는 근로자 밀집도, 환기상태, 업무방식 등을 고려하여 방역지침 만들기

○ 매일 비접촉식 체온계나 열화상카메라 등으로 증상 여부(발열, 호흡기 증상 등) 확인하기

○ 조회(TBM), 교육 등은 온라인 또는 유인물 배포 등으로 대체, 대면이 필요한 경우는 체온 측정, 마스크 착용, 소독용품 비치 후 가급적 소규모로 실시하기

○ 작업대 위치 및 방향을 조정하거나 유휴공간을 활용하여 가급적 근로자 간 간격을 2m(최소 1m) 이상 유지하도록 안내하기

○ 통풍이 불충분한 작업 공간은 충분히 환기된 상태에서 작업 실시하기

○ 작업자의 동선, 같은 장소에서의 작업자 수 등을 고려하여 작업계획 수립하기

○ 현장 내 개인 청결을 위한 개수대 등을 설치하기

○ 침방울이 튀는 행위(구호외치기 등) 유도하지 않기

○ 점심시간 및 휴게시간은 시차를 두어 분산하여 운영하기

(예: A조/11:30~12:30, B조/12:30~13:30)

○ 구내식당 좌석 간 투명 가림막을 설치하거나 가급적 일렬 또는 지그재그로 앉게 하기

○ 개인용 청소·소독용품을 지급 또는 비치하기

○ 마스크 및 위생물품을 사업장 상황에 맞게 지급·비치하거나 구입 지원하기

○ 손 씻기, 손 소독제 사용, 기침예절 등 위생관리방안을 게시 또는 교육하기

○ 휴게실 등은 여러 명이 함께 이용하지 않도록 안내하기

1-9. 생활 속 거리 두기 지침 : 은행지점

1 이용자

[공통사항]

○ 발열 또는 호흡기 증상(기침, 인후통 등)이 있거나 최근 14일 이내 해외여행을 한 경우 방문 자제하기

○ 다른 사람과 2m(최소 1m) 이상 거리 두기

○ 흐르는 물과 비누로 30초 이상 손을 씻거나 손 소독제로 손 소독하기

○ 기침이나 재채기를 할 때는 휴지, 옷소매로 입과 코 가리기

○ 침방울이 튀는 행위(노래부르기, 소리지르기 등)나 신체접촉(악수, 포옹 등) 자제하기

○ 실내 다중이용시설을 이용하는 경우 마스크 착용하기

○ 실외에서 2m 거리 유지가 안되는 경우 마스크 착용하기

[해당 유형 적용사항]

○ 비대면 채널(스마트뱅킹, 인터넷뱅킹, 폰뱅킹 및 전화상담 등)을 최대한 활용해 은행지점 방문을 최소화하기

○ 은행 지점 방문시 ATM기기를 적극 활용하기

2 책임자·종사자

[공통사항]

○ 방역관리자 지정 및 지역 보건소 담당자의 연락망을 확보하는 등 방역 협력체계 구축하기

○ 공동체 내 밀접 접촉이 일어나는 동일 부서, 동일 장소 등에 2~3명 이상의 유증상자가 3~4일 내에 발생 시 유증상자가 코로나19 검사를 받도록 안내하며, 유증상자가 추가 발생 시 보건소에 집단감염 가능성을 신고하기

○ 종사자가 발열 또는 호흡기 증상이 있는 경우 출근 중단 및 즉시 퇴근 조치하기

○ 사람 간 간격을 2m(최소 1m) 이상 거리 두기

○ 손을 씻을 수 있는 시설 또는 손 소독제를 비치하고, 손 씻기 및 기침예절 준수 안내문 게시하기

○ 자연 환기가 가능한 경우 창문을 상시 열어두고, 에어컨 사용 등으로 상시적으로 창문을 열어두기 어려운 경우 2시간마다 1회 이상 환기하기

○ 공용으로 사용하는 물건(출입구 손잡이 등) 및 표면은 매일 1회 이상 자주 소독하기

○ 고객(이용자)을 직접 응대하는 경우 마스크 착용하기

○ 발열 또는 호흡기 증상이 있거나 최근 14일 이내 해외여행을 한 경우 방문 자제 안내하기

○ 실내 다중이용시설을 이용하는 경우 마스크 착용 안내하기

○ 실외에서 2m 거리 유지가 안되는 경우 마스크 착용 안내하기

[해당 유형 적용사항]

○ 비대면 채널(스마트 뱅킹, 인터넷 뱅킹, 폰뱅킹 및 전화상담, ATM기기 등) 이용 안내하기

○ 자체실정에 맞는 방역대책 마련·시행하기(예: 은행 창구 투명 가림막 설치, 마스크 미착용 방문객 1회용 마스크 제공, 열화상카메라 설치 등 발열 체크 등)

○ 손 소독제 등 방역 물품 비치(은행 창구, ATM기기, 공용공간, 화장실 등), 손이 닿는 출입문 등 수시 소독하기

○ 창구 직원 대상으로 예방 수칙, 손 씻기, 기침예절 등 감염병 예방 교육 실시하기

1-10. 생활 속 거리 두기 지침 : 물류센터

1 근로자

[공통사항]

○ 발열 또는 호흡기 증상(기침, 인후통 등)이 있거나 최근 14일 이내 해외여행을 한 경우 출근을 자제하기
○ 다른 사람과 2m(최소 1m) 이상 거리 두기
○ 흐르는 물과 비누로 30초 이상 손을 씻거나 손 소독제로 손 소독하기
○ 기침이나 재채기를 할 때는 휴지, 옷소매로 입과 코 가리기
○ 실내 다중이용시설을 이용하는 경우 마스크 착용하기
○ 실외에서 2m 거리 유지가 안 되는 경우 마스크 착용하기
○ 침방울이 튀는 행위(소리지르기, 구호외치기 등)는 자제하기

[해당 유형 적용사항]

○ 택배 배송 시 마스크를 착용하고 가능한 비대면으로 전달하기
○ 택배 운반 차량 운행 전·후 소독하기
○ 사업장 내 근로자, 일용직(아르바이트생 포함) 및 방문자는 매일 비접촉식 체온계나 열화상카메라 등으로 증상 여부(발열, 호흡기 증상 등) 확인 및 명부(전자 또는 수기) 기록 관리(4주 보관 후 폐기) 등 방역에 협조하기
○ 손이 자주 닿는 곳 장비 등은 매일 1회 이상 주기적으로 소독하기
- 하역·운반 장비, 공용 택배차량의 운전대·손잡이, 화물 손잡이 등
○ 사업장 내 작업 시 작업자 간 2m 이상 거리를 유지하거나 불가피할 경우 마스크를 착용하고 충분히 환기시키기
○ 물류센터 내 실내 공간에서 작업하거나, 실외라도 2m 거리 유지가 안되는 경우 마스크 착용하기
○ 흡연 시 지정된 흡연장소에서 흡연자 간 2m 이상 이격하고 마주 보고 말하지 않기
○ 통근버스, 엘리베이터 등 밀폐된 공간에서는 반드시 마스크를 착용하고 옆 사람과 대화를 자제하기
○ 개인별 작업복은 위생관리를 철저히 하고 타인과 공유하지 않기
○ 밀폐된 실내 사업장에서 근무하는 동안에는 마스크 상시 착용하고 근무하기
○ 구내식당 이용 시 가급적 일렬 또는 지그재그로 앉고 대화는 자제하기

○ 근무 중 발열, 기침 등이 나타나면 사업주에게 알린 후 마스크 착용하고 퇴근하기
○ 유연근무제(재택근무, 시차출퇴근 등), 휴가제도(가족돌봄휴가, 연차휴가, 병가 등) 적극 활용하기
○ 사무실, 작업장 등을 환기하기
○ 휴게실 등은 여러 명이 함께 이용하지 않기
○ 소규모 모임, 동아리활동, 회식 등은 자제하고, 퇴근 후 일찍 귀가하기

2 사업주

[공통사항]

○ 방역을 관리하는 담당부서(관리자)를 지정하고 지역 보건소 담당자의 연락망을 확보하는 등 방역 협력체계 구축하기
○ 공동체 내 밀접 접촉이 일어나는 동일 부서, 동일 장소 등에 2~3명 이상의 유증상자가 3~4일 내에 발생 시 유증상자가 코로나19 검사를 받도록 안내하며, 유증상자가 추가 발생 시 보건소에 집단감염 가능성을 신고하기
○ 종사자가 발열 또는 호흡기 증상이 있는 경우 출근 자제 및 즉시 퇴근 조치하기
○ 사람 간 간격을 2m(최소 1m) 이상 거리 두기
○ 손을 씻을 수 있는 시설 또는 손 소독제를 비치하고, 손 씻기 및 기침예절 준수 안내문 게시하고 교육하기
○ 자연 환기가 가능한 경우 창문을 상시 열어두고, 에어컨 사용 등으로 상시적으로 창문을 열어두기 어려운 경우 사무실·작업장 면적과 인원을 고려하여 2시간마다 1회 이상 환기하기
○ 공용으로 사용하는 물건(출입구 손잡이 등) 및 표면은 매일 1회 이상 자주 소독하기
○ 고객(이용자)을 직접 응대하는 경우 마스크 착용하게 하기
○ 발열 또는 호흡기 증상이 있거나 최근 14일 이내 해외여행을 한 경우 방문 자제 안내하기
○ 실내 다중이용시설을 이용하는 경우 마스크 착용 안내하기
○ 실외에서 2m 거리 유지가 안 되는 경우 마스크 착용 안내하기

[해당 유형 적용사항]

○ 택배 배송 시 대면 접촉자 마스크 착용 및 가급적 비대면으로 전달하도록 교육·관리하기
○ 택배 운반 차량 및 허브간 이동 차량 운전자는 발송 전, 하역 후 손이 자주

닿는 운전대 등의 소독 ·확인 관리하기

○ 사업장 채용 일용직(아르바이트생 포함) 방역지침 교육 시행 및 명부(전자 또는 수기)를 기록 관리(4주 보관 후 폐기)하기

- 일용직 근로자도 유증상 시 책임자에게 보고하고, 불이익 없이 즉시 퇴근할 수 있도록 안내하기

○ 사업장 외부인 출입금지하고 불가피한 경우 외부인의 방문목적 및 명부(전자 또는 수기)를 기록 관리(4주 보관 후 폐기)하기

○ 작업장 내 냉동/특수작업복(유니폼 포함)은 개인별로 지급하고 공유시키지 않기

○ 작업공간에서는 자주 환기하며, 송풍기 등을 이용할 때에는 작업자에게 바람이 가지 않도록 지도하기

○ 밀폐된 사업장 내에서 근무하는 종사자에 대해 가급적 마스크를 상시 착용할 수 있도록 지도하기

○ 물류시설별로(필요 시 구역별로) 방역 책임자를 지정하고, 마스크 착용 여부 및 간격 유지 등을 수시 점검할 수 있도록 하기

○ 매일 비접촉식 체온계나 열화상카메라 등으로 체온 검사, 호흡기 증상여부 확인하기

○ 하역·운반 장비 등은 매일 1회 이상 소독 안내하기

○ 냉장·냉동창고는 매일 1회 이상 소독하기

○ 마스크 및 위생물품을 사업장 상황에 맞게 지급·비치하거나 구입 지원하기

○ 작업대 위치 및 방향을 조정하거나 근로자 간 간격을 2m(최소 1m) 이상 유지하되 불가피한 경우 마스크를 착용하고 충분한 환기가 되는지 확인하기

○ 휴식 시간을 보장하고 휴게실 등은 여러 명이 함께 이용하지 않도록 분위기 조성하기

○ 흡연 장소에 2m 거리 두기를 가시적으로 표시(스티커, 페인트 등)하기

○ 통근버스, 엘리베이터 등 밀폐된 공간에서는 반드시 마스크를 착용하고, 대화를 자제하도록 안내하기

○ 근로자 밀집도, 환기 상태, 작업방식 등을 고려하여 방역지침 만들고 이행 여부를 수시로 확인하기

○ 침방울이 튀는 행위(소리지르기, 구호외치기 등) 유도하지 않기

○ 구내식당 좌석 간 투명 가림막을 설치하거나 가급적 일렬 또는 지그재그로 앉게 하기

○ 소규모 모임, 동아리 활동, 회식 등은 자제하고, 퇴근 후 일찍 귀가하기

1-11. 생활 속 거리 두기 지침 : 전시행사

※ 불가피하게 행사를 개최하는 경우, 「코로나19 집단행사 방역관리 지침(2판, '20.02,26)」 및 「코로나19 관련 정부·지자체 행사 운영지침(2판, '20.02.26)」 등에 따른 방역 조치를 반드시 준수하고, 그 외 세부사항은 본 세부지침을 준용

1 이용자

[공통사항]

○ 발열 또는 호흡기 증상(기침, 인후통 등)이 있거나 최근 14일 이내 해외여행을 한 경우 방문 자제하기

○ 다른 사람과 2m(최소 1m) 이상 거리 두기

○ 흐르는 물과 비누로 30초 이상 손을 씻거나 손 소독제로 손 소독하기

○ 기침이나 재채기를 할 때는 휴지, 옷소매로 입과 코 가리기

○ 침방울이 튀는 행위(노래부르기, 소리지르기 등)나 신체접촉(악수, 포옹 등) 자제하기

○ 실내 다중이용시설을 이용하는 경우 마스크 착용하기

○ 실외에서 2m 거리 유지가 안되는 경우 마스크 착용하기

[해당 유형 적용사항]

○ 가능하다면 온라인 또는 영상으로 참여하기

○ 불가피하게 현장 참여를 하는 경우 다른 사람과 2m(최소 1m) 이상 거리 두기, 마스크 착용, 손위생(물과 비누로 30초 이상 손 씻기, 손 소독제 사용) 등 개인 방역수칙 준수하기

○ 전시회 입장 시 현장 등록보다 사전 온라인 등록하기

- 불가피하게 현장 등록을 하는 경우 등록신청서(신원확인) 작성하기

○ 고위험군(65세 이상 어르신, 임신부, 만성질환자 등)은 가급적 전시회 방문 자제하기

○ 전시회 출입 시 손 위생, 증상여부(발열, 호흡기 증상 등) 확인 및 명부(전자 또는 수기) 기록 관리(4주 보관 후 폐기) 등 방역에 협조하기

○ 한 개의 전시 부스에 2m(최소 1m) 거리 두기가 가능하도록 분산하여 관람하기

○ 전시회 참가업체 및 바이어와 상담 시 악수 등 신체접촉을 자제하고 밀폐된 공간을

피하며 마스크를 착용하고 상담 진행하기

○ 상담회장, 회의실 등 다중이용시설 이용 시 2m(최소 1m) 거리 두기 유지가 가능하도록 지그재그로 앉고 대화는 자제하기

○ 전시회 현장에서 물품을 직접 구매하는 경우 현금보다는 전자 결제방식 이용하기 (모바일페이, QR코드, NFC카드, 신용카드 등)

○ 전시시설 내에서 음식 섭취 자제하기

○ 실내 휴게시설, 카페, 매점 등 다중이용공간에서 다른 사람과 2m(최소 1m) 거리 두기가 가능하도록 분산하여 이용하기

○ 줄서기 등 대기할 때에는 사람 간 2m(최소 1m) 간격을 두기

○ 전시회 참여 중 발열, 호흡기 증상이 나타나면 보건용 마스크를 착용하고 전시회 관계자에게 알리기

※ 전시행사 시설 내 기념식, 음식점·카페, 공중화장실 등 이용 시 해당 유형의 지침을 준용

2 책임자·종사자

[공통사항]

○ 방역관리자 지정 및 지역 보건소 담당자의 연락망을 확보하는 등 방역 협력체계 구축하기

○ 공동체 내 밀접 접촉이 일어나는 동일 부서, 동일 장소 등에 2~3명 이상의 유증상자가 3~4일 내에 발생 시 유증상자가 코로나19 검사를 받도록 안내하며, 유증상자가 추가 발생할 시 보건소에 집단감염 가능성 신고하기

○ 종사자가 발열 또는 호흡기 증상이 있는 경우 출근 중단 및 즉시 퇴근 조치하기

○ 사람 간 간격을 2m(최소 1m) 이상 거리 두기

○ 손을 씻을 수 있는 시설 또는 손 소독제를 비치하고, 손 씻기 및 기침예절 준수 안내문 게시하기

○ 자연 환기가 가능한 경우 전시장 문 및 창문을 상시 열어두고, 에어컨 사용 등으로 상시적으로 창문을 열어두기 어려운 경우 2시간마다 1회 이상 환기하기

○ 공용으로 사용하는 물건(출입구 손잡이 등) 및 표면은 매일 1회 이상 자주 소독하기

○ 고객(이용자)을 직접 응대하는 경우 마스크 착용하기

○ 발열 또는 호흡기 증상이 있거나 최근 14일 이내 해외여행을 한 경우 방문 자제 안내하기

○ 실내 다중이용시설을 이용하는 경우 마스크 착용 안내하기

○ 실외에서 2m 거리 유지가 안되는 경우 마스크 착용 안내하기

[해당 유형 적용사항]

○ 가능하다면 온라인 또는 영상을 활용하는 방식으로 진행하기

○ 불가피하게 현장 전시회를 개최할 시 코로나19 예방을 위한 방역 조치를 충분히 병행하여 추진하기

○ 현장 전시회를 개최하는 경우, 온라인·오프라인 병행, 사전 예약제, 시간제 운영 등의 방식을 활용하여 참관객이 한꺼번에 몰리지 않도록 분산 유도하기

○ 현장에서 등록하기보다 사전 온라인 등록 안내하기

○ 전시장이 밀집되지 않고 2m(최소 1m) 거리 두기가 유지되도록 입장 정원을 제한하기

* 예) 행사장 바닥 면적 4㎡ 당 입장객 1명으로 제한, 행사장 수용 인원의 50%로 입장 정원 제한 등 거리 두기가 가능한 방안 마련

○ 참관객, 진행요원 등 모든 전시장 출입자의 증상여부(발열, 호흡기 증상 등) 확인 및 명부(전자 또는 수기)를 기록 관리(4주 보관 후 폐기)하고, 등록신청서(신원확인) 제출자에 한해 출입을 허용하기

- 전시시설, 전시회 주최기관, 참가업체 직원 대상 체온 등 증상 여부 일일 2회 점검 후 대장 작성 하기

○ 입구와 출구를 달리하여 이용자들이 한 방향으로 이동하도록 동선을 마련하기

○ 한 개의 전시 부스 내에 참관객 간 2m(최소 1m) 거리 두기가 가능하도록 한꺼번에 밀집되지 않도록 하기

○ 이동 시 참관객 및 이용자 간 2m(최소 1m) 거리 두기가 유지될 수 있도록 통로를 충분히 확보하기

○ 전시회 참가업체와 바이어는 상담 시 악수 등 가급적 신체접촉을 자제하고 밀폐된 공간을 피하며 마스크 착용 후 상담진행 하도록 유도 및 수시 안내하기

○ 경연대회, 설명회 등 협소한 공간에 다수의 인원이 모이는 부대행사 운영은 자제하기

○ 침방울이 튀는 행위(노래부르기, 구호외치기 등)와 프로그램은 최소화하기

○ 마이크를 사용하는 경우 마이크는 커버를 씌우고, 개인마다 마이크 덮개를 새것으로 교체하기

○ 전시장 외부에 기업, 제품 홍보 부스(코너)는 운영을 중단하거나 최소화하되, 불가피하게 운영 시에는 부스 간에 2m 이상 간격을 유지하고, 참여자들이 한꺼번에

모이지 않도록 안내하기

○ 생활 속 거리 두기(현수막, 문자전광판, 안내방송 등) 및 개인 위생수칙 홍보·안내하기

○ 단체식사 제공은 자제하며, 불가피한 경우에는 개인 도시락 형태로 제공하기

○ 가능한 포장 판매 활성화 및 대규모 인원이 밀집되지 않도록 유도하기

○ 현장 결제 시 전자 결제방식(모바일페이, QR코드, 신용카드 등)을 활용하도록 하고 필요 시 투명 가림막 등을 설치하기

○ 식당, 휴게실 등 다중이용시설 이용 시 2m(최소 1m) 거리 두기가 유지 가능하도록 한 칸 띄어 지그재그로 앉도록 자리를 배치하기

○ 외국 업체, 해외 거주민 등의 국내 초청은 자제하기

- 불가피하게 초청할 경우 '코로나19 대한민국 입국자를 위한 격리 주의사항 안내' 등 방역 당국의 해외 입국자 조치사항 확인·안내하기(해외 인사 초청 시, 해외 입국자에 대해 의무 자가격리 필요 등)

○ 전시회 출입구 대기열에서 2m(최소 1m) 이상 거리를 유지할 수 있도록 바닥 스티커 등으로 표시하고 안내하기

○ 출입구 및 시설 내 여러 곳에 손 소독제·휴지 및 뚜껑 있는 쓰레기통 비치하기

○ 전시행사 전・중・후 소독 및 환기 실시(일시, 관리자 확인 포함 대장 작성)하기

○ 셔틀버스 운영 시, 마스크 착용 및 다른 사람과 2m(최소 1m) 이상 거리가 유지되도록 한 칸씩 띄어 앉고 대화 자제 안내하기

○ 매표원, 출입 관리요원 등은 수시로 손세정제(위생장갑 착용시 수시로 교체) 사용하기

○ 진행요원 등의 식사 시간은 시차를 두고 운영하여 관계자 간 밀집을 최소화하기 (예: A조/18:30~19:30, B조/19:30~20:30)

※ 전시행사 시설 내 기념식, 음식점·카페, 공중화장실 등 이용 시 해당 유형의 지침을 준용

Ⅱ. 일 상 (1. 이동할 때)

1-1. 생활 속 거리 두기 지침 : 대중교통

1 이용자

[공통사항]

○ 발열 또는 호흡기 증상(기침, 인후통 등)이 있거나 최근 14일 이내 해외여행을 한 경우 이용 자제하기

○ 다른 사람과 2m(최소 1m) 이상 거리 두기

○ 흐르는 물과 비누로 30초 이상 손을 씻거나 손 소독제로 손 소독하기

○ 기침이나 재채기를 할 때는 휴지, 옷소매로 입과 코 가리기

○ 침방울이 튀는 행위(큰소리로 대화, 불필요한 대화, 통화 등)나 신체접촉(악수, 포옹 등) 자제하기

○ 실내 다중이용시설을 이용하는 경우 마스크 착용하기

○ 실외에서 2m 거리 유지가 안되는 경우 마스크 착용하기

[해당 유형 적용사항]

○ 버스, 지하철, 택시, 기차, 항공 이용 시 마스크 착용하고 대화 자제하기

○ 대중교통 이용 시 또는 항공 보안검색과 입출국 심사 시 최대한 다른 사람과 거리 유지하기

○ 기차·고속버스·항공 등 좌석제 대중교통 좌석 예매 시 한 좌석 띄워(창가 좌석 우선 예매 등) 예매하고, 가능한 비대면 서비스(모바일 체크인 등) 우선하여 이용하기

○ 차내가 혼잡할 경우에는 가능하면 다음 차 이용하기

○ 택시 이용 시 비대면 자동결제 방식, 신용카드 등 전자결제를 활용하기

○ 대중교통 내에서 전화 통화할 때도 마스크 착용하기

○ 대중교통 내에서 음식물 섭취 자제하기

2 책임자·종사자

[공통사항]

- ○ 방역관리자 지정 및 지역 보건소 담당자의 연락망을 확보하는 등 방역 협력체계 구축하기
- ○ 공동체 내 밀접 접촉이 일어나는 동일 부서, 동일 장소 등에 2~3명 이상의 유증상자가 3~4일 내에 발생 시 유증상자가 코로나19 검사를 받도록 안내하며, 유증상자가 추가 발생 시 보건소에 집단감염 가능성을 신고하기
- ○ 종사자가 발열 또는 호흡기 증상이 있는 경우 출근 중단 및 즉시 퇴근 조치하기
- ○ 사람 간 간격을 2m(최소 1m) 이상 거리 두기
- ○ 손을 씻을 수 있는 시설 또는 손 소독제를 비치하고, 손 씻기 및 기침예절 준수 안내문 게시하기
- ○ 자연 환기가 가능한 경우 창문을 상시 열어두고, 에어컨 사용 등으로 상시적으로 창문을 열어두기 어려운 경우 2시간마다 1회 이상 환기하기
- ○ 공용으로 사용하는 물건(출입구 손잡이 등) 및 표면은 매일 1회 이상 자주 소독하기
- ○ 고객(이용자)을 직접 응대하는 경우 마스크 착용하기
- ○ 발열 또는 호흡기 증상이 있거나 최근 14일 이내 해외여행을 한 경우 이용 자제 안내하기
- ○ 실내 다중이용시설을 이용하는 경우 마스크 착용 안내하기
- ○ 실외에서 2m 거리 유지가 안되는 경우 마스크 착용 안내하기

[해당 유형 적용사항]

- ○ 대중교통(항공 포함) 이용시 마스크 착용, 최대한 다른 사람과 거리를 유지하도록 안내하기
- ○ 환기가 가능한 교통 수단에 대해서는 운영 전후 자주 환기하기
- ○ 대중교통 밀집도 완화를 위해 혼잡 시간대를 수시로 파악하여 유연하게 배차 조정하기
- ○ 철도, 항공, 고속·시외버스 등 예약 시 창가 우선 배정 등 승객 간 좌석을 띄워 배정하기
- ○ 승차권 예약 또는 택시 호출 시 결제 방법을 비대면 자동결제 방식으로 유도하기
- ○ 전광판, 안내방송 등을 통해 이용자 준수 예방수칙 홍보하기

1-2. 생활 속 거리 두기 지침 : 여객선(국제·연안)

1 이용자

[공통사항]

- ○ 발열 또는 호흡기 증상(기침, 인후통 등)이 있거나 최근 14일 이내 해외여행을 한 경우 이용 자제하기
- ○ 다른 사람과 2m(최소 1m) 이상 거리 두기
- ○ 흐르는 물과 비누로 30초 이상 손을 씻거나 손 소독제로 손 소독하기
- ○ 기침이나 재채기를 할 때는 휴지, 옷소매로 입과 코 가리기
- ○ 침방울이 튀는 행위(큰소리로 대화, 불필요한 대화, 통화 등)나 신체접촉(악수, 포옹 등) 자제하기
- ○ 실내 다중이용시설을 이용하는 경우 마스크 착용하기
- ○ 실외에서 2m 거리 유지가 안되는 경우 마스크 착용하기

[해당 유형 적용사항]

- ○ 여객선 승선 시 증상여부(발열, 호흡기 증상 등) 확인 등 방역에 협조하기
- ○ 여객선 이용 직전 또는 이용 중, 의심증상 발현 시에는 즉시 지역보건소에 신고하고, 보건당국의 조치가 있기 전까지 별도 격리장소에서 대기하기
- ○ 대합실, 객실 등 공동이용 장소에서 타인과 거리 유지하기
- ○ 객실 좌석은 가급적 한 칸 띄워 앉기
- ○ 여객선 및 여객터미널 내에서 마스크를 착용하기
- ○ 연안여객선은 객실이 혼잡할 경우 객실갑판 등 열린 공간에서 대기하기

2 책임자·종사자

[공통사항]

- ○ 방역관리자 지정 및 지역 보건소 담당자의 연락망을 확보하는 등 방역 협력체계 구축하기
- ○ 공동체 내 밀접 접촉이 일어나는 동일 부서, 동일 장소 등에 2~3명 이상의 유증상자가 3~4일 내에 발생 시 유증상자가 코로나19 검사를 받도록 안내하며, 유증상자가 추가 발생 시 보건소에 집단감염 가능성을 신고하기

○ 종사자가 발열 또는 호흡기 증상이 있는 경우 출근 중단 및 즉시 퇴근 조치하기
○ 사람 간 간격을 2m(최소 1m) 이상 거리 두기
○ 손을 씻을 수 있는 시설 또는 손 소독제를 비치하고, 손 씻기 및 기침예절 준수 안내문 게시하기
○ 자연 환기가 가능한 경우 창문을 상시 열어두고, 에어컨 사용 등으로 상시적으로 창문을 열어두기 어려운 경우 2시간마다 1회 이상 환기하기
○ 공용으로 사용하는 물건(출입구 손잡이 등) 및 표면은 매일 1회 이상 자주 소독하기
○ 고객(이용자)을 직접 응대하는 경우 마스크 착용하기
○ 발열 또는 호흡기 증상이 있거나 최근 14일 이내 해외여행을 한 경우 방문 자제 안내하기
○ 실내 다중이용시설을 이용하는 경우 마스크 착용 안내하기
○ 실외에서 2m 거리 유지가 안되는 경우 마스크 착용 안내하기

[해당 유형 적용사항]

○ 터미널 출·입구를 최소한으로 개방하여 이동경로를 단순화하고, 모든 터미널 이용객이 손 소독 후 터미널을 이용할 수 있도록 조치하기
○ 지정좌석제의 경우 좌석간 거리를 두고 한 칸씩 띄워 앉도록 조정하기
○ 연안여객선의 다인실은 일부 다인실에 집중되지 않고 전체 다인실에 분산되도록 배치 및 발권 조정하여 거리 두기 유지하게 하기
○ 국제카페리선은 개인실 위주로 운영하되, 부득이 다인실로 운영시 침상간 간격을 1~2m 이상 두어 이용객 간 거리 조정하기
○ 여객선에 승선하는 승객에 대한 증상여부(발열, 호흡기 증상 등) 확인하기

Ⅱ. 일 상 (2. 식사할 때)

2-1. 생활 속 거리 두기 지침 : 음식점 · 카페/스터디카페

1 이용자

[공통사항]

○ 발열 또는 호흡기 증상(기침, 인후통 등)이 있거나 최근 14일 이내 해외여행을 한 경우 방문 자제하기

○ 다른 사람과 2m(최소 1m) 이상 거리 두기

○ 흐르는 물과 비누로 30초 이상 손을 씻거나 손 소독제로 손 소독하기

○ 기침이나 재채기를 할 때는 휴지, 옷소매로 입과 코 가리기

○ 침방울이 튀는 행위(노래부르기, 소리지르기 등)나 신체접촉(악수, 포옹 등) 자제하기

○ 실내 다중이용시설을 이용하는 경우 마스크 착용하기

○ 실외에서 2m 거리 유지가 안되는 경우 마스크 착용하기

[해당 유형 적용사항]

1 일반식당

○ 혼잡한 시간대를 피해서 방문하고 머무르는 시간 최소화하기

○ 일행이 아닌 다른 사람들과 2m(최소 1m) 이상 거리를 유지하며, 가급적 최대한 간격 두고 앉기

○ 가능한 지그재그로 앉거나 한 방향을 바라보도록 앉기

○ 식사 전, 흐르는 물에 비누로 30초 이상 손을 씻거나, 손 소독제로 손 소독하기

○ 식당을 입장할 때와 식사 시간을 제외한 식사 전·후 대화 시에는 마스크 착용하고, 식사를 할 때는 대화를 자제하기

○ 침방울이 튀는 행위(큰 소리로 말하기, 노래 부르기, 구호 외치기 등)하지 않기

○ 식사 중에라도 음식을 가지러 가는 등의 이동 시에는 마스크를 착용하고 다른 사람과 2m(최소 1m) 이상 거리 지속적으로 유지하기

○ 가능한 포장 및 배달 주문 등을 이용하거나 야외 탁자를 이용하기

○ 술잔, 식기 등은 함께 사용하지 않고, 개인별로 사용하기

○ 공용으로 먹는 음식은 공용집게 등을 사용하고, 침방울이 섞이지 않도록 주의하기

○ 공용집게・접시・수저 등 사용 전・후 손 소독제 또는 비닐장갑을 사용하기

2 뷔페

○ 출입시 증상여부(발열, 호흡기 증상 등) 확인 및 명부(전자 또는 수기) 기록 관리 (4주 보관 후 폐기) 등 방역에 협조하기

○ 혼잡한 시간대를 피해서 방문하고 머무르는 시간 최소화하기

○ 일행이 아닌 다른 사람들과 2m(최소 1m) 이상 거리를 유지하며, 가급적 최대한 간격 두고 앉기

○ 식사 전, 흐르는 물에 비누로 30초 이상 손을 씻거나, 손 소독제로 손 소독하기

○ 식당을 입장할 때와 식사 시간을 제외한 식사 전·후 대화 시에는 마스크 착용하고, 식사를 할 때는 대화를 자제하기

○ 식사 중에라도 음식을 가지러 이동하거나 대기하는 경우 마스크를 착용하고, 다른 사람과 2m(최소 1m) 이상 거리 지속적으로 유지하기

○ 공용집게・접시・수저 등 사용 전・후 손 소독제 또는 비닐장갑을 사용하기

○ 술잔, 식기 등은 함께 사용하지 않고, 개인별로 사용하기

○ 한 접시에 담긴 음식 나눠 먹지 않기

3 구내식당

○ 분산된 시간에 이용하고 머무르는 시간 최소화하기

(예: 1조 11:30~12:00, 2조 12:00~12:30, 3조 12:30~13:00)

○ 가능한 지그재그로 앉거나 한 방향을 바라보도록 앉기

○ 식사 전, 흐르는 물에 비누로 30초 이상 손을 씻거나, 손 소독제로 손 소독하기

○ 식당을 입장할 때와 식사 시간을 제외한 식사 전·후 대화 시에는 마스크 착용하고, 식사를 할 때는 대화를 자제하기

○ 식사 중에라도 음식을 가지러 가는 등의 이동 시에는 마스크를 착용하고 다른 사람과 2m(최소 1m) 이상 거리 지속적으로 유지하기

○ 공용집게・접시・수저 등 사용 전・후 손 소독제 또는 비닐장갑을 사용하기

4 카페/스터디카페

○ 카페 등에 머무르는 시간 최소화하기

○ 탁자 사이 간격을 2m(최소 1m) 두고 앉거나, 일행이 아닌 다른 사람들과 가급적 최대한 간격을 띄워 앉기

○ 가능한 지그재그로 앉거나 한 방향을 바라보도록 앉기

○ 카페 내에서는 대화를 자제하기

○ 먹거나 마시는 시간 외에는 마스크 착용하기

○ 음식은 각자 개인 접시에 덜어 먹기

○ 가능한 포장 및 배달주문 등을 이용하거나 야외 탁자를 이용하기

2 책임자·종사자

[공통사항]

○ 방역관리자 지정 및 지역 보건소 담당자의 연락망을 확보하는 등 방역 협력체계 구축하기

○ 공동체 내 밀접 접촉이 일어나는 동일 부서, 동일 장소 등에 2~3명 이상의 유증상자가 3~4일 내에 발생 시 유증상자가 코로나19 검사를 받도록 안내하며, 유증상자가 추가 발생 시 보건소에 집단감염 가능성을 신고하기

○ 종사자가 발열 또는 호흡기 증상이 있는 경우 출근 중단 및 즉시 퇴근 조치하기

○ 사람 간 간격을 2m(최소 1m) 이상 거리 두기

○ 손을 씻을 수 있는 시설 또는 손 소독제를 비치하고, 손 씻기 및 기침예절 준수 안내문 게시하기

○ 자연 환기가 가능한 경우 창문을 상시 열어두고, 에어컨 사용 등으로 상시적으로 창문을 열어두기 어려운 경우 2시간마다 1회 이상 환기하기

○ 공용으로 사용하는 물건(출입구 손잡이 등) 및 표면은 매일 1회 이상 자주 소독하기

○ 고객(이용자)을 직접 응대하는 경우 마스크 착용하기

○ 발열 또는 호흡기 증상이 있거나 최근 14일 이내 해외여행을 한 경우 방문 자제 안내하기

○ 실내 다중이용시설을 이용하는 경우 마스크 착용 안내하기
○ 실외에서 2m 거리 유지가 안되는 경우 마스크 착용 안내하기

[해당 유형 적용사항]

1 일반식당

○ 개인 위생수칙 준수, 생활 속 거리 두기의 필요성 등에 대해 주기적으로 종사자 교육 실시하기

○ 이용자들의 밀집을 최소화하도록 이용 인원 제한 또는 시간 예약제 등 실시하기

○ 계산 시 마스크를 착용하고, 비대면기기 또는 투명 가림막 등을 설치하는 방법으로 가급적 고객과 마주 보지 않도록 하기

○ 식사하는 경우 외(식사 전·후, 이동, 대기 등)에는 마스크를 착용하고, 다른 사람과 2m(최소 1m) 이상 거리를 유지하도록 안내하고, 안내문 게시하기

○ 매장 입구, 테이블 등 음식점 내 손 소독제를 비치하기(필요 시 비닐장갑 함께 비치)

○ 탁자 사이 간격을 2m(최소 1m)로 배치하고, 테이블 간에 칸막이 또는 1인 테이블 설치, 고정형 탁자 일부를 사용 금지 등 탁자 간에 거리를 두는 방법 마련하기

○ 의자를 한 방향 또는 지그재그로 배치하는 등 서로 마주 보지 않도록 노력하기

○ 대규모 행사 및 다중이 모이는 이벤트성 행사는 자제하기

- 불가피하게 단체 예약 등 다수가 밀집하는 경우에는 방역수칙 철저하게 준수하도록 안내하기

○ 가능한 포장 및 배달 판매 등을 이용하거나 야외 탁자 이용 활성화하기

○ 대기자 발생 시 번호표를 활용하거나 대기자 간 2m(최소 1m) 이상 간격을 두고 대기하도록 안내하기

○ 음식(반찬, 국 포함)은 되도록 개인별 용기에 제공하고, 음식을 나눠 먹지 않도록 안내하기

- 개별포장 수저 제공 등 수저를 위생적으로 관리하고, 개인 접시에 덜어 먹도록 개인 접시와 국자, 집게 등을 제공하기

○ 큰 소리로 말하기, 노래 부르기, 구호 외치기 등 침방울이 발생하는 행위는 자제하도록 안내하고 유도하기

- 특히, 이용자들이 큰 소리로 대화하지 않도록 업소 내 음악 소리 등 소음 줄이기

○ 자주 사용하는 모든 부위*를 지속적으로 소독하기

* 출입문 손잡이, 스위치, 변기커버 및 뚜껑, 물내림 버튼, 세면대, 수도꼭지, 손 건조기 등

○ '일반식당 유형별 핵심수칙 자가점검표(붙임)'에 따라 점검을 하고 개선 노력하기

2 뷔페

○ 출입 시 증상 여부(발열, 호흡기 증상 등)를 확인하고, 이용자, 종사자 등의 명부(전자 또는 수기)를 기록 관리(4주 보관 후 폐기)하기

○ 개인 위생수칙 준수, 생활 속 거리 두기의 필요성 등에 대해 주기적으로 종사자 교육 실시하기

○ 이용자들의 밀집을 최소화하도록 이용 인원 제한 또는 시간 예약제 등 실시하기

○ 계산 시 마스크를 착용하고, 비대면기기 또는 투명 가림막 등을 설치하는 방법으로 가급적 고객과 마주 보지 않도록 하기

○ 식사하는 경우 외(입장, 식사 전·후, 음식을 가지러 이동, 대기 등)에는 마스크를 착용하고, 다른 사람과 2m(최소 1m) 이상 거리를 유지하도록 안내 및 감독하고, 안내문 게시하기

○ 매장 입구, 테이블 등 음식점 내 손 소독제를 비치하기(필요 시 비닐장갑 함께 비치)

○ 탁자 사이 간격을 2m(최소 1m)로 배치하고, 이용자 간 최대한 간격을 두고 앉도록 안내하기

○ 대규모 행사 및 다중이 모이는 이벤트성 행사는 자제하기

- 불가피하게 단체 예약 등 다중이 모이는 경우에는 방역수칙 철저하게 준수하도록 안내하기

○ 대기자 발생 시 번호표를 활용하거나 대기자 간 2m(최소 1m) 이상 간격을 두고 대기하도록 안내하기

○ 개별포장 수저 제공 등 위생적으로 수저를 관리하고, 한 접시에 담긴 음식을 나눠 먹지 않도록 안내하기

○ 자주 사용하는 모든 부위*를 지속적으로 소독하기

* 출입문 손잡이, 스위치, 변기커버 및 뚜껑, 물내림 버튼, 세면대, 수도꼭지, 손 건조기 등

3 구내식당

○ 이용자들이 시차를 두고 분산 이용하여 밀집을 최소화하도록 유도하기 (예: 1조 11:30~12:00, 2조 12:00~12:30, 3조 12:30~13:00)

○ 개인 위생수칙 준수, 생활 속 거리 두기의 필요성 등에 대해 주기적으로 종사자 교육 실시하기

○ 식사하는 경우 외(입장, 식사 전·후, 이동, 대기 등)에는 마스크를 착용하고 다른 사람과 2m(최소 1m) 이상 거리를 유지하도록 안내하고, 대화 자제하도록 안내 방송 주기적 실시 및 안내문 게시하기

○ 매장 입구, 테이블 등 음식점 내 손 소독제를 비치하기(필요 시 비닐장갑 함께 비치)

○ 좌석 간에 칸막이를 설치하거나 지그재그 또는 한 방향으로 앉도록 안내하기

○ 대기 시 이용자 간 최소 2m(최소 1m) 이상 간격을 두도록 안내하기

○ 자주 사용하는 모든 부위*를 지속적으로 소독하기

* 출입문 손잡이, 스위치, 변기커버 및 뚜껑, 물내림 버튼, 세면대, 수도꼭지, 손 건조기 등

4 카페/스터디카페

○ 계산 시 마스크를 착용하고, 비대면 기기 또는 투명 가림막 등을 설치하는 등 방법으로 가급적 고객과 마주 보지 않고 최대한 간격을 유지하도록 하기

○ 탁자 사이 간격을 가급적 2m(최소 1m) 이상 두거나 테이블 간에 칸막이 설치, 고정형 탁자 일부를 사용 금지 등 탁자 간에 거리를 두는 방법 마련하기

○ 의자를 한 방향 또는 지그재그로 배치하는 등 서로 마주 보지 않도록 노력하기

○ 대규모 행사 개최 자제하기

○ 가능한 포장 및 배달 판매 등을 이용하고 야외공간 이용 활성화하기

○ 개인 위생수칙 준수, 생활 속 거리 두기의 필요성 등에 대해 주기적으로 종사자 교육 실시하기

○ 대기자 발생 시 번호표를 활용하거나 대기자 간 2m(최소 1m) 이상 간격을 두고 대기하도록 안내하기

○ 음식은 각자 개인 접시에 덜어 먹도록 개인 접시와 집게 등을 제공하기

○ 카페 내 공용사용 공간 등 곳곳에 손 소독제 비치하기(필요 시 비닐장갑 함께 비치)

○ 큰 소리로 말하기, 노래 부르기, 구호 외치기 등 침방울이 발생하는 행위는 자제하도록 안내하고 유도하기

ㅇ 자주 사용하는 모든 부위*를 지속적으로 소독하기

* 출입문 손잡이, 스위치, 변기 커버 및 뚜껑, 물내림 버튼, 세면대, 수도꼭지, 손 건조기 등

붙임 일반식당 유형별 핵심수칙 자가점검표

점검일자 : 년 월 일
시 설 명 :
방역관리자 :

① 운영형태별 점검 항목		준수여부 (해당에 v표시)	
		준수	미준수
홀 (Hall)	다른 사람과 2m(최소 1m) 간격 유지, 테이블 간 칸막이 또는 1인 테이블 설치		
	밀집 최소화를 위해 이용 인원 제한 또는 시간예약제 실시		
룸 (Room)	자연환기가 가능한 경우 창문을 상시 열어두기(에어컨 사용으로 창문을 열어두기 어려운 경우 2시간마다 1회 이상 환기하기)		
	머무르는 시간 최소화하도록 안내하기		

② 규모별 점검 항목		준수여부 (해당에 v표시)	
		준수	미준수
$100m^2$ 이상	다중이 모이는 이벤트성 행사 및 단체 예약 자제하기 (불가피하게 단체 예약 등 다수밀집의 경우 방역수칙 철저 준수 안내)		
	침방울이 발생하는 행위(구호외치기, 큰소리로 말하기 등) 자제 안내		
$100m^2$ 미만	머무르는 시간 최소화하도록 안내하기		
	밀집 최소화를 위해 이용 인원 제한 또는 시간예약제 실시		
	가능한 포장 및 배달판매 이용 안내하기		

③ 음식제공 형태별 점검 항목		준수여부 (해당에 v표시)	
		준수	미준수
공동음식 제공	1인 반상 제공 또는 개인별 접시, 집게, 국자 등 제공		
	공용 집게 등 사용 전·후 손 소독이 가능하도록 테이블 등에 손 소독제 비치하기(필요 시 비닐장갑 함께 비치)		
개별음식 제공	음식 또는 음료 등을 나눠 먹지 않도록 안내하기		
	밀집 최소화를 위해 이용 인원 제한 또는 시간예약제 실시		

④ 주류판매 여부별 점검 항목		준수여부 (해당에 v표시)	
		준수	미준수
주류 판매	머무르는 시간 최소화하도록 안내하기		
	침방울이 발생하는 행위(구호외치기, 큰소리로 말하기 등) 자제 안내		
	테이블당 손 소독제 비치 또는 메뉴제공 시 손 소독제 등 함께 제공		
주류 미판매	분산 이용, 밀집을 최소화하도록 식사 시차제 운영		
	공용 집게 등 사용 전·후 손 소독이 가능하도록 테이블 등에 손 소독제 비치하기(필요 시 비닐장갑 함께 비치)		

⑤ 환기가능 여부별 점검 항목		준수여부 (해당에 v표시)	
		준수	미준수
환기 불가능	다른 사람과 2m(최소 1m) 간격 유지, 영업 전후 등 주기적 소독		
	테이블 간 칸막이 또는 1인 테이블 설치		
	테이블당 손 소독제 비치 또는 메뉴 제공 시 손 소독제 등 함께 제공		
환기 가능	자연환기 가능한 경우 창문을 상시 열어두기		
	에어컨을 가동하는 경우 2시간마다 1회 이상 환기하기		

※ 일반식당 유형별 핵심수칙 이행 여부를 자가 진단할 수 있는 점검표입니다.

Ⅱ. 일 상 (3. 공부할 때)

3-1. 생활 속 거리 두기 지침 : 학원 · 독서실 등

1 이용자

[공통사항]

○ 발열 또는 호흡기 증상(기침, 인후통 등)이 있거나 최근 14일 이내 해외여행을 한 경우 방문 자제하기

○ 다른 사람과 2m(최소 1m) 이상 거리 두기

○ 흐르는 물과 비누로 30초 이상 손을 씻거나 손 소독제로 손 소독하기

○ 기침이나 재채기를 할 때는 휴지, 옷소매로 입과 코 가리기

○ 침방울이 튀는 행위(노래부르기, 소리지르기 등)나 신체접촉(악수, 포옹 등) 자제하기

○ 실내 다중이용시설을 이용하는 경우 마스크 착용하기

○ 실외에서 2m 거리 유지가 안되는 경우 마스크 착용하기

[해당 유형 적용사항]

○ 실내 휴게실, 카페, 매점 등 다중이용공간 밀집되지 않도록 분산하여 이용하기

○ 마스크 미착용, 발열 등 유증상자 발견시 서로서로 방역지침 준수 요청하기

○ 고위험군은 시설이용·방문 자제, 불가피하게 방문할 경우 마스크 착용하기

* 고위험군: 65세 이상 어르신, 임신부, 만성질환자 등

○ 출입 시 증상 여부(발열, 호흡기 증상 등) 확인 및 명부(전자 또는 수기) 기록 관리(4주 보관 후 폐기) 등 방역에 협조하기

※ 시설 내 음식점·카페 등 이용 시 해당 유형의 지침을 준용

2 책임자·종사자

[공통사항]

○ 방역관리자 지정 및 지역 보건소 담당자의 연락망을 확보하는 등 방역 협력체계 구축하기

○ 공동체 내 밀접 접촉이 일어나는 동일 부서, 동일 학급, 동일 장소 등에 2~3명 이상의 유증상자가 3~4일 내에 발생 시 유증상자가 코로나19 검사를 받도록 안내하며, 유증상자가 추가 발생 시 보건소에 집단감염 가능성을 신고하기

○ 종사자가 발열 또는 호흡기 증상이 있는 경우 출근 중단 및 즉시 퇴근 조치하기

○ 사람 간 간격을 2m(최소 1m) 이상 거리 두기

○ 손을 씻을 수 있는 시설 또는 손 소독제를 비치하고, 손 씻기 및 기침예절 준수 안내문 게시하기

○ 자연 환기가 가능한 경우 창문을 상시 열어두고, 에어컨 사용 등으로 상시적으로 창문을 열어두기 어려운 경우 2시간마다 1회 이상 환기하기

○ 공용으로 사용하는 물건·기기(출입구 손잡이, 컴퓨터 등) 표면은 매일 1회 이상 자주 소독하기

○ 고객(이용자)을 직접 응대하는 경우 마스크 착용하기

○ 발열 또는 호흡기 증상이 있거나 최근 14일 이내 해외여행을 한 경우 방문 자제 안내하기

○ 실내 다중이용시설을 이용하는 경우 마스크 착용 안내하기

○ 실외에서 2m 거리 유지가 안되는 경우 마스크 착용 안내하기

[해당 유형 적용사항]

○ 강의실 및 열람실 내 2m(최소 1m) 이상 거리 두도록 하고, 옆 자리와 앞 자리가 비도록 지그재그로 자리를 배치하거나 투명 가림막 등 설치하기

○ 강사 등 종사자 강의시 반드시 마스크 착용 및 이용자 대상 마스크 착용 안내하기

○ 출입구 및 시설 내 곳곳에 휴지 및 뚜껑 있는 쓰레기통 비치하기

* 기침이나 재채기 때 사용한 휴지를 깨끗이 버릴수 있어야 함

○ 주요 공간 일상소독*(최소 2회/일), 대형학원(규모 1,000㎡ 이상)의 경우 주 1회 이상 전문방역소독, 수시 환기 실시(일시·관리자 확인을 포함한 대장 작성)하기

* 문손잡이, 난간 등 특히 통행이 빈번한 장소 및 손이 자주 닿는 물건

○ 단체 식사 제공 금지(단, 기숙 및 종일반을 운영하는 학원의 경우 음식점·카페 지침을 준용하여 운영)하기

○ 노트북, 테블릿PC 등 전산용품 이용 시 직원은 가급적 개인기기 사용하기

○ 개인 위생수칙 준수, 생활 속 거리 두기의 필요성 등에 대한 주기적 교육·안내하기

○ 시간대별 이용자수 및 이용공간 제한 등을 통해 이용자 집중 방지하기

○ 고위험군은 시설이용 자제, 불가피하게 방문할 경우 마스크 착용 안내하기

* 고위험군: 65세 이상 어르신, 임신부, 만성질환자 등

○ 출입하는 사람에 대한 증상 여부(발열, 호흡기 증상 등) 확인 및 명부(전자 또는 수기)를 작성 관리(4주 보관 후 폐기)하기

※ 시설 내 음식점·카페 등이 있는 경우 해당 유형의 지침을 준용

3-2. 생활 속 거리 두기 지침 : 고시원

1 이용자

[공통사항]

○ 발열 또는 호흡기 증상(기침, 인후통 등)이 있거나 최근 14일 이내 해외여행을 한 경우 이용을 자제하기

○ 다른 사람과 2m(최소 1m) 이상 거리 두기

○ 흐르는 물과 비누로 30초 이상 손을 씻거나 손 소독제로 손 소독하기

○ 기침이나 재채기를 할 때는 휴지, 옷소매로 입과 코 가리기

○ 침방울이 튀는 행위(노래 부르기, 구호외치기 등)나 신체접촉(악수, 포옹 등) 자제하기

○ 실내 다중이용시설(공용공간)을 이용하는 경우 마스크 착용하기

○ 실외에서 2m 거리 유지가 안되는 경우 마스크 착용하기

[해당 유형 적용사항]

○ 고시원 이용자 외 보호자·외부인 등 방문은 자제하기

○ 불가피하게 방문하는 보호자・외부인은 증상 여부(발열, 호흡기 증상 등) 확인 및 명부(전자 또는 수기) 작성 관리(4주 보관 후 폐기) 등 방역에 협조하기

○ 발열, 호흡기 증상이 나타나면 방역관리자에게 알리고, 공용공간 이동은 최대한 자제하기(다인실 내, 개인실 밖에서는 마스크 착용)

○ 공용공간(조리실, 세탁실, 화장실 등)은 혼잡하지 않도록 이용 시간을 분산하여 이용하고 머무르는 시간 최소화하기

○ 방 밖을 이동하는 경우 다른 사람과 2m(최소 1m) 이상 거리를 두고, 거리 유지가 안되는 경우 마스크 착용하기

○ 공용공간에서 음식 섭취, 대화, 전화 등 비말 발생이 가능한 행위 자제하기

○ 공용 물품(식기류, 수건 등)은 공유하지 않고 개인 물품 사용하기

○ 자연 환기가 가능한 경우 창문을 상시 열어두고, 환기가 어려운 경우 매일 2회 이상 주기적으로 환기하기(방, 복도, 주방 등 공용공간 포함)

○ 고시원 내 이동(지인 방 방문 등) 자제하기

※ 고시원 내 공중화장실 이용 시 해당 유형의 지침을 준용

2 시설 책임자·관리자

[공통사항]

- ○ 방역을 관리하는 담당부서(관리자)를 지정하고 지역 보건소 담당자의 연락망을 확보하는 등 방역 협력체계 구축하기
- ○ 공동체 내 밀접 접촉이 일어나는 동일 부서, 동일 장소 등에 2~3명 이상의 유증상자가 3~4일 내에 발생 시 유증상자가 코로나19 검사를 받도록 안내하며, 유증상자가 추가 발생 시 보건소에 집단감염 가능성을 신고하기
- ○ 종사자가 발열 또는 호흡기 증상이 있는 경우 출근 중단 및 즉시 퇴근 조치하기
- ○ 사람 간 간격을 2m(최소 1m) 이상 거리 두기
- ○ 손을 씻을 수 있는 시설 또는 손 소독제를 비치하고, 손 씻기 및 기침예절 준수 안내문 게시하기
- ○ 자연 환기가 가능한 경우 창문을 상시 열어두고, 에어컨 사용 등으로 상시적으로 창문을 열어두기 어려운 경우 2시간마다 1회 이상 환기하기(방, 복도, 주방 등 공용공간 포함)
- ○ 공용으로 사용하는 물건(출입구 손잡이 등) 및 표면은 매일 1회 이상 자주 소독하기
- ○ 고객(이용자)을 직접 응대하는 경우 마스크 착용하게 하기
- ○ 발열 또는 호흡기 증상이 있거나 최근 14일 이내 해외여행을 한 경우 방문 자제 안내하기
- ○ 실내 다중이용시설(공용공간)을 이용하는 경우 마스크 착용 안내하기
- ○ 실외에서 2m 거리 유지가 안되는 경우 마스크 착용 안내하기

[해당 유형 적용사항]

- ○ 방역 지침(체온 측정, 마스크 착용, 거리 유지, 손 위생, 기침예절) 준수 안내하기
- ○ 고시원 이용자 외 보호자·외부인 등 방문 제한하기
- ○ 불가피하게 방문하는 보호자ㆍ외부인이 있는 경우에는 증상 여부(발열, 호흡기 증상 등) 확인 및 이용자, 관리자 등의 명부(전자 또는 수기)를 작성 관리(4주 보관 후 폐기)하기
- ○ 발열, 호흡기 증상이 나타나면 방역관리자에게 알리고, 공용 공간 이동은 최대한 자제하도록 안내하기(다인실 내, 개인실 밖에서는 마스크 착용)
- ○ 공용공간(조리실, 세탁실, 화장실 등)은 혼잡하지 않도록 이용 시간 분산하여 이용하고 머무르는 시간 최소화하도록 안내하기

○ 공용공간에서 음식 섭취, 대화, 전화 등 비말 발생이 가능한 행위 자제 안내하기

○ 공용공간은 매일 1회 이상 소독하되, 손이 자주 닿는 표면*은 더 자주 소독하기

* 자주 사용하는 모든 부위, 출입문 손잡이, 승강기 버튼, 스위치, 세탁기 표면, 화장실 변기, 수도꼭지, 책상, 의자 등

○ 시설 내 곳곳에 휴지와 뚜껑이 있는 쓰레기통, 손 소독제를 비치하고, 기침이나 재채기 후 사용한 휴지를 깨끗이 버리고 손 소독을 할 수 있도록 조치하기

※ 고시원 내 공중화장실 등이 있는 경우 해당 유형의 지침을 준용(소독 등 철저)

※ 소독은 집단시설·다중이용시설 소독 안내[3-3판] 지침을 준용

3-3. 생활 속 거리 두기 지침 : 연수시설

1 이용자

[공통사항]

○ 발열 또는 호흡기 증상(기침, 인후통 등)이 있거나 최근 14일 이내 해외여행을 한 경우 이용을 자제하기

○ 다른 사람과 2m(최소 1m) 이상 거리 두기

○ 흐르는 물과 비누로 30초 이상 손을 씻거나 손 소독제로 손 소독하기

○ 기침이나 재채기를 할 때는 휴지, 옷소매로 입과 코 가리기

○ 침방울이 튀는 행위(노래부르기, 구호외치기 등)나 신체접촉(악수, 포옹 등) 자제하기

○ 실내 다중이용시설을 이용하는 경우 마스크 착용하기

○ 실외에서 2m 거리 유지가 안되는 경우 마스크 착용하기

[해당 유형 적용사항]

○ 가급적 온라인 연수를 이용하기

○ 불가피하게 오프라인 연수에 참여할 경우 다른 사람과 거리 두기, 마스크 착용, 손 위생 등 개인위생수칙 준수하기

○ 셔틀버스 이용 시는 마스크를 착용하고, 다른 사람과 2m(최소1m)이상 거리 유지가 되도록 한 칸씩 띄어 앉고 대화 자제하기

○ 연수 중 발열, 호흡기 증상이 나타나면 연수 책임자에게 즉시 알리고 귀가하기

○ 실내 휴게실, 매점 등 다중이용공간 이용 시 밀집되지 않도록 분산하여 이용하기

○ 강의 전·후에 악수 등 다른 사람과 신체적 접촉 자제하기

○ 강의시간 및 공용 공간 이용 시 마스크 착용하기

○ 구내식당 이용 시 가급적 일렬 또는 지그재그로 띄어 앉고 대화 자제하기

○ 타인과 물품은 공유하지 않고 개인물품 사용하기

○ 불필요한 외출 및 교육시간 외의 소모임 등 자제하기

○ 매일 증상 여부(발열, 호흡기 증상 등) 확인 등 방역에 협조하기

○ 연수시설 이용자 외 외부인 등 방문은 자제하기

※ 시설 내 음식점·카페, 실내체육시설, 공중화장실 등 이용 시 해당 유형의 지침을 준용

2 책임자·관리자

[공통사항]

○ 방역을 관리하는 담당부서(관리자)를 지정하고 지역 보건소 담당자의 연락망을 확보하는 등 방역 협력체계 구축하기

○ 공동체 내 밀접 접촉이 일어나는 동일 부서, 동일 장소 등에 2~3명 이상의 유증상자가 3~4일 내에 발생 시 유증상자가 코로나19 검사를 받도록 안내하며, 유증상자가 추가 발생 시 보건소에 집단감염 가능성을 신고하기

○ 종사자가 발열 또는 호흡기 증상이 있는 경우 출근 중단 및 즉시 퇴근 조치하기

○ 사람 간 간격을 2m(최소 1m) 이상 거리 두기

○ 손을 씻을 수 있는 시설 또는 손 소독제를 비치하고, 손 씻기 및 기침예절 준수 안내문 게시하기

○ 자연 환기가 가능한 경우 창문을 상시 열어두고, 에어컨 사용 등으로 상시적으로 창문을 열어두기 어려운 경우 2시간마다 1회 이상 환기하기

○ 공용으로 사용하는 물건(출입구 손잡이 등) 및 표면은 매일 1회 이상 자주 소독하기

○ 고객(이용자)을 직접 응대하는 경우 마스크 착용하게 하기

○ 발열 또는 호흡기 증상이 있거나 최근 14일 이내 해외여행을 한 경우 방문 자제 안내하기

○ 실내 다중이용시설을 이용하는 경우 마스크 착용 안내하기

○ 실외에서 2m 거리 유지가 안되는 경우 마스크 착용 안내하기

[해당 유형 적용사항]

○ 온라인 연수를 활용하기

○ 불가피하게 오프라인 연수를 개최할 경우, 가급적 운영과정에서 소규모 단위로 분반, 편성하고 숙박 없이 하루만 집합교육 실시하기

○ 오프라인 연수 시, 방역수칙(체온측정, 마스크 착용, 손위생, 소독용품 비치 등) 준수하여 진행하기

○ 교육장 규모와 2m(최소 1m) 거리 두기를 감안하여 연수인원을 제한하기

○ 숙박은 가급적 자제하되, 필요한 경우 1인 1실을 배정하고, 다인실의 경우 침대간 거리를 충분히 확보하는 등 거리 두기를 유지할 수 있도록 인원 배정하기

○ 연수 중 발열, 호흡기 증상이 나타난 연수생은 귀가 조치하기

○ 한 방향으로 일자형 테이블을 배치하고, 좌석은 가급적 2m(최소 1m) 이상 거리를 둘 수 있도록 한 칸 띄어 지정좌석제로 운영하기

○ 강의시간에 강사와 교육생은 마스크를 계속 쓰고, 토론·발표와 같이 말을 해야 하는 경우에도 마스크를 쓰도록 안내하기

- 다만, 머리가 아프거나 숨이 차는 등 이상증세가 있는 경우 즉시 마스크를 벗도록 하며, 이상증세가 길어지는 경우 연수에 참석하지 않고 휴식하도록 하기

○ 마이크는 커버를 씌우고, 개인마다 마이크 덮개를 새것으로 교체하도록 안내하기

○ 집단 레크레이션 등 밀집한 공간에서 다중이 참여하는 프로그램 자제하기

○ 식사 시간은 시차를 두고 운영하는 등 이용자 간 밀집을 최소화하기

(예: A조/11:30~12:30, B조/12:30~13:30)

○ 구내식당 좌석 간 투명 가림막을 설치하거나 가급적 일렬 또는 지그재그로 앉게 하기

○ 연수시설 이용자 외 외부인 등 방문 제한하기

○ 연수시설 이용자에 대해 매일 증상 여부(발열, 호흡기 증상 등) 확인하기

※ 시설 내 음식점·카페, 실내체육시설, 공중화장실 등 이용 시 해당 유형의 지침을 준용

3-4. 생활 속 거리 두기 지침 : 학술행사

1 참석자

[공통사항]

○ 발열 또는 호흡기 증상(기침, 인후통 등)이 있거나 최근 14일 이내 해외여행을 한 경우 참석을 자제하기

○ 다른 사람과 2m(최소 1m) 이상 거리 두기

○ 흐르는 물과 비누로 30초 이상 손을 씻거나 손 소독제로 손 소독하기

○ 기침이나 재채기를 할 때는 휴지, 옷소매로 입과 코 가리기

○ 침방울이 튀는 행위(노래부르기, 구호외치기 등)나 신체접촉(악수, 포옹 등) 자제하기

○ 실내 다중이용시설을 이용하는 경우 마스크 착용하기

○ 실외에서 2m 거리 유지가 안되는 경우 마스크 착용하기

[해당 유형 적용사항]

○ 학술행사는 가급적 온라인으로 참여하고, 오프라인 행사 참여는 자제하기

○ 오프라인(현장) 학술행사에 참석할 경우에는 사전등록을 하고 현장 등록은 자제하기

○ 오프라인 학술행사 참석 일정(시간)은 최소화하기

○ 학술행사장 내에서는 마스크를 착용하고, 음식 섭취를 자제하기

○ 줄서기 시 다른 사람과의 간격을 2m(최소 1m) 이상 유지하기

○ 좌석에 앉을 때는 지그재그로 한 칸 띄어 앉기

○ 타인과 물품은 공유하지 않고(책자, 펜, 티스푼 등) 개인 물품 사용하기

○ 식사 시간에는 서로 마주 보지 않고 한 방향을 바라보거나 지그재그로 앉고 대화 자제하기

○ 실내 휴게실, 카페, 매점 등 다중이용공간 밀집되지 않도록 분산하여 이용하기

○ 소규모 모임, 회식 등은 자제하고, 종료 후 일찍 귀가하기

○ 출입 시 증상 여부(발열, 호흡기 증상 등) 확인 등 방역에 협조하기

○ 학술행사 중 발열, 호흡기 증상이 나타나면 행사 관계자에게 즉시 알리고 귀가하기

※ 학술행사장 내에 회의 진행, 음식점·카페, 공중화장실 등이 있는 경우 해당 유형의 지침을 준용

2 책임자·관리자

[공통사항]

○ 방역을 관리하는 담당부서(관리자)를 지정하고 지역 보건소 담당자의 연락망을 확보하는 등 방역 협력체계 구축하기

○ 공동체 내 밀접 접촉이 일어나는 동일 부서, 동일 장소 등에 2~3명 이상의 유증상자가 3~4일 내에 발생 시 유증상자가 코로나19 검사를 받도록 안내하며, 유증상자가 추가 발생 시 보건소에 집단감염 가능성을 신고하기

○ 종사자가 발열 또는 호흡기 증상이 있는 경우 출근 중단 및 즉시 퇴근 조치하기

○ 사람 간 간격을 2m(최소 1m) 이상 거리 두기

○ 손을 씻을 수 있는 시설 또는 손 소독제를 비치하고, 손 씻기 및 기침예절 준수 안내문 게시하기

○ 자연 환기가 가능한 경우 창문을 상시 열어두고, 에어컨 사용 등으로 상시적으로 창문을 열어두기 어려운 경우 2시간마다 1회 이상 환기하기

○ 공용으로 사용하는 물건(출입구 손잡이 등) 및 표면은 매일 1회 이상 자주 소독하기

○ 고객(이용자)을 직접 응대하는 경우 마스크 착용하게 하기

○ 발열 또는 호흡기 증상이 있거나 최근 14일 이내 해외여행을 한 경우 방문 자제 안내하기

○ 실내 다중이용시설을 이용하는 경우 마스크 착용 안내하기

○ 실외에서 2m 거리 유지가 안되는 경우 마스크 착용 안내하기

[해당 유형 적용사항]

○ 학술행사는 가급적 온라인으로 개최하며 오프라인 행사는 자제하되, 불가피한 경우 온라인과 병행하여 밀집도를 최소화하기

○ 오프라인(현장) 학술행사를 개최할 경우, 사전등록을 독려하고 현장등록은 최소화하여 혼잡하지 않도록 분산시키기

○ 오프라인 학술행사는 참석 일정(반나절, 1일), 시간을 최소화하고 소규모로 진행하기

○ 학술행사 장소 규모와 거리 두기(2m)를 감안하여 참석 인원을 제한하기

○ 사전에 발열, 호흡기 증상(인후통, 기침, 호흡곤란, 권태감, 두통, 근육통 등) 등이 있거나 14일 이내 해외 여행력이 있으면 학술행사 참여하지 말 것을 공지하기

○ 해외거주 발제자 국내 초청은 가급적 자제하되, 필요시 출입국 방역절차 확인하기

○ 해외발제자는 영상 참여 활용하도록 안내하기

○ 학술행사 시작 전·후 및 수시로 손 씻기, 손 소독제 사용, 기침 예절 등 개인 위생관리 사항 안내 및 협조 요청하기

  * 질병관리본부 홈페이지(www.kcdc.go.kr)에 게시된 홍보자료 부착 활용

○ 한 방향으로 일자형 테이블을 배치하고, 좌석은 가급적 2m(최소 1m) 이상 거리를 둘 수 있도록 한 칸 띄어 지정좌석제로 운영하기

○ 마이크를 사용하는 경우 마이크는 커버를 씌우고, 개인마다 마이크 덮개를 새것으로 교체하기

○ 단체식사 제공은 자제하며, 불가피한 경우에는 개인 도시락 형태로 제공하기

○ 1시간 주기로 휴식을 가지며 행사장 출입문과 창문을 열고 환기하기

○ 밀집한 공간에서 다중이 참여하는 프로그램, 부대행사 등 자제하기

○ 기관, 후원사 홍보 부스 운영은 자제하되, 운영 시에는 부스 간에 2m 이상 간격을 유지하고, 참석자들이 한꺼번에 모이지 않도록 안내하기

○ 출입구 및 시설 내 곳곳에 손 소독제와 뚜껑 있는 쓰레기통 비치하기

○ 출입하는 사람에 대한 증상 여부(발열, 호흡기 증상 등) 확인하기

○ 숙박은 가급적 자제하되, 필요한 경우 1인 1실을 배정하고, 다인실의 경우 침대간 거리를 충분히 확보하는 등 거리 두기를 유지할 수 있도록 인원 배정하기

○ 발열, 호흡기 증상이 있는 참자가가 발견되면 마스크 착용과 귀가하도록 안내하기

※ 학술행사장 내에 회의 진행, 음식점·카페, 공중화장실 등이 있는 경우 해당 유형의 지침을 준용

Ⅱ. 일 상 (4. 쇼핑할 때)

4-1. 생활 속 거리 두기 지침 : 대형유통시설

(백화점 · 대형마트 · 복합쇼핑몰 · 기업형슈퍼마켓 · 아울렛 등)

1 이용자

[공통사항]

- ○ 발열 또는 호흡기 증상(기침, 인후통 등)이 있거나 최근 14일 이내 해외여행을 한 경우 방문 자제하기
- ○ 다른 사람과 2m(최소 1m) 이상 거리 두기
- ○ 흐르는 물과 비누로 30초 이상 손을 씻거나 손 소독제로 손 소독하기
- ○ 기침이나 재채기를 할 때는 휴지, 옷소매로 입과 코 가리기
- ○ 침방울이 튀는 행위(노래부르기, 소리지르기 등)나 신체접촉(악수, 포옹 등) 자제하기
- ○ 실내 다중이용시설을 이용하는 경우 마스크 착용하기
- ○ 실외에서 2m 거리 유지가 안되는 경우 마스크 착용하기

[해당 유형 적용사항]

- ○ 물건을 고르거나 계산 줄에 서 있는 동안 다른 방문객과 2m(최소 1m) 이상의 거리 유지하기
- ○ 최소 인원으로 쇼핑하기
- ○ 공용 쇼핑카트, 장바구니를 이용하기 전 표면을 소독하거나, 손 소독제를 사용하기
- ○ 화장품 견본품 얼굴이나 입술에 직접 사용 자제(손등 테스트 등으로 대체하고, 테스트 후 손 소독 또는 손 씻기)하기
- ○ 계산 시 가능한 전자 결제방식 이용(모바일페이, QR코드, NFC카드, 신용카드 등)하기

※ 시설 내 음식점·카페 등 이용 시 해당 유형의 지침을 준용

2 책임자·종사자

[공통사항]

- ○ 방역관리자 지정 및 지역 보건소 담당자의 연락망을 확보하는 등 방역 협력체계 구축하기
- ○ 공동체 내 밀접 접촉이 일어나는 동일 부서, 동일 장소 등에 2~3명 이상의

유증상자가 3~4일 내에 발생 시 유증상자가 코로나19 검사를 받도록 안내하며, 유증상자가 추가 발생 시 보건소에 집단감염 가능성을 신고하기

○ 종사자가 발열 또는 호흡기 증상이 있는 경우 출근 중단 및 즉시 퇴근 조치하기

○ 사람 간 간격을 2m(최소 1m) 이상 거리 두기

○ 손을 씻을 수 있는 시설 또는 손 소독제를 비치하고, 손 씻기 및 기침예절 준수 안내문 게시하기

○ 자연 환기가 가능한 경우 창문을 상시 열어두고, 에어컨 사용 등으로 상시적으로 창문을 열어두기 어려운 경우 2시간마다 1회 이상 환기하기

○ 공용으로 사용하는 물건(출입구 손잡이 등) 및 표면은 매일 1회 이상 자주 소독하기

○ 고객(이용자)을 직접 응대하는 경우 마스크 착용하기

○ 발열 또는 호흡기 증상이 있거나 최근 14일 이내 해외여행을 한 경우 방문 자제 안내하기

○ 실내 다중이용시설을 이용하는 경우 마스크 착용 안내하기

○ 실외에서 2m 거리 유지가 안되는 경우 마스크 착용 안내하기

[해당 유형 적용사항]

○ 종사자의 유연근무 및 휴가를 가급적 자유롭게 사용할 수 있도록 조치하고, 가능한 한도 내에서 대체 인력 확보하기

○ 많은 이용객이 일시에 한 장소에 집중될 수 있는 이벤트성 행사를 자제(예: 선착순, 악수·사인회 등)하기

- 부득이하게 집객행사 등을 실시하는 경우 이용객을 분산시킬 수 있는 방안 마련

○ 큰 소리로 호객행위를 하는 등 비말이 튈 수 있는 행위를 자제하고, 안내방송 및 리플릿 등으로 대체하기

○ 시식 및 화장품 테스트 코너 운영을 중단하거나 최소화하기

- 시식·화장품 테스트 코너에서 발생하는 이쑤시개, 컵, 휴지, 솜 등 침이 묻을 수 있는 쓰레기는 타인의 손이 닿지 않도록 별도로 깨끗이 버릴 수 있게 조치하기

○ 입장, 계산 등 대기열에서 이용객 간 간격을 2m(최소 1m) 이상 유지할 수 있도록 안내하기

- 바닥 스티커, 안내문 등을 통해 거리를 유지할 수 있도록 안내하기
- 2m 이상 거리 두기가 어려운 경우 마스크 착용하고 최소 1m 거리 유지하기 안내하기

○ 물건을 고르는 고객 등을 따라다니지 않도록 직원 안내하기

○ 계산원, 접객 직원과 이용객 사이 2m(최소 1m) 이상 거리를 유지할 수 있도록 하고, 필요 시 투명 가림막 등을 설치하기

○ 공용 쇼핑카트, 장바구니 근처에 손 소독제를 배치하고 손잡이 수시 소독하기

○ 계산 시 가능한 전자·비접촉 결제방식 사용을 권장하기

○ 문화센터, 어린이 놀이시설 등 공용시설 운영을 최소화하고, 운영하는 경우 이용자들 간에 거리 유지가 이루어질 수 있도록 조치하기

※ 시설 내 음식점·카페 등 이용 시 해당 유형의 지침을 준용

4-2. 생활 속 거리 두기 지침 : 전통시장

1 이용자·방문객

[공통사항]

○ 발열 또는 호흡기 증상(기침, 인후통 등)이 있거나 최근 14일 이내 해외여행을 한 경우 방문 자제하기

○ 다른 사람과 2m(최소 1m) 이상 거리 두기

○ 흐르는 물과 비누로 30초 이상 손을 씻거나 손 소독제로 손 소독하기

○ 기침이나 재채기를 할 때는 휴지, 옷소매로 입과 코 가리기

○ 침방울이 튀는 행위(노래부르기, 소리지르기 등)나 신체접촉(악수, 포옹 등) 자제하기

○ 실내 다중이용시설을 이용하는 경우 마스크 착용하기

○ 실외에서 2m 거리 유지가 안되는 경우 마스크 착용하기

※ 시설 내 음식점·카페 등 이용 시 해당 유형의 지침을 준용

2 책임자·종사자

[공통사항]

○ 방역관리자 지정 및 지역 보건소 담당자의 연락망을 확보하는 등 방역 협력체계 구축하기

○ 공동체 내 밀접 접촉이 일어나는 동일 부서, 동일 장소 등에 2~3명 이상의 유증상자가 3~4일 내에 발생 시 유증상자가 코로나19 검사를 받도록 안내하며, 유증상자가 추가 발생 시 보건소에 집단감염 가능성을 신고하기

○ 종사자가 발열 또는 호흡기 증상이 있는 경우 출근 중단 및 즉시 퇴근 조치하기

○ 사람 간 간격을 2m(최소 1m) 이상 거리 두기

○ 손을 씻을 수 있는 시설 또는 손 소독제를 비치하고, 손 씻기 및 기침예절 준수 안내문 게시하기

○ 자연 환기가 가능한 경우 창문을 상시 열어두고, 에어컨 사용 등으로 상시적으로 창문을 열어두기 어려운 경우 2시간마다 1회 이상 환기하기

○ 공용으로 사용하는 물건(출입구 손잡이 등) 및 표면은 매일 1회 이상 자주 소독하기

○ 고객(이용자)을 직접 응대하는 경우 마스크 착용하기

○ 발열 또는 호흡기 증상이 있거나 최근 14일 이내 해외여행을 한 경우 방문 자제 안내하기

○ 실내 다중이용시설을 이용하는 경우 마스크 착용 안내하기

○ 실외에서 2m 거리 유지가 안되는 경우 마스크 착용 안내하기

[해당 유형 적용사항]

○ 침방울이 튀는 행위(큰 소리로 호객 행위를 하는 등) 자제하기

○ 집객 행사를 가급적 자제하고, 부득이하게 실시하는 경우 이용객 분산 유도하기

※ 시설 내 음식점·카페 등 이용 시 해당 유형의 지침을 준용

4-3. 생활 속 거리 두기 지침 : 중소슈퍼

1 이용자

[공통사항]

○ 발열 또는 호흡기 증상(기침, 인후통 등)이 있거나 최근 14일 이내 해외여행을 한 경우 방문 자제하기

○ 다른 사람과 2m(최소 1m) 이상 거리 두기

○ 흐르는 물과 비누로 30초 이상 손을 씻거나 손 소독제로 손 소독하기

○ 기침이나 재채기를 할 때는 휴지, 옷소매로 입과 코 가리기

○ 침방울이 튀는 행위(노래부르기, 소리지르기 등)나 신체접촉(악수, 포옹 등) 자제하기

○ 실내 다중이용시설을 이용하는 경우 마스크 착용하기

○ 실외에서 2m 거리 유지가 안되는 경우 마스크 착용하기

[해당 유형 적용사항]

○ 상점 내 머무르는 시간 최소화하기

2 책임자·종사자

[공통사항]

○ 방역관리자 지정 및 지역 보건소 담당자의 연락망을 확보하는 등 방역 협력체계 구축하기

○ 공동체 내 밀접 접촉이 일어나는 동일 부서, 동일 장소 등에 2~3명 이상의 유증상자가 3~4일 내에 발생 시 유증상자가 코로나19 검사를 받도록 안내하며, 유증상자가 추가 발생 시 보건소에 집단감염 가능성을 신고하기

○ 종사자가 발열 또는 호흡기 증상이 있는 경우 출근 중단 및 즉시 퇴근 조치하기

○ 사람 간 간격을 2m(최소 1m) 이상 거리 두기

○ 손을 씻을 수 있는 시설 또는 손 소독제를 비치하고, 손 씻기 및 기침예절 준수 안내문 게시하기

○ 자연 환기가 가능한 경우 창문을 상시 열어두고, 에어컨 사용 등으로 상시적으로 창문을 열어두기 어려운 경우 2시간마다 1회 이상 환기하기

○ 공용으로 사용하는 물건(출입구 손잡이 등) 및 표면은 매일 1회 이상 자주 소독하기

○ 고객(이용자)을 직접 응대하는 경우 마스크 착용하기

○ 발열 또는 호흡기 증상이 있거나 최근 14일 이내 해외여행을 한 경우 방문 자제 안내하기

○ 실내 다중이용시설을 이용하는 경우 마스크 착용 안내하기

○ 실외에서 2m 거리 유지가 안되는 경우 마스크 착용 안내하기

[해당 유형 적용사항]

○ 계산대 등 줄을 서는 곳에는 2m(최소 1m) 이상 간격을 둘 수 있도록 표시하기

Ⅱ. 일 상 (5. 특별한 날)

5-1. 생활 속 거리 두기 지침 : 결혼식 등 가족 행사

1 방문객

[공통사항]

- ○ 발열 또는 호흡기 증상(기침, 인후통 등)이 있거나 최근 14일 이내 해외여행을 한 경우 행사 참석을 자제하고, 다른 방법으로 마음 전하기
- ○ 다른 사람과 2m(최소 1m) 이상 거리 두기
- ○ 흐르는 물과 비누로 30초 이상 손을 씻거나 손 소독제로 손 소독하기
- ○ 기침이나 재채기를 할 때는 휴지, 옷소매로 입과 코 가리기
- ○ 침방울이 튀는 행위(노래부르기, 소리지르기 등)나 신체접촉(악수, 포옹 등) 자제하기
- ○ 실내 다중이용시설을 이용하는 경우 마스크 착용하기
- ○ 실외에서 2m 거리 유지가 안되는 경우 마스크 착용하기

[해당 유형 적용사항]

- ○ 행사 시 탁자 사이를 가급적 2m(최소 1m) 이상 간격을 두기
- ○ 식사시간에 가능한 서로 마주보지 않고 한 방향을 바라보거나 지그재그로 앉기
- ○ 식사를 할 경우 음식은 각자 개인 접시에 덜어 먹기
- ○ 축의금은 가급적 온라인으로 하기
- ○ 가급적 악수보다 목례로 인사하기
- ○ 출입 시 증상 여부(발열, 호흡기 증상 등) 확인 및 명부(전자 또는 수기) 기록 관리 (4주 보관 후 폐기) 등 방역에 협조하기

2 행사주관자

[공통사항]

- ○ 발열 또는 호흡기 증상이 있거나 최근 14일 이내 해외여행을 한 경우 참석 자제 안내하기
- ○ 참석자 간 간격을 2m(최소 1m) 이상 거리 두기 안내하기
- ○ 실내 다중이용시설을 이용하는 경우 마스크 착용 안내하기
- ○ 실외에서 2m 거리 유지가 안되는 경우 마스크 착용 안내하기

[해당 유형 적용사항]

○ 가족 행사는 가급적 검소(간소)하게 준비하거나, 필요시 온라인으로 진행하기

○ 행사장 규모를 고려하여 밀집하지 않도록 초청 인원 수 정하기

○ 발열, 호흡기 증상이 있거나 최근 14일 이내 해외여행을 한 경우 행사 연기하기

○ 초청자에게 생활 속 거리 두기 지침을 사전에 충분히 안내하기

○ 생활 속 거리 두기 지침을 우선적으로 고려하여 행사 진행하기

○ 악수는 자제하고, 식사보다 답례품을 제공하기

3 책임자·종사자

[공통사항]

○ 방역관리자 지정 및 지역 보건소 담당자의 연락망을 확보하는 등 방역 협력체계 구축하기

○ 공동체 내 밀접 접촉이 일어나는 동일 부서, 동일 장소 등에 2~3명 이상의 유증상자가 3~4일 내에 발생 시 유증상자가 코로나19 검사를 받도록 안내하며, 유증상자가 추가 발생 시 보건소에 집단감염 가능성을 신고하기

○ 종사자가 발열 또는 호흡기 증상이 있는 경우 출근 중단 및 즉시 퇴근 조치하기

○ 사람 간 간격을 2m(최소 1m) 이상 거리 두기

○ 손을 씻을 수 있는 시설 또는 손 소독제를 비치하고, 손 씻기 및 기침예절 준수 안내문 게시하기

○ 자연 환기가 가능한 경우 창문을 상시 열어두고, 에어컨 사용 등으로 상시적으로 창문을 열어두기 어려운 경우 2시간마다 1회 이상 환기하기

○ 공용으로 사용하는 물건(출입구 손잡이 등) 및 표면은 매일 1회 이상 자주 소독하기

○ 고객(이용자)을 직접 응대하는 경우 마스크 착용하기

○ 발열 또는 호흡기 증상이 있거나 최근 14일 이내 해외여행을 한 경우 방문 자제 안내하기

○ 실내 다중이용시설을 이용하는 경우 마스크 착용 안내하기

○ 실외에서 2m 거리 유지가 안되는 경우 마스크 착용 안내하기

[해당 유형 적용사항]

○ 행사 시 탁자 사이 간격을 가급적 2m(최소 1m) 이상 두거나 고정형 탁자는 일부를 사용하지 않는 등 최소 1m 거리 유지하기

○ 가능한 의자 배치는 서로 마주 보지 않고 한 방향을 바라보거나 지그재그로 배치하기

○ 행사 시간은 가능한 간격을 충분히 두고 진행하기

○ 직원들에게 개인위생수칙 준수, 생활 속 거리 두기의 필요성 등에 대한 주기적으로 교육 실시하기

○ 음식은 각자 개인 접시에 덜어 먹도록 개인 접시와 국자, 집게 등을 제공하기

○ 출입하는 사람에 대한 증상 여부(발열, 호흡기 증상 등) 확인 및 명부(전자 또는 수기)를 작성 관리(4주 보관 후 폐기)하기

5-2. 생활 속 거리 두기 지침 : 장례식장

1 조문객·유족

[공통사항]

○ 발열 또는 호흡기 증상(기침, 인후통 등)이 있거나 최근 14일 이내 해외여행을 한 경우 직접 조문을 자제하고 다른 방법으로 마음 전하기

○ 다른 사람과 2m(최소 1m) 이상 거리 두기

○ 흐르는 물과 비누로 30초 이상 손을 씻거나 손 소독제로 손 소독하기

○ 기침이나 재채기를 할 때는 휴지, 옷소매로 입과 코 가리기

○ 침방울이 튀는 행위(노래부르기, 소리지르기 등)나 신체접촉(악수, 포옹 등) 자제하기

○ 실내 다중이용시설을 이용하는 경우 마스크 착용하기

○ 실외에서 2m 거리 유지가 안되는 경우 마스크 착용하기

[해당 유형 적용사항]

○ 조문객을 맞이할 때 마스크를 착용하고, 악수보다는 목례로 인사하기

○ 빈소에서 식사를 하는 경우 서로 마주보지 않고 한 방향을 보거나 지그재그로 식사하기

○ 조문시 가급적 악수보다는 고개 숙여 위로의 마음을 표하기

○ 조문과 위로는 가급적 간략하게 하고, 30분 이상 머물지 않도록 권장하기

○ 가족 중심의 간소한 장례를 치르고, 입관 및 발인식 등 장례절차 진행시 최소 인원이 참여하며, 참여자간 1m 거리의 간격 유지하기

○ 출입 시 증상 여부(발열, 호흡기 증상 등) 확인 및 명부(전자 또는 수기) 기록 관리 (4주 보관 후 폐기) 등 방역에 협조하기

2 책임자·종사자

[공통사항]

○ 방역관리자 지정 및 지역 보건소 담당자의 연락망을 확보하는 등 방역 협력체계 구축하기

○ 공동체 내 밀접 접촉이 일어나는 동일 부서, 동일 장소 등에 2~3명 이상의 유증상자가 3~4일 내에 발생 시 유증상자가 코로나19 검사를 받도록 안내하며,

유증상자가 추가 발생 시 보건소에 집단감염 가능성을 신고하기

○ 종사자가 발열 또는 호흡기 증상이 있는 경우 출근 중단 및 즉시 퇴근 조치하기
○ 사람 간 간격을 2m(최소 1m) 이상 거리 두기
○ 손을 씻을 수 있는 시설 또는 손 소독제를 비치하고, 손 씻기 및 기침예절 준수 안내문 게시하기
○ 자연 환기가 가능한 경우 창문을 상시 열어두고, 에어컨 사용 등으로 상시적으로 창문을 열어두기 어려운 경우 2시간마다 1회 이상 환기하기
○ 공용으로 사용하는 물건(출입구 손잡이 등) 및 표면은 매일 1회 이상 자주 소독하기
○ 고객(이용자)을 직접 응대하는 경우 마스크 착용하기
○ 발열 또는 호흡기 증상이 있거나 최근 14일 이내 해외여행을 한 경우 방문 자제 안내하기
○ 실내 다중이용시설을 이용하는 경우 마스크 착용 안내하기
○ 실외에서 2m 거리 유지가 안되는 경우 마스크 착용 안내하기

[해당 유형 적용사항]

○ 탁자 사이 간격을 가급적 2m(최소 1m) 이상 두거나 고정형 탁자는 일부를 사용하지 않는 등 거리 유지하기
○ 참관실 및 발인실 등 앞 뒤 사용 시간을 일정 시차를 두고 사용하기
○ 입관 및 발인식에 가능한 최소 인원이 참여할 수 있도록 안내하기
○ 장례식장 직원, 상조회사 및 장례용품을 공급하는 외부 사람들이 개인위생수칙(손 소독과 마스크 착용 등) 준수하도록 안내하기
○ 음식은 각자 개인 접시에 덜어 먹도록 개인접시와 국자, 집게 등을 제공하기
○ 사용하지 않는 빈소는 1일 1시간 이상 2회~3회 환기장치 가동하기
○ 염습실, 참관실 및 발인실 사용 직후 소독하기
○ 운구차량을 운영할 경우 이용 후 소독하기
○ 출입하는 사람에 대한 증상 여부(발열, 호흡기 증상 등) 확인 및 명부(전자 또는 수기)를 작성 관리(4주 보관 후 폐기)하기

5-3. 생활 속 거리 두기 지침 : 산후조리원

1 이용자

[공통사항]

○ 발열 또는 호흡기 증상(기침, 인후통 등)이 있거나 최근 14일 이내 해외여행을 한 경우 방문 자제하기

○ 다른 사람과 2m(최소 1m) 이상 거리 두기

○ 흐르는 물과 비누로 30초 이상 손을 씻거나 손 소독제로 손 소독하기

○ 기침이나 재채기를 할 때는 휴지, 옷소매로 입과 코 가리기

○ 침방울이 튀는 행위(노래부르기, 소리지르기 등)나 신체접촉(악수, 포옹 등) 자제하기

○ 실내 다중이용시설을 이용하는 경우 마스크 착용하기

○ 실외에서 2m 거리 유지가 안되는 경우 마스크 착용하기

[해당 유형 적용사항]

○ 이용 시 증상 여부(발열, 호흡기 증상 등) 확인 등 방역에 협조하기

○ 영유아가 새로 입실하는 경우 적어도 입실 당일에는 사전관찰실에서 관찰 또는 모자동실하기

○ 산모 개인물품(개인 물병, 식기류 등) 사용하기

○ 공동으로 수유실을 사용하는 경우 이용 시간 간격 두어 1인씩 이용하기

○ 식당 이용 시 개별로 식사하며, 가급적 일렬 또는 지그재그로 앉고 대화는 자제하기

○ 진료 목적의 의료기관 방문 등을 제외한 외출을 자제하기

- 부득이하게 외출하는 경우 외출 일시·장소·사유 등을 기록하기

○ 1일 2회 이상 주기적으로 산모실 환기하기

○ 면회를 최소화하여 배우자 등 지정된 사람만 하기

2 책임자·종사자

[공통사항]

- ○ 방역관리자 지정 및 지역 보건소 담당자의 연락망을 확보하는 등 방역 협력체계 구축하기
- ○ 공동체 내 밀접 접촉이 일어나는 동일 부서, 동일 장소 등에 2~3명 이상의 유증상자가 3~4일 내에 발생 시 유증상자가 코로나19 검사를 받도록 안내하며, 유증상자가 추가 발생 시 보건소에 집단감염 가능성을 신고하기
- ○ 종사자가 발열 또는 호흡기 증상이 있는 경우 출근 중단 및 즉시 퇴근 조치하기
- ○ 사람 간 간격을 2m(최소 1m) 이상 거리 두기
- ○ 손을 씻을 수 있는 시설 또는 손 소독제를 비치하고, 손 씻기 및 기침예절 준수 안내문 게시하기
- ○ 자연 환기가 가능한 경우 창문을 상시 열어두고, 에어컨 사용 등으로 상시적으로 창문을 열어두기 어려운 경우 2시간마다 1회 이상 환기하기
- ○ 공용으로 사용하는 물건(출입구 손잡이 등) 및 표면은 매일 1회 이상 자주 소독하기
- ○ 고객(이용자)을 직접 응대하는 경우 마스크 착용하기
- ○ 발열 또는 호흡기 증상이 있거나 최근 14일 이내 해외여행을 한 경우 방문 자제 안내하기
- ○ 이용자에게 실내 다중이용시설을 이용하는 경우 마스크 착용 안내하기
- ○ 이용자에게 실외에서 2m 거리 유지가 안되는 경우 마스크 착용 안내하기

[해당 유형 적용사항]

□ 종사자 관리

- ○ 출입 시 사전 위생 확인 등을 위한 전담직원 배치하기
- ○ 근무 중 2회 이상 발열 및 호흡기 증상 확인하기

□ 신생아 및 산모 건강관리

- ○ 매일 1회 이상 산모 감염병 의심 증상 확인하기
- ○ 신생아실, 산모실 및 공용공간의 청결을 유지하고, 가급적 공용물품을 사용하지 않도록 안내하기
- ○ 산모 외출 후 발열 및 호흡기 증상 확인하기

□ **방문객 관리**

○ 면회를 최소화하여 배우자 등 지정된 사람만 하게 하기

○ 방문객 방문 시 증상 여부(발열, 호흡기 증상 등) 확인하기

○ 방문객 예방 교육 시행: 소독, 마스크(이후 분리 처리), 가운 착용 등 예방조치를 철저히 하기

○ 방문객 상담은 별도의 공간에서 실시하기

○ 불가피하게 산전 교육 진행시 별도의 공간에서 실시하기

□ **감염 확산 방지를 위한 관리**

○ 마스크 미착용 출입자 방문 제한하기

○ 식당 이용 시 개별로 식사하며, 가급적 일렬 또는 지그재그로 앉고 대화는 자제하기

○ 집단으로 이루어지는 교육 등의 서비스 제공은 불가피한 경우가 아니라면 자제하기

- 반드시 필요한 교육(모유 수유 및 신생아 관리 등)은 영상이나 서면 자료 활용 등의 비대면 교육으로 대체하기

○ 일 2회 이상 주기적 환기하기

○ 시설이용자, 종사자 및 기타 방문객을 대상으로 위생수칙 교육·홍보하기

○ 손 씻기, 기침 예절 등 위생수칙에 대한 각종 홍보물*을 시설 내 주요 장소에 부착하기

* 관련 홍보물은 질병관리본부 홈페이지(www.kcdc.go.kr)에 게시된 자료 활용

5-4. 생활 속 거리 두기 지침 : 기념식

※ 불가피하게 행사를 개최하는 경우, 「코로나19 집단행사 방역관리 지침(2판, '20.02,26)」 및 「코로나19 관련 정부·지자체 행사 운영지침(2판, '20.02.26)」 등에 따른 방역 조치를 반드시 준수하고, 그 외 세부사항은 본 세부지침을 준용

1 참석자

[공통사항]

- ○ 발열 또는 호흡기 증상(기침, 인후통 등)이 있거나 최근 14일 이내 해외여행을 한 경우 방문 자제하기
- ○ 다른 사람과 2m(최소 1m) 이상 거리 두기
- ○ 흐르는 물과 비누로 30초 이상 손을 씻거나 손 소독제로 손 소독하기
- ○ 기침이나 재채기를 할 때는 휴지, 옷소매로 입과 코 가리기
- ○ 침방울이 튀는 행위(노래부르기, 소리지르기 등)나 신체접촉(악수, 포옹 등) 자제하기
- ○ 실내 다중이용시설을 이용하는 경우 마스크 착용하기
- ○ 실외에서 2m 거리 유지가 안되는 경우 마스크 착용하기

[해당 유형 적용사항]

- ○ 가급적 온라인 또는 영상으로 참여하기
- ○ 불가피하게 현장 참여를 하는 경우 다른 사람과 2m(최소 1m) 이상 거리 두기, 마스크 착용, 손위생(물과 비누로 30초 이상 손 씻기, 손 소독제 사용) 등 개인 방역 수칙 준수하기
- ○ 고위험군(65세 이상 어르신, 임신부, 만성질환자 등)은 가급적 전시회 방문을 자제하기
- ○ 기념식 출입 시 증상 여부(발열, 호흡기 증상 등) 확인 및 및 명부(전자 또는 수기) 기록 관리(4주 보관 후 폐기) 등 방역에 협조하기
- ○ 기념식 객석, 휴게시설 이용 시 좌석은 2m(최소 1m) 거리 두기가 가능하도록 지그재그로 한 칸 띄워 앉고 대화는 자제하기
- ○ 실내 휴게시설, 카페, 매점 등 다중이용공간이 밀집되지 않도록 2m(최소 1m) 거리 두기가 가능하도록 분산하여 이용하기
- ○ 기념식장 내에서 음식물 섭취를 자제하기

○ 줄서기 등 대기할 때에는 사람 간 2m(최소 1m) 간격을 두기

○ 수건, 물병 등은 개인물품을 사용하고 공동 사용하지 않기

○ 셔틀버스 이용 시는 마스크를 착용하고, 다른 사람과 2m(최소 1m) 이상 거리가 유지되도록 한 칸씩 띄어 앉고 대화는 자제하기

○ 기념식 참여 중 발열, 호흡기 증상이 나타나면 보건용 마스크를 착용하고 행사 관계자에게 알리고 귀가하기

※ 기념식장 내 지역축제, 전시행사, 음식점·카페, 공연장, 유원시설, 공중화장실 등 유형 해당시 관련 지침을 준용

2 책임자·종사자

[공통사항]

○ 방역관리자 지정 및 지역 보건소 담당자의 연락망을 확보하는 등 방역 협력체계 구축하기

○ 공동체 내 밀접 접촉이 일어나는 동일 부서, 동일 장소 등에 2~3명 이상의 유증상자가 3~4일 내에 발생 시 유증상자가 코로나19 검사를 받도록 안내하며, 유증상자 추가 발생 시 보건소에 집단감염 가능성을 신고하기

○ 종사자가 발열 또는 호흡기 증상이 있는 경우 출근 중단 및 즉시 퇴근 조치하기

○ 사람 간 간격을 2m(최소 1m) 이상 거리 두기

○ 손을 씻을 수 있는 시설 또는 손 소독제를 비치하고, 손 씻기 및 기침예절 준수 안내문 게시하기

○ 자연 환기가 가능한 경우 창문을 상시 열어두고, 에어컨 사용 등으로 상시적으로 창문을 열어두기 어려운 경우 2시간마다 1회 이상 환기하기

○ 공용으로 사용하는 물건(출입구 손잡이 등) 및 표면은 매일 1회 이상 자주 소독하기

○ 고객(이용자)을 직접 응대하는 경우 마스크 착용하기

○ 발열 또는 호흡기 증상이 있거나 최근 14일 이내 해외여행을 한 경우 방문 자제 안내하기

○ 실내 다중이용시설을 이용하는 경우 마스크 착용 안내하기

○ 실외에서 2m 거리 유지가 안되는 경우 마스크 착용 안내하기

[해당 유형 적용사항]

○ 가급적 온라인으로 진행하기

- 불가피하게 현장 기념식을 개최하는 경우, 온라인·오프라인 병행, 사전 예약제, 시간제 운영 등의 방식을 활용하여 관람객이 한꺼번에 몰리지 않도록 분산 유도하기

○ 입장권은 현장 판매보다 사전예매를 안내하기

○ 기념식장이 밀집되지 않고 2m(최소 1m) 거리 두기가 유지되도록 입장 정원을 제한하기

* 예) 기념식장 바닥 면적 $4m^2$ 당 입장객 1명으로 제한, 행사장 수용 인원의 50%로 입장 정원 제한 등 거리 두기가 가능한 방안 마련

○ 참석자, 행사 관계자 등 출입하는 사람에 대한 증상 여부(발열, 호흡기 증상 등) 확인 및 명부(전자 또는 수기)를 기록 관리(4주 보관 후 폐기)하기

- 행사 중 발열, 호흡기 증상이 나타난 참석자, 관계자 등은 귀가 조치하기

○ 출입 관리요원 등은 수시로 손 소독제 사용하기

○ 기념식 객석은 2m(최소 1m) 거리 두기가 유지되도록 지그재그로 한 칸 띄워 배치하기

○ 기념식 무대와 객석 간 사이에는 최대한 거리(최소 2m) 유지하기

○ 침방울이 튀는 행위(노래부르기, 구호외치기 등)와 프로그램은 최소화 하기

○ 마이크를 사용하는 경우 마이크는 커버를 씌우고, 개인마다 마이크 덮개를 새것으로 교체하기

○ 실내 기념식인 경우, 충분히 환기를 실시하고 객석과 무대 등은 소독하기

○ 출입구 및 행사장 내 곳곳에 휴지 및 뚜껑 있는 쓰레기통 비치하기

○ 단체식사 제공은 자제하며, 불가피한 경우에는 개인 도시락 형태로 제공하기

○ 해외 거주하는 인사들의 초대는 자제하기

○ 행사장 셔틀버스 운영 시, 마스크 착용 및 다른 사람과 2m(최소 1m) 이상 거리가 유지되도록 한 칸씩 띄어 앉고 대화 자제 안내하기

※ 기념시장 내 지역축제, 전시행사, 음식점·카페, 공연장, 유원시설, 공중화장실 등 유형 해당시 관련 지침을 준용

Ⅱ. 일 상 (6. 종교생활)

6-1. 생활 속 거리 두기 지침 : 종교시설

1 이용자

[공통사항]

○ 발열 또는 호흡기 증상(기침, 인후통 등)이 있거나 최근 14일 이내 해외여행을 한 경우 방문 자제하기

○ 다른 사람과 2m(최소 1m) 이상 거리 두기

○ 흐르는 물과 비누로 30초 이상 손을 씻거나 손 소독제로 손 소독하기

○ 기침이나 재채기를 할 때는 휴지, 옷소매로 입과 코 가리기

○ 침방울이 튀는 행위(노래부르기, 소리지르기 등)나 신체접촉(악수, 포옹 등) 자제하기

○ 실내 다중이용시설을 이용하는 경우 마스크 착용하기

○ 실외에서 2m 거리 유지가 안되는 경우 마스크 착용하기

[해당 유형 적용사항]

○ 출입 시 증상여부(발열, 호흡기 증상 등) 확인 및 명부(전자 또는 수기) 기록 관리(4주 보관 후 폐기) 등 방역에 협조하기

○ 고위험군은 시설이용을 자제하거나 불가피하게 방문할 경우 마스크 착용하기

* 고위험군: 65세 이상 어르신, 임신부, 만성질환자 등

○ 온라인 등 비대면 · 비접촉 종교행사 활용하기

○ 접촉·대면 모임(구역, 성경공부, 연령별 등) 및 행사(수련회, 기도회, 여름캠프 등)는 자제하고, 부득이한 경우에는 방역수칙을 철저히 준수하기

○ 좌석 간격 등 다른 사람들과 2m(최소 1m) 이상 거리 유지하기

○ 입과 코를 가리도록 적절하게 마스크 상시 착용하기

○ 합창 등 노래를 부르거나(성가대, 합창단 등), 큰 소리로 말하거나 기도하는 등 침방울이 많이 발생하는 행위는 하지 않기

○ 종교시설 내에서는 음식 섭취하지 않기

○ 공용물품(책, 컵 등) 사용은 자제하고, 개인물품 사용하기

○ 공용 차량 이용 시, 손 소독, 마스크 착용, 대화 자제 등 개인 방역수칙 준수하기

2 책임자·종사자

[공통사항]

○ 방역관리자 지정 및 지역 보건소 담당자의 연락망을 확보하는 등 방역 협력체계 구축하기

○ 공동체 내 밀접 접촉이 일어나는 동일 부서, 동일 장소 등에 2~3명 이상의 유증상자가 3~4일 내에 발생 시 유증상자가 코로나19 검사를 받도록 안내하며, 유증상자가 추가 발생 시 보건소에 집단감염 가능성을 신고하기

○ 종사자가 발열 또는 호흡기 증상이 있는 경우 출근 중단 및 즉시 퇴근 조치하기

○ 사람 간 간격을 2m(최소 1m) 이상 거리 두기

○ 손을 씻을 수 있는 시설 또는 손 소독제를 비치하고, 손 씻기 및 기침예절 준수 안내문 게시하기

○ 자연 환기가 가능한 경우 창문을 상시 열어두고, 에어컨 사용 등으로 상시적으로 창문을 열어두기 어려운 경우 2시간마다 1회 이상 환기하기

○ 공용으로 사용하는 물건(출입구 손잡이 등) 및 표면은 매일 1회 이상 자주 소독하기

○ 고객(이용자)을 직접 응대하는 경우 마스크 착용하기

○ 발열 또는 호흡기 증상이 있거나 최근 14일 이내 해외여행을 한 경우 방문 자제 안내하기

○ 실내 다중이용시설을 이용하는 경우 마스크 착용 안내하기

○ 실외에서 2m 거리 유지가 안되는 경우 마스크 착용 안내하기

[해당 유형 적용사항]

○ 이용자 및 종사자에 대한 증상 여부(발열, 호흡기 증상 등) 확인 및 명부(전자 또는 수기)를 작성 관리(4주 보관 후 폐기)하기

○ 고위험군은 시설이용을 자제하고, 불가피하게 방문할 경우 마스크 착용 안내하기

* 고위험군: 65세 이상 어르신, 임신부, 만성질환자 등

○ 온라인 등 비대면·비접촉 종교행사 등을 활성화하고, 대규모 행사·단체회합 등은 최대한 자제하고, 각종 모임(구역, 성경공부, 연령별 등) 및 행사(수련회, 기도회, 여름캠프 등)는 자제하기

- 부득이하게 개최해야 할 경우 방역수칙을 철저히 준수하도록 안내하기

○ 종교 행사 시 자리 배치, 입·퇴장 시간 분산, 이용 인원 제한 등으로 다른 사람과 2m(최소 1m) 이상 거리 유지가 가능하도록 하기

○ 개인 위생수칙 준수, 생활 속 거리 두기의 필요성 등에 대한 주기적 교육·안내하기

○ 종교시설 종사자 및 이용자 전원에게 마스크 착용을 안내하고, 마스크 미착용 방문자를 위한 일회용 마스크 비치하기

○ 마이크 사용 시, 반드시 덮개 사용 및 가급적 개인별 사용토록 비치하기

○ 이용자가 합창 등 노래를 부르거나(성가대, 합창단 등), 큰 소리로 말하거나 기도하는 등 침방울이 많이 발생하는 행위를 하지 않도록 안내하기

○ 종교시설 내에서 음식은 제공하지 않고, 이용자가 음식을 섭취하지 않도록 안내하기

○ 종교시설 내 곳곳에 손 소독제 비치 및 손 소독 안내하기

○ 종교 행사 후 시설 주기적 환기 및 소독 실시하기(소독 관리대장 작성)

* 손이 자주 닿는 표면(출입문 손잡이, 전기스위치, 승강기 버튼, 손잡이, 계단 손잡이, 화장실 변기 손잡이, 수도꼭지 손잡이 등) 수시 소독

○ 공용물품(책, 컵 등) 비치는 자제하고, 가능한 개인물품을 사용하도록 안내하기

○ 공용차량 운행 시, 차량 내부에 손 소독제 비치, 탑승자 마스크 착용 등 개인 위생 수칙 준수 안내 및 차량 내부 수시 소독하기

* 차량 운행 시 자주 창문을 열어 환기시키기

※ 종교시설 내 공중화장실, 공용차량 등은 해당 유형(공중화장실, 대중교통 등)의 지침을 준용

Ⅱ. 일 상 (7. 병·의원 갈 때)

7-1. 생활 속 거리 두기 지침 : 병·의원(외래진료 및 면회)

※ 코로나19 환자·자가격리자 등 대상이 아닌 일반적인 외래 방문·면회 시 권고임

1. 외래 진료(예방접종 포함) 시

(1) 병·의원 방문 전

- ○ 발열 또는 호흡기 증상(기침, 인후통 등)이 있거나 최근 14일 이내 해외여행을 한 경우 방문 자제하기
  - 발열 또는 호흡기 증상이 있으나 병·의원을 방문해야 할 경우, 콜센터(☎1339, ☎지역번호+120) 및 보건소에 문의하여 선별진료소를 이용하기
- ○ 병·의원 방문 전, 사전 예약하여 대기시간 최소화하기

(2) 병·의원 방문 시

- ○ 보호자 인원은 최소화하기
- ○ 환자와 보호자는 반드시 마스크를 착용하기
  - 단 24개월 미만 유아, 주변의 도움 없이 스스로 마스크를 제거하기 어려운 사람, 마스크 착용시 호흡이 어려운 사람은 마스크를 착용하지 않기 (마스크 착용한 경우 각별한 주의 필요)
  - 마스크 착용을 유지하고, 귀가 후 마스크를 벗을 때까지 손으로 만지지 않기
- ○ 의료기관 출입 전, 진료를 마친 후에는, 반드시 흐르는 물과 비누로 30초 이상 손을 씻거나 손 소독제로 손 소독하기
- ○ 접수, 대기 시 사람 간 거리 2m(최소 1m) 유지하기
- ○ 기침, 재채기를 할 때는 휴지, 옷소매로 입과 코를 가리기
- ○ 침방울이 튀는 행위(노래부르기, 소리지르기 등)나 신체접촉(악수, 포옹) 등 자제하기

(3) 병·의원 방문 후

- ○ 귀가 후 마스크를 벗고, 손 씻기(물과 비누로 30초 이상 또는 손 소독제 이용)

2. 면회(면회자)

○ 방문 면회는 자제하고 전화, 영상통화 등의 방법 활용하기

○ 불가피하게 면회 시, 면회 인원은 최소화하고 가급적 빠른 시간에 면회를 끝내기

- 면회 가능 여부를 사전에 확인하기
- 방문객은 발열 또는 호흡기 증상(기침, 인후통 등) 및 확진자 접촉여부 등 위험요인이 있는 경우 방문을 하지 않기

  * 해당 의료기관의 면회에 관한 내규가 있는 경우 사전에 확인하기
- 출입 시 증상 여부(발열, 호흡기 증상 등) 확인 및 명부(전자 또는 수기) 작성 관리 (4주 보관 후 폐기) 등 방역에 협조하기
- 환자 방문 전과 후에는 반드시 흐르는 물과 비누로 30초 이상 손을 씻거나 손 소독제로 손 소독하기
- 환자와 2m(최소 1m) 이상 거리를 유지하고, 반드시 마스크를 착용하고 대화하기

※ 면회관련 행정명령 등 별도 규정이 있는 경우 이를 준수

Ⅱ. 일 상(8. 에어컨 사용할 때)

8-1. 생활 속 거리 두기 지침 : 에어컨 사용

1 일반원칙

○ **(기본 방향)** 에어컨 사용 시 실내공기가 재순환되고 바람으로 인해 비말이 더 멀리 확산될 우려가 있으므로 환기, 풍량에 주의하여 사용하기

○ **(환기)** 에어컨 사용으로 실내공기가 오래 머물게 되면 감염위험이 높아질 수 있기 때문에 신선한 외부 공기로 환기를 자주하기

○ **(풍량)** 에어컨 바람이 사람의 몸에 직접 닿지 않도록 하고, 바람의 세기를 낮춰서 사용하기

2 다중이용시설 사용시

○ 창문을 닫고 에어컨을 사용하되, 2시간마다 1회 이상 환기하기

○ 환기 시에는 가급적 자연환기 하며, 창문을 개방하여 맞통풍하기

○ 자연환기가 아닌 기계환기를 하는 경우에 외부공기 도입량을 가능한 높게 설정하여 최대한 외부공기로 환기하기

○ 기계환기를 하는 경우에도 자연환기가 가능하면 병행하기

○ 에어컨 바람의 방향은 사람에게 직접 향하지 않게 하며, 바람의 세기는 약하게 하기

○ 에어컨을 가동하면서 선풍기를 사용하는 것은 내부공기 재순환을 유발할 수 있어 주의하기

○ 에어컨 필터는 기기 매뉴얼에 따라 적절하게 유지관리 하기

○ 에어컨 필터 청소 또는 교체 시에는 마스크, 장갑 등 기본적인 방호조치 하에 실시하고, 완료 후 손 씻기 등 위생수칙을 준수하기

○ 환기가 불가능한 밀폐시설에서 에어컨을 사용할 때에는

- 모든 이용자가 마스크를 착용하도록 관리를 강화하고,
- 시설 내의 소독을 자주(최소 일 1회 이상) 실시하며,
- 유증상자가 시설을 이용하지 않도록 사전안내, 출입관리를 강화하기

3 코로나19 환자가 다수 발생하는 유행지역의 경우, 환기가 불가능한 밀폐시설은 가급적 에어컨을 사용하지 않기

Ⅱ. 일 상(9. 공동생활)

9-1. 생활 속 거리 두기 지침 : 기숙사

1 이용자

[공통사항]

○ 발열 또는 호흡기 증상(기침, 인후통 등)이 있거나 최근 14일 이내 해외여행을 한 경우 이용을 자제하기

○ 다른 사람과 2m(최소 1m) 이상 거리 두기

○ 흐르는 물과 비누로 30초 이상 손을 씻거나 손 소독제로 손 소독하기

○ 기침이나 재채기를 할 때는 휴지, 옷소매로 입과 코 가리기

○ 침방울이 튀는 행위(노래부르기, 구호외치기 등)나 신체접촉(악수, 포옹 등) 자제하기

○ 실내 다중이용시설을 이용하는 경우 마스크 착용하기

○ 실외에서 2m 거리 유지가 안되는 경우 마스크 착용하기

[해당 유형 적용사항]

○ 집에서 통학 또는 출근 등이 가능한 경우 기숙사 이용을 자제하기

○ 기숙사 입소 전, 호흡기질환 등 건강상태 확인하기

○ 매일 증상 여부(발열, 호흡기 증상 등) 확인 등 방역에 협조하기

○ 발열, 호흡기 증상이 나타나면 보건용 마스크를 착용하고 기숙사 관리자에게 즉시 알리고 본인 방에서 쉬면서 증상 확인하기

○ 기숙사 내 이동(지인 방 방문 등) 및 공용공간(식당, 세탁실, 휴게실, 매점 등)에 머무르는 시간 최소화하기

○ 방 밖을 이동하는 경우 다른 사람과 2m(최소 1m) 이상 거리를 두고, 거리 유지가 안되는 경우 마스크 착용하기

○ 자연 환기가 가능한 경우 창문을 상시 열어두고, 에어컨 사용 등으로 상시적으로 창문을 열어두기 어려운 경우 2시간마다 1회 이상 환기하기

○ 공용 물품을 공유하지 않고 개인 물품 사용하기

○ 구내식당 이용 시 가급적 일렬 또는 지그재그로 떨어 앉고 대화 자제하기

○ 기숙사 이용자 외 보호자·외부인 등 방문하지 않기

※ **기숙사 내 음식점·카페·실내체육시설·공중화장실 등 이용 시 해당 유형의 지침을 준용**

2 책임자·관리자

[공통사항]

- ○ 방역을 관리하는 담당부서(관리자)를 지정하고 지역 보건소 담당자의 연락망을 확보하는 등 방역 협력체계 구축하기
- ○ 공동체 내 밀접 접촉이 일어나는 동일 부서, 동일 학급, 동일 장소 등에 2~3명 이상의 유증상자가 3~4일 내에 발생 시 유증상자가 코로나19 검사를 받도록 안내하며, 유증상자가 추가 발생 시 보건소에 집단감염 가능성을 신고하기
- ○ 종사자가 발열 또는 호흡기 증상이 있는 경우 출근 중단 및 즉시 퇴근 조치하기
- ○ 사람 간 간격을 2m(최소 1m) 이상 거리 두기
- ○ 손을 씻을 수 있는 시설 또는 손 소독제를 비치하고, 손 씻기 및 기침예절 준수 안내문 게시하기
- ○ 자연 환기가 가능한 경우 창문을 상시 열어두고, 에어컨 사용 등으로 상시적으로 창문을 열어두기 어려운 경우 2시간마다 1회 이상 환기하기
- ○ 공용으로 사용하는 물건(출입구 손잡이 등) 및 표면은 매일 1회 이상 자주 소독하기
- ○ 고객(이용자)을 직접 응대하는 경우 마스크 착용하게 하기
- ○ 발열 또는 호흡기 증상이 있거나 최근 14일 이내 해외여행을 한 경우 방문 자제 안내하기
- ○ 실내 다중이용시설을 이용하는 경우 마스크 착용 안내하기
- ○ 실외에서 2m 거리 유지가 안되는 경우 마스크 착용 안내하기

[해당 유형 적용사항]

- ○ 집에서 통학 또는 출근 등이 가능한 경우 기숙사 이용을 자제하고 집에서 통학 또는 출근하도록 하기
- ○ 기숙사 운영 전에 모든 입소자에 대한 건강상태(발열 및 호흡기 질환 여부 등) 확인하기
- ○ 가급적 1인 1실을 배정하고, 다인실의 경우 침대 간 거리를 충분히 확보하는 등 거리 두기를 유지할 수 있도록 인원 배정하기
- ○ 기숙사 이용자에 대해 매일 증상 여부(발열, 호흡기 증상 등) 확인하기
- ○ 유증상자는 보건용 마스크를 착용하고 주변 사람과 만나는 것을 최대한 삼가도록 안내하기

○ 식사시간은 시차를 두고 운영하는 등 이용자 간 밀집을 최소화하기<br>(예: A조/18:30~19:30, B조/19:30~20:30)<br>○ 구내식당 좌석 간 투명 가림막을 설치하거나 가급적 일렬 또는 지그재그로 앉게 하기<br>○ 기숙사 이용자 외 보호자·외부인 등 방문 제한하기<br>**※ 기숙사 내 음식점·카페·실내체육시설·공중화장실 등 이용 시 해당 유형의 지침을 준용**

Ⅲ. 여 가 (1. 여행할 때)

1-1. 생활 속 거리 두기 지침 : 호텔 · 콘도업

1 이용자

[공통사항]

○ 발열 또는 호흡기 증상(기침, 인후통 등)이 있거나 최근 14일 이내 해외여행을 한 경우 방문 자제하기

○ 다른 사람과 2m(최소 1m) 이상 거리 두기

○ 흐르는 물과 비누로 30초 이상 손을 씻거나 손 소독제로 손 소독하기

○ 기침이나 재채기를 할 때는 휴지, 옷소매로 입과 코 가리기

○ 침방울이 튀는 행위(노래부르기, 소리지르기 등)나 신체접촉(악수, 포옹 등) 자제하기

○ 실내 다중이용시설을 이용하는 경우 마스크 착용하기

○ 실외에서 2m 거리 유지가 안되는 경우 마스크 착용하기

[해당 유형 적용사항]

○ 출입 시 증상 여부(발열, 호흡기 증상 등) 확인 등 방역에 협조하기

※ 시설 내 음식점·카페, 수영장, 실내체육관 등 이용 시 해당 유형의 지침을 준용

2 책임자·종사자

[공통사항]

○ 방역관리자 지정 및 지역 보건소 담당자의 연락망을 확보하는 등 방역 협력체계 구축하기

○ 공동체 내 밀접 접촉이 일어나는 동일 부서, 동일 장소 등에 2~3명 이상의 유증상자가 3~4일 내에 발생 시 유증상자가 코로나19 검사를 받도록 안내하며, 유증상자가 추가 발생 시 보건소에 집단감염 가능성을 신고하기

○ 종사자가 발열 또는 호흡기 증상이 있는 경우 출근 중단 및 즉시 퇴근 조치하기

○ 사람 간 간격을 2m(최소 1m) 이상 거리 두기

○ 손을 씻을 수 있는 시설 또는 손 소독제를 비치하고, 손 씻기 및 기침예절 준수 안내문 게시하기

○ 자연 환기가 가능한 경우 창문을 상시 열어두고, 에어컨 사용 등으로 상시적으로 창문을 열어두기 어려운 경우 2시간마다 1회 이상 환기하기

○ 공용으로 사용하는 물건(출입구 손잡이 등) 및 표면은 매일 1회 이상 자주 소독하기

○ 고객(이용자)을 직접 응대하는 경우 마스크 착용하기

○ 발열 또는 호흡기 증상이 있거나 최근 14일 이내 해외여행을 한 경우 방문 자제 안내하기

○ 실내 다중이용시설을 이용하는 경우 마스크 착용 안내하기

○ 실외에서 2m 거리 유지가 안되는 경우 마스크 착용 안내하기

[해당 유형 적용사항]

○ 연회 행사 후 소독 및 환기 실시(일시·관리자 확인 포함 대장 작성)하기

○ 출입구 및 시설 내 곳곳에 휴지 및 뚜껑 있는 쓰레기통 비치하기

○ 투숙객 이용 후 객실 창문을 열어 환기, 화장실 등 객실 청소, 소독하기

○ 개인 위생수칙 준수, 생활 속 거리 두기의 필요성 등에 대한 주기적 교육·안내하기

○ 출입하는 사람에 대한 증상 여부(발열, 호흡기 증상 등) 확인하기

※ 시설 내 음식점·카페, 수영장, 실내체육관 등이 있는 경우 해당 유형의 지침을 준용

1-2. 생활 속 거리 두기 지침 : 유원시설
(놀이공원, 워터파크 등 종합 · 일반 · 기타 유원시설)

1 이용자

[공통사항]

○ 발열 또는 호흡기 증상(기침, 인후통 등)이 있거나 최근 14일 이내 해외여행을 한 경우 방문 자제하기

○ 다른 사람과 2m(최소 1m) 이상 거리 두기

○ 흐르는 물과 비누로 30초 이상 손을 씻거나 손 소독제로 손 소독하기

○ 기침이나 재채기를 할 때는 휴지, 옷소매로 입과 코 가리기

○ 침방울이 튀는 행위(노래부르기, 소리지르기 등)나 신체접촉(악수, 포옹 등) 자제하기

○ 실내 다중이용시설을 이용하는 경우 마스크 착용하기(단, 물속에서는 사용 제외)

○ 실외에서 2m 거리유지가 안되는 경우 마스크 착용하기(단, 물속에서는 사용 제외)

[해당 유형 적용사항]

○ 유원시설(놀이공원, 워터파크, 키즈카페 등) 출입 시 증상 여부(발열, 호흡기 증상 등) 확인 등 방역 협조하기

○ 수건, 수영복, 수경, 스노클 등 휴대용 용품은 개인물품을 사용하기

○ 칸막이가 없는 샤워실의 경우, 다른 사람과의 거리유지를 위해 한 칸씩 띄워 사용하기

○ 탈의실(락커룸), 대기실(휴게실) 등 실내 공용공간에 머무르는 시간을 최소화하기

○ 가급적 실내보다 실외에 위치한 벤치, 정자, 그늘막(카바나), 일광용 의자(선베드) 등의 휴게시설을 이용하기

○ 실내 휴게실, 카페, 매점 등 다중이용공간 밀집되지 않도록 분산하여 이용하기

※ 시설 내 음식점·카페, 공중화장실, 목욕탕 등 이용 시 해당 유형의 지침을 준용

2 책임자·종사자

[공통사항]

- ○ 방역관리자 지정 및 지역 보건소담당자의 연락망을 확보하는 등 방역 협력체계 구축하기
- ○ 공동체 내 밀접 접촉이 일어나는 동일 장소 등에 2~3명 이상의 유증상자가 3~4일 내에 발생 시 유증상자가 코로나19 검사를 받도록 안내하며, 유증상자가 추가 발생 시 보건소에 집단감염 가능성을 신고하기
- ○ 종사자가 발열 또는 호흡기 증상이 있는 경우 출근 중단 및 즉시 퇴근 조치하기
- ○ 사람 간 간격을 2m(최소 1m) 이상 거리 두기
- ○ 손을 씻을 수 있는 시설 또는 손 소독제를 비치하고, 손 씻기 및 기침예절 준수 안내문 게시하기
- ○ 자연 환기가 가능한 경우 창문을 상시 열어두고, 에어컨 사용 등으로 상시적으로 창문을 열어두기 어려운 경우 2시간마다 1회 이상 환기하기
- ○ 공용으로 사용하는 물건(출입구 손잡이 등) 및 표면은 매일 1회 이상 자주 소독하기
- ○ 고객(이용자)을 직접 응대하는 경우 마스크 착용하기
- ○ 발열 또는 호흡기 증상이 있거나 최근 14일 이내 해외여행을 한 경우 방문 자제 안내하기
- ○ 실내 다중이용시설을 이용하는 경우 마스크 착용 안내하기(단, 물속에서는 사용 제외)
- ○ 실외에서 2m 거리유지가 안되는 경우 마스크 착용 안내하기(단, 물속에서는 사용 제외)

[해당 유형 적용사항]

- ○ 유원시설에 출입하는 사람에 대한 증상여부(발열, 호흡기 증상 등) 확인하기
- ○ 관람객과 신체접촉을 피하고 거리 2m(최소 1m) 이상 유지할 수 있도록 직원 행동지침 마련하기
- ○ 매일 1회 이상 놀이기구(손잡이, 난간 등 특히 손이 자주 닿는 표면) 소독 실시하기
- ○ 직원들이 휴게실, 탈의실 등 공용구역에서 쉬는 경우, 마스크를 착용하고 시간 차 두고 이용하기
- ○ 예약제도 운영 등 시간대별 관람객 수를 제한하여 관람객 집중 방지하기
- ○ 입장권 현장판매보다 사전예매 독려, 구역별 입·퇴장 시간 구분, 공용구역 밀집 방지를 위한 동선 관리 등 이용객 분산 유도하기
- ○ 놀이기구 탑승 시 좌석은 지그재그로 한 칸 띄어 앉도록 안내하기

○ 줄서는 장소 등 사람이 몰리는 곳에는 거리 유지할 수 있도록 2m(최소 1m) 이상 간격으로 위치 표시하여 안내하기

○ 대규모 행사, 공동 활동 등 최소화하기

○ 식사시간 또는 쉬는 시간에 다른 사람과 간격 유지(한 방향 보기, 띄워 앉기 등) 유도하기

○ 출입구 및 시설 내 각처에 휴지 및 뚜껑 있는 쓰레기통 비치하기

○ 개인위생수칙 준수, 생활 속 거리 두기의 필요성 등에 대한 주기적으로 교육·안내하기

[물놀이형 유원시설 추가 적용사항]

○ 수영복, 수건, 물놀이용품(개인별 휴대가능용품) 등 개인물품 사용 안내하기

○ 공용물품(그늘막(카바나), 일광용 의자(선베드) 등)은 사용 후 다른 사람이 이용하기 전에 소독하기

○ 탈의실(락커룸), 대기실(휴게실) 등 부대시설의 손이 자주 닿는 표면은 매일 1회 이상 소독하기

○ 탈의실(락커룸), 샤워실, 대기실(휴게실) 등 공용시설은 거리두기를 유지할 수 있도록 사용 인원을 줄이고, 머무르는 시간 최소화하도록 안내하기

○ 일광용 의자(선베드) 등 개인 휴게시설은 실외에 2m(최소 1m) 이상 간격으로 배치하기

○ 실내보다 실외에 위치한 벤치, 정자, 그늘막(카바나), 일광용 의자(선베드) 등의 휴게시설을 이용하도록 안내하기

○ 물놀이 시설 내 이용자 간 2m(최소 1m) 이상 거리두기가 유지될 수 있도록 관리하기

○ 이용자 간 거리 유지 2m(최소 1m)가 가능하도록 입장 인원을 관리하기

○ 안전요원에게 기본수칙 준수 감시(마스크 착용, 사회적 거리 유지 등)에 대한 업무를 중복 배정하지 않고, 다른 담당 직원에게 업무 배정하기

※ 시설 내 음식점·카페, 공중화장실, 목욕탕 등 이용 시 해당 유형의 지침을 준용

1-3. 생활 속 거리 두기 지침 : 야영장

1 이용자

[공통사항]

○ 발열 또는 호흡기 증상(기침, 인후통 등)이 있거나 최근 14일 이내 해외여행을 한 경우 방문 자제하기

○ 다른 사람과 2m(최소 1m) 이상 거리 두기

○ 흐르는 물과 비누로 30초 이상 손을 씻거나 손 소독제로 손 소독하기

○ 기침이나 재채기를 할 때는 휴지, 옷소매로 입과 코 가리기

○ 침방울이 튀는 행위(노래부르기, 소리지르기 등)나 신체접촉(악수, 포옹 등) 자제하기

○ 실내 다중이용시설을 이용하는 경우 마스크 착용하기

○ 실외에서 2m 거리 유지가 안되는 경우 마스크 착용하기

[해당 유형 적용사항]

○ 가족 구성원 외 다수 인원의 야영장 방문 자제하며, 텐트 설치 시 최소 2m 이상 거리를 두어 설치하기

○ 개인 텐트, 글램핑, 야영용 트레일러, 캠핑카 등 실내 공간인 야영시설을 자주 환기하기

○ 관리사무소 및 취사장, 공용 개수대, 샤워실 등 야영장 내 공용시설 이용 시 다른 사람과 2m(최소 1m) 이상 거리 두고 손을 자주 씻거나 손 소독제 자주 사용하기

2 책임자·종사자

[공통사항]

○ 방역관리자 지정 및 지역 보건소 담당자의 연락망을 확보하는 등 방역 협력체계 구축하기

○ 공동체 내 밀접 접촉이 일어나는 동일 부서, 동일 장소 등에 2~3명 이상의 유증상자가 3~4일 내에 발생 시 유증상자가 코로나19 검사를 받도록 안내하며, 유증상자가 추가 발생 시 보건소에 집단감염 가능성을 신고하기

○ 종사자가 발열 또는 호흡기 증상이 있는 경우 출근 중단 및 즉시 퇴근 조치하기
○ 사람 간 간격을 2m(최소 1m) 이상 거리 두기
○ 손을 씻을 수 있는 시설 또는 손 소독제를 비치하고, 손 씻기 및 기침예절 준수 안내문 게시하기
○ 자연 환기가 가능한 경우 창문을 상시 열어두고, 에어컨 사용 등으로 상시적으로 창문을 열어두기 어려운 경우 2시간마다 1회 이상 환기하기
○ 공용으로 사용하는 물건(출입구 손잡이 등) 및 표면은 매일 1회 이상 자주 소독하기
○ 고객(이용자)을 직접 응대하는 경우 마스크 착용하기
○ 발열 또는 호흡기 증상이 있거나 최근 14일 이내 해외여행을 한 경우 방문 자제 안내하기
○ 실내 다중이용시설을 이용하는 경우 마스크 착용 안내하기
○ 실외에서 2m 거리 유지가 안되는 경우 마스크 착용 안내하기

[해당 유형 적용사항]

○ 사업주가 설치하여 제공하는 야영용 시설(글램핑, 야영용 트레일러) 및 공용시설(취사장, 샤워실, 화장실 등) 수시 소독 및 환기 실시(일시·관리자 확인 포함 대장작성)하기
○ 단체 식사 제공 금지하기
○ 예약제도 운영 등 일 이용객 수를 제한하여 야영지 내 공간 확보하기
○ 야영객 텐트 설치 시 최소 2m 이상 거리를 두도록 안내하기
○ 관리사무소 및 취사장, 공용 개수대, 샤워실 등 야영장 내 공용시설에 이용객이 2m(최소 1m) 이상 간격을 둘 수 있도록 시설 배치하거나 표시하기

1-4. 생활 속 거리 두기 지침 : 동물원

1 이용자

[공통사항]

○ 발열 또는 호흡기 증상(기침, 인후통 등)이 있거나 최근 14일 이내 해외여행을 한 경우 방문 자제하기

○ 다른 사람과 2m(최소 1m) 이상 거리 두기

○ 흐르는 물과 비누로 30초 이상 손을 씻거나 손 소독제로 손 소독하기

○ 기침이나 재채기를 할 때는 휴지, 옷소매로 입과 코 가리기

○ 침방울이 튀는 행위(노래부르기, 소리지르기 등)나 신체접촉(악수, 포옹 등) 자제하기

○ 실내 다중이용시설을 이용하는 경우 마스크 착용하기

○ 실외에서 2m 거리 유지가 안되는 경우 마스크 착용하기

[해당 유형 적용사항]

○ 입장권 구매 시 현장 구매보다 사전 예매하기

○ 관람 시 동물과의 직접 접촉 최대한 자제 및 접촉 전·후 즉시 손 씻기·소독하기

※ 시설 내 음식점·카페 등 이용 시 해당 유형의 지침을 준용

2 책임자·종사자

[공통사항]

○ 방역관리자 지정 및 지역 보건소 담당자의 연락망을 확보하는 등 방역 협력체계 구축하기

○ 공동체 내 밀접 접촉이 일어나는 동일 부서, 동일 장소 등에 2~3명 이상의 유증상자가 3~4일 내에 발생 시 유증상자가 코로나19 검사를 받도록 안내하며, 유증상자가 추가 발생 시 보건소에 집단감염 가능성을 신고하기

○ 종사자가 발열 또는 호흡기 증상이 있는 경우 출근 중단 및 즉시 퇴근 조치하기

○ 사람 간 간격을 2m(최소 1m) 이상 거리 두기

○ 손을 씻을 수 있는 시설 또는 손 소독제를 비치하고, 손 씻기 및 기침예절 준수 안내문 게시하기

○ 자연 환기가 가능한 경우 창문을 상시 열어두고, 에어컨 사용 등으로 상시적으로 창문을 열어두기 어려운 경우 2시간마다 1회 이상 환기하기

○ 공용으로 사용하는 물건(출입구 손잡이 등) 및 표면은 매일 1회 이상 자주 소독하기

○ 고객(이용자)를 직접 응대하는 경우 마스크 착용하기

○ 발열 또는 호흡기 증상이 있거나 최근 14일 이내 해외여행을 한 경우 방문 자제 안내하기

○ 실내 다중이용시설을 이용하는 경우 마스크 착용 안내하기

○ 실외에서 2m 거리 유지가 안되는 경우 마스크 착용 안내하기

[해당 유형 적용사항]

1. 관람자 안내 및 캠페인(현수막, 포스터 부착 및 안내방송 송출 등)

○ 관람객 동물 접촉 자제 및 접촉 전·후 즉시 손 소독하기

○ 인기 동물 우리 또는 식당 등 주요 밀집 장소에 오래 머무르지 않기

○ 입장권 구매창구 관람객 밀집 방지를 위해 현장 구입 보다 사전예매 독려하기

2. 동물감염 예방관리

○ 관리자(사육사, 수의사 등) 동물 접촉 시 마스크 및 장갑 등 방역장비 착용하기

○ 관리 동물감염 여부 예찰 및 특이사항 발생 시 즉시 전파하기
(동물원 관리자 → 지자체 → 환경부)

3. 운영 및 시설관리

○ 인수공통 감염병 보유 가능 동물종에게 관람객 먹이 주기 체험 및 접촉 차단하기

○ 관람 동선을 따라 2m(최소 1m) 간격의 바닥 표시물 부착으로 사람 간 간격 유지 유도하기

○ 구역별 입·퇴장시간 설정, 공용구역 밀집 방지를 위한 동선관리 등 이용객 분산 유도하기

○ 대중이 많이 모이는 행사, 이벤트 등 개최 자제하기

○ 예약제도 운영 등 시간대별 관람객 수를 제한하여 관람객 집중 방지하기

○ 직원들이 휴게실, 탈의실 등 공용구역에서 쉬는 시간 차이 두기

○ 식사 시간 또는 쉬는 시간에 다른 사람과 2m(최소 1m) 간격 유지 안내하고 단체식사 제공 금지하기

○ 출입구 및 시설 내 곳곳에 손 소독제·휴지 및 뚜껑 있는 쓰레기통 비치하기

※ 시설 내 음식점·카페 등이 있는 경우 해당 유형의 지침을 준용

1-5. 생활 속 거리 두기 지침 : 국립공원

1 이용자·탐방객

[공통사항]

○ 발열 또는 호흡기 증상(기침, 인후통 등)이 있거나 최근 14일 이내 해외여행을 한 경우 방문 자제하기

○ 다른 사람과 2m(최소 1m) 이상 거리 두기

○ 흐르는 물과 비누로 30초 이상 손을 씻거나 손 소독제로 손 소독하기

○ 기침이나 재채기를 할 때는 휴지, 옷소매로 입과 코 가리기

○ 침방울이 튀는 행위(노래부르기, 소리지르기 등)나 신체접촉(악수, 포옹 등) 자제하기

○ 실내 다중이용시설을 이용하는 경우 마스크 착용하기

○ 실외에서 2m 거리 유지가 안되는 경우 마스크 착용하기

[해당 유형 적용사항]

○ 탐방로 입구 손 소독제, 직원 안내에 적극 협조하기

○ 단체 방문(산행)을 자제하고 탐방 인원은 최소화하기

○ 탐방로에서 우측으로 한 줄 통행하기

○ 마주보고 식사하지 않고 음식 나눠 먹지 않기

○ 대피소, 야영장 등 다중이용시설 이용 시 아래 사항에 협조하기

- 출입 전 직원 안내에 따라 체온 측정하기(37.5℃ 이상 시 출입 금지)
- 출입 후 발열 또는 호흡기 증상이 있는 경우 이용이 자제하기
- 최근 2주 사이 해외 여행력이 있는 경우 시설물 예약하지 않기
- 시설 내 공용구역(화장실, 샤워실 등) 차례대로 이용하기(타인 접촉 최소화)

※ **시설 내 식당·카페, 숙박시설, 야영장 이용 시 관련 지침을 준용**

2 시설운영자·관리자

[공통사항]

- ○ 방역관리자 지정 및 지역 보건소 담당자의 연락망을 확보하는 등 방역 협력체계 구축하기
- ○ 공동체 내 밀접 접촉이 일어나는 동일 부서, 동일 장소 등에 2~3명 이상의 유증상자가 3~4일 내에 발생 시 유증상자가 코로나19 검사를 받도록 안내하며, 유증상자가 추가 발생 시 보건소에 집단감염 가능성을 신고하기
- ○ 종사자가 발열 또는 호흡기 증상이 있는 경우 출근 중단 및 즉시 퇴근 조치하기
- ○ 사람 간 간격을 2m(최소 1m) 이상 거리 두기
- ○ 손을 씻을 수 있는 시설 또는 손 소독제를 비치하고, 손 씻기 및 기침예절 준수 안내문 게시하기
- ○ 자연 환기가 가능한 경우 창문을 상시 열어두고, 에어컨 사용 등으로 상시적으로 창문을 열어두기 어려운 경우 2시간마다 1회 이상 환기하기
- ○ 공용으로 사용하는 물건(출입구 손잡이 등) 및 표면은 매일 1회 이상 자주 소독하기
- ○ 고객(이용자)을 직접 응대하는 경우 마스크 착용하게 하기
- ○ 발열 또는 호흡기 증상이 있거나 최근 14일 이내 해외여행을 한 경우 방문 자제 안내하기
- ○ 실내 다중이용시설을 이용하는 경우 마스크 착용 안내하기
- ○ 실외에서 2m 거리 유지가 안되는 경우 마스크 착용 안내하기

[해당 유형 적용사항]

1. 코로나19 관리체계 및 유관기관 협조체계 구성

- ○ 각 사무소에서는 방역관리자*를 지정하여 코로나19 예방 및 관리 책임성 부여하기
  - * 자체 점검표 활용(홍보, 환경 위생 관리 분야 이행 여부 확인)
- ○ 의심환자(의사환자, 조사대상 유증상자) 등 발생 시 즉시 대응을 위해 유관기관(시·도, 시·군·구 보건소 및 의료기관)과의 비상연락체계 구축하기
  - 탐방객 중 발열, 호흡기 증상 등 발생 시 지자체와 연계할 수 있는 핫라인 구축하기

2. 감염병 예방 홍보

- ○ 탐방 거리 두기 홍보(현수막, 문자전광판, 안내방송 등)하기
  - 주요 밀집장소(쉼터, 정상부 등): 2m 이상 거리 두기, 오래 머무르지 않기, 마스크 착용하기

- 탐방로: 2m 이상 거리 두고 우측통행하기
- 공용공간(화장실, 탐방지원센터 등): 손 소독(손 씻기), 마스크 착용하기

○ 개인 위생수칙 홍보(현수막, 문자전광판, 안내방송 등)

- 손 씻기, 기침 예절 등 코로나19 예방수칙 지키기
- 탐방로 입구 손 소독제 사용 후 공원 출입하기

○ 체온 측정 안내 현수막 게시, 관리인력 확보 및 어깨띠 부착, 체온측정 독려 확성기 안내 방송하기

3. 환경 위생 관리

○ 탐방로 입구 손 소독제(알코올 70% 이상) 비치하기

○ 화장실 내 손 세정제(액체비누)와 종이타월 등 충분히 비치하기

○ 주요 공간의 청소, 다중이용시설(화장실 등) 소독 및 주기적 환기하기

- 청소 및 소독 작업을 수행하는 직원은 적절한 개인보호구* 착용하기

* 일회용 장갑, 보건용 마스크 착용하고 필요 시 일회용 방수용 긴팔 가운 또는 방수 앞치마, 고글 또는 안면보호구 등 착용

- 사람들이 자주 접촉하는 물체의 표면(문 손잡이, 난간, 스위치 등)에 대한 소독 강화하기
- 알코올(70% 에탄올), 희석 차아염소산나트륨(500~1000ppm)등 소독제 묻은 천으로 닦기

4. 탐방객 대응

○ 마스크 착용 및 탐방객 응대 시 안전거리(2m(최소 1m) 거리 두기, 한 줄 통행) 유지하기

○ 사무소 복귀 시 개인위생 철저(손 씻기 및 마스크 착용하기)하게 지키기

○ 식사 또는 쉬는 시간에 다른 사람과 간격 유지(한 방향 보기, 띄워 앉기) 유도하기

5. 대피소, 야영장, 탐방원 등 다중이용시설 운영·관리

○ 유증상자나 최근 14일 이내에 해외여행을 한 자는 시설 이용 금지하기

○ 야영장 또는 대피소 등에 자리를 배치할 경우 서로 2m(최소 1m) 이상 간격을 두고 배치하기

○ 공용구역(화장실, 샤워실 등)에 건강거리 유지를 위해 사람들이 몰려 오래 머무르지 않도록 하기

○ 이용객 대상 마스크 착용 안내 및 실내의 경우 마스크 미착용자 입장 자제하기

○ 탐방객 이용시설은 시간당 최소 2회, 1일당 최소 2시간 이상 환기 실시 및 1일당 최소 1회 이상 소독하기

※ 시설 내 식당·카페, 숙박시설, 야영장 이용 시 관련 지침을 준용

1-6. 생활 속 거리 두기 지침 : 해수욕장

1 이용자

[공통사항]

○ 발열 또는 호흡기 증상(기침, 인후통 등)이 있거나 최근 14일 이내 해외여행을 한 경우 방문 자제하기

○ 다른 사람과 2m(최소 1m) 이상 거리 두기

○ 흐르는 물과 비누로 30초 이상 손을 씻거나 손 소독제로 손 소독하기

○ 기침이나 재채기를 할 때는 휴지, 옷소매로 입과 코 가리기

○ 침방울이 튀는 행위(노래부르기, 소리지르기 등)나 신체접촉(악수, 포옹 등) 자제하기

○ 실내 다중이용시설을 이용하는 경우 마스크 착용하기

○ 실외에서 2m 거리 유지가 안되는 경우 마스크 착용하기(단, 물속에서는 사용 제외)

[해당 유형 적용사항]

○ 가족 단위·소규모로 방문하고 동호회, 단체모임 등 많은 인원이 함께 방문하는 것은 자제하기

○ 개인 차양시설은 차양 끝을 기준으로 최소 2m 이상 거리를 두어 설치하기

○ 해수욕(물놀이, 백사장 활동) 시 다른 사람과 신체적 접촉이 없도록 주의하기

○ 백사장 및 물놀이 구역에서 침 뱉기, 코 풀기 등 체액 배출을 자제하기

○ 해수욕장 내에서 음식물 섭취를 최소화하고 대화 자제하기

○ 탈의실, 샤워실 등은 가급적 개인 숙소·시설 등을 이용하기

- 불가피하게 공용시설을 이용하는 경우, 다른 사람과 2m(최소 1m) 이상 거리 유지가 가능하도록 한 칸 떨어져 사용하고 대화는 자제하며, 머무르는 시간을 최소화하기

○ 물놀이 용품(튜브, 물안경, 구명조끼, 스노클링 장비(숨 대롱) 등)은 개인물품을 사용하기

※ 시설 내 음식점·카페, 공용화장실, 야영장 등 이용 시 해당 유형의 지침을 준용

2 책임자·종사자

[공통사항]

○ 방역관리자 지정 및 지역 보건소 담당자의 연락망을 확보하는 등 방역 협력체계 구축하기

○ 공동체 내 밀접 접촉이 일어나는 동일 부서, 동일 장소 등에 2~3명 이상의 유증상자가 3~4일 내에 발생 시 유증상자가 코로나19 검사를 받도록 안내하며, 유증상자가 추가 발생 시 보건소에 집단감염 가능성을 신고하기

○ 종사자가 발열 또는 호흡기 증상이 있는 경우 출근 중단 및 즉시 퇴근 조치하기

○ 사람 간 간격을 2m(최소 1m) 이상 거리 두기

○ 손을 씻을 수 있는 시설 또는 손 소독제를 비치하고, 손 씻기 및 기침예절 준수 안내문 게시하기

○ 자연 환기가 가능한 경우 창문을 상시 열어두고, 에어컨 사용 등으로 상시적으로 창문을 열어두기 어려운 경우 2시간마다 1회 이상 환기하기

○ 공용으로 사용하는 물건(출입구 손잡이 등) 및 표면은 매일 1회 이상 자주 소독하기

○ 고객(이용자)을 직접 응대하는 경우 마스크 착용하기

○ 발열 또는 호흡기 증상이 있거나 최근 14일 이내 해외여행을 한 경우 방문 자제 안내하기

○ 실내 다중이용시설을 이용하는 경우 마스크 착용 안내하기

○ 실외에서 2m 거리 유지가 안되는 경우 마스크 착용 안내하기(단, 물속에서는 사용 제외)

[해당 유형 적용사항]

○ 물놀이 용품(튜브, 물안경, 구명조끼, 스노클링 장비(숨 대롱) 등)은 개인용품 사용 안내하기

○ 차양막(파라솔), 일광욕 의자(선베드), 돗자리, 평상 등 해수욕장에서 대여하여 이용하는 물품은 사용 후 다른 사람이 이용하기 전에 소독하기

○ 해수욕장에 설치하는 차양시설(파라솔, 그늘막 텐트)은 사면을 개방하고 차양 끝을 기준으로 2m(최소 1m) 이상 거리를 확보하여 설치하기

○ 탈의실, 샤워실 등 공용시설은 2m(최소 1m) 이상 거리 두기를 유지할 수 있도록 사용 인원을 제한하고, 머무르는 시간 최소화하도록 안내하기

- 칸막이가 없는 샤워실의 경우, 다른 사람과의 거리 유지를 위해 한 칸씩 띄워 사용하기
- 탈의실, 샤워실 등 부대시설 소독 철저히 하기

○ 생활 속 거리 두기 홍보(현수막, 문자전광판, 안내방송 등) 및 안내하기

○ 개인 위생수칙 홍보(현수막, 문자전광판, 안내방송 등) 및 안내하기

- 손 씻기, 기침 예절, 거리 두기 등 코로나19 예방수칙 등

○ 관리사무소, 진료시설, 화장실 등 주요 다중이용시설 입구에 손 소독제 비치하기

○ 관리사무소, 진료시설에 방문하는 이용객에 대한 마스크 착용 및 발열검사 실시하기

○ 관리사무소, 진료시설, 화장실, 샤워시설 등 주요 다중이용시설은 매일 소독하고 매일 2회 이상 주기적으로 환기하기

○ 수상오토바이, 구명보트, 구명튜브, 감시탑 등 안전시설과 장비는 매일 소독하기

○ 안전관리요원은 근무교대 시 발열 검사(최소 1일 2회 이상)를 실시하고 가급적 마스크를 착용하고 근무하기

○ 수상안전요원에게 기본 수칙 준수 감시(마스크 착용, 사회적 거리 유지 등)에 대한 업무를 중복 배정하지 않고, 다른 담당 직원에게 업무 배정하기

※ 시설 내 음식점·카페, 공용화장실, 야영장 등이 있는 경우 유형의 지침을 준용

1-7. 생활 속 거리 두기 지침 : 하천·계곡

1 이용자·물놀이객

[공통사항]

○ 발열 또는 호흡기 증상(기침, 인후통 등)이 있거나 최근 14일 이내 해외여행을 한 경우 방문 자제하기
○ 다른 사람과 2m(최소 1m) 이상 거리 두기
○ 흐르는 물과 비누로 30초 이상 손을 씻거나 손 소독제로 손 소독하기
○ 기침이나 재채기를 할 때는 휴지, 옷소매로 입과 코 가리기
○ 침방울이 튀는 행위(노래부르기, 소리지르기 등)나 신체접촉(악수, 포옹 등) 자제하기
○ 실내 다중이용시설을 이용하는 경우 마스크 착용하기
○ 실외에서 2m 거리 유지가 안되는 경우 마스크 착용하기(단, 물속에서는 사용 제외)

[해당 유형 적용사항]

○ 가족 단위의 소규모로 방문하고, 동호회, 단체방문 등은 자제하기
○ 수건, 수영복, 수경, 구명조끼, 튜브 등은 개인물품을 사용하고 공동사용 자제하기
○ 물놀이 시 다른 사람과 2m(최소 1m) 이상 거리를 유지하고, 신체접촉 주의하기
○ 물놀이 지역 내에서 침·가래 뱉기, 코 풀기 등 체액이 배출되지 않도록 하기
○ 텐트, 돗자리, 그늘막 등은 다른 사람과 2m(최소 1m) 이상 거리 두어 설치하기
○ 탈의실(락커룸), 대기실(휴게실) 등 실내 공용공간에 머무르는 시간을 최소화하기
○ 칸막이가 없는 샤워실의 경우, 다른 사람과의 거리 유지를 위해 한 칸씩 띄워 사용하기
○ 물놀이 지역 내에서 음식물 섭취는 가급적 자제하기
○ 실내 휴게실, 카페, 매점 등의 다중이용공간은 2m(최소 1m) 이상 거리가 유지되도록 분산하여 이용하기

※ 시설 내 국립공원, 유원시설, 실내체육시설, 야영장 및 공중화장실 등 이용 시 해당 유형의 지침을 준용

2 관리자·시설운영자

[공통사항]

○ 방역관리자 지정 및 지역 보건소 담당자의 연락망을 확보하는 등 방역 협력체계 구축하기

○ 공동체 내 밀접 접촉이 일어나는 동일 부서, 동일 장소 등에 2~3명 이상의 유증상자가 3~4일 내에 발생 시 유증상자가 코로나19 검사를 받도록 안내하며, 유증상자가 추가 발생 시 보건소에 집단감염 가능성을 신고하기

○ 종사자가 발열 또는 호흡기 증상이 있는 경우 출근 중단 및 즉시 퇴근 조치하기

○ 사람 간 간격을 2m(최소 1m) 이상 거리 두기

○ 손을 씻을 수 있는 시설 또는 손 소독제를 비치하고, 손 씻기 및 기침예절 준수 안내문 게시하기

○ 자연 환기가 가능한 경우 창문을 상시 열어두고, 에어컨 사용 등으로 상시적으로 창문을 열어두기 어려운 경우 2시간마다 1회 이상 환기하기

○ 공용으로 사용하는 물건(출입구 손잡이, 대여물품 등) 및 표면은 매일 1회 이상 자주 소독하기

○ 고객(이용자)을 직접 응대하는 경우 마스크 착용하기

○ 발열 또는 호흡기 증상이 있거나 최근 14일 이내 해외여행을 한 경우 방문 자제 안내하기

○ 실내 다중이용시설을 이용하는 경우 마스크 착용 안내하기

○ 실외에서 2m 거리 유지가 안되는 경우 마스크 착용 안내하기(단, 물속에서는 사용 제외)

[해당 유형 적용사항]

○ 안전관리요원 등에 대한 방역수칙 교육을 실시하기

○ 유료 물놀이 지역 입장권은 현장 판매보다 사전예매를 독려하고, 이용자 간 거리 유지 2m(최소 1m)가 가능하도록 입장 인원을 제한하기

○ 물놀이 지역 내 다수 인원의 밀집을 유도하는 이벤트성 행사 등 자제하기

○ 생활 속 거리 두기 홍보(현수막, 문자전광판, 안내방송 등) 하기

○ 매일(출근 후, 퇴근 전, 교대근무 시 등) 비접촉식 체온계 등으로 안전관리요원 등에 대한 체온을 검사하고, 호흡기 증상 여부 확인하기

○ 마이크, 확성기, 경광봉, 구명조끼 등 안전관리요원의 공용장비 등은 매일 소독하여 사용하기

○ 직원들이 휴게실, 탈의실 등 공용구역에서 쉬는 경우, 마스크를 착용하고 시간 차 두고 이용하기

○ 텐트, 돗자리 그늘막 등은 2m(최소 1m) 이상 안전거리 확보 후 설치하도록 안내하기

○ 탈의실(락커룸), 대기실(휴게실) 등 부대시설의 손이 자주 닿는 표면은 매일 1회 이상 소독 및 실내 시설은 매일 2회 이상 환기하기

○ 공용시설(탈의실(락커룸), 샤워실, 대기실(휴게실), 취사장 등), 다중이용시설(카페, 매점, 식당 등)은 2m(최소 1m) 거리 두기를 유지할 수 있도록 사용 인원을 관리하고, 머무르는 시간 최소화하도록 안내하기

○ 기본 수칙(마스크 착용, 사회적 거리 유지 등) 준수 여부 안내하기

※ 시설 내 국립공원, 유원시설, 실내체육시설, 야영장 및 공중화장실 등 이용 시 해당 유형의 지침을 준용

1-8. 생활 속 거리 두기 지침 : 수상레저

1 이용자

[공통사항]

○ 발열 또는 호흡기 증상(기침, 인후통 등)이 있거나 최근 14일 이내 해외여행을 한 경우 방문 자제하기

○ 다른 사람과 2m(최소 1m) 이상 거리 두기

○ 흐르는 물과 비누로 30초 이상 손을 씻거나 손 소독제로 손 소독하기

○ 기침이나 재채기를 할 때는 휴지, 옷소매로 입과 코 가리기

○ 침방울이 튀는 행위(노래부르기, 소리지르기 등)나 신체접촉(악수, 포옹 등) 자제하기

○ 실내 다중이용시설을 이용하는 경우 마스크 착용하기

○ 실외에서 2m 거리 유지가 안되는 경우 마스크 착용하기(단, 물속에서는 사용 제외)

[해당 유형 적용사항]

○ 가족 단위의 소규모로 방문하고, 동호회 등 단체방문은 자제하기

○ 출입 시 증상 여부(발열, 호흡기 증상 등) 확인 등 방역에 협조하기

○ 수영복, 수건 및 휴대용 수상레저 용품은 개인물품을 사용하기

○ 수상레저 활동 시 다른 사람과 가급적 신체적 접촉이 없도록 주의하기

○ 수상레저기구 이용 시 사업자가 비치한 소독용품 등으로 기구표면 닦기

○ 수상레저기구 이용 시 가능하면 2m(최소 1m) 거리 두기가 유지되도록 띄어 앉기

○ 수상레저사업장(기구) 내에서 음식물 섭취를 가급적 자제하기

○ 탈의실(락커룸), 대기실(휴게실) 등 실내 공용공간에 머무르는 시간을 최소화하기

○ 칸막이가 없는 샤워실의 경우, 다른 사람과의 거리 유지를 위해 한 칸씩 띄워 사용하기

※ 수상레저사업장 및 시설 내 음식점·카페, 중소슈퍼, 공중화장실 등 이용 시 해당 유형의 지침을 준용

2 운영자·종사자

[공통사항]

- ○ 방역관리자 지정 및 지역 보건소 담당자의 연락망을 확보하는 등 방역 협력체계 구축하기
- ○ 공동체 내 밀접 접촉이 일어나는 동일 부서, 동일 장소 등에 2~3명 이상의 유증상자가 3~4일 내에 발생 시 유증상자가 코로나19 검사를 받도록 안내하며, 유증상자가 추가 발생 시 보건소에 집단감염 가능성을 신고하기
- ○ 종사자가 발열 또는 호흡기 증상이 있는 경우 출근 중단 및 즉시 퇴근 조치하기
- ○ 사람 간 간격을 2m(최소 1m) 이상 거리 두기
- ○ 손을 씻을 수 있는 시설 또는 손 소독제를 비치하고, 손 씻기 및 기침예절 준수 안내문 게시하기
- ○ 자연 환기가 가능한 경우 창문을 상시 열어두고, 에어컨 사용 등으로 상시적으로 창문을 열어두기 어려운 경우 2시간마다 1회 이상 환기하기
- ○ 공용으로 사용하는 물건(출입구 손잡이, 대여물품 등) 및 표면(기구 손잡이 등)은 매일 1회 이상 자주 소독하기
- ○ 고객(이용자)을 직접 응대하는 경우 마스크 착용하기
- ○ 발열 또는 호흡기 증상이 있거나 최근 14일 이내 해외여행을 한 경우 방문 자제 안내하기
- ○ 실내 다중이용시설을 이용하는 경우 마스크 착용 안내하기
- ○ 실외에서 2m 거리 유지가 안되는 경우 마스크 착용 안내하기(단, 물속에서는 사용 제외)

[해당 유형 적용사항]

- ○ 시간대별 이용객 수를 조정하기 위해 사전 예약제, 시간대별 이용인원 제한 등을 통해 이용객이 집중되지 않도록 하기
- ○ 사업장에 방문하는 이용객에 대한 마스크 착용 안내 및 증상 여부(발열, 호흡기 증상 등) 확인하기
- ○ 생활 속 거리 두기 및 개인 위생수칙 홍보(현수막, 문자전광판, 안내방송 등) 하기
- ○ 수영복, 수건, 물놀이용품(개인별 휴대가능용품) 등 개인물품 사용 안내하기
- ○ 밀폐된 장소에서 다수의 수강생을 대상으로 한 교육프로그램은 자제하되, 실시할 경우 2m(최소 1m) 거리 유지가 가능하도록 사용 인원을 관리하기
- ○ 2인 이상 수상레저기구 탑승 시 가능한 2m(최소 1m) 이상 거리를 두고 지그재그로 띄어 앉도록 안내하기

○ 시설 내 이용자 간 2m(최소 1m) 이상 거리 두기가 유지될 수 있도록 관리하기

○ 탈의실(락커룸), 대기실(휴게실) 등 부대시설의 손이 자주 닿는 표면은 매일 1회 이상 소독하기

○ 탈의실(락커룸), 샤워실, 대기실(휴게실) 등 공용시설은 2m(최소 1m) 거리 두기를 유지할 수 있도록 사용 인원을 줄이고, 머무르는 시간 최소화하도록 안내하기

○ 출입구 및 시설 내 곳곳에 휴지 및 뚜껑 있는 쓰레기통 비치하기

○ 안전요원에게 기본수칙 준수 확인(마스크 착용, 사회적 거리 유지 등)에 대한 업무를 중복 배정하지 않고, 다른 담당 직원에게 업무 배정하기

※ 수상레저사업장 및 시설 내 음식점·카페, 중소슈퍼, 공중화장실 등 이용 시 해당 유형의 지침을 준용

1-9. 생활 속 거리 두기 지침 : 지역축제

※ 불가피하게 행사를 개최하는 경우, 「코로나19 집단행사 방역관리 지침(2판, '20.02,26)」 및 「코로나19 관련 정부·지자체 행사 운영지침(2판, '20.02.26)」 등에 따른 방역 조치를 반드시 준수하고, 그 외 세부사항은 본 세부지침을 준용

1 참석자

[공통사항]

○ 발열 또는 호흡기 증상(기침, 인후통 등)이 있거나 최근 14일 이내 해외여행을 한 경우 방문 자제하기

○ 다른 사람과 2m(최소 1m) 이상 거리 두기

○ 흐르는 물과 비누로 30초 이상 손을 씻거나 손 소독제로 손 소독하기

○ 기침이나 재채기를 할 때는 휴지, 옷소매로 입과 코 가리기

○ 침방울이 튀는 행위(노래 부르기, 소리 지르기 등)나 신체접촉(악수, 포옹 등) 자제하기

○ 실내 다중이용시설을 이용하는 경우 마스크 착용하기

○ 실외에서 2m 거리 유지가 안 되는 경우 마스크 착용하기

[해당 유형 적용사항]

○ 가급적 온라인 또는 영상으로 참여하기

○ 불가피하게 현장 참여를 하는 경우 다른 사람과 2m(최소 1m)이상 거리 두기, 마스크 착용, 손 위생(물과 비누로 30초 이상 손 씻기, 손 소독제 사용) 등 개인 방역수칙 철저하게 준수하기

○ 축제 입장권 구매 시 현장 구매보다 사전 온라인 예매하기

○ 고위험군(65세 이상 어르신, 임신부, 만성질환자 등)은 가급적 현장 참여 자제하기

○ 행사장 출입 시 손 위생, 증상 여부(발열, 호흡기 증상 등) 확인 및 명부(전자 또는 수기) 기록 관리(4주 보관 후 폐기) 등 방역에 협조하기

○ 행사장 객석, 휴게 시설, 식당 이용 시 2m(최소 1m) 거리 유지가 가능하도록 지그재그로 한 칸 띄어 앉고 대화는 자제하기

○ 휴게 시설, 카페, 매점 등 다중이용공간이 밀집되지 않도록 2m(최소 1m) 거리가 유지되도록 분산하여 이용하기

○ 행사장에서 마스크 착용하고, 음식물 섭취 자제하기

○ 줄서기 등 대기할 때에는 사람 간 2m(최소 1m) 간격 두기

○ 행사장 내 셔틀버스 이용 시는 마스크를 착용하고, 다른 사람과 2m(최소 1m) 이상 거리가 유지되도록 한 칸씩 띄어 앉고 대화 자제하기

○ 행사 참여 중 발열, 호흡기 증상이 나타나면 보건용 마스크를 착용하고 지역축제 관계자에게 알리기

※ 축제 행사장 내 전시행사, 기념식, 음식점·카페, 공연장, 유원시설, 공중화장실 등 이용 시 해당 유형의 지침을 준용

2 책임자·종사자

[공통사항]

○ 방역관리자 지정 및 지역 보건소 담당자의 연락망을 확보하는 등 방역 협력체계 구축하기

○ 공동체 내 밀접 접촉이 일어나는 동일 부서, 동일 장소 등에 2~3명 이상의 유증상자가 3~4일 내에 발생 시 유증상자가 코로나19 검사를 받도록 안내하며, 유증상자가 추가 발생 시 보건소에 집단 감염 가능성을 신고하기

○ 종사자가 발열 또는 호흡기 증상이 있는 경우 출근 중단 및 즉시 퇴근 조치하기

○ 사람 간 간격을 2m(최소 1m) 이상 거리 두기

○ 손을 씻을 수 있는 시설 또는 손 소독제를 비치하고, 손 씻기 및 기침 예절 준수 안내문 게시하기

○ 자연 환기가 가능한 경우 창문을 상시 열어두고, 에어컨 사용 등으로 상시적으로 창문을 열어두기 어려운 경우 2시간마다 1회 이상 환기하기

○ 공용으로 사용하는 물건(출입구 손잡이 등) 및 표면은 매일 1회 이상 자주 소독하기

○ 고객(이용자)을 직접 응대하는 경우 마스크 착용하기

○ 발열 또는 호흡기 증상이 있거나 최근 14일 이내 해외여행을 한 경우 방문 자제 안내하기

○ 실내 다중이용시설을 이용하는 경우 마스크 착용 안내하기

○ 실외에서 2m 거리 유지가 안되는 경우 마스크 착용 안내하기

[해당 유형 적용사항]

○ 불가피하게 오프라인 축제를 개최하는 경우, 온라인·오프라인 병행, 사전 예약제,

시간제 운영 등의 방식을 활용하여 관람객이 한꺼번에 몰리지 않도록 분산 유도하기

○ 행사장이 밀집되지 않고 2m(최소 1m) 거리 두기가 유지되도록 입장 정원을 제한하기

* 예) 기념식인 경우 기념식장의 바닥면적 4㎡ 당 입장객 1명으로 제한, 행사장 수용인원의 50%로 입장 정원 제한 등 거리 두기가 가능한 방안 마련

- 대기자 발생 시 번호표를 활용하거나 대기자 간 2m(최소 1m) 간격을 두고 대기하도록 안내하기

○ 입장권은 현장 판매보다 사전예매 안내하기

○ 축제 관람객, 참가자, 관계자 등 출입하는 사람에 대한 증상 여부(발열, 호흡기 증상 등) 확인 및 명부(전자 또는 수기)를 기록 관리(4주 보관 후 폐기)하기

○ 입구와 출구를 달리하여 이용자들이 한 방향으로 이동하도록 동선을 마련하기

○ 공연, 체험행사 등은 실외 중심으로 운영하며 2m(최소 1m) 거리 두기가 가능하도록 탁상과 의자를 지그재그 등으로 배치하기

○ 실내에서 공연, 체험행사 등을 개최할 경우 마스크를 착용하고 음식물 섭취는 자제하며 충분히 환기하기

- 에어컨을 사용하는 경우 바람의 방향이 사람에게 직접 향하지 않도록 하고, 최소 2시간마다 1회 이상 환기
- 무대와 객석 간 사이에는 최대한 거리(최소 2m)를 유지하며, 공연 전·후로 객석과 무대 등 공연시설을 소독하기

○ 많은 사람이 일시에 한 장소에 집중될 수 있는 이벤트성 행사(선착순, 악수·사인회 등)는 자제하기

○ 침방울이 튀는 행위(노래부르기, 구호외치기 등)와 프로그램은 최소화하기

○ 마이크를 사용하는 경우 마이크는 커버를 씌우고, 개인마다 마이크 덮개를 새것으로 교체하기

○ 시식 및 홍보 부스(코너)는 운영을 중단하거나 최소화하되, 불가피하게 운영 시에는 부스 간에 2m 이상 간격을 유지하고, 참석자들이 한꺼번에 모이지 않도록 참석자 간에 2m(최소 1m) 유지 안내하기

- 음식은 각자 개인 접시에 덜어 먹도록 개인 접시와 국자, 집게 등을 제공하기
- 시식, 시음, 홍보 코너에서 발생하는 이쑤시개, 컵, 휴지 등 침이 묻을 수 있는 쓰레기는 타인의 손이 닿지 않도록 별도로 깨끗이 버릴 수 있게 조치하기
- 음식물은 정해진 장소에서만 섭취하기

○ 판매원과 이용객 사이 2m(최소 1m) 이상 거리를 유지할 수 있도록 하고, 필요 시 투명 가림막 등을 설치하기

○ 가능한 포장 판매 활성화 및 대규모 인원이 밀집되지 않도록 유도하기

○ 현장 결제 시 전자 결제방식(모바일 페이, QR코드, NFC카드, 신용카드 등)을 활용하도록 하기

○ 식당, 휴게실 등 다중이용시설 이용 시 일렬 또는 지그재그로 앉도록 자리를 배치하고, 2m(최소 1m) 거리가 유지되도록 분산 이용 안내하기

○ 생활 속 거리 두기(현수막, 문자 전광판, 안내방송 등) 및 개인 위생수칙 홍보·안내하기

○ 출입구 및 행사장 내 여러 곳에 휴지 및 뚜껑 있는 쓰레기통 비치하기

○ 단체식사 제공은 자제하며, 불가피한 경우에는 개인 도시락 형태로 제공하기

○ 해외 거주하는 인사들의 행사 초대는 자제하기

○ 매표원, 출입 관리요원 등은 수시로 손 소독제 사용하기

○ 진행요원 등의 식사 시간은 시차를 두고 운영하여 관계자 간 밀집을 최소화하기 (예: A조/18:30~19:30, B조/19:30~20:30)

○ 축제 진행요원 등에게 방역수칙 교육을 실시하고, 참여자에게 기본수칙(마스크 착용, 거리 유지 등) 준수 안내 업무를 수행하게 하기

○ 행사장 셔틀버스 운영 시, 마스크 착용 및 다른 사람과 2m(최소 1m) 이상 거리가 유지되도록 한 칸씩 띄어 앉고 대화 자제 안내하기

※ 축제 행사장 내 음식점·카페, 공연장, 유원시설, 공중화장실 등 이용 시 해당 유형의 지침을 준용

Ⅲ. 여 가 (2. 여가 등)

2-1. 생활 속 거리 두기 지침 : 야외 활동

[공통사항]

○ 발열 또는 호흡기 증상(기침, 인후통 등)이 있거나 최근 14일 이내 해외여행을 한 경우 방문 자제하기

○ 다른 사람과 2m(최소 1m) 이상 거리 두기

○ 흐르는 물과 비누로 30초 이상 손을 씻거나 손 소독제로 손 소독하기

○ 기침이나 재채기를 할 때는 휴지, 옷소매로 입과 코 가리기

○ 침방울이 튀는 행위(노래부르기, 소리지르기 등)나 신체접촉(악수, 포옹 등) 자제하기

○ 실내 다중이용시설을 이용하는 경우 마스크 착용하기

○ 실외에서 2m 거리 유지가 안되는 경우 마스크 착용하기

[해당 유형 적용사항]

○ 놀이공원, 관광지 등 입장권 구입 시 현장 구매보다 사전 예매하기

○ 많은 인원이 밀집한 장소는 출입 자제하기

○ 이동할 때에 맞은편에 오는 사람과 동선이 겹치지 않게 오른쪽으로 이동하기

2-2. 생활 속 거리 두기 지침 : 공중화장실 등

[1] 이용자

[공통사항]

○ 발열 또는 호흡기 증상(기침, 인후통 등)이 있거나 최근 14일 이내 해외여행을 한 경우 방문 자제하기

○ 다른 사람과 2m(최소 1m) 이상 거리 두기

○ 흐르는 물과 비누로 30초 이상 손을 씻거나 손 소독제로 손 소독하기

○ 기침이나 재채기를 할 때는 휴지, 옷소매로 입과 코 가리기

○ 침방울이 튀는 행위(노래부르기, 소리지르기 등)나 신체접촉(악수, 포옹 등) 자제하기

○ 실내 다중이용시설을 이용하는 경우 마스크 착용하기

○ 실외에서 2m 거리 유지가 안되는 경우 마스크 착용하기

[해당 유형 적용사항]

○ 다수가 이용하는 공간임을 고려하여 깨끗하게 사용하기를 철저히 준수하기

○ 대변기 칸 내 사용 시, 변기 뚜껑을 닫고 물 내리기

○ 사용 후 발생하는 휴지 등의 폐기물은 대변기에 흘려보내거나, 변기에 넣을 수 없는 위생용품은 위생용품 수거함에 버려 주변을 깨끗하게 유지하기

[2] 책임자·관리자

[공통사항]

○ 방역관리자 지정 및 지역 보건소 담당자의 연락망을 확보하는 등 방역 협력체계 구축하기

○ 공동체 내 밀접 접촉이 일어나는 동일 부서, 동일 장소 등에 2~3명 이상의 유증상자가 3~4일 내에 발생 시 유증상자가 코로나19 검사를 받도록 안내하며, 유증상자가 추가 발생 시 보건소에 집단감염 가능성을 신고하기

○ 종사자가 발열 또는 호흡기 증상이 있는 경우 출근 중단 및 즉시 퇴근 조치하기

○ 사람 간 간격을 2m(최소 1m) 이상 거리 두기

○ 손을 씻을 수 있는 시설 또는 손 소독제를 비치하고, 손 씻기 및 기침예절 준수 안내문 게시하기

○ 자연 환기가 가능한 경우 창문을 상시 열어두고, 에어컨 사용 등으로 상시적으로 창문을 열어두기 어려운 경우 2시간마다 1회 이상 환기하기

○ 공용으로 사용하는 물건(출입구 손잡이 등) 및 표면은 매일 1회 이상 자주 소독하기

○ 고객(이용자)을 직접 응대하는 경우 마스크 착용하기

○ 발열 또는 호흡기 증상이 있거나 최근 14일 이내 해외여행을 한 경우 방문 자제 안내하기

○ 실내 다중이용시설을 이용하는 경우 마스크 착용 안내하기

○ 실외에서 2m 거리 유지가 안되는 경우 마스크 착용 안내하기

[해당 유형 적용사항]

□ 소독 등 방역관리

○ 공중화장실 담당 부서장을 방역관리책임자로 지정하여 방역소독 철저히 지키기

□ 가급적이면 전문소독업체에 위탁 권고

○ 공중화장실 시설 내·외에 대해 소독 등 방역 수시로 실시하기

○ 스프레이를 사용하여 소독제를 분사하지 말기

○ 천(타올)에 소독제(알코올, 희석된 차아염소산나트륨(500~1000ppm)등)를 충분히 묻힌 후 신체접촉이 빈번하거나 자주 사용하는 모든 부위*를 지속적으로 닦기

* 출입문 손잡이, 스위치, 변기커버 및 뚜껑, 물내림버튼, 세면대, 수도꼭지, 손건조기, 기저귀교환대, 장애인손잡이 등

○ 바닥 한쪽 끝에서 다른 쪽 끝까지 반복하여 소독하기

- 소독 부위가 더러워지지 않도록 소독하지 않은 장소에서 소독한 장소로 이동하지 말 것

○ 소독 후에는 충분히 건조된 후 개방하기

○ 화장실 입구에 청소 또는 소독 중임을 알리는 안내표지판이나 안전띠를 두어 이용자가 알 수 있도록 하기

□ 위생 및 시설관리

○ 쓰레기 등이 장시간 방치되지 않도록 수시로 휴지통 및 위생용품 수거함 비우기 (휴지통 추가 비치)

○ 공중화장실 등 주요 설비(소변기 · 대변기 · 세면대 기저귀교환대 · 손건조기 등)의 주기적 청소 및 관리하기

○ 공중화장실 내 설비가 고장 나서 방치되지 않도록 수시로 점검 · 관리하기

◈ 환자 노출이 된 공중화장실 등은 본 안내서에 따라 소독을 실시 한 후 소독제의 종류별 특성 및 사용여건(주변 화장실 유무 등)을 고려하여 사용재개

※ 소독 이후 바이러스는 소독 당일 사멸하나 소독제 사용에 따른 위해 가능성 등을 고려

□ **홍보관리**

○ 공중화장실 이용자에 대한 적극적인 위생관리 홍보 실시하기

- 흐르는 물에 비누로 30초 이상 손 씻기, 화장실 깨끗하게 이용하기, 공중화장실 내 마스크 착용, 2m(최소 1m) 간격으로 줄서기, 변기 뚜껑 닫고 물 내리기, 의심증상자 이용 자제 요청 등 하기

- 화장실 줄서기 중 2m(최소 1m) 간격이 유지되도록 바닥 표시하기

  * 표시가 어려운 경우, 출입구 또는 바닥 등에 줄서기 간격 안내문구 부착 권고

- 출·입 동선이 겹치지 않도록 분리대 설치 (출입구 공간적 여유가 있는 경우 권고)하기

○ 공중화장실 등의 위생관리인 등에게 전달되도록 교육 · 홍보하기

  * 불필요한 집합교육은 지양하고, 문서 등을 통하여 추진

2-3. 생활 속 거리 두기 지침 : 이 · 미용업

1 이용자

[공통사항]

○ 발열 또는 호흡기 증상(기침, 인후통 등)이 있거나 최근 14일 이내 해외여행을 한 경우 방문 자제하기

○ 다른 사람과 2m(최소 1m) 이상 거리 두기

○ 흐르는 물과 비누로 30초 이상 손을 씻거나 손 소독제로 손 소독하기

○ 기침이나 재채기를 할 때는 휴지, 옷소매로 입과 코 가리기

○ 침방울이 튀는 행위(노래부르기, 소리지르기 등)나 신체접촉(악수, 포옹 등) 자제하기

○ 실내 다중이용시설을 이용하는 경우 마스크 착용하기

○ 실외에서 2m 거리 유지가 안되는 경우 마스크 착용하기

[해당 유형 적용사항]

○ 직원이 소독·청소·환기 등을 위해 퇴장을 요구하거나 해외여행력, 발열 또는 호흡기 등 증상 있어서 출입을 거부할 경우 협조하기

○ 출입 시 증상 여부(발열, 호흡기 증상 등) 확인 등 방역에 협조하기

2 책임자·종사자

[공통사항]

○ 방역관리자 지정 및 지역 보건소 담당자의 연락망을 확보하는 등 방역 협력체계 구축하기

○ 공동체 내 밀접 접촉이 일어나는 동일 부서, 동일 장소 등에 2~3명 이상의 유증상자가 3~4일 내에 발생 시 유증상자가 코로나19 검사를 받도록 안내하며, 유증상자가 추가 발생 시 보건소에 집단감염 가능성을 신고하기

○ 종사자가 발열 또는 호흡기 증상이 있는 경우 출근 중단 및 즉시 퇴근 조치하기

○ 사람 간 간격을 2m(최소 1m) 이상 거리 두기

○ 손을 씻을 수 있는 시설 또는 손 소독제를 비치하고, 손 씻기 및 기침예절 준수 안내문 게시하기

○ 자연 환기가 가능한 경우 창문을 상시 열어두고, 에어컨 사용 등으로 상시적으로 창문을 열어두기 어려운 경우 2시간마다 1회 이상 환기하기

○ 공용으로 사용하는 물건(출입구 손잡이 등) 및 표면은 매일 1회 이상 자주 소독하기

○ 고객(이용자)을 직접 응대하는 경우 마스크 착용하기

○ 발열 또는 호흡기 증상이 있거나 최근 14일 이내 해외여행을 한 경우 방문 자제 안내하기

○ 실내 다중이용시설을 이용하는 경우 마스크 착용 안내하기

○ 실외에서 2m 거리 유지가 안되는 경우 마스크 착용 안내하기

[해당 유형 적용사항]

○ 시설(의자, 침대 등) 간격을 2m(최소 1m) 이상으로 두거나, 1칸 건너 사용하기

○ 출입하는 사람에 대한 증상 여부(발열, 호흡기 증상 등) 확인하기

2-4. 생활 속 거리 두기 지침 : 목욕장업(목욕탕, 찜질방)

1 이용자

[공통사항]

- ○ 발열 또는 호흡기 증상(기침, 인후통 등)이 있거나 최근 14일 이내 해외여행을 한 경우 방문 자제하기
- ○ 다른 사람과 2m(최소 1m) 이상 거리 두기
- ○ 흐르는 물과 비누로 30초 이상 손을 씻거나 손 소독제로 손 소독하기
- ○ 기침이나 재채기를 할 때는 휴지, 옷소매로 입 가리기
- ○ 침방울이 튀는 행위(노래부르기, 소리지르기 등)나 신체접촉(악수, 포옹 등) 자제하기
- ○ 실내 다중이용시설을 이용하는 경우 마스크 착용하기(단, 물속에서는 제외)
- ○ 실외에서 2m 거리 유지가 안되는 경우 마스크 착용하기

[해당 유형 적용사항]

1 목욕탕, 사우나

- ○ 코로나19 임상증상(발열, 기침, 호흡곤란, 오한, 근육통, 두통, 인후통, 후각·미각 소실 등)이 있거나 의심증상자와 동거하는 자는 방문하지 않기
  - 고위험군(65세 이상 어르신, 임신부, 만성질환자 등)은 방문 자제하기
- ○ 출입 시 증상 여부(발열, 호흡기 증상 등) 확인, 청소·소독·환기 등을 위한 퇴장 요구 등 방역에 협조하기
- ○ 탈의실, 샤워실 등 공용시설을 이용하는 경우는 혼잡한 시간을 피하고, 대화는 자제하며, 다른 사람과 한 칸 떨어져 사용하는 등 거리 두기 유지하기
- ○ 목욕탕 외 탈의실 등에서는 마스크 착용하기
- ○ 목욕탕과 탈의실 내 음식 섭취 또는 대화하지 않기
- ○ 공용물품(드라이기, 빗, 로션 등)은 사용하지 않고, 가능한 개인용품 사용하기
- ○ 가급적 선풍기 등 사용하지 않기

2 찜질방

- ○ 코로나19 임상증상(발열, 기침, 호흡곤란, 오한, 근육통, 두통, 인후통, 후각·미각 소실 등)이 있거나 의심증상자와 동거하는 자는 방문하지 않기
  - 고위험군(65세 이상 어르신, 임신부, 만성질환자 등)은 방문 자제하기
- ○ 출입 시 증상 여부(발열, 호흡기 증상 등) 확인, 청소·소독·환기 등을 위한 퇴장 요구, 명부(전자 또는 수기) 작성 관리(4주 보관 후 폐기) 등 방역에 협조하기
- ○ 머무르는 시간 최소화하기
- ○ 공용공간 내에서는 다른 사람과 2m(최소 1m) 이상 거리를 유지하기
- ○ 목욕탕, 찜질방 외 공간에서는 마스크 착용하기
- ○ 공용물품(베개, 매트, 안마의자, TV 리모컨 등)은 비치된 소독제로 사용 전·후 표면 소독하기
- ○ 지정된 장소 외의 공간에서 음식 섭취하지 않기
  - 지정된 장소에서는 일렬 또는 지그재그로 앉아 대화하지 않고 섭취
- ○ 찜질방 내 부대시설(음식점, PC방, 오락실, 마사지실 등)은 가능한 이용 자제하기
  - 불가피한 경우 머무르는 시간 최소화, 마스크 착용, 손 소독, 대화하지 않기
  - 노래방 기기는 이용하지 않고, 유아·어린이는 pc방, 오락실, 놀이방 이용하지 않기
- ○ 오랜 시간 동안 마스크를 착용할 수 없고, 사람 간 거리 유지가 어려운 밀폐된 공간(수면실 등)은 가급적 이용하지 않기
- ○ 가급적 선풍기 등 사용하지 않기

※ 찜질방이 함께 있는 목욕탕은 1 목욕탕, 사우나 적용사항 준용

2 책임자·종사자

[공통사항]

- ○ 방역관리자 지정 및 지역 보건소 담당자의 연락망을 확보하는 등 방역 협력체계 구축하기
- ○ 공동체 내 밀접 접촉이 일어나는 동일 부서, 동일 장소 등에 2~3명 이상의 유증상자가 3~4일 내에 발생 시 유증상자가 코로나19 검사를 받도록 안내하며, 유증상자가 추가 발생 시 보건소에 집단감염 가능성을 신고하기
- ○ 종사자가 발열 또는 호흡기 증상이 있는 경우 출근 중단 및 즉시 퇴근 조치하기

○ 사람 간 간격을 2m(최소 1m) 이상 거리 두기
○ 손을 씻을 수 있는 시설 또는 손 소독제를 비치하고, 손 씻기 및 기침예절 준수 안내문 게시하기
○ 자연 환기가 가능한 경우 창문을 상시 열어두고, 에어컨 사용 등으로 상시적으로 창문을 열어두기 어려운 경우 2시간마다 1회 이상 환기하기
○ 공용으로 사용하는 물건(출입구 손잡이 등) 및 표면은 매일 1회 이상 자주 소독하기
○ 고객(이용자)을 직접 응대하는 경우 마스크 착용하기
○ 발열 또는 호흡기 증상이 있거나 최근 14일 이내 해외여행을 한 경우 방문 자제 안내하기
○ 실내 다중이용시설을 이용하는 경우 마스크 착용 안내하기(단, 물속에서는 제외)
○ 실외에서 2m 거리 유지가 안되는 경우 마스크 착용 안내하기

[해당 유형 적용사항]

1 목욕탕, 사우나

○ 출입하는 사람에 대한 증상 여부(발열, 호흡기 증상 등) 확인 등 방역 관리하기
○ 코로나19 임상증상(발열, 기침, 호흡곤란, 오한, 근육통, 두통, 인후통, 후각·미각소실 등)이 있거나 유증상자와 동거하는 자는 방문하지 않도록 안내하고, 고위험군은 이용을 자제할 것을 안내하기

* 고위험군: 65세 이상 어르신, 임신부, 만성질환자 등

○ 목욕탕 외 탈의실 등에서 마스크 착용하도록 안내하기
○ 개인 위생수칙 준수, 생활 속 거리 두기의 필요성 등에 대한 주기적 교육·안내하기
○ 이용자들이 시차를 두고 분산 이용하고 밀집을 최소화하는 방안 마련하기
-예) 시간 예약제, 이용자 간 2m(최소 1m) 거리 두기가 가능하도록 이용 인원 제한 등
○ 공용공간 내에서는 다른 사람과 2m(최소 1m) 이상 거리를 유지하도록 안내하기
○ 공용물품(드라이기, 빗, 로션 등)은 가능한 비치하지 않고, 개인용품 사용하도록 하기
○ 영업 전·후로 탈의실·휴게공간 등 환기 및 시설 소독하기(소독 관리대장 작성)
- 소독·청소·환기 시간을 미리 정하여 잘 보이는 곳에 안내하기
○ 가급적 비치된 선풍기 등은 사용하지 않도록 안내하기
○ 목욕탕과 탈의실 내 음식 섭취 또는 대화하지 않도록 안내하기

② 찜질방

○ 찜질방 내 사람 간 2m(최소 1m) 이상 거리 두기가 가능하도록 입장 인원 관리하기

* 예시: 바닥면적 4㎡당 1명 등

○ 출입하는 사람에 대한 증상 여부(발열, 호흡기 증상 등) 확인 및 명부(전자 또는 수기)를 작성 관리(4주 보관 후 폐기)하기

○ 코로나19 임상증상(발열, 기침, 호흡곤란, 오한, 근육통, 두통, 인후통, 후각·미각소실 등)이 있거나 유증상자와 동거하는 자는 방문하지 않도록 안내하고, 고위험군은 이용을 자제할 것을 안내하기

* 고위험군: 65세 이상 어르신, 임신부, 만성질환자 등

○ 목욕탕, 찜질방 외 탈의실 등에서는 마스크를 착용하도록 안내하기

○ 찜질방 내 곳곳에 손 소독제를 비치하고, 손 소독 안내하기

○ 음식 섭취 장소를 지정하고, 해당 장소 외에는 음식 섭취 금지 안내하기

- 지정된 장소에서는 일렬 또는 지그재그로 앉아 대화하지 않고 섭취하도록 안내하기

○ 찜질방 내 사용하는 베개, 매트는 개인별로 사용하도록 배부하기

- 1인 사용 후에는 회수하여 표면 소독하기

○ 이용자가 안마의자, tv 리모컨 등 사용 전후 소독할 수 있도록 소독제 비치하기

○ 영업 전·후로 탈의실·휴게공간 등 시설 소독(소독 관리대장 작성)하고, 찜질방 내 안마의자, tv 리모컨 등 공용으로 사용하는 물품은 자주 소독하기

○ 찜질방 내 부대시설(음식점, PC방, 오락실, 마사지실 등)은 가능한 운영 자제하기

- 불가피한 경우, **(음식점)** 머무르는 시간 최소화, 음식 섭취 전·후 마스크 착용, 대화 자제 안내하고, 손이 자주 닿는 표면(메뉴판 등)은 주기적 소독하기(1일 1회 이상)

- **(pc방, 오락실)** 마스크 지속 착용, 음식 섭취 금지, 대화자제 안내하고, 손이 자주 닿는 표면(키보드, 마우스, 게임기, 스틱기 등)은 자주 소독하기(1일 1회 이상)

- **(마사지실)** 종사자 마스크 착용, 손님과 대화 하지 않기, 손님도 가능한 경우 마스크 착용 안내하기, 마사지실 내 입장 인원 관리하여 밀집도 조절하기

- **(놀이방)** 공용 장난감 등 공용물품 비치하지 않기, 놀이기구의 표면(트럼플린 등)은 자주 소독하기(1일 1회 이상)

- **(노래방기기)** 노래방기기 등은 이용하지 않도록 안내하기

- **(유아·어린이)** pc방, 오락실, 놀이방 이용하지 않도록 안내하기

- 불가피한 경우 머무르는 시간 최소화, 마스크 착용, 손 소독, 대화하지 않기

○ 오랜 시간 동안 마스크를 착용할 수 없고, 사람 간 거리 유지가 어려운 밀폐된 공간(수면실 등)은 가급적 운영하지 않기

○ 가급적 비치된 선풍기 등은 사용하지 않도록 안내하기

※ 찜질방이 함께 있는 목욕탕은 [1] 목욕탕, 사우나 적용사항 준용

※ 시설 내 부대시설(음식점·카페, 공중화장실, PC방 등)은 해당 유형의 지침을 준용

2-5. 생활 속 거리 두기 지침 : 도서관

[1] 이용자

[공통사항]

- ○ 발열 또는 호흡기 증상(기침, 인후통 등)이 있거나 최근 14일 이내 해외여행을 한 경우 방문 자제하기
- ○ 다른 사람과 2m(최소 1m) 이상 거리 두기
- ○ 흐르는 물과 비누로 30초 이상 손을 씻거나 손 소독제로 손 소독하기
- ○ 기침이나 재채기를 할 때는 휴지, 옷소매로 입과 코 가리기
- ○ 침방울이 튀는 행위(큰소리 대화)나 신체접촉(악수, 포옹 등) 자제하기
- ○ 실내 다중이용시설을 이용하는 경우 마스크 착용하기
- ○ 실외에서 2m 거리 유지가 안되는 경우 마스크 착용하기

[해당 유형 적용사항]

- ○ 실내 휴게실, 카페, 매점 등 다중이용공간 밀집되지 않도록 분산하여 이용하기
- ○ 출입 시 증상 여부(발열, 호흡기 증상 등) 확인 등 방역에 협조하기

※ 시설 내 음식점·카페 등 이용 시 해당 유형의 지침을 준용

[2] 책임자·종사자

[공통사항]

- ○ 방역관리자 지정 및 지역 보건소 담당자의 연락망을 확보하는 등 방역 협력체계 구축하기
- ○ 공동체 내 밀접 접촉이 일어나는 동일 부서, 동일 장소 등에 2~3명 이상의 유증상자가 3~4일 내에 발생 시 유증상자가 코로나19 검사를 받도록 안내하며, 유증상자가 추가 발생 시 보건소에 집단감염 가능성을 신고하기
- ○ 종사자가 발열 또는 호흡기 증상이 있는 경우 출근 중단 및 즉시 퇴근 조치하기
- ○ 사람 간 간격을 2m(최소 1m) 이상 거리 두기
- ○ 손을 씻을 수 있는 시설 또는 손 소독제를 비치하고, 손 씻기 및 기침예절 준수 안내문 게시하기

○ 자연 환기가 가능한 경우 창문을 상시 열어두고, 에어컨 사용 등으로 상시적으로 창문을 열어두기 어려운 경우 2시간마다 1회 이상 환기하기

○ 공용으로 사용하는 물건·기기(출입구 손잡이, 컴퓨터 등)등 표면은 매일 1회 이상 자주 소독하기

○ 고객(이용자)을 직접 응대하는 경우 마스크 착용하기

○ 발열 또는 호흡기 증상이 있거나 최근 14일 이내 해외여행을 한 경우 방문 자제 안내하기

○ 실내 다중이용시설을 이용하는 경우 마스크 착용 안내하기

○ 실외에서 2m 거리 유지가 안되는 경우 마스크 착용 안내하기

[해당 유형 적용사항]

○ 시간대별 이용자 수 및 이용공간 제한 등을 통해 이용자 집중 방지하기

○ 온라인 서비스가 가능하도록 관련 장비와 교육과정 등을 갖춰 운영하기

○ 직원들 대면 최소화를 위해 식사 시간 등 차이 두기

○ 대규모 행사 및 공동 활동 등 자제하기

○ 교육·행사를 자제하되, 교육·행사를 실시하는 경우 참가자 안전거리 2m(최소 1m) 유지를 위한 방안(책상 간격 조정 등) 마련하기

○ 노트북, 테블릿 PC 등 전산용품 이용 시 직원은 가급적 개인기기 사용하기

○ 개인 위생수칙 준수, 생활 속 거리 두기의 필요성 등에 대한 주기적 교육 · 안내하기

○ 옆 자리와 앞 자리가 비도록 지그재그로 자리를 배치하거나 투명 가림막 등을 설치하기

○ 출입하는 사람에 대한 증상 여부(발열, 호흡기 증상 등) 확인하기

※ 시설 내 음식점·카페 등이 있는 경우 해당 유형의 지침을 준용

2-6. 생활 속 거리 두기 지침 : 공연장

1 이용자·관람객

[공통사항]

○ 발열 또는 호흡기 증상(기침, 인후통 등)이 있거나 최근 14일 이내 해외여행을 한 경우 방문 자제하기

○ 다른 사람과 2m(최소 1m) 이상 거리 두기

○ 흐르는 물과 비누로 30초 이상 손을 씻거나 손 소독제로 손 소독하기

○ 기침이나 재채기를 할 때는 휴지, 옷소매로 입과 코 가리기

○ 침방울이 튀는 행위(노래부르기, 소리지르기 등)나 신체접촉(악수, 포옹 등) 자제하기

○ 실내 다중이용시설을 이용하는 경우 마스크 착용하기

○ 실외에서 2m 거리 유지가 안되는 경우 마스크 착용하기

[해당 유형 적용사항]

○ 입장권 구매 시 가급적 온라인 사전 예매하기

○ 시간을 충분히 두고 도착하여 천천히 입장하기

○ 실내 휴게실, 카페, 매점 등 다중이용공간 밀집되지 않도록 분산하여 이용하기

○ 공연 관람 시 좌석은 지그재그로 한 칸 띄워 앉도록 예매하며 착석하기

○ 공연장 내 마스크 착용 및 음식물 섭취 자제하기

○ 출입 시 증상 여부(발열, 호흡기 증상 등) 확인 등 방역에 협조하기

※ 시설 내 음식점·카페 등 이용 시 해당 유형의 지침을 준용

2 책임자·종사자

[공통사항]

○ 방역관리자 지정 및 지역 보건소 담당자의 연락망을 확보하는 등 방역 협력체계 구축하기

○ 공동체 내 밀접 접촉이 일어나는 동일 부서, 동일 장소 등에 2~3명 이상의 유증상자가 3~4일 내에 발생 시 유증상자가 코로나19 검사를 받도록 안내하며, 유증상자가 추가 발생 시 보건소에 집단감염 가능성을 신고하기

○ 종사자가 발열 또는 호흡기 증상이 있는 경우 출근 중단 및 즉시 퇴근 조치하기
○ 사람 간 간격을 2m(최소 1m) 이상 거리 두기
○ 손을 씻을 수 있는 시설 또는 손 소독제를 비치하고, 손 씻기 및 기침예절 준수 안내문 게시하기
○ 자연 환기가 가능한 경우 창문을 상시 열어두고, 에어컨 사용 등으로 상시적으로 창문을 열어두기 어려운 경우 2시간마다 1회 이상 환기하기
○ 공용으로 사용하는 물건(출입구 손잡이 등) 및 표면은 매일 1회 이상 자주 소독하기
○ 고객(이용자)을 직접 응대하는 경우 마스크 착용하기
○ 발열 또는 호흡기 증상이 있거나 최근 14일 이내 해외여행을 한 경우 방문 자제 안내하기
○ 실내 다중이용시설을 이용하는 경우 마스크 착용 안내하기
○ 실외에서 2m 거리 유지가 안되는 경우 마스크 착용 안내하기

[해당 유형 적용사항]

○ 매표원은 수시로 손세정제(위생장갑 착용 시 수시로 교체) 사용하기
○ 무대와 객석 간 사이에는 최대한 거리 유지하기(최소 2m)
○ 매회 공연 후, 공연자와 관람객 간 악수, 포옹, 기념촬영 등 신체접촉 금하도록 사전에 안내하기
○ 공연 후 충분히 환기를 실시하고 객석, 무대 등 공연장 시설은 반드시 소독하기 (소독 관리대장 작성)
○ 출입구 및 시설 내 곳곳에 휴지 및 뚜껑 있는 쓰레기통 비치하기
○ 개인 위생수칙 준수, 생활 속 거리 두기의 필요성 등에 대한 주기적 교육·안내하기
○ 공연 입장 시 밀집되지 않도록 시간을 두고 천천히 입장시키기
○ 이동할 때나 줄을 설 때 등의 경우 다른 사람과 2m(최소 1m) 이상 간격을 두도록 유도할 수 있는 바닥 스티커 등 조치하기
○ 실내 휴게실, 카페, 매점 등 다중이용공간은 이용자가 밀집되지 않도록 관리하기
○ 좌석은 지그재그로 한 칸 띄워 앉도록 예매 진행하며 착석 안내하기
○ 공연장 내 마스크 착용 및 음식물 섭취 자제 안내하기
○ 출입하는 사람에 대한 증상 여부(발열, 호흡기 증상 등) 확인하기

※ 시설 내 음식점·카페 등이 있는 경우 해당 유형의 지침을 준용

2-7. 생활 속 거리 두기 지침 : 영화상영관

1 이용자·관람객

[공통사항]

- ○ 발열 또는 호흡기 증상(기침, 인후통 등)이 있거나 최근 14일 이내 해외여행을 한 경우 방문 자제하기
- ○ 다른 사람과 2m(최소 1m) 이상 거리 두기
- ○ 흐르는 물과 비누로 30초 이상 손을 씻거나 손 소독제로 손 소독하기
- ○ 기침이나 재채기를 할 때는 휴지, 옷소매로 입과 코 가리기
- ○ 침방울이 튀는 행위(노래부르기, 소리지르기 등)나 신체접촉(악수, 포옹 등) 자제하기
- ○ 실내 다중이용시설을 이용하는 경우 마스크 착용하기
- ○ 실외에서 2m 거리 유지가 안되는 경우 마스크 착용하기

[해당 유형 적용사항]

- ○ 입장권 구매 시 가급적 온라인 사전 예매하기
- ○ 시간을 충분히 두고 도착하여 천천히 입장하기
- ○ 실내 휴게실, 카페, 매점 등 다중이용공간 밀집되지 않도록 분산하여 이용하기
- ○ 영화 관람 시 좌석은 지그재그로 한 칸 띄워 앉도록 예매하며 착석하기
- ○ 영화상영관 내 마스크 착용 및 음식물 섭취 자제하기
- ○ 출입 시 증상 여부(발열, 호흡기 증상 등) 확인 등 방역에 협조하기

※ 시설 내 음식점·카페, 공중화장실, 에어컨 등 이용 시 해당 유형의 지침을 준용

2 책임자·종사자

[공통사항]

- ○ 방역관리자 지정 및 지역 보건소 담당자의 연락망을 확보하는 등 방역 협력체계 구축하기
- ○ 공동체 내 밀접 접촉이 일어나는 동일 부서, 동일 장소 등에 2~3명 이상의 유증상자가 3~4일 내에 발생 시 유증상자가 코로나19 검사를 받도록 안내하며, 유증상자가 추가 발생 시 보건소에 집단감염 가능성을 신고하기

○ 종사자가 발열 또는 호흡기 증상이 있는 경우 출근 중단 및 즉시 퇴근 조치하기
○ 사람 간 간격을 2m(최소 1m) 이상 거리 두기
○ 손을 씻을 수 있는 시설 또는 손 소독제를 비치하고, 손 씻기 및 기침예절 준수 안내문 게시하기
○ 자연 환기가 가능한 경우 창문을 상시 열어두고, 에어컨 사용 등으로 상시적으로 창문을 열어두기 어려운 경우 2시간마다 1회 이상 환기하기
○ 공용으로 사용하는 물건(출입구 손잡이 등) 및 표면은 매일 1회 이상 자주 소독하기
○ 고객(이용자)을 직접 응대하는 경우 마스크 착용하기
○ 발열 또는 호흡기 증상이 있거나 최근 14일 이내 해외여행을 한 경우 방문 자제 안내하기
○ 실내 다중이용시설을 이용하는 경우 마스크 착용 안내하기
○ 실외에서 2m 거리 유지가 안되는 경우 마스크 착용 안내하기

[해당 유형 적용사항]

○ 매표원은 수시로 손세정(위생장갑 착용시 수시로 교체) 사용하기
○ 상영 후 충분히 환기를 실시하고 좌석 팔걸이 등 손이 자주 닿는 부분은 반드시 소독하기
○ 대규모 인원참석 홍보(프로모션) 행사 자제하기
○ 개인 위생수칙 준수, 생활 속 거리 두기의 필요성 등에 대한 주기적 교육·안내하기
○ 상영관 입장 시 밀집되지 않도록 시간을 두고 천천히 입장시키기
○ 이동할 때나 줄을 설 때 등의 경우 다른 사람과 2m(최소 1m) 이상 간격을 두도록 유도할 수 있는 바닥 스티커 등 조치하기
○ 실내 휴게실, 카페, 매점 등 다중이용공간은 이용자가 밀집되지 않도록 관리하기
○ 좌석은 지그재그로 한 칸 띄워 앉도록 예매 진행하며 착석 안내하기
○ 영화상영관 내 마스크 착용 및 음식물 섭취 자제 안내하기
○ 출입하는 사람에 대한 증상 여부(발열, 호흡기 증상 등) 확인하기

※ 시설 내 음식점·카페, 공중화장실, 에어컨 등이 있는 경우 해당 유형의 지침을 준용

2-8. 생활 속 거리 두기 지침 : 박물관 · 미술관

1 이용자·관람객

[공통사항]

- ○ 발열 또는 호흡기 증상(기침, 인후통 등)이 있거나 최근 14일 이내 해외여행을 한 경우 방문 자제하기
- ○ 다른 사람과 2m(최소 1m) 이상 거리 두기
- ○ 흐르는 물과 비누로 30초 이상 손을 씻거나 손 소독제로 손 소독하기
- ○ 기침이나 재채기를 할 때는 휴지, 옷소매로 입과 코 가리기
- ○ 침방울이 튀는 행위(노래부르기, 소리지르기 등)나 신체접촉(악수, 포옹 등) 자제하기
- ○ 실내 다중이용시설을 이용하는 경우 마스크 착용하기
- ○ 실외에서 2m 거리 유지가 안되는 경우 마스크 착용하기

[해당 유형 적용사항]

- ○ 전시 관람, 이동할 때나 줄을 설 때 등 다른 사람과 2m(최소 1m) 이상 거리 두기
- ○ 실내 휴게실, 카페, 매점 등 다중이용공간 밀집되지 않도록 분산하여 이용하기

※ 시설 내 음식점·카페, 공연장, 공중화장실 등 이용 시 해당 유형의 지침을 준용

2 책임자·종사자

[공통사항]

- ○ 방역관리자 지정 및 지역 보건소 담당자의 연락망을 확보하는 등 방역 협력체계 구축하기
- ○ 공동체 내 밀접 접촉이 일어나는 동일 부서, 동일 장소 등에 2~3명 이상의 유증상자가 3~4일 내에 발생 시 유증상자가 코로나19 검사를 받도록 안내하며, 유증상자가 추가 발생 시 보건소에 집단감염 가능성을 신고하기
- ○ 종사자가 발열 또는 호흡기 증상이 있는 경우 출근 중단 및 즉시 퇴근 조치하기
- ○ 사람 간 간격을 2m(최소 1m) 이상 거리 두기
- ○ 손을 씻을 수 있는 시설 또는 손 소독제를 비치하고, 손 씻기 및 기침예절 준수 안내문 게시하기

○ 자연 환기가 가능한 경우 창문을 상시 열어두고, 에어컨 사용 등으로 상시적으로 창문을 열어두기 어려운 경우 2시간마다 1회 이상 환기하기

○ 공용으로 사용하는 물건(출입구 손잡이 등) 및 표면은 매일 1회 이상 자주 소독하기

○ 고객(이용자)을 직접 응대하는 경우 마스크 착용하기

○ 발열 또는 호흡기 증상이 있거나 최근 14일 이내 해외여행을 한 경우 방문 자제 안내하기

○ 실내 다중이용시설을 이용하는 경우 마스크 착용 안내하기

○ 실외에서 2m 거리 유지가 안되는 경우 마스크 착용 안내하기

[해당 유형 적용사항]

○ 온라인 서비스가 가능하도록 관련 장비와 교육과정 등을 갖춰 운영하기

○ 관람객과 신체접촉을 피하고 거리 2m(최소 1m) 이상 유지할 수 있도록 직원 행동지침 마련하기

○ 출입구 및 시설 내 곳곳에 휴지 및 뚜껑 있는 쓰레기통 비치하기

○ 교육·행사를 자제하기

○ 노트북, 테블릿PC 등 전산용품 이용 시 직원은 가급적 개인기기 사용하기

○ 직원 휴게실, 탈의실 등 공용구역 사용시간 차이 두기

○ 증상이 나타난 이용자·종사자 격리 공간 마련, 의심환자 발생 시 행동수칙을 마련하여 유사시 대비하기

○ 개인 위생수칙 준수, 생활 속 거리 두기의 필요성 등에 대한 주기적 교육・안내하기

○ 예약제도 운영 등 시간대별 관람객 수를 제한하여 관람객 집중 방지하기

※ 시설 내 음식점·카페, 공연장, 공중화장실 등이 있는 경우 해당 유형의 지침을 준용

2-9. 생활 속 거리 두기 지침 : 야구장 · 축구장

1 이용자·관람객

[공통사항]

- ○ 발열 또는 호흡기 증상(기침, 인후통 등)이 있거나 최근 14일 이내 해외여행을 한 경우 방문 자제하기
- ○ 다른 사람과 2m(최소 1m) 이상 거리 두기
- ○ 흐르는 물과 비누로 30초 이상 손을 씻거나 손 소독제로 손 소독하기
- ○ 기침이나 재채기를 할 때는 휴지, 옷소매로 입과 코 가리기
- ○ 침방울이 튀는 행위(노래부르기, 소리지르기 등)나 신체접촉(악수, 포옹 등) 자제하기
- ○ 실내 다중이용시설을 이용하는 경우 마스크 착용하기
- ○ 실외에서 2m 거리 유지가 안되는 경우 마스크 착용하기

[해당 유형 적용사항]

- ○ 입장권 구매 시 가급적 온라인 사전 예매하기
- ○ 경기장 내 모여서 음식물 취식 자제하기
- ○ 운동용품, 응원도구, 운동복 및 수건 등은 개인물품 사용하기
- ○ 탈의실, 샤워실 등 공용시설 이용 자제하기
- ○ 경기 관람 시 좌석은 지그재그로 한 칸 띄워 앉도록 예매하며 착석하기

※ 시설 내 음식점·카페 등이 있는 경우 해당 유형의 지침을 준용

※ 기타 경기 관람을 수반하는 각종 체육시설에서는 동 지침 준용

2 책임자·종사자

[공통사항]

- ○ 방역관리자 지정 및 지역 보건소 담당자의 연락망을 확보하는 등 방역 협력체계 구축하기
- ○ 공동체 내 밀접 접촉이 일어나는 동일 부서, 동일 장소 등에 2~3명 이상의 유증상자가 3~4일 내에 발생 시 유증상자가 코로나19 검사를 받도록 안내하며, 유증상자가 추가 발생 시 보건소에 집단감염 가능성을 신고하기

○ 종사자가 발열 또는 호흡기 증상이 있는 경우 출근 중단 및 즉시 퇴근 조치하기

○ 사람 간 간격을 2m(최소 1m) 이상 거리 두기

○ 손을 씻을 수 있는 시설 또는 손 소독제를 비치하고, 손 씻기 및 기침예절 준수 안내문 게시하기

○ 자연 환기가 가능한 경우 창문을 상시 열어두고, 에어컨 사용 등으로 상시적으로 창문을 열어두기 어려운 경우 2시간마다 1회 이상 환기하기

○ 공용으로 사용하는 물건(출입구 손잡이 등) 및 표면은 매일 1회 이상 자주 소독하기

○ 고객(이용자)을 직접 응대하는 경우 마스크 착용하기

○ 발열 또는 호흡기 증상이 있거나 최근 14일 이내 해외여행을 한 경우 방문 자제 안내하기

○ 실내 다중이용시설을 이용하는 경우 마스크 착용 안내하기

○ 실외에서 2m 거리 유지가 안되는 경우 마스크 착용 안내하기

[해당 유형 적용사항]

○ 선수단 숙소 체류 시 수시 발열검사 및 문진 실시하기

○ 증상이 나타난 이용자·종사자 독립된 공간 마련하기

○ 화장실 등 공동이용시설 등 경기장 내·외부, 선수단 락커, 숙소 등 손이 많이 닿는 곳 소독 철저히 하기

○ 개인위생수칙 준수, 생활 속 거리 두기의 필요성 등에 대한 교육 및 홍보(전광판 홍보, 안내요원 교육 등)하기

○ 입장권 현장 판매보다 사전예매 독려, 구역별 입·퇴장 시간 구분, 공용구역 밀집 방지를 위한 동선 관리 등 이용객 분산 유도하기

○ 사람 간 접촉을 유도하는 행위 및 행사(손뼉맞장구(하이파이브), 사인회, 악수회 등) 자제하기

○ 시설 내 곳곳에 휴지와 뚜껑이 있는 쓰레기통을 비치해 기침이나 재채기 후 사용한 휴지를 깨끗이 버릴 수 있도록 하기

○ 좌석은 지그재그로 한 칸 띄워 앉도록 예매 진행하며 착석 안내하기

※ 시설 내 음식점·카페 등이 있는 경우 해당 유형의 지침을 준용

※ 기타 경기 관람을 수반하는 각종 체육시설에서는 동 지침 준용

2-10. 생활 속 거리 두기 지침 : 노래연습장

1 이용자

[공통사항]

○ 발열 또는 호흡기 증상(기침, 인후통 등)이 있거나 최근 14일 이내 해외여행을 한 경우 방문 자제하기

○ 다른 사람과 2m(최소 1m) 이상 거리 두기

○ 흐르는 물과 비누로 30초 이상 손을 씻거나 손 소독제로 손 소독하기

○ 기침이나 재채기를 할 때는 휴지, 옷소매로 입과 코 가리기

○ 침방울이 튀는 행위나 신체접촉(악수, 포옹 등) 자제하기

○ 실내 다중이용시설을 이용하는 경우 마스크 착용하기

○ 실외에서 2m 거리 유지가 안되는 경우 마스크 착용하기

[해당 유형 적용사항]

○ 노래연습장 내에서 마스크 착용하기(노래 부르지 않는 경우 착용)

○ 마이크는 커버를 씌우고 개인별로 사용하기

○ 고위험군은 시설이용 자제, 불가피하게 방문할 경우 마스크 착용하기

* 고위험군: 65세 이상 어르신, 임신부, 만성질환자 등

○ 출입 시 증상 여부(발열, 호흡기 증상 등) 확인 및 명부(전자 또는 수기) 기록 관리(4주 보관 후 폐기) 등 방역에 협조하기

○ 노래연습장 내에서 음식물 섭취를 최소화하고 대화 자제하기

※ 시설 내 음식점·카페 등이 있는 경우 해당 유형의 지침을 준용

2 책임자·종사자

[공통사항]

○ 방역관리자 지정 및 지역 보건소 담당자의 연락망을 확보하는 등 방역 협력체계 구축하기

○ 공동체 내 밀접 접촉이 일어나는 동일 부서, 동일 장소 등에 2~3명 이상의

유증상자가 3~4일 내에 발생 시 유증상자가 코로나19 검사를 받도록 안내하며, 유증상자가 추가 발생 시 보건소에 집단감염 가능성을 신고하기

○ 종사자가 발열 또는 호흡기 증상이 있는 경우 출근 중단 및 즉시 퇴근 조치하기

○ 사람 간 간격을 2m(최소 1m) 이상 거리 두기

○ 손을 씻을 수 있는 시설 또는 손 소독제를 비치하고, 손 씻기 및 기침예절 준수 안내문 게시하기

○ 자연 환기가 가능한 경우 창문을 상시 열어두고, 에어컨 사용 등으로 상시적으로 창문을 열어두기 어려운 경우 2시간마다 1회 이상 환기하기

○ 공용으로 사용하는 물건(출입구 손잡이, 마이크, 리모콘 등) 및 표면은 매일 1회 이상 자주 소독하기

○ 고객(이용자)을 직접 응대하는 경우 마스크 착용하기

○ 발열 또는 호흡기 증상이 있거나 최근 14일 이내 해외여행을 한 경우 방문 자제 안내하기

○ 실내 다중이용시설을 이용하는 경우 마스크 착용 안내하기

○ 실외에서 2m 거리 유지가 안되는 경우 마스크 착용 안내하기

[해당 유형 적용사항]

○ 출입구 및 시설 내 곳곳에 휴지 및 뚜껑 있는 쓰레기통 비치하기

○ 개인 위생수칙 준수, 생활 속 거리 두기의 필요성 등에 대한 주기적 교육·안내하기

○ 마이크 커버를 충분히 비치하기

○ 실내 휴게실, 카페, 매점 등 다중이용공간은 이용자가 밀집되지 않도록 관리하기

○ 고위험군은 시설이용 자제, 불가피하게 방문할 경우 마스크 착용 안내하기

* 고위험군: 65세 이상 어르신, 임신부, 만성질환자 등

○ 출입하는 사람에 대한 증상 여부(발열, 호흡기 증상 등) 확인 및 명부(전자 또는 수기)를 작성 관리(4주 보관 후 폐기)하기

○ 손님이 이용한 룸은 분무기 등으로 물을 뿌린 후 문을 닫고, 30분 뒤 소독 실시 후 재사용하기(대장작성)

※ 시설 내 음식점·카페 등이 있는 경우 해당 유형의 지침을 준용

2-11. 생활 속 거리 두기 지침 : 실내체육시설

1 이용자

[공통사항]

- ○ 발열 또는 호흡기 증상(기침, 인후통 등)이 있거나 최근 14일 이내 해외여행을 한 경우 방문 자제하기
- ○ 다른 사람과 2m(최소 1m) 이상 거리 두기
- ○ 흐르는 물과 비누로 30초 이상 손을 씻거나 손 소독제로 손 소독하기
- ○ 기침이나 재채기를 할 때는 휴지, 옷소매로 입과 코 가리기
- ○ 침방울이 튀는 행위(노래부르기, 소리지르기 등)나 신체접촉(악수, 포옹 등) 자제하기
- ○ 실내 다중이용시설을 이용하는 경우 마스크 착용하기
- ○ 실외에서 2m 거리 유지가 안되는 경우 마스크 착용하기

[해당 유형 적용사항]

- ○ 운동복, 수건 및 휴대용 운동기구 등은 개인물품을 사용권고하기
- ○ 탈의실, 샤워실 등 공용시설 이용 자제하기
- ○ 운동기구를 이용한 후에 운영자가 비치한 소독용품 등으로 기구표면 닦기
- ○ 고위험군은 시설이용 자제, 불가피하게 방문할 경우 마스크 착용하기
  - * 고위험군: 65세 이상 어르신, 임신부, 만성질환자 등
- ○ 출입 시 증상 여부(발열, 호흡기 증상 등) 확인 및 명부(전자 또는 수기) 기록 관리 (4주 보관 후 폐기) 등 방역에 협조하기

※ '실외 체육시설'은 동 지침 준용

2 책임자·종사자

[공통사항]

- ○ 방역관리자 지정 및 지역보건소 담당자의 연락망을 확보하는 등 방역 협력체계 구축하기
- ○ 공동체 내 밀접 접촉이 일어나는 동일 부서, 동일 강습, 동일 장소 등에 2~3명 이상의 유증상자가 3~4일 내에 발생 시 유증상자가 코로나19 검사를 받도록

안내하며, 유증상자가 추가 발생 시 보건소에 집단감염 가능성을 신고하기

○ 종사자가 발열 또는 호흡기 증상이 있는 경우 출근 중단 및 즉시 퇴근 조치하기

○ 사람 간 간격을 2m(최소 1m) 이상 거리 두기

○ 손을 씻을 수 있는 시설 또는 손 소독제를 비치하고, 손 씻기 및 기침예절 준수 안내문 게시하기

○ 자연 환기가 가능한 경우 창문을 상시 열어두고, 에어컨 사용 등으로 상시적으로 창문을 열어두기 어려운 경우 2시간마다 1회 이상 환기하기

○ 공용으로 사용하는 물건(출입구 손잡이 등) 및 표면은 매일 1회 이상 자주 소독하기

○ 고객(이용자)을 직접 응대하는 경우 마스크 착용하기

○ 발열 또는 호흡기 증상이 있거나 최근 14일 이내 해외여행을 한 경우 방문 자제 안내하기

○ 실내 다중이용시설을 이용하는 경우 마스크 착용 안내하기

○ 실외에서 2m 거리 유지가 안되는 경우 마스크 착용 안내하기

[해당 유형 적용사항]

○ 체육지도자, 강습자 및 이용자 마스크 착용 지도 및 신체접촉 자제하기

○ 운동복, 수건, 운동장비(개인별 휴대가능용품) 등 개인물품 사용 권고, 공용물품 제공 시에는 소독 철저히 하기

○ 탈의실(락커룸), 샤워실, 대기실(휴게실) 등 부대시설 소독 철저히 하기

○ 개인위생수칙 준수, 생활 속 거리 두기의 필요성 등에 대한 주기적으로 교육 및 홍보하기

○ 시설 내 이용자 간 2m(최소 1m) 이상 적정 간격 유지 될 수 있도록 입장 인원 관리하기

○ 탈의실(락커룸), 샤워실, 대기실 등 부대시설 적정 사용 인원 관리하기

○ 어린이통학버스 내 전원 마스크 착용, 탑승 전후 소독 및 환기 실시하기

○ 밀폐된 장소에서 다수의 수강생을 대상으로 한 운동프로그램(GX류) 자제하되, 실시할 경우 적정 간격 유지(2m) 등 방역 철저히 지키기

* GX(Group Exercise): 줌바, 태보, 스피닝 등

○ 고위험군은 시설이용 자제, 불가피하게 방문할 경우 마스크 착용 안내하기

* 고위험군: 65세 이상 어르신, 임신부, 만성질환자 등

○ 출입하는 사람에 대한 증상 여부(발열, 호흡기 증상 등) 확인 및 명부(전자 또는 수기)를 작성 관리(4주 보관 후 폐기)하기

※ ‘실외 체육시설’은 동 지침 준용

2-12. 생활 속 거리 두기 지침 : 수영장

1 이용자

[공통사항]

○ 발열 또는 호흡기 증상(기침, 인후통 등)이 있거나 최근 14일 이내 해외여행을 한 경우 방문 자제하기

○ 다른 사람과 2m(최소 1m) 이상 거리 두기

○ 흐르는 물과 비누로 30초 이상 손을 씻거나 손 소독제로 손 소독하기

○ 기침이나 재채기를 할 때는 휴지, 옷소매로 입과 코 가리기

○ 침방울이 튀는 행위(구령외치기, 소리지르기 등)나 신체접촉(악수, 포옹 등) 자제하기

○ 실내 다중이용시설을 이용하는 경우 마스크 착용하기(단, 물속에서는 사용 제외)

○ 실외에서 2m 거리 유지가 안되는 경우 마스크 착용하기(단, 물속에서는 사용 제외)

[해당 유형 적용사항]

○ 가족 단위·소규모로 방문하고 동호회, 단체모임 등 많은 인원이 함께 방문하는 것은 자제하기

○ 고위험군은 시설이용 자제, 불가피하게 방문할 경우 마스크 착용하기

* 고위험군: 65세 이상 어르신, 임신부, 만성질환자 등

○ 수영복, 수경 및 수건 등 신체에 접촉하는 물품은 개인물품 사용하기

○ 수영장 내(물 속 포함)에서 다른 사람과 2m(최소 1m) 이상 간격을 유지하고 이용자 간 신체접촉 및 대화는 자제하기

○ 탈의실(락커룸), 샤워실, 대기실 등 공용시설 사용 시 다른 사람과 거리를 유지하고 대화는 자제하며 머무르는 시간을 최소화하기

- 탈의실(락커룸)은 거리 두기가 가능하도록 한 칸씩 띄워 사용하기
- 칸막이가 없는 샤워실의 경우, 다른 사람과의 거리 유지를 위해 한 칸씩 띄워 사용하기

○ 출입 시 증상 여부(발열, 호흡기 증상 등) 확인 및 명부(전자 또는 수기) 기록 관리 (4주 보관 후 폐기) 등 방역에 협조하기

※ 시설 내 실내체육시설, 음식점·카페, 공중화장실 등 이용 시 해당 유형의 지침을 준용

2 책임자·종사자

[공통사항]

○ 방역관리자 지정 및 지역 보건소 담당자의 연락망을 확보하는 등 방역 협력체계 구축하기

○ 공동체 내 밀접 접촉이 일어나는 동일 부서, 동일 장소 등에 2~3명 이상의 유증상자가 3~4일 내에 발생 시 유증상자가 코로나19 검사를 받도록 안내하며, 유증상자가 추가 발생 시 보건소에 집단감염 가능성을 신고하기

○ 종사자가 발열 또는 호흡기 증상이 있는 경우 출근 중단 및 즉시 퇴근 조치하기

○ 사람 간 간격을 2m(최소 1m) 이상 거리 두기

○ 손을 씻을 수 있는 시설 또는 손 소독제를 비치하고, 손 씻기 및 기침예절 준수 안내문 게시하기

○ 자연 환기가 가능한 경우 창문을 상시 열어두고, 에어컨 사용 등으로 상시적으로 창문을 열어두기 어려운 경우 2시간마다 1회 이상 환기하기

○ 공용으로 사용하는 물건(출입구 손잡이 등) 및 표면은 매일 1회 이상 자주 소독하기

○ 고객(이용자)을 직접 응대하는 경우 마스크 착용하기

○ 발열 또는 호흡기 증상이 있거나 최근 14일 이내 해외여행을 한 경우 방문 자제 안내하기

○ 실내 다중이용시설을 이용하는 경우 마스크 착용 안내하기(단, 물속에서는 사용 제외)

○ 실외에서 2m 거리 유지가 안되는 경우 마스크 착용 안내하기(단, 물속에서는 사용 제외)

[해당 유형 적용사항]

○ 예약제도, 이용 시간제 등으로 이용자 간 2m(최소 1m) 이상 거리 두기가 가능하도록 수영장 이용자가 한꺼번에 몰리지 않도록 하기

○ 고위험군은 시설이용 자제, 불가피하게 방문할 경우 마스크 착용 안내하기

* 고위험군: 65세 이상 어르신, 임신부, 만성질환자 등

○ 출입하는 사람에 대한 증상 여부(발열, 호흡기 증상 등) 확인 및 명부(전자 또는 수기)를 작성 관리(4주 보관 후 폐기)하기

○ 수영강습 시 체육지도자는 강습생과 2m(최소 1m) 이상 거리를 유지하고, 불가피한 경우에는 얼굴을 마주 보고 대화하지 않기

○ 수영복, 수경 및 수건 등 신체에 접촉하는 물품은 개인물품 사용 안내, 공용물품 제공 시에는 소독 철저히 하기

○ 수영장 내(물 속 포함) 다른 사람과 2m(최소 1m) 이상 간격을 유지하고 이용자

간 신체 접촉 및 대화 자제 안내하기

○ 탈의실(락커룸), 샤워실, 대기실 등 공용공간은 다른 사람과 거리 두기를 유지 할 수 있도록 강습 종료 시간 조정 등 적정 인원 관리하기

* 예시) 탈의실(락커룸) 내 개인 사물함(락커) 배정시 일정 간격을 띄워 배정 등

○ 탈의실(락커룸), 샤워실, 대기실 등 부대시설의 손이 자주 닿는 표면은 매일 1회 이상 철저히 소독하기

○ 체온유지실 등 밀폐된 공간은 수시로 환기하고 이용자 간 거리 유지가 가능하도록 한꺼번에 몰리지 않도록 조치하기

○ 일광용 의자(선베드), 파라솔 등 공용시설은 2m(최소 1m) 이상 간격이 유지 되도록 배치하기

○ 개인위생수칙 준수, 생활 속 거리 두기의 필요성 등에 대한 주기적으로 교육 및 홍보하기(현수막, 문자전광판, 안내방송 등)

○ 어린이통학버스 내 전원 마스크 착용, 거리 두기가 가능하도록 한 칸 띄워 앉고, 대화는 자제하며 탑승 전·후 소독 및 환기 실시하기

○ 체육지도자 및 수상안전요원은 수영장 내 질서유지 및 안전관리 철저히 하기

○ 수영조 욕수는 수질 기준을 준수하여 청결하게 관리하기

○ 매 1시간마다 수영조 안의 수영자를 밖으로 나오도록 하고, 욕수의 조절, 침전물의 유무 및 사고의 유무 등을 점검 후 수영자 입장하기

※ 시설 내 실내체육시설, 음식점·카페, 공중화장실 등 이용 시 해당 유형의 지침을 준용

2-13. 생활 속 거리 두기 지침 : 피시(PC)방

1 이용자

[공통사항]

○ 발열 또는 호흡기 증상(기침, 인후통 등)이 있거나 최근 14일 이내 해외여행을 한 경우 방문 자제하기

○ 다른 사람과 2m(최소 1m) 이상 거리 두기

○ 흐르는 물과 비누로 30초 이상 손을 씻거나 손 소독제로 손 소독하기

○ 기침이나 재채기를 할 때는 휴지, 옷소매로 입과 코 가리기

○ 침방울이 튀는 행위(노래부르기, 소리지르기 등)나 신체접촉(악수, 포옹 등) 자제하기

○ 실내 다중이용시설을 이용하는 경우 마스크 착용하기

○ 실외에서 2m 거리 유지가 안되는 경우 마스크 착용하기

[해당 유형 적용사항]

○ 좌석은 한 칸 띄워 앉기

○ 고위험군은 시설이용 자제, 불가피하게 방문할 경우 마스크 착용하기

* 고위험군: 65세 이상 어르신, 임신부, 만성질환자 등

○ 출입 시 증상 여부(발열, 호흡기 증상 등) 확인 및 명부(전자 또는 수기) 기록 관리 (4주 보관 후 폐기) 등 방역에 협조하기

2 책임자·종사자

[공통사항]

○ 방역관리자 지정 및 지역 보건소 담당자의 연락망을 확보하는 등 방역 협력체계 구축하기

○ 공동체 내 밀접 접촉이 일어나는 동일 부서, 동일 장소 등에 2~3명 이상의 유증상자가 3~4일 내에 발생 시 유증상자가 코로나19 검사를 받도록 안내하며, 유증상자가 추가 발생 시 보건소에 집단감염 가능성을 신고하기

○ 종사자가 발열 또는 호흡기 증상이 있는 경우 출근 중단 및 즉시 퇴근 조치하기

○ 사람 간 간격을 2m(최소 1m) 이상 거리 두기

○ 손을 씻을 수 있는 시설 또는 손 소독제를 비치하고, 손 씻기 및 기침예절 준수 안내문 게시하기

○ 자연 환기가 가능한 경우 창문을 상시 열어두고, 에어컨 사용 등으로 상시적으로 창문을 열어두기 어려운 경우 2시간마다 1회 이상 환기하기

○ 공용으로 사용하는 물건(출입구 손잡이, 키보드, 마우스 등) 및 표면은 매일 1회 이상 자주 소독하기

○ 고객(이용자)을 직접 응대하는 경우 마스크 착용하기

○ 발열 또는 호흡기 증상이 있거나 최근 14일 이내 해외여행을 한 경우 방문 자제 안내하기

○ 실내 다중이용시설을 이용하는 경우 마스크 착용 안내하기

○ 실외에서 2m 거리 유지가 안되는 경우 마스크 착용 안내하기

[해당 유형 적용사항]

○ 출입구 및 시설 내 곳곳에 휴지 및 뚜껑 있는 쓰레기통 비치하기

○ 개인 위생수칙 준수, 생활 속 거리 두기의 필요성 등에 대한 주기적 교육·안내하기

○ 좌석은 한 칸 띄워 앉도록 착석 안내하기

○ 고위험군은 시설이용 자제, 불가피하게 방문할 경우 마스크 착용 안내하기

* 고위험군: 65세 이상 어르신, 임신부, 만성질환자 등

○ 출입하는 사람에 대한 증상 여부(발열, 호흡기 증상 등) 확인 및 명부(전자 또는 수기)를 작성 관리(4주 보관 후 폐기)하기

2-14. 생활 속 거리 두기 지침 : 유흥시설

1 이용자

[공통사항]<br>○ 발열 또는 호흡기 증상(기침, 인후통 등)이 있거나 최근 14일 이내 해외여행을 한 경우 방문 자제하기<br>○ 다른 사람과 2m(최소 1m) 이상 거리 두기<br>○ 흐르는 물과 비누로 30초 이상 손을 씻거나 손 소독제로 손 소독하기<br>○ 기침이나 재채기를 할 때는 휴지, 옷소매로 입과 코 가리기<br>○ 침방울이 튀는 행위(노래부르기, 소리지르기 등)나 신체접촉(악수, 포옹 등) 자제하기<br>○ 실내 다중이용시설을 이용하는 경우 마스크 착용하기<br>○ 실외에서 2m 거리 유지가 안되는 경우 마스크 착용하기<br><br>[해당 유형 적용사항]<br>○ 유흥시설에서 머무르는 시간 최소화하기<br>○ 탁자 사이 간격을 2m(최소 1m) 두고 앉거나, 일행이 아닌 다른 사람들과 가급적 최대한 간격을 띄워 앉기<br>○ 가능한 서로 마주 보지 않고 한 방향을 바라보도록 앉기<br>○ 식사를 할 때는 대화 자제하기<br>○ 음식은 각자 개인 접시에 덜어 먹기<br>○ 술잔 돌리지 않기<br>○ 고위험군은 시설이용 자제, 불가피하게 방문할 경우 마스크 착용하기<br>* 고위험군: 65세 이상 어르신, 임신부, 만성질환자 등<br>○ 출입 시 증상 여부(발열, 호흡기 증상 등) 확인 및 명부(전자 또는 수기) 기록 관리 (4주 보관 후 폐기) 등 방역에 협조하기

2 책임자·종사자

[공통사항]

○ 방역관리자 지정 및 지역 보건소 담당자의 연락망을 확보하는 등 방역 협력체계 구축하기

○ 공동체 내 밀접 접촉이 일어나는 동일 부서, 동일 장소 등에 2~3명 이상의 유증상자가 3~4일 내에 발생 시 유증상자가 코로나19 검사를 받도록 안내하며, 유증상자가 추가 발생 시 보건소에 집단감염 가능성을 신고하기

○ 종사자가 발열 또는 호흡기 증상이 있는 경우 출근 중단 및 즉시 퇴근 조치하기

○ 사람 간 간격을 2m(최소 1m) 이상 거리 두기

○ 손을 씻을 수 있는 시설 또는 손 소독제를 비치하고, 손 씻기 및 기침예절 준수 안내문 게시하기

○ 자연 환기가 가능한 경우 창문을 상시 열어두고, 에어컨 사용 등으로 상시적으로 창문을 열어두기 어려운 경우 2시간마다 1회 이상 환기하기

○ 사람들이 자주 접촉하는 출입구 손잡이, 탁자, 의자 등은 매일 1회 이상 표면을 소독하기

○ 고객(이용자)을 직접 응대하는 경우 마스크 착용하기

○ 발열 또는 호흡기 증상이 있거나 최근 14일 이내 해외여행을 한 경우 방문 자제 안내하기

○ 실내 다중이용시설을 이용하는 경우 마스크 착용 안내하기

○ 실외에서 2m 거리 유지가 안되는 경우 마스크 착용 안내하기

[해당 유형 적용사항]

○ 계산 시 비대면 기기 또는 투명 가림막 등을 설치하는 등 방법으로 가급적 고객과 마주 보지 않도록 하기

○ 탁자 사이 간격을 가급적 2m(최소 1m) 이상 두거나 테이블 간에 투명 가림막 설치, 고정형 탁자 일부를 사용 금지 등 탁자 간에 거리를 두는 방법 마련하기

○ 의자를 한 방향 또는 지그재그로 배치하는 등 서로 마주 보지 않도록 노력하기

○ 대규모 행사 개최 자제하기

○ 개인위생수칙 준수, 생활 속 거리 두기의 필요성 등에 대해 주기적으로 종사자 교육 실시하기

○ 대기자 발생 시 번호표를 활용하거나 대기자 간 1m 간격을 두고 대기하도록 안내하기

○ 음식은 각자 개인 접시에 덜어 먹도록 개인 접시와 국자, 집게 등을 제공하기

○ 노래를 부르는 시설을 갖춘 경우에는 손님 변경 시 마다 마이크 덮개를 새것으로 교체하기

○ 고위험군은 시설이용 자제, 불가피하게 방문할 경우 마스크 착용 안내하기

* 고위험군: 65세 이상 어르신, 임신부, 만성질환자 등

○ 출입하는 사람에 대한 증상 여부(발열, 호흡기 증상 등) 확인 및 명부(전자 또는 수기)를 작성 관리(4주 보관 후 폐기)하기
